# Perioperative corticosteroid treatment impairs tumor-infiltrating dendritic cells in patients with newly diagnosed adult-type diffuse gliomas

**DOI:** 10.3389/fimmu.2022.1074762

**Published:** 2023-01-10

**Authors:** Claudia Carenza, Sara Franzese, Alessandra Castagna, Sara Terzoli, Matteo Simonelli, Pasquale Persico, Lorenzo Bello, Marco Conti Nibali, Federico Pessina, Paolo Kunderfranco, Clelia Peano, Simone Balin, Joanna Mikulak, Francesca Calcaterra, Raffaella Bonecchi, Benedetta Savino, Massimo Locati, Silvia Della Bella, Domenico Mavilio

**Affiliations:** ^1^ Department of Medical Biotechnologies and Translational Medicine, University of Milan, Milan, Italy; ^2^ Laboratory of Clinical and Experimental Immunology, IRCCS Humanitas Research Hospital, Rozzano, Milan, Italy; ^3^ Laboratory of Leukocyte Biology, IRCCS Humanitas Research Hospital, Rozzano, Milan, Italy; ^4^ Department of Biomedical Sciences, Humanitas University, Pieve Emanuele, Milan, Italy; ^5^ Department of Medical Oncology and Hematology, IRCCS Humanitas Research Hospital, Rozzano, Milan, Italy; ^6^ Unit of Oncological Neurosurgery, IRCCS Istituto Ortopedico Galeazzi, Milan, Italy; ^7^ Department of Oncology and Hemato-Oncology, University of Milan, Milan, Italy; ^8^ Department of Neurosurgery, IRCCS Humanitas Research Hospital, Rozzano, Milan, Italy; ^9^ Bioinformatics Unit, IRCCS Humanitas Research Hospital, Rozzano, Milan, Italy; ^10^ Institute of Genetic and Biomedical Research, UoS Milan, National Research Council, Rozzano, Milan, Italy; ^11^ Laboratory of Chemokine Biology, IRCCS Humanitas Research Hospital, Rozzano, Milan, Italy

**Keywords:** dendritic cells, brain tumors, perioperative corticosteroids, immune suppressive tumor microenvironment, single cell-RNA sequencing

## Abstract

**Introduction:**

Adult-type diffuse gliomas are malignant primary brain tumors characterized by very poor prognosis. Dendritic cells (DCs) are key in priming antitumor effector functions in cancer, but their role in gliomas remains poorly understood.

**Methods:**

In this study, we characterized tumor-infiltrating DCs (TIDCs) in adult patients with newly diagnosed diffuse gliomas by using multi-parametric flow cytometry and single-cell RNA sequencing.

**Results:**

We demonstrated that different subsets of DCs are present in the glioma microenvironment, whereas they are absent in cancer-free brain parenchyma. The largest cluster of TIDCs was characterized by a transcriptomic profile suggestive of severe functional impairment. Patients undergoing perioperative corticosteroid treatment showed a significant reduction of conventional DC1s, the DC subset with key functions in antitumor immunity. They also showed phenotypic and transcriptional evidence of a more severe functional impairment of TIDCs.

**Discussion:**

Overall, the results of this study indicate that functionally impaired DCs are recruited in the glioma microenvironment. They are severely affected by dexamethasone administration, suggesting that the detrimental effects of corticosteroids on DCs may represent one of the mechanisms contributing to the already reported negative prognostic impact of steroids on glioma patient survival.

## Introduction

Gliomas represent 75% of malignant primary brain tumors in adults, and still remain among the most difficult cancers to treat (1). Their severity relies on a combination of histological features and signature molecular genetic alterations. According to the increasingly recognized role of molecular markers in predicting clinical behavior, the classification of gliomas is rapidly changing. The 2021 WHO classification of central nervous system tumors subdivides adult-type diffuse gliomas into isocitrate dehydrogenase (IDH)-mutant astrocytoma, IDH-mutant and 1p/19q codeleted oligodendroglioma, and IDH-wildtype glioblastoma (2). Although all diffuse gliomas are highly infiltrative and resistant to therapy, IDH-wildtype glioblastomas are characterized by the worst prognosis, with most patients not surviving beyond a year despite standard of care treatment, which consists of maximal safe surgical resection followed by chemoradiation (3).

The urgent need for more efficacious treatments for patients with gliomas, together with the recent progresses of anticancer immunotherapies (4), has renewed the interest in developing novel immunotherapeutic approaches also for gliomas. In this regard, the use of immune checkpoint inhibitors and peptide vaccination have so far failed to improve the survival in these patients, likely because of the low immunogenicity and the highly immunosuppressive tumor microenvironment (TME) that characterize gliomas (5, 6). Among other immunotherapeutic approaches, dendritic cell (DC)-based immunotherapy represents a promising strategy to better control the clinical progression of gliomas (7, 8). Indeed, recent clinical trials demonstrated the ability of DC vaccination protocols to generate potent tumor-specific immune responses *in vivo* and partial benefit on overall and progression-free-survival rates (8). In order to further improve the efficacy of these immunotherapeutic protocols, next generation DC-based vaccines aim at exploiting specific DC subsets able to infiltrate gliomas and to prime/boost cytotoxic T cell-driven anti-cancer immunity (9, 10). Other developing strategies aimed at potentiating the effects of DCs in cancer immunotherapy include the use of DC vaccines in combination with other anticancer therapies, and the reprogramming of tumor-infiltrating DCs towards the promotion of tumor rejection (9, 11, 12). In order to achieve these goals for the treatment of gliomas, a precise characterization of glioma-infiltrating DC subsets, their activatory/tolerogenic profile, and the molecular mechanisms involved in glioma-induced DC tolerogenicity is needed.

DCs are a heterogenous population of professional antigen presenting cells (APCs) that play a central role in the activation and regulation of all immune responses (13). DC-lineage DCs are subdivided into plasmacytoid DCs (pDCs) and conventional DCs (cDCs), which are further divided into cDC1 and cDC2 subsets. pDCs are endowed with the ability to produce high amounts of type I interferon (IFN) in response to viral infections, but in resting conditions they are mainly tolerogenic. Therefore, pDCs in the TME can contribute to tumor-specific tolerance and are associated with a bad prognosis (14). cDC1s are the most efficient DCs in priming cytotoxic T cells due to their high cross-presentation properties, and their presence in the TME is associated with better survival across several types of human cancers (15). cDC2s are mainly specialized in the activation of helper T cells that can be differentially polarized depending on the environmental conditions that sustain cDC2 activation (16). Further subsets of inflammatory DCs can also contribute to the overall shaping of antitumor immune responses exerted by DCs (13). They include monocyte-derived DCs (moDCs), which are rare in human peripheral tissues at the steady-state but rapidly increase during inflammation (13); and 6-sulfo-LacNAc (slan)DCs, which in the blood have a transcriptional profile overlapping with CD16+ non-classical monocytes but in peripheral tissues can acquire typical DC functions (17).

Beyond their belonging to one of these subsets, the behaviour of DCs depends also on their state of activation that is in turn affected by stimuli provided by the tissue microenvironment where DCs reside or are recruited. Upon exposure to inflammatory stimulation, DCs up-regulate the expression of MHC and costimulatory molecules, secrete pro-inflammatory cytokines, and present antigens to T cells in fully stimulatory conditions. On the other hand, DCs exposed to immunosuppressive environment express low levels of MHC and costimulatory molecules, up-regulate the expression of inhibitory molecules, secrete immunosuppressive cytokines, and present antigens to T cells in tolerogenic conditions (18). Accordingly, in cancer patients DCs are affected by the TME that undergoes profound changes during cancer progression (19, 20). While in the initial stages of cancer DCs activate robust tumor-specific cytotoxic T cells (11), during cancer progression DCs contribute to the tumor escape from immune surveillance by promoting tumor-specific immune tolerance and the development of an immunosuppressive TME (20).

The identification of DC subsets in the TME, together with the characterization of their activatory/tolerogenic profile, has been hampered so far by the low number of DCs in the TME and the lack of DC-specific markers. The recent implementation of high-dimensional single-cell technologies is making possible to define DC features at an unprecedented definition, both at the phenotypic and transcriptomic levels. Accordingly, DCs have started to be deeply characterized in the TME of different types of tumors, providing evidence that tumor immune evasion involves crippling normal DC functions, and that DC heterogeneity and states are conserved across various solid human cancers (21, 22). In the present study, we characterized peripheral blood DCs (PBDCs) and tumor-infiltrating DCs (TIDCs) in newly diagnosed adult-type diffuse glioma patients by using high-dimensional flow cytometry and single cell-RNA sequencing (scRNA-seq) approaches. Our results provide evidence that PBDCs are reduced in glioma patients, and that all subsets of DCs are recruited in the core lesions of glioma but they are functionally impaired. We also observed that the most dramatic reduction and functional impairment of DCs is evident in glioma patients undergoing perioperative steroid treatment to control peritumoral edema.

## Methods

### Study participants and ethics approval

The study was conducted on 27 newly diagnosed, non-relapsing adult patients with diffuse glioma undergoing surgical resection at the unit of Neuro-Oncology of Humanitas Research Hospital, Rozzano, Milan, Italy. Clinical patient information is provided in **Supplementary Table 1**. The study protocol was approved by the Institutional Review Boards of Humanitas Research Hospital (ONC-OSS-04-2017; 29/19), and written informed consents were provided by all participants before inclusion in the study in compliance with the Declaration of Helsinki. Twelve age- and sex-matched healthy volunteers were included as controls.

### Sample processing and staining

Peripheral blood samples were collected from patients and controls in K2 EDTA BD vacutainer tubes (BD Diagnostics, Franklin Lakes, NJ, USA) and stained with an 18-color DC-dedicated flow cytometry panel of monoclonal antibodies (mAbs) as previously reported (23). 500 μL of whole blood were incubated with ammonium chloride (ACK, Ammonium chloride 0.83% w/v, Potassium Bicarbonate 0.1% w/v, Titriplex 0.004% w/v, Merck KGaA) to lyse erythrocytes and samples were stained with Fixable Viability Stain 780 (BD Biosciences), then washed and stained with the combination of mAbs listed in **Supplementary Table 2**. Staining conditions for each mAb were preliminarily determined in titration assays, as previously described (24).

Brain tissue samples obtained during surgery were collected, stored at 4°C in supplemented Dulbecco’s Modified Eagle Medium (DMEM) high glucose (Lonza) added with 1% Penicillin/Streptomycin and 1% L-Glutamine and digested within 2 hours from excision with type IV Collagenase (1.6 mg/mL) (Merck KGaA) and type I DNase (0.4 mg/mL) (Merck KGaA) in Roswell Park Memorial Institute (RPMI) 1640 medium (Euroclone SpA) at 37°C for 1 hour. Homogenates were then smashed on a 70 μm filter (BD Biosciences), washed with RPMI with the addition of 2% fetal bovine serum (FBS) (Lonza), and collected in 50 mL collection tubes. Samples were then centrifuged at 290 rcf for 7 min, and the pelleted cells were incubated for 2 min with 1 mL of ACK 1X to lyse erythrocytes. Samples were then washed with FACS buffer (Hank’s Balanced Salt Solution, HBSS, w/o Ca^2+^ and Mg^2+^, Lonza, with the addition of 2% FBS), and centrifuged at 290 rcf for 7 min. The samples were then incubated with FACS buffer and Myelin Removal Beads II (Milteny Biotec) and passed through LS Columns (Milteny Biotec) according to manufacturer’s instructions. The samples were stained with the same DC-dedicated flow cytometry panel used for peripheral blood samples.

### Flow cytometry data acquisition and analysis

All data were acquired on a FACSymphony™ A5 flow cytometer (BD Biosciences). Flow Cytometry Standard (FCS) 3.0 files were imported into FlowJo software version 9.9.6 (FlowJo LLC), and data were compensated by using single-stained antibody-capture beads (CompBeads, BD Biosciences) as previously described (23–25). These data were analyzed by standard gating strategy, as previously reported (14, 23). Briefly, gated on single, live CD45^+^ (PB samples) or CD45^br^ (tissue samples) mononuclear cells, DC-lineage DCs were identified within the gate of lineage (CD3, CD19, CD20, CD56)^−^/CD14^−^/CD16^−^/HLA-DR^+^ cells. Gated on these cells, pDCs were identified as CD123^+^/CD11c^−^ cells; cDCs were identified as CD11c^+^/CD123^−^ cells, and further divided into cDC1s and cDC2s based on the expression of CD141 and CD1c, respectively. Inflammatory DCs were identified as lin^−^/HLA^-^DR^+^/CD11c^+^ cells that could be positive or negative for CD14 and CD16 expression. They included slanDCs that expressed M-DC8, and moDCs that expressed CD1a. The activation state of each DC subset was examined by assessing the expression of the activatory molecules CD40, CD80 and CD86, and the inhibitory molecules PD-L1, ILT2 and TIM-3. The compensated data were further imported into FlowJo software version 10.7.1 and visualised with a uniform manifold approximation and projection (UMAP). For the UMAP analysis, 2 different concatenated files were created, containing the same number of live CD45^+^/lin^-^/HLA-DR^+^ cells derived respectively from whole blood of untreated patients (n=12) and whole blood of dex-treated patients (n=11). A unique computational barcode was assigned to each concatenated file. These files were then concatenated in a single file for further visualization in UMAP dot plots (distance function: Euclidean; nearest neighbours: 15; minimum distance: 0.5), based on the expression of the following markers: CD45, CD14, CD16, HLA-DR, CD11c, CD123, CD141, CD1c, M-DC8, CD1a, CD40, CD80, CD86, PD-L1, ILT2, TIM-3. The same analysis was applied also to the cells derived from the tumor, where 2 different concatenated files were created, containing the same number of live CD45^br^/lin^-^/HLA-DR^+^ cells derived respectively from tumour tissue of untreated patients (n=5) and tumor tissue of dex-treated patients (n=3).

### ScRNA-seq data processing and analysis

Feature-barcode matrices generated by Savino et al. were down-loaded from Zenodo Repository, where the original data have been deposited (https://zenodo.org/record/6046299#.YgZ6bpbSKN4) and analyzed with R (v3.5.1) toolkit Seurat (v3.0.2). For each sample, Seurat objects were created from feature-barcode matrices. Cells containing > 200 genes and ≤ 10% mitochondrial genes were kept for downstream analysis. Gene expression matrices were then log-normalized with a scale factor of 10,000.

Datasets of each sample were integrated by Seurat data integration pipeline and CD45^+^ cells were subjected re-clustering, resulting in a total of 28 clusters (resolution level = 1.1). Cluster annotation was performed in silico using SingleR. The cell cluster enriched in DCs (cluster 19) was manually identified based on literature data obtained with scRNA-seq analyses of sorted DC subsets (26) and confirmed by using The Human Protein Atlas database (v20.1). The first 50 DEGs (p_adj_<0.05) of cluster 19 were then identified by using the ‘FindAllMarkers’ function in Seurat, with the parameter ‘test.use=wilcox’ used by default. The aggregated expression scores of these DEGs were calculated on single-cell base using the ‘AddModuleScore’ function in Seurat. The distribution of DC subsets across different clusters at resolution 0.5 was investigated by analyzing the expression of genes characteristics of classical DC subsets and other subsets recently described on the basis of their transcriptomic profile, including preDCs, migDCs, cDC2A and cDC2B (21, 27–32).

### Ingenuity pathway analysis

In order to investigate whether the cluster distribution of TIDCs may reflect DC functional state, we analyzed cell clusters at resolution 0.3 using IPA software program (Qiagen), which analyzes gene expression patterns using a built-in scientific literature-based database. DEGs that were characterized by p_adj_<0.01, and |log_2_FC|>0.58 were used for IPA analysis in the comparison between clusters 0 and 1, and between clusters 2 and 0. The *core analysis* function included in the software was performed on each cluster, applying the *immune cell* filter. DEGs were interrogated by Diseases and Functions (DFs) and Canonical Pathways (CPs) tools on IPA software. Only statistically significant DFs and CPs characterized by p<0.05 and |z-score|>1.5 were considered. Each gene identifier was mapped to its corresponding gene object in the Ingenuity Pathway Knowledge Base (IPKB).

### Statistical analysis

Statistical analysis of flow cytometric results was performed using GraphPad Prism software, version 9.0.0. The normal distribution of data was tested by using Shapiro-Wilk’s test. The t-test was used for comparisons between samples. All statistical analyses assumed a two-sided significance level of 0.05.

## Results

### DC-lineage DC subsets are decreased in the blood of patients with diffuse glioma.

**Figure 1:**
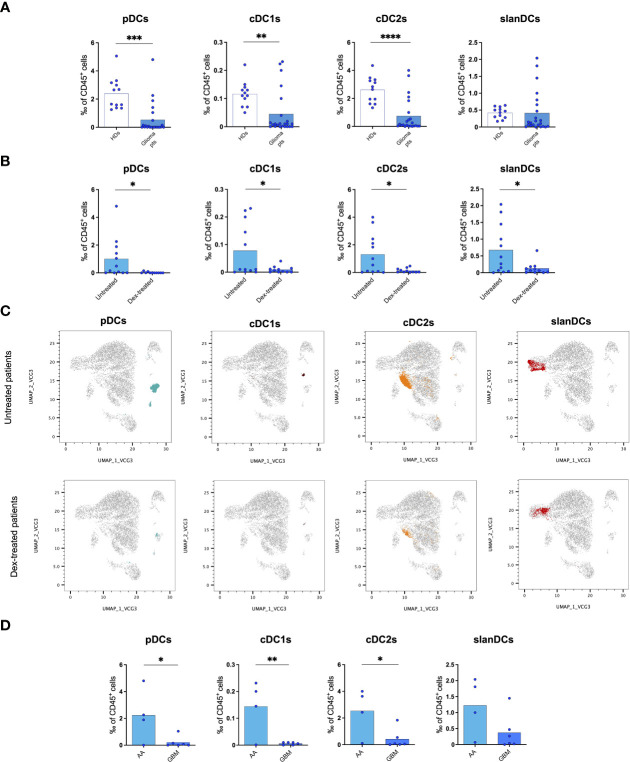
Flow cytometric analysis of PBDC subsets showing a reduction of circulating DCs in glioma patients. **(A)** Frequency of PBDC subsets in healthy donors (HDs, n=12) and glioma patients (Glioma pts, n=23). **(B)** Frequency of PBDC subsets in glioma patients either untreated (Untreated, n=12) or treated with dexamethasone (Dex-treated, n=11). Data expressed as per-thousand (‰) of CD45^+^ cells. Each symbol represents a single sample. In each series, the mean is shown. *p<0.05, **p<0.01, ***p<0.001, ****p<0.0001, calculated using the t-test. **(C)** UMAP plots showing the clustering of PBDC subsets in untreated and dex-treated glioma patients. Each plot shows a single DC subset as identified with manual gating strategy. Viable circulating CD45^+^/lin^−^/HLA-DR^+^ cells of down-sampled, concatenated files obtained from all glioma patients are shown in gray. pDCs are highlighted in dark turquoise, cDC1s in brown, cDC2s in orange, slanDCs in red. **(D)** Frequency of PBDC subsets in untreated IDH-wildtype glioma patients stratified based on histopathological diagnosis (anaplastic astrocytoma: AA, n=4; glioblastoma: GBM, n=6).

We first analysed PBDCs by using a high-dimensional flow cytometry panel that allows the identification of five distinct DC subsets, namely pDCs, cDC1s, cDC2s, slanDCs, and moDCs (20, 23). Our results showed that the frequency of all subsets of DC-lineage PBDCs were significantly decreased in glioma patients compared with controls (**Figure 1A**). Among inflammatory DCs, slanDCs did not significantly differ in glioma patients compared with controls. moDCs were almost undetectable in all blood samples, as expected (23). Similar results were observed when the absolute count of PBDC subsets was considered.

In order to investigate whether the reduction of PBDCs was associated with perioperative steroid treatment, we analysed PBDC subsets in our glioma patients stratified according to dexamethasone administration (dex-treated *vs* untreated patients). The frequency of all circulating DC subsets, including pDCs, cDC1s, cDC2s and slanDCs, was significantly lower in dex-treated compared with untreated patients (**Figure 1B**). Similar results were observed when the absolute count of PBDC subsets was considered. PBDC reduction in dex-treated patients was even more evident when DC subsets were visualized in UMAP plots of viable CD45^+^/lin^-^/HLA-DR^+^ cells obtained from down-sampled and concatenated files of all blood samples of dex-treated and untreated glioma patients (**Figure 1C**).

According to the WHO 2021 classification of primary brain tumors, the majority of our patients subjected to PBDC investigation were affected by glioblastoma IDH-wildtype, the glioma group that accounts for all IDH-wildtype gliomas independently from histopathological diagnosis, and all labelled as WHO grade 4. However, a certain proportion of our patients belonging to this group had a histopathological diagnosis of anaplastic astrocytoma, which in the previous classification (WHO 2016) was labelled as WHO grade 3. In order to investigate whether the reduction of PBDCs was associated with the histopathological diagnosis of gliomas, we analysed PBDC subsets in untreated patients (to avoid the confounding effect of dexamethasone) further stratified according to their histopathology and observed that, among patients with IDH-wildtype gliomas, the frequency of circulating pDCs, cDC1s and cDC2s was significantly lower in patients with a histopathological diagnosis of glioblastoma compared with those with anaplastic astrocytoma (**Figure 1D**).

Finally, we investigated the state of activation of PBDCs, and observed that the expression of the activation markers HLA-DR, CD40, CD80 and CD86, and inhibitory molecules PD-L1, ILT2 and TIM-3 on DC subsets did not differ between glioma patients and healthy donors, nor among patients stratified according to dex-treatment or histological diagnosis (data not shown).

### All subsets of myeloid DCs infiltrate glioma lesions, whereas they are absent in tumor-free brain parenchyma

**Figure 2:**
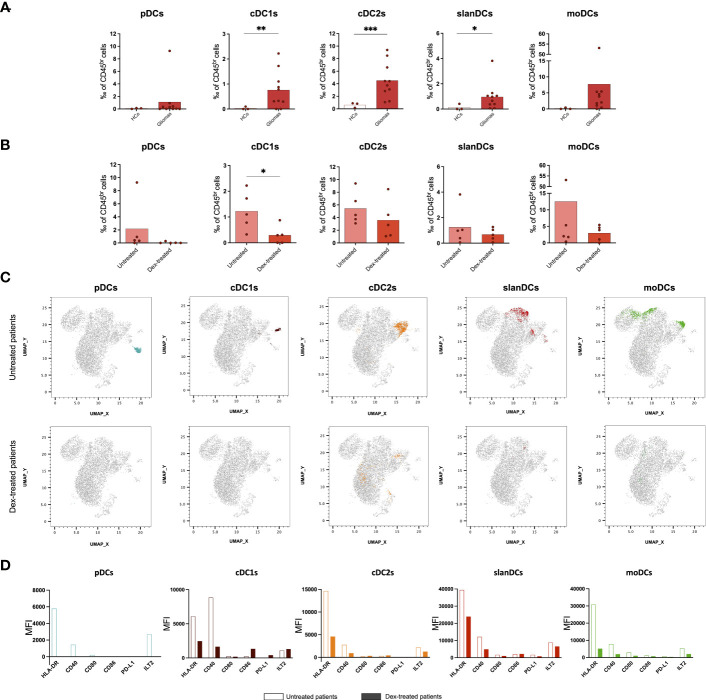
Flow cytometric analysis of TIDC subsets showing that perioperative corticosteroid treatment inhibits intratumoral DC recruitment and activation. **(A)** Frequency of DC subsets in healthy tissues (heathy controls: HCs, n=3) and tumor tissues (Gliomas, n=10) obtained from glioma patients. **(B)** Frequency of TIDC subsets in glioma patients either untreated (Untreated, n=5) or treated with dexamethasone (Dex-treated, n=5). Data expressed as per-thousand (‰) of CD45^br^ cells. Each symbol represents a single sample. In each series, the mean is shown. *p<0.05, **p<0.01, ***p<0.001, calculated using the t-test. **(C)** UMAP plots showing the clustering of TIDC subsets in untreated and dex-treated glioma patients. Each plot shows a single DC subset as identified with manual gating strategy. Viable tumor-infiltrating CD45^br^/lin^−^/HLA-DR^+^ cells of down-sampled, concatenated files obtained from all glioma patients are shown in gray. pDCs are highlighted in dark turquoise, cDC1s in brown, cDC2s in orange, slanDCs in red, and moDCs in green. **(D)** Expression of HLA-DR, activatory molecules (CD40, CD80, CD86), and inhibitory molecules (PD-L1, ILT2) on each DC subset, expressed as MFI measured on concatenated files, and compared between untreated and dex-treated glioma patients.

We then investigated the presence of TIDCs in glioma lesions by using the same flow cytometric approach used for their circulating counterparts. Three samples of healthy brain tissues obtained from patients affected by gliomas were included as controls. Our results showed that whereas the presence of all DC subsets was negligible in tumour-free brain parenchyma, cDC1s, cDC2s and the inflammatory slanDCs and moDCs, were abundant in the tumor infiltrate of glioma patients, without differences related to tumor histomolecular features. pDCs were detected only in one untreated glioblastoma, IDH-wildtype patient (**Figure 2A**). When assessing the impact of perioperative steroid treatment on TIDCs, we observed that dex-treated patients showed an overall reduction of TIDCs that was significant in the case of cDC1s, the DC subset with a prominent role in anti-tumor immunity (15) (**Figure 2B**). These results were even more evident in the UMAP plots of viable CD45^br^/lin^-^/HLA-DR^+^ cells obtained from down-sampled and concatenated files of all tissue samples (**Figure 2C**).

Because DCs were negligible in tumor-free brain tissue, a comparison of DC phenotype between tumor and healthy brain was not possible. In order to investigate whether the state of activation of TIDCs was affected by perioperative steroid treatment, we also compared the expression of HLA-DR, the costimulatory molecules CD40, CD80 and CD86, and the immune checkpoints PD-L1 and ILT2 on each DC subset between dex-treated and untreated patients. Because of the low number of TIDCs, the analysis was performed on concatenated files of glioma samples. As shown in **Figure 2D**, we observed that tumor-infiltrating cDC1s, cDC2s, slanDCs and moDCs obtained from dex-treated patients showed a lower expression of HLA-DR and CD40 compared with untreated patients. Dex-induced immunophenotypic changes of pDCs could not be assessed because, as reported above, pDCs were negligible in the tumor infiltrate of dex-treated patients. The expression of the inhibitory molecule TIM-3 could not be assessed on TIDCs, because TIM-3 is cleaved by the collagenase treatment used for glioma tissue processing, as already reported (20).

### ScRNA-seq analysis reveals distinct clusters of TIDCs in glioma lesions

**Figure 3:**
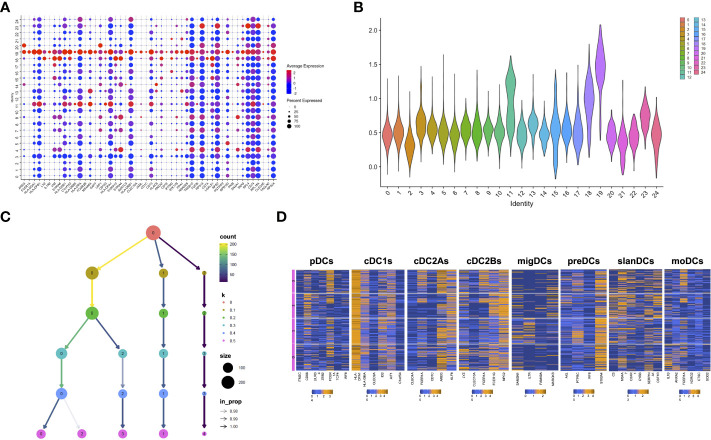
scRNAseq confirms the presence and heterogeneity of TIDCs. **(A)** Dot plot showing the first 50 DEGs (p_adj_<0.05) between cluster 19 and all the other clusters that compose CD45^+^ cells obtained from 7 tumor tissues and 2 healthy brain tissues from 8 glioma patients. All 50 genes are known to be enriched or expressed by human DCs, thus indicating that cluster 19 is the cluster of DCs. Color scale indicates the average expression level of genes; dot size indicates the percentage of gene-expressing cells in each cluster. **(B)** By applying the AddModuleScore function that allows to compare the expression of a specific set of genes among clusters, the expression of the 50-gene signature characterizing cluster 19 was visualized in a violin plot. **(C)** Reclustering of cluster 19 represented in a clustering tree based on kk-means. Nodes colored according to the value of k and sized according to the number of cells they represent. Edges colored according to the number of cells (from blue representing few to yellow representing many). Cluster labels are randomly assigned by the kk-means algorithm. **(D)** Heatmaps showing the mean expression of genes characteristic of pDCs, cDC1s, cDC2As, cDC2Bs, preDCs, migDCs, slanDCs, and moDCs, in clusters from 0 to 3 at resolution 0.5. Expression values are zero-centered and scaled for each gene. Each gene name is reported on the bottom of each heatmap.

After having demonstrated the presence of DCs in glioma core lesions, we characterized their molecular and functional features by analyzing their transcriptomic profile. To this aim, we analyzed scRNA-seq data generated from CD45^+^ cells isolated from 7 core glioma lesions and 2 healthy brain tissue samples obtained from 8 different adult-type diffuse glioma patients, available in Zenodo Repository (https://zenodo.org/record/6046299#.YgZ6bpbSKN4). The Seurat integration procedure was used to remove batch effects. Based on their transcriptomes, unsupervised graph-based clustering partitioned 36,237 cells into 28 distinct clusters. Clusters 25, 26 and 27 were filtered-out because of their small size (less than 20 cells) and excluded from the analysis. We identified cluster 19 as the putative cluster of DCs based on previously reported DC transcriptomic signatures (27). In order to confirm the DC annotation of cluster 19, we selected the first 50 differentially expressed genes (DEGs) between cells included in this cluster and all the others (padj<0.05) (**Figure 3A**). Based on the information available in the human Blood Atlas (www.proteinatlas.org), we verified that all the 50 DEGs composing the signature were expressed by human DCs and, in particular, 22 of them were enriched in myeloid and/or plasmacytoid DCs (**Supplementary Table 3**). We then applied to the 50-gene signature the AddModuleScore function from Seurat package, which allows to compare the expression of a specific set of genes among different clusters, and we visualized the expression of this signature on a violin plot (**Figure 3B**). Taken together, these data confirmed that cluster 19 was the one containing DCs.

To investigate TIDC heterogeneity, we then performed a reclustering of cluster 19, and compared different clustering results for each resolution parameter, from 0 to 0.5. At resolution 0.1, we observed the formation of three main branches, one of which continuing to split up to the resolution 0.5 (**Figure 3C**). The smallest cluster, stable at resolutions from 0.1 to 0.5, was filtered-out because of its small size and excluded from subsequent analyses. We then focused our analyses on the remaining 4 clusters observed at resolution 0.5. In particular, in order to investigate whether they reflected the distribution of DCs in different subsets, we examined the expression of genes characteristic of DC subsets recently described on the basis of their transcriptomic profiles. Beyond the DC subsets that we investigated by flow cytometry (namely, pDCs, cDC1s, cDC2s, slanDCs, moDCs), they include preDCs, migratory DCs (migDCs) and the cDC2 subclusters A and B endowed with regulatory and pro-inflammatory properties, respectively (27). Our results confirmed that genes belonging to the gene signature of all DC subsets were indeed expressed by glioma TIDCs (**Figure 3D**). However, the expression of the genes characteristic of each DC subset was widely spread among the 4 clusters, indicating that none of the clusters of TIDCs corresponded to any defined DC subset. Notably, according to the lack of DCs observed by flow cytometry in healthy brain tissues, cells obtained from healthy brain samples were negligible, indicating that all DCs analysed for transcriptome profiling were derived from core glioma lesions.

### The largest cluster of TIDCs has a transcriptomic signature indicative of functional impairment

**Figure 4:**
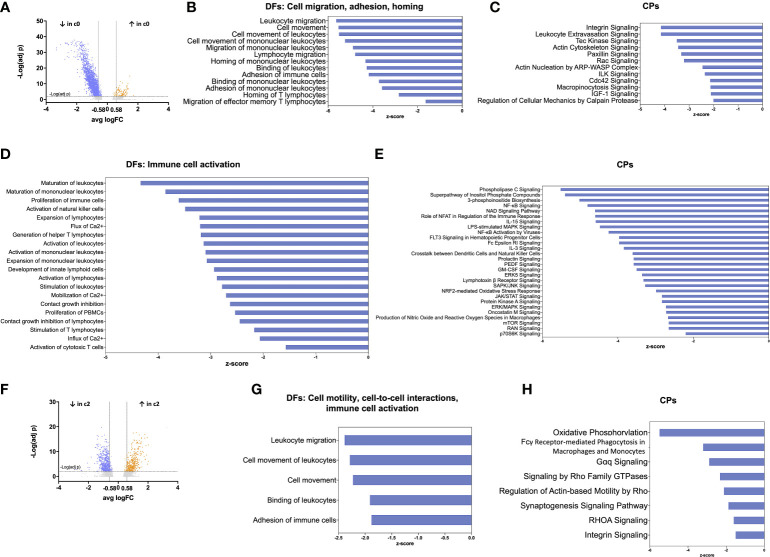
Functional annotation of TIDC clusters by IPA analysis reveals impairment of the largest cluster of DCs. **(A)** Volcano plot showing DEGs between cluster 0 (the largest cluster of TIDCs) and cluster 1, at resolution 0.3. Grey dots indicate genes that were not statistically significant (p_adj_>0.01); orange dots indicate significantly up-regulated genes (with log_2_FC>0.58), and blue dots indicate significantly down-regulated genes (with log_2_FC<-0.58). **(B)** Bar plot showing DFs of sub-categories related to cell migration, adhesion and homing that were significantly down-regulated in cluster 0 compared with cluster 1. **(C)** Bar plot showing CPs related to DFs shown in **b** that were significantly down-regulated in cluster 0 compared with cluster 1. **(D)** Bar plot showing DFs related to immune cell activation that were significantly down-regulated in cluster 0 compared with cluster 1. **(E)** Bar plots showing CPs related to DFs shown in D that were significantly down-regulated in cluster 0 compared with cluster 1. **(F)** Volcano plot showing DEGs between cluster 2 (mostly composed of cells deriving from *dex-treated* patients) and cluster 0, at resolution 0.3. Grey dots indicate genes that were not statistically significant (p_adj_>0.01); orange dots indicate significantly up-regulated genes (with log_2_FC>0.58), and blue dots indicate significantly down-regulated genes (with log_2_FC<-0.58). **(G)** Bar plot showing DFs of sub-categories related to cell motility, cell-to-cell interactions, and immune cell activation that were significantly down-regulated in cluster 2 compared with cluster 0. **(H)** Bar plots showing CPs related to DFs shown in G that were significantly down-regulated in cluster 2 compared with cluster 0. In all the bar plots, the functions or pathways, listed on the left side of the plot, are ranked according to the z-score that predicts a down-regulation (blue, z-score <-1.5).

We further investigated whether the distribution of TIDC clusters in glioma may reflect different DC functional states, as similarly reported in human hepatocarcinoma (33). To this aim, we analysed cell clusters at resolution 0.3 by using the Ingenuity Pathway Analysis (IPA) software, an advanced bioinformatic tool that analyzes gene expression patterns using a built-in scientific literature-based database. We focused on the analysis of DEGs between the two largest clusters, namely clusters 0 and 1. Among 2309 DEGs between the two clusters, 2216 were down-regulated and 93 were up-regulated. By further setting a threshold on |log2FC|>0.58, corresponding to a 1.5-fold change, we selected 1935 down-regulated and 80 up-regulated DEGs in cluster 0 compared with cluster 1 (**Figure 4A**). These genes were used for IPA functional annotation, applying a filter on immune cells. In particular, we applied the Diseases and Functions (DFs) analytics tool to define cellular processes and biological functions predicted to be affected on the basis of relative gene expression changes, and the Canonical Pathways (CPs) tool to predict which pathways were affected. The directional changes in both analyses were predicted by z-score. The analysis of DEGs categorized by DFs indicated that 502 processes and functions were differentially regulated (p<0.05) between cluster 0 and 1. Among these processes and functions, 173 were down-regulated in cluster 0 (as defined based on z-score <-1.5) and only 3 were up-regulated (as defined based on z-score >1.5); the remaining functions lacked z-score, or had a z-score between -1.5 and +1.5 (**Supplementary Table 4**). The analysis of DEGs categorized by CPs indicated that 191 pathways were differentially regulated (p<0.05) between cluster 0 and 1. Among these pathways, 141 were down-regulated in cluster 0 and 5 were up-regulated (**Supplementary Table 5**). The results of IPA functional annotation most relevant to TIDC functions in glioma microenvironment are summarized in **Figures 4B–E**. In particular, the analysis of DEGs categorized by DFs indicated that, based on gene expression, a relevant number of processes and functions relative to cellular migration, adhesion and homing were down-regulated in cluster 0 compared with cluster 1 (**Figure 4B**). Consistent with this observation, CPs involved in cellular motility, cytoskeleton rearrangement and cell-to-cell interactions were similarly down-regulated in cluster 0 (**Figure 4C**). In order to gain more insights into the DEGs underlying the down-regulation of these functions and pathways in glioma TIDCs, we examined the DEGs composing the processes and functions reported in **Figure 4B** and the pathways reported in **Figure 4C**, and obtained a list of 163 genes (reported in **Supplementary Table 6**). Supporting the impairment of functions relevant to DC migration and homing, DEGs in this group included genes encoding chemokine receptors or other chemotactic receptors (e.g., CXCR4, SLAMF1, ADGRE5, PTGER4), molecules involved in cytoskeleton rearrangement relevant to cell motility (e.g., S1PR1, MYH9, AKIRIN1, FGD3), metalloproteinases (e.g., MMP7), integrins (e.g., ITGA1, ITGA4, ITGAL), and other adhesion molecules involved in cell-to-cell interactions (e.g., F11R, CD44). Moreover, the analysis of DEGs categorized by DFs also indicated that a high number of processes and functions involved in immune cell activation were down-regulated in cluster 0 compared with cluster 1 (**Figure 4D**). Consistent with these observations, CPs relative to receptor signalling, signal transduction, and cytokine-induced responses were significantly down-regulated in cluster 0 (**Figure 4E**). We then examined the DEGs composing the processes and functions reported in **Figure 4D** and the pathways reported in **Figure 4E** and obtained a list of 304 genes (reported in **Supplementary Table 7**). They included transcripts encoding molecules playing key roles in different steps of DC activation, including signal transduction pathways (e.g., JAK1, STAT4, and several molecules belonging to MAPK, PI and NF-kB pathways), endocytosis and phagocytosis (e.g., FNBP1, CLTC, RAB27A), antigen processing and presentation (e.g., ISG15, AKAP11, ATG5, HLA-DRB5), cytokines and cytokine receptors (e.g., TNFSF14, LTB, IL18R1), molecules involved in DC interactions with other immune cells (e.g., SLAMF6, LY9, CYTIP). These 304 DEGs also included genes involved in cell metabolism and cell proliferation (e.g., BRAF, PIM1, KRAS).

### A cluster of TIDCs mainly derived from dex-treated patients has a transcriptomic signature suggestive of further functional impairment

We then focused on the analysis of DEGs between clusters 0 and 2, both originating from the splitting of one single cluster. We observed that cluster 2 was mainly composed of cells deriving from dex-treated patients (78%), whereas these cells were a minority (11%) in cluster 0. Among 967 DEGs between the two clusters, 576 were down-regulated and 391 were up-regulated. By further setting a threshold on |log2FC|>0.58 (corresponding to a 1.5-fold change), we selected 531 down-regulated and 362 up-regulated DEGs in cluster 2 compared with cluster 0 (**Figure 4F**). These genes were used for IPA functional annotation. The analysis of these genes, categorized by DFs, indicated that 81 processes were differentially regulated between cluster 2 and 0 (**Supplementary Table 8**). Among these processes, 8 were down-regulated in cluster 2 compared with cluster 0, whereas the remaining processes lacked z-score, or had a z-score between -1.5 and +1.5. Relevant to TIDC functions in glioma microenvironment, DFs down-regulated in cluster 2 included processes related to cellular motility and cell-to-cell interactions (**Figure 4G**). The analysis of DEGs categorized by CPs indicated that 83 pathways were differentially regulated between cluster 2 and cluster 0 (**Supplementary Table 9**). Relevant to TIDC functions in glioma microenvironment, down-regulated CPs in cluster 2 included pathways crucial to signalling, cell-to-cell interactions and phagocytosis (**Figure 4H**). According to the functions and pathways down-regulated in cluster 2, the 74 DEGs composing the processes and functions reported in **Figure 4G** and the pathways reported in **Figure 4H** included transcripts encoding molecules crucially involved in: DC activation and migration pathways (e.g. S100A10, CD63), endocytosis and phagocytosis (e.g. AP2S1, MYO1G, LRP1, FCER1G), antigen processing and presentation (e.g. CTSZ, CALR, LITAF, RAC1), cytokines and cytokine receptors (e.g. TNFSF12, IL4R), cytoskeleton rearrangement relevant to cell motility (e.g. PFN1, ARPC1A, ARPC1B), adhesion molecules involved in cell-to-cell interactions (e.g. ADAM9, GAS6) (**Supplementary Table 10**). They also included genes involved in cell metabolism and cell proliferation (e.g. G6PC3, SMPD2, CREB3L4, RPS6KB2). As expected, taking into consideration that cluster 2 was mainly composed of cells deriving from dex-treated patients, genes involved in stabilization of glucocorticoid receptor (HSPA1A, HSPA1B) were up-regulated in cluster 2 compared with cluster 0.

## Discussion

In this study, we performed a deep characterization of PBDCs and TIDCs in patients with newly diagnosed adult-type diffuse glioma and demonstrated that both the tumor and corticosteroid therapy have profound effects on DCs.

We observed that both cDCs and pDCs are reduced in the blood of glioma patients. These results are in partial agreement with previous studies that reported discordant results, indeed, including reduced, unchanged and increased cDCs and/or pDCs in glioma patients, likely related to different criteria used for patient selection (34, 35). As suggested in other types of cancer, the reduction of PBDCs in our patients may be sustained partly by DC recruitment into the tumor microenvironment, and partly by tumor-derived cytokines, such as VEGF and IL-6 that are produced by glioma cells (26, 36) and inhibit DC maturation in the bone marrow (37). Because only part of the patients enrolled in our study underwent perioperative dexamethasone treatment, we had the opportunity to investigate the impact of corticosteroids on PBDCs in glioma patients. We observed indeed that, compared with untreated patients, dex-treated patients had a significant and marked reduction of all PBDC subsets, thus confirming the high sensitivity of circulating DCs to systemic corticoid administration reported in other settings (38, 39). Notably, we further observed that, among untreated patients with IDH-wildtype gliomas, the reduction of circulating DC-lineage DCs was more marked in patients with a histopathological diagnosis of glioblastoma compared with patients with a histopathological diagnosis of anaplastic astrocytoma. This observation is relevant to the consideration that in several human cancers a more marked PBDC reduction has been described in patients with more advanced disease, possibly related to higher tumor secretion of soluble factors affecting DC generation and distribution (40–44). Although the 2021 WHO classification of central nervous system tumors include all IDH-wildtype diffuse gliomas in the most severe group of glioblastomas independently from their histopathological features^2^, it is not yet clear if astrocytomas with molecular but not histopathological features of glioblastomas have exactly the same overall biology and response to treatment as IDH-wildtype gliomas with overt necrosis and/or microvascular proliferation (45). Indeed, our results demonstrating that PBDC counts differ in IDH-wildtype glioma patients stratified based on histopathological diagnosis may suggest that the histopathological grade of these tumors still affects their overall impact on the immune system.

When we moved to the characterization of tissue DCs, first of all we observed that DCs were negligible in healthy brain samples, thus demonstrating the lack of parenchymal DCs in healthy human brain. This finding represents a novelty because the current knowledge on the role of DCs in the central nervous system has been acquired in murine models, so far, showing that DCs in healthy mouse brains are present only in the choroid plexus and in the meninges but not in the brain parenchyma (46).

In our study we further observed that all subsets of DCs were recruited in the core lesions of diffuse gliomas. Notably, this was observed in all patients independently from tumor histomolecular features, indicating that also the most severe type of gliomas retains the ability to recruit DCs in the TME. This observation may provide a possible explanation to the high susceptibility of gliomas to DC vaccines (7, 8), and may suggest the feasibility of targeting TIDCs in these patients with DC reprogramming immunotherapeutic strategies. Notably, the presence of several DC subsets in IDH-wildtype glioblastoma lesions has also been reported by Pombo Antunes and colleagues in a recent study addressing single-cell profiling of myeloid cells by scRNA-seq and cellular indexing of transcriptomes and epitopes (CITE)-seq approaches (47). Indeed, patients with either newly diagnosed or recurrent disease were enrolled, and this fact allowed the observation that TIDCs were far more abundant in recurrent patients. As a consequence, the analysis of TIDCs in Pombo Antunes’ study was performed primarily on recurrent tumors, demonstrating the presence not only of cDC1s, cDC2s and pDCs, but also of more recently identified DC subsets, including cDC2A and cDC2B subtypes, migDCs, and preDCs (47). In this respect, our study confirms and extends these observations, by demonstrating the presence of these same DC subsets in primary tumors, at the immunophenotypic and/or transcriptomic level. In our study we further investigated the functional state of TIDCs. By performing IPA functional annotation that predicts affected cellular functions and pathways based on gene expression, we demonstrated that the most abundant cluster of TIDCs in gliomas was characterized by a transcriptomic signature suggestive of functional impairment. In particular, cellular processes crucial to the primary function of DCs in cancer immunity, namely capturing tumor antigens, migrating to lymph nodes, and activating T cell responses, all resulted down-regulated in the largest cluster of TIDCs. Among the down-regulated genes most relevant to DC functions, we identified CLTC that encodes clathrin, and RAB27A that encodes Rab27a, two molecules that play a key role in DC endocytosis and phagocytosis, respectively (48, 49). The same negative regulation was observed for SLAMF1, a gene encoding the polyfunctional molecule SLAM that, by triggering Nox2 activation, positively regulates DC migration to draining lymph nodes (50). The most abundant cluster of TIDCs was also characterized by a down-regulation of ITGA4 and ITGAL, encoding the integrin-a4 and integrin-aL chains, respectively. These two molecules had been reported as positive prognostic factors in breast cancer (51), likely because of their ability to sustain immune cell infiltration in the tumor, and their role in the formation of the immunological synapses needed for T cell activation. Also AKAP11, member of A-kinase anchoring proteins required for optimal antigen presentation by DCs (52) and ATG5, a key autophagy gene needed for optimal phagosome-to-lysosome fusion and subsequent antigen processing and loading on MHC molecules (53), resulted down-regulated in the largest cluster of glioma TIDCs. Although the list of relevant down-regulated genes may be extended to a huge number of other genes controlling essential DC functions, it is evident from our study that, based on gene expression, a relevant proportion of DCs infiltrating glioma lesions are likely impaired in their ability to efficiently present tumor antigens and activate effective anti-tumor immune responses.

Notably, when we assessed the impact of perioperative corticosteroid treatment on TIDCs, we observed indeed that, compared with untreated glioma patients, dex-treated patients had a significant and marked reduction of tumor-infiltrating cDC1s, the subset most relevant to antitumor immune responses. According to the tolerogenic DC profile induced by dexamethasone *in vitro* (54), dex-treated patients showed an overall reduction in TIDC expression of HLA-DR and CD40 molecules. Moreover, the transcriptomic profile of the cluster enriched in TIDCs obtained from dex-treated patients was characterized by down-regulation of pathways and functions crucial to sustain the role of DCs in cancer immunity, including signal transduction pathways involved in cell activation, and processes involved in antigen presentation and cell migration. These findings are in line with previous studies that characterized the transcriptomic profile of tolerogenic DCs differentiated *in vitro* in the presence of dexamethasone, reporting a down-regulation of DEGs spanning functional families relevant to the ability of DCs to stimulate adaptive immune responses (55). Taken together, our experimental evidence indicates that perioperative steroid treatment reduces the amount and impairs the activity of TIDCs in glioma patients, thus suggesting that these detrimental effects of steroids on DCs may represent one of the mechanisms contributing to the already reported negative prognostic impact of steroids on glioma patient survival (56).

In conclusion, in this study we demonstrated that gliomas have the potential to recruit different DC subsets into the tumor site, but these cells undergo phenotypic and transcriptomic profile changes suggestive of functional DC impairment. This evidence paves the way to the development of new therapeutic strategies aimed at reactivating *in situ* TIDCs and switching their behavior towards promotion of tumor rejection. Moreover, by demonstrating the detrimental effects of perioperative dexamethasone treatment on circulating and glioma-infiltrating DCs, the results of this study support previous clinical evidence that discourages the use of steroids in these patients, suggesting the use of alternative therapeutic strategies for the control of symptomatic peritumoral vasogenic cerebral edema (57).

## Data availability statement

The data underlying this article are available in Zenodo, at https://zenodo.org/record/6046299#.YgZ6bpbSKN4. The dataset was derived from sources in the public domain.

## Ethics statement

The studies involving human participants were reviewed and approved by Institutional Review Boards of Humanitas Research Hospital (ONC-OSS-04-2017; 29/19). The patients/participants provided their written informed consent to participate in this study.

## Author contributions

Data curation and Resources by CC, SF, AC, ST, MS, PP, LB, MN, FP, PK, CP, RB, BS, ML, SDB and DM. Conceptualization and Methodology by CC, SF, AC, ST, MS, PP, LB, MN, FP, PK, CP, RB, BS, ML, SDB and DM. Investigation by CC, SF, AC, ST, PK, CP, BS and Formal Analysis by CC, SF, AC, ST, SDB, PK, JM, FC. Writing – Original Draft by CC, SDB, DM. Writing – Review and Editing by CC, SF, AC, ST, MS, PP, LB, MN, FP, PK, CP, SDB, JM, FC, RB, BS, ML, SDB, DM. Funding Acquisition by ML, DM, SDB. Supervision by MS, LB, ML, SDB, DM. All authors contributed to the article and approved the submitted version.
