# Supplementary material for: Perioperative corticosteroid treatment impairs tumor-infiltrating dendritic cells in patients with newly diagnosed adult-type diffuse gliomas
Source: Front Immunol. 2023 Jan 10;13:1074762. doi: 10.3389/fimmu.2022.1074762 (PMC9872516; doi:10.3389/fimmu.2022.1074762)
Supplement: Supplementary file 1 [file DataSheet_1.pdf]

Supplementary Table 1. Clinical features of patients and controls.

	**donors of healthy control samples**					***Untreated glioma patients***					***dex-treated* glioma patients**				
**assay**	**n**	**M:F**	**mean age [range]**	**WHO 2021 diagnosis**	**histological diagnosis**	**n**	**M:F**	**mean age [range]**	**WHO 2021 diagnosis**	**histological diagnosis**	**n**	**M:F**	**mean age [range]**	**WHO 2021 diagnosis**	**histological diagnosis**
PBDCs by flow cytometry	12	7:5	47<br>[25-69]		healthy donors	12	7:5	54,6<br>[36-74]	astrocytoma, IDH mutant (n=2)<br>glioblastoma, IDH wildtype (n=10)	anaplastic astrocytoma (n=1)<br>glioblastoma (n=1)<br>anaplastic astrocytoma (n=4)<br>glioblastoma (n=6)	11	6:5	61,5<br>[43-74]	astrocytoma, IDH mutant (n=1)<br>glioblastoma, IDH wildtype (n=10)	anaplastic astrocytoma (n=1)<br>glioblastoma (n=10)
TIDCs by flow cytometry	3	2:1	59,3<br>[53-69]	ganglioglioma (n=1)<br>astrocytoma, IDH mutant (n=1)<br>glioblastoma, IDH-wildtype (n=1)	healthy tissue	5	3:2	52,4<br>[41-71]	astrocytoma, IDH mutant (n=1)<br>glioblastoma, IDH wildtype (n=4)	anaplastic astrocytoma (n=1)<br>anaplastic astrocytoma (n=1)<br>glioblastoma (n=3)	5	2:3	64<br>[48-74]	oligodendroglioma, IDH-mutant and 1p/19q-codeleted<br>glioblastoma, IDH wildtype (n=4)	anaplastic oligodendroglioma (n=1)<br>glioblastoma (n=4)
TIDCs by scRNA-seq	2	1:1	53<br>[53-53]	glioblastoma, IDH-wildtype (n=2)	anaplastic astrocytoma	5	3:3	54,25<br>[41-71]	astrocytoma, IDH mutant (n=2)<br>glioblastoma, IDH wildtype (n=1)<br>glioblastoma, IDH wildtype (n=2)	anaplastic astrocytoma (n=2)<br>anaplastic astrocytoma (n=1)<br>glioblastoma (n=2)	2	1:1	68<br>[67-71]	glioblastoma, IDH wildtype (n=2)	glioblastoma (n=2)

Supplementary Table 2. List of monoclonal antibodies and reagents used for the analysis of DC subsets by flow cytometry

Marker	Description	Fluorochrome	Clone	Company
Fixable Viability Stain 780	Viability marker	APC-Cy7		BD Biosciences
CD45	Leukocyte common antigen	AF700	HI30	BD Biosciences
CD3	Lineage marker—T cells	FITC	HIT3A	Biolegend
CD14	Lineage marker—monocytes	BV570	M5E2	Biolegend
CD16	Lineage marker—NK cells and granulocytes	BUV496	3G8	BD Biosciences
CD19	Lineage marker—B cells	FITC	4G7	BD Biosciences
CD20	Lineage marker—B cells	FITC	2H7	BD Biosciences
CD56	Lineage marker—NK cells	FITC	NCAM 16,2	BD Biosciences
HLA-DR	Major histocompatibility complex class II molecule	BUV661	G46-6	BD Biosciences
CD123	pDC marker	PE-Cy7	7G3	BD Biosciences
CD11c	cDC marker	PE-Cy5	B-ly6	BD Biosciences
CD141 (BDCA-3)	DC1 marker	BV605	M80	Biolegend
CD1c (BDCA-1)	cDC2 marker	BV421	L161	Biolegend
M-DC8 (anti-slan)	slanDC marker	APC	DD-1	Milteny Biotec
CD1a	moDC marker	BUV395	HI149	BD Biosciences
CD40	Costimulatory molecule	BV650	5C3	BD Biosciences
CD80	Costimulatory molecule	BV510	L307	BD Biosciences
CD86	Costimulatory molecule	BUV737	2331	BD Biosciences
CD274 (PD-L1)	Inhibitory molecule	PE-CF594	MIH1	BD Biosciences
CD85j (ILT2)	Inhibitory molecule	PE	GHI/75	Biolegend
CD366 (TIM-3)	Inhibitory molecule	BV711	7D3	BD Biosciences

Supplementary Table 3. DEGs between cluster 19 and other clusters.

Gene	RNA cell type specificity
AREG	Group enriched (eosinophil, basophil, NK-cell)
FCER1A	Cell type enriched (basophil)
HLA-DQA1	Cell type enhanced (myeloid DC)
HLA-DPB1	Cell type enhanced (myeloid DC)
LYZ	Cell type enhanced (classical monocyte, myeloid DC)
IL1R2	Cell type enriched (neutrophil)
VIM	Low cell type specificity
S100A6	Low cell type specificity
HLA-DQB1	Group enriched (myeloid DC, naive B-cell, memory B-cell, classical monocyte, intermediate monocyte, plasmacytoid DC)
C1orf162	Low cell type specificity
HLA-DRB5	Cell type enhanced (myeloid DC)
HLA-DRA	Group enriched (naive B-cell, memory B-cell, intermediate monocyte, classical monocyte, plasmacytoid DC, non-classical monocyte)
FCGR2B	Cell type enhanced (basophil, memory B-cell)
MS4A6A	Group enriched (classical monocyte, myeloid DC, plasmacytoid DC, intermediate monocyte, basophil)
GAPT	Cell type enriched (eosinophil)
LSP1	Low cell type specificity
CD163	Group enriched (classical monocyte, myeloid DC)
HLA-DPA1	Group enriched (myeloid DC, intermediate monocyte, memory B-cell, naive B-cell, non-classical monocyte, plasmacytoid DC, classical monocyte)
S100A10	Low cell type specificity
S100A4	Low cell type specificity
AKR1C3	Cell type enhanced (basophil, gdT-cell)
HLA-DRB1	Cell type enhanced (myeloid DC)
CLEC10A	Cell type enriched (myeloid DC)
JAML	Cell type enhanced (eosinophil)
CD1C	Group enriched (myeloid DC, memory B-cell)
CST3	Group enriched (myeloid DC, intermediate monocyte, classical monocyte, plasmacytoid DC, non-classical monocyte)
PLAC8	Cell type enhanced (plasmacytoid DC)
ANXA2	Low cell type specificity
CD1E	Cell type enriched (myeloid DC)
IFITM3	Cell type enriched (myeloid DC)
STK2B	Low cell type specificity
PPA1	Low cell type specificity
RNASE6	Cell type enhanced (plasmacytoid DC)
TMSB10	Low cell type specificity
TGFBI	Group enriched (plasmacytoid DC, classical monocyte, myeloid DC, intermediate monocyte, non-classical monocyte)
RPS18	Low cell type specificity
CSTA	Group enriched (classical monocyte, myeloid DC, intermediate monocyte, neutrophil, non-classical monocyte)
LGALS1	Low cell type specificity
RPS2	Low cell type specificity
PSTPIP2	Cell type enhanced (eosinophil)
GPR183	Low cell type specificity
PKIB	Cell type enriched (myeloid DC)
ANXA1	Low cell type specificity
SNX3	Low cell type specificity
RPL4	Low cell type specificity
CD74	Group enriched (naive B-cell, memory B-cell, myeloid DC, intermediate monocyte, plasmacytoid DC, classical monocyte, non-classical monocyte)
RPL18A	Low cell type specificity
CLEC4A	Group enriched (classical monocyte, myeloid DC, neutrophil, intermediate monocyte, basophil, non-classical monocyte)
TAGLN2	Low cell type specificity
RPS24	Low cell type specificity

Supplementary Table 4. List of Diseases and Functions deregulated in cluster 0 compared with cluster 1.

Diseases or Functions Annotation	p-Value	Activation z-score	Molecules
Quantity of lymphocytes	4,27E-12	-7,62	IRF1,AKAP13,ICOS,SIPA1,P2RX5,RASGRP1,ELF1,CXCR4,SETD3,LCK,PRKCB,TRAF1,LAG3,S1PR1,IL2,TXNIP,HTRA2,SMAD2,MAF,PRDM1,TAPBP,KAT6A,CD47,DNAJA3,CAMK2G,GNLY,CDKN2B,BCLAF1,FCMR,BAX,FAS,EPS15,RICTOR,PTPN22,NDFIP1,IKZF3,RASAL3,ELK4,TAP1,B4GALT1,GPR18,ITPKB,CD3D,CREBBP,GSTP1,CD27,GPR132,BCL11B,CD3G,EP300,FUBP1,ARID4A,PARP1,CD40LG,BCL2,ZBTB7A,TNFRSF18,ORMDL3,TBX21,ITGAL,PLAU,RBL2,PRKACB,RPS6KA5,STAT3,PIK3AP1,CBLB,PRKCH,CYTIP,CD3E,PRF1,SMPD1,JAK1,HCST,BAD,CDKN1B,STK17B,SEMA4D,TNFAIP8L2,AP3B1,IL13RA1,CD6,SPPL3,IKZF1,CCND3,INPP5D,ITK,TOX,UPF2,PRKDC,ANGPT2,MYBL1,CD8B,MARK2,ATM,TIGIT,SLA,PDCD1,IRF2,THEMIS,RGS1,UBASH3A,SOCS1,TNIP1,KMT2E,SH2D1A,ATXN1,CDKN2A,FCER1G,IL2RG,STARD7,STING1,IL7R,LAT,POLM,TLE4,LTB,TLN1,SIT1,BATF,RAP1B,BCL2L1,ETS1,STAT5B,STK4,FLT3LG,CBFB,RHOH,SLC6A6,IFNG,CREB1,ZBTB20,DNMT1,WIPF1,FYN,ISG15,STAT5A,TRIB2,MS4A1,CCR2,TRAF3IP3,STAT4,NOTCH1,MBP,RAP1GDS1,CD5,NABP1,MAP3K14,CAMK4,LY9,PIKFYVE,FYB1,MATK,KLRC1,PYCARD,GIMAP1,HLA-A,CD7,ARHGEF1,XCL1,DUSP10,ATG5,TOB1,PRKCQ,GATA3,MALT1,DOCK8,SYK,ARHGEF6,CD8A,ASXL1,ZAP70,CYLD,GRAP2,PAG1,CCL5,ZEB1,IKBKB,PIM1,CD69,PPARD,DOCK11,ITGA4,CASP3,MAPK14,AKT3,ITGAE,IL2RB,PIK3R1,RAC2,DEF6,SLAMF6,NMT2,N4BP1,PTGDR,KRAS,RC3H2,MSN,PPP3CB,SERPINB6,TACC3,NABP2,TNFAIP3,ARID4B,RUNX3,PTPR
Quantity of mononuclear leukocytes	3,54E-11	-7,40	IRF1,PML,AKAP13,ICOS,SIPA1,P2RX5,RASGRP1,ELF1,CXCR4,SETD3,LCK,PRKCB,TRAF1,LAG3,S1PR1,IL2,TXNIP,HTRA2,SMAD2,MAF,PRDM1,TAPBP,KAT6A,CD47,DNAJA3,CAMK2G,GNLY,CDKN2B,BCLAF1,FCMR,BAX,FAS,EPS15,RICTOR,PTPN22,NDFIP1,IKZF3,RASAL3,ELK4,TAP1,B4GALT1,GPR18,ITPKB,CD3D,CREBBP,GSTP1,CD27,GPR132,BCL11B,CD3G,EP300,FUBP1,ARID4A,PARP1,CD40LG,BCL2,ZBTB7A,TNFRSF18,ORMDL3,TBX21,ITGAL,PLAU,RBL2,PRKACB,RPS6KA5,STAT3,PIK3AP1,CBLB,PRKCH,CYTIP,CD3E,PRF1,SMPD1,JAK1,HCST,BAD,CDKN1B,STK17B,SEMA4D,TNFAIP8L2,AP3B1,IL13RA1,CD6,SPPL3,IKZF1,CCND3,INPP5D,ITK,TOX,UPF2,PRKDC,ANGPT2,MYBL1,CD8B,MARK2,ATM,TIGIT,SLA,PDCD1,IRF2,THEMIS,RGS1,UBASH3A,SOCS1,TNIP1,KMT2E,SH2D1A,ATXN1,CDKN2A,FCER1G,IL2RG,STARD7,STING1,IL7R,LAT,POLM,TLE4,LTB,TLN1,SIT1,BATF,RAP1B,BCL2L1,ETS1,STAT5B,STK4,FLT3LG,CBFB,RHOH,SLC6A6,IFNG,CREB1,ZBTB20,DNMT1,DOK2,WIPF1,FYN,ISG15,STAT5A,TRIB2,MS4A1,CCR2,TRAF3IP3,STAT4,NOTCH1,MBP,RAP1GDS1,CD5,NABP1,MAP3K14,CAMK4,LY9,PIKFYVE,FYB1,MATK,KLRC1,PYCARD,GIMAP1,HLA-A,CD7,ARHGEF1,XCL1,DUSP10,ATG5,TOB1,PRKCQ,GATA3,MALT1,DOCK8,SYK,ARHGEF6,CD8A,ASXL1,ZAP70,CYLD,GRAP2,PAG1,CCL5,ZEB1,IKBKB,PIM1,CD69,PPARD,SLFN12L,DOCK11,ITGA4,CASP3,MAPK14,AKT3,ITGAE,IL2RB,PIK3R1,RAC2,DEF6,SLAMF6,NMT2,N4BP1,PTGDR,KRAS,RC3H2,MSN,PPP3CB,SERPINB6,TACC3,NABP2,TNFAIP3,ARID4B,RU
Quantity of cells	2,05E-07	-6,60	IRF1,PML,AKAP13,ICOS,SIPA1,P2RX5,RASGRP1,ELF1,CXCR6,CXCR4,HMGB1,SETD3,LCK,PRKCB,TRAF1,SRP54,LAG3,S1PR1,IL2,TXNIP,HTRA2,SMAD2,MAF,PRDM1,TAPBP,GBA,KAT6A,CD47,DNAJA3,CAMK2G,GNLY,CDKN2B,BCLAF1,FCMR,BAX,FAS,EPS15,RICTOR,PTPN22,NDFIP1,IKZF3,RASAL3,TAP1,ELK4,B4GALT1,GPR18,ITPKB,CD3D,CREBBP,GSTP1,CD27,GPR132,BCL11B,CD3G,EP300,FUBP1,ARID4A,PARP1,CD40LG,BCL2,ZBTB7A,TNFRSF18,ORMDL3,TBX21,ITGAL,PLAU,RBL2,PRKACB,RPS6KA5,STAT3,PIK3AP1,CBLB,CYTIP,PRKCH,CD3E,PRF1,SMPD1,JAK1,HCST,BAD,CDKN1B,STK17B,SEMA4D,S1PR4,TNFAIP8L2,KDM5A,AP3B1,IL13RA1,CD6,SPPL3,ZFP36L2,IKZF1,CCND3,INPP5D,ITK,TOX,UPF2,PRKDC,ANGPT2,MYBL1,CD8B,MARK2,ATM,TIGIT,SLA,PDCD1,IRF2,THEMIS,RGS1,UBASH3A,SOCS1,TNIP1,KMT2E,SH2D1A,ATXN1,CDKN2A,FCER1G,IL2RG,STARD7,STING1,IL7R,LAT,POLM,TLE4,MDM4,LTB,TLN1,PTGER2,SIT1,BATF,RAP1B,BCL2L1,ETS1,STAT5B,STK4,JUNB,FLT3LG,CBFB,RHOH,SLC6A6,IFNG,CREB1,ZBTB20,DNMT1,DOK2,WIPF1,PDE4B,FYN,STAT5A,ISG15,TRIB2,PPP2CA,MS4A1,CCR2,TRAF3IP3,STAT4,NOTCH1,MBP,RAP1GDS1,CD5,NABP1,MAP3K14,CAMK4,LY9,PIKFYVE,FYB1,KLRC1,MATK,PYCARD,GIMAP1,SOAT1,HLA-A,CD7,ARHGEF1,XCL1,LYST,DUSP10,ATG5,TOB1,PRKCQ,GATA3,MALT1,DOCK8,SYK,ARHGEF6,CD8A,ASXL1,ZAP70,CYLD,GRAP2,PAG1,CCL5,ZEB1,IKBKB,PIM1,CD69,RORA,ANXA1,PPARD,SLFN12L,ADGRE5,DOCK11,ITGA4,CASP3,GFUS,MAPK14,AKT3,ITGAE,IL2RB,PIK3R1,RAC2,DEF6,SLAMF6,NMT2,N4BP1,PTGDR,SIGIRR,KRAS,PREX1,RC3H2,MSN,PPP3CB,SERPINB6,TACC3,NABP2,TNFAIP3,ARID4B,BUB1,RUNX3,PTPRC,EPAS1,CD247,CASP8,XKR8
Quantity of leukocytes	2,45E-07	-6,54	IRF1,PML,AKAP13,ICOS,SIPA1,P2RX5,RASGRP1,ELF1,CXCR6,CXCR4,HMGB1,SETD3,LCK,PRKCB,TRAF1,SRP54,LAG3,S1PR1,IL2,TXNIP,HTRA2,SMAD2,MAF,PRDM1,TAPBP,GBA,KAT6A,CD47,DNAJA3,CAMK2G,GNLY,CDKN2B,BCLAF1,FCMR,BAX,FAS,EPS15,RICTOR,PTPN22,NDFIP1,IKZF3,RASAL3,ELK4,TAP1,B4GALT1,GPR18,ITPKB,CD3D,CREBBP,GSTP1,CD27,GPR132,BCL11B,CD3G,EP300,FUBP1,ARID4A,PARP1,CD40LG,BCL2,TNFRSF18,ZBTB7A,ORMDL3,TBX21,ITGAL,PLAU,RBL2,PRKACB,RPS6KA5,STAT3,PIK3AP1,CBLB,PRKCH,CYTIP,CD3E,PRF1,SMPD1,JAK1,HCST,BAD,CDKN1B,STK17B,SEMA4D,S1PR4,TNFAIP8L2,KDM5A,AP3B1,IL13RA1,CD6,SPPL3,ZFP36L2,IKZF1,CCND3,INPP5D,ITK,TOX,UPF2,PRKDC,ANGPT2,MYBL1,CD8B,MARK2,ATM,TIGIT,SLA,PDCD1,IRF2,THEMIS,RGS1,UBASH3A,SOCS1,TNIP1,KMT2E,SH2D1A,ATXN1,CDKN2A,FCER1G,IL2RG,STARD7,STING1,IL7R,LAT,POLM,TLE4,MDM4,LTB,TLN1,PTGER2,SIT1,BATF,RAP1B,BCL2L1,ETS1,STAT5B,STK4,FLT3LG,CBFB,RHOH,SLC6A6,IFNG,CREB1,ZBTB20,DNMT1,DOK2,WIPF1,PDE4B,FYN,ISG15,STAT5A,TRIB2,PPP2CA,MS4A1,CCR2,TRAF3IP3,STAT4,NOTCH1,MBP,RAP1GDS1,CD5,NABP1,MAP3K14,CAMK4,LY9,PIKFYVE,FYB1,MATK,KLRC1,PYCARD,GIMAP1,SOAT1,HLA-A,CD7,ARHGEF1,XCL1,LYST,DUSP10,ATG5,TOB1,PRKCQ,GATA3,MALT1,DOCK8,SYK,ARHGEF6,CD8A,ASXL1,ZAP70,CYLD,GRAP2,PAG1,CCL5,ZEB1,IKBKB,PIM1,CD69,RORA,ANXA1,PPARD,SLFN12L,ADGRE5,DOCK11,ITGA4,CASP3,GFUS,MAPK14,AKT3,ITGAE,IL2RB,PIK3R1,RAC2,DEF6,SLAMF6,NMT2,N4BP1,PTGDR,SIGIRR,KRAS,PREX1,RC3H2,MSN,PPP3CB,SERPINB6,TACC3,NABP2,TNFAIP3,ARID4B,BUB1,RUNX3,PTPRC,EPAS1,CD247,CASP8,XKR8
Leukopoiesis	8,00E-11	-6,53	IRF1,AKAP13,ICOS,RASGRP4,HNRNPL,RASGRP1,ELF1,CXCR4,HMGB1,LCK,SRP54,S1PR1,IL2,TCF7,SMAD2,CD46,PTGER4,MAF,PRDM1,TAPBP,GBA,PBXIP1,PCID2,KLRD1,CD47,USP15,DNAJA3,TRAT1,GNLY,CDKN2B,JADE1,BAX,IFI16,FAS,SLAMF1,FOXP1,RICTOR,PTPN22,NDFIP1,IKZF3,ELK4,GPR174,ITPKB,CD3D,CREBBP,DGKZ,PRDX2,NCOR1,SLA2,CD27,BCL11B,CD3G,EP300,PPP3CA,ARID4A,PARP1,CD40LG,BCL2,ZBTB7A,TNFRSF18,CD2,PGAP3,TBX21,ITGAL,RBL2,CARD9,ELK1,STAT3,PIK3AP1,FUT8,CBLB,PRKCH,CD3E,SMPD1,JAK1,SH2D2A,BAD,CDKN1B,ZRSR2,AP3B1,IL13RA1,PLA2G6,CD6,ZFP36L2,IKZF1,CCND3,INPP5D,PPP2R3C,ITK,TOX,KAT7,HSP90AA1,PRKDC,ITM2A,CD8B,ATM,FAM102A,SLA,PDCD1,IRF2,LBR,THEMIS,NCSTN,ZNF831,SOCS1,KMT2E,SH2D1A,CDKN2A,FCER1G,IL2RG,F2R,IL7R,TFAP4,LAT,POLM,WWP1,LTB,TLN1,EZR,SOCS2,SIT1,BATF,AZI2,BCL2L1,ETS1,STAT5B,FLT3LG,CBFB,RHOH,IFNG,CREB1,DOK2,ZBTB16,WIPF1,LCP1,FYN,STAT5A,MS4A1,CCR2,TRAF3IP3,STAT4,NOTCH1,STIM2,CD5,BRD3,MAP3K14,CAMK4,LY9,MATK,JMJD6,GIMAP1,HLA-A,SP3,DUSP10,ATG5,PRKCQ,PLEKHA2,PRKAA1,PHC1,GATA3,MALT1,DOCK8,SYK,CD8A,ZAP70,PIK3C3,IL18R1,NR4A2,CYLD,GRAP2,PAG1,N4BP2L2,CCL5,DNAJB1,ZEB1,IKBKB,PIM1,CD69,RORA,ANXA1,BRAF,DOCK11,ITGA4,NCAPH2,NKAP,GSK3B,MAPK14,HECTD1,IL2RB,TAB2,PIK3R1,RAC2,DEF6,SLAMF6,NMT2,GADD45G,KRAS,PREX1,PPP3CB,SENP1,T
Cellular homeostasis	1,13E-13	-6,52	IRF1,ICOS,RASGRP4,CDKN2A,FCER1G,HNRNPL,RASGRP1,IL2RG,ELF1,CXCR4,HMGB1,IL7R,LCK,TFAP4,LAT,LAG3,WWP1,SKIL,LTB,S1PR1,IL2,DLG1,TCF7,SMAD2,CD46,EZR,LAX1,SOCS2,PTGER4,MAF,PRDM1,TAPBP,GBA,ZC3H8,KLRD1,CD47,USP15,SIT1,DNAJA3,TRAT1,BATF,BCL2L1,BAX,ETS1,STAT5B,FAS,JUNB,FLT3LG,CBFB,RHOH,SLAMF1,IFNG,CREB1,DOK2,ZBTB16,RICTOR,PTPN22,NDFIP1,PDE4B,ELK4,GPR174,FYN,ITPKB,STAT5A,CD3D,PLCB3,CREBBP,DGKZ,PRDX2,NCOR1,CD27,CCR2,TRAF3IP3,STAT4,NOTCH1,GPR132,STIM2,CD5,BCL11B,CD3G,EP300,MAP3K14,WDR37,LY9,PPP3CA,JMJD6,GIMAP1,PARP1,SOAT1,HLA-A,CD40LG,BCL2,SP3,TNFRSF18,CD2,PGAP3,TBX21,DUSP10,ITGAL,ATG5,RBL2,PRKCQ,PRKAA1,CARD9,ELK1,GATA3,MALT1,SYK,STAT3,CD8A,CBLB,PRKCH,BACE1,ZAP70,PIK3C3,IL18R1,NR4A2,CYLD,GRAP2,CD3E,PAG1,CCL5,DNAJB1,ZEB1,IKBKB,JAK1,PIM1,CD69,RORA,SH2D2A,BAD,ANXA1,BRAF,CDKN1B,DOCK11,ITGA4,CASP3,NCAPH2,NKAP,MAPK14,HECTD1,AP3B1,IL2RB,PIK3R1,CD6,RAC2,ZFP36L2,IKZF1,INPP5D,DEF6,PPP2R3C,ITK,TOX,SLAMF6,KAT7,NMT2,HSP90AA1,PRKDC,GADD45G,HEBP1,KRAS,CD8B,PREX1,RC3H2,ATM,PPP3CB,SLA,PDCD1,IRF2,THEMIS,TNFAIP3,RUNX3,SOCS1,PTPRC,CD247,SH2D1A
Lymphopoiesis	2,14E-13	-6,50	IRF1,ICOS,RASGRP4,CDKN2A,FCER1G,HNRNPL,RASGRP1,IL2RG,ELF1,CXCR4,HMGB1,IL7R,LCK,TFAP4,LAT,POLM,WWP1,LTB,S1PR1,IL2,TLN1,TCF7,SMAD2,CD46,EZR,SOCS2,PTGER4,MAF,PRDM1,TAPBP,GBA,PCID2,KLRD1,USP15,SIT1,DNAJA3,TRAT1,BATF,BCL2L1,BAX,ETS1,STAT5B,FAS,FLT3LG,CBFB,RHOH,SLAMF1,IFNG,CREB1,DOK2,FOXP1,ZBTB16,RICTOR,PTPN22,NDFIP1,WIPF1,IKZF3,ELK4,LCP1,GPR174,FYN,ITPKB,STAT5A,CD3D,CREBBP,DGKZ,PRDX2,NCOR1,CD27,MS4A1,CCR2,TRAF3IP3,STAT4,NOTCH1,STIM2,CD5,BCL11B,CD3G,EP300,MAP3K14,LY9,PPP3CA,JMJD6,GIMAP1,PARP1,HLA-A,CD40LG,BCL2,SP3,TNFRSF18,ZBTB7A,CD2,PGAP3,TBX21,DUSP10,ITGAL,ATG5,RBL2,PRKCQ,PRKAA1,PLEKHA2,CARD9,ELK1,PHC1,GATA3,MALT1,DOCK8,SYK,STAT3,CD8A,PIK3AP1,FUT8,CBLB,PRKCH,ZAP70,PIK3C3,IL18R1,NR4A2,CYLD,GRAP2,CD3E,PAG1,CCL5,DNAJB1,ZEB1,JAK1,IKBKB,PIM1,CD69,RORA,SH2D2A,BAD,ANXA1,BRAF,CDKN1B,DOCK11,ITGA4,NCAPH2,NKAP,MAPK14,HECTD1,AP3B1,IL2RB,TAB2,PIK3R1,CD6,ZFP36L2,IKZF1,CCND3,INPP5D,DEF6,PPP2R3C,ITK,TOX,SLAMF6,KAT7,NMT2,HSP90AA1,PRKDC,GADD45G,ITM2A,KRAS,CD8B,PREX1,ATM,PPP3CB,SLA,PDCD1,IRF2,THEMIS,NCSTN,TNFAIP3,RUNX3,SOCS1,PTPRC,CD247,SH2D1A
Differentiation of mononuclear leukocytes	1,15E-11	-6,47	IRF1,ICOS,RASGRP4,CDKN2A,FCER1G,HNRNPL,RASGRP1,IL2RG,ELF1,CXCR4,HMGB1,IL7R,LCK,TFAP4,LAT,POLM,WWP1,LTB,S1PR1,IL2,TLN1,TCF7,SMAD2,CD46,EZR,SOCS2,PTGER4,MAF,PRDM1,TAPBP,GBA,PCID2,KLRD1,USP15,SIT1,DNAJA3,TRAT1,BATF,GNLY,BCL2L1,BAX,IFI16,ETS1,STAT5B,FAS,FLT3LG,CBFB,RHOH,SLAMF1,IFNG,CREB1,DOK2,FOXP1,ZBTB16,RICTOR,PTPN22,NDFIP1,WIPF1,IKZF3,ELK4,LCP1,GPR174,FYN,ITPKB,STAT5A,CD3D,CREBBP,DGKZ,PRDX2,NCOR1,CD27,MS4A1,CCR2,TRAF3IP3,STAT4,NOTCH1,STIM2,CD5,BCL11B,CD3G,EP300,MAP3K14,LY9,PPP3CA,JMJD6,GIMAP1,ARID4A,PARP1,HLA-A,CD40LG,BCL2,SP3,TNFRSF18,ZBTB7A,CD2,TBX21,PGAP3,DUSP10,ITGAL,ATG5,RBL2,PRKCQ,PRKAA1,PLEKHA2,CARD9,ELK1,PHC1,GATA3,MALT1,DOCK8,SYK,STAT3,CD8A,PIK3AP1,FUT8,CBLB,PRKCH,ZAP70,PIK3C3,IL18R1,NR4A2,CYLD,GRAP2,CD3E,PAG1,CCL5,DNAJB1,ZEB1,IKBKB,JAK1,PIM1,RORA,CD69,SH2D2A,BAD,ANXA1,BRAF,CDKN1B,DOCK11,ITGA4,NCAPH2,NKAP,MAPK14,HECTD1,AP3B1,IL2RB,TAB2,PIK3R1,CD6,ZFP36L2,IKZF1,CCND3,INPP5D,DEF6,PPP2R3C,ITK,TOX,SLAMF6,KAT7,NMT2,HSP90AA1,PRKDC,GADD45G,ITM2A,KRAS,CD8B,PREX1,ATM,PPP3CB,SENP1,SLA,PDCD1,IRF2,THEMIS,NCSTN,TNFAIP3,RUNX3,SOCS1,PTPRC,CD247,SH2D1A
Hematopoiesis of mononuclear leukocytes	1,46E-11	-6,47	IRF1,ICOS,RASGRP4,CDKN2A,FCER1G,HNRNPL,RASGRP1,IL2RG,ELF1,CXCR4,HMGB1,IL7R,LCK,TFAP4,LAT,POLM,WWP1,LTB,S1PR1,IL2,TLN1,TCF7,SMAD2,CD46,EZR,SOCS2,PTGER4,MAF,PRDM1,TAPBP,GBA,PCID2,KLRD1,USP15,SIT1,DNAJA3,TRAT1,BATF,GNLY,BCL2L1,BAX,IFI16,ETS1,STAT5B,FAS,FLT3LG,CBFB,RHOH,SLAMF1,IFNG,CREB1,DOK2,FOXP1,ZBTB16,RICTOR,PTPN22,NDFIP1,WIPF1,IKZF3,ELK4,LCP1,GPR174,FYN,ITPKB,STAT5A,CD3D,CREBBP,DGKZ,PRDX2,NCOR1,CD27,MS4A1,CCR2,TRAF3IP3,STAT4,NOTCH1,STIM2,CD5,BCL11B,CD3G,EP300,MAP3K14,LY9,PPP3CA,JMJD6,GIMAP1,PARP1,HLA-A,CD40LG,BCL2,SP3,TNFRSF18,ZBTB7A,CD2,TBX21,PGAP3,DUSP10,ITGAL,ATG5,RBL2,PRKCQ,PRKAA1,PLEKHA2,CARD9,ELK1,PHC1,GATA3,MALT1,DOCK8,SYK,STAT3,CD8A,PIK3AP1,FUT8,CBLB,PRKCH,ZAP70,PIK3C3,IL18R1,NR4A2,CYLD,GRAP2,CD3E,PAG1,CCL5,DNAJB1,ZEB1,IKBKB,JAK1,PIM1,CD69,RORA,SH2D2A,BAD,ANXA1,BRAF,CDKN1B,DOCK11,ITGA4,NCAPH2,NKAP,MAPK14,HECTD1,AP3B1,IL2RB,TAB2,PIK3R1,CD6,ZFP36L2,IKZF1,CCND3,INPP5D,DEF6,PPP2R3C,ITK,TOX,SLAMF6,KAT7,NMT2,HSP90AA1,PRKDC,GADD45G,ITM2A,KRAS,CD8B,PREX1,ATM,PPP3CB,SENP1,SLA,PDCD1,IRF2,THEMIS,NCSTN,TNFAIP3,RUNX3,SOCS1,PTPRC,CD247,SH2D1A
Quantity of T lymphocytes	2,91E-15	-6,20	IRF1,ICOS,SIPA1,P2RX5,CDKN2A,FCER1G,RASGRP1,IL2RG,STARD7,ELF1,STING1,CXCR4,IL7R,LCK,PRKCB,LAT,LAG3,TLE4,LTB,S1PR1,IL2,TXNIP,TLN1,MAF,PRDM1,TAPBP,KAT6A,CD47,SIT1,DNAJA3,CAMK2G,BATF,GNLY,BCLAF1,BCL2L1,FCMR,BAX,ETS1,STAT5B,FAS,STK4,FLT3LG,CBFB,RHOH,SLC6A6,IFNG,CREB1,ZBTB20,DNMT1,RICTOR,PTPN22,NDFIP1,WIPF1,RASAL3,ELK4,TAP1,GPR18,FYN,ITPKB,STAT5A,CD3D,CREBBP,TRIB2,CD27,CCR2,TRAF3IP3,NOTCH1,MBP,GPR132,RAP1GDS1,CD5,BCL11B,CD3G,EP300,MAP3K14,CAMK4,LY9,PIKFYVE,FYB1,KLRC1,PYCARD,ARID4A,PARP1,HLA-A,CD40LG,CD7,BCL2,ARHGEF1,TNFRSF18,XCL1,TBX21,DUSP10,ITGAL,ATG5,TOB1,PRKCQ,PRKACB,GATA3,MALT1,DOCK8,RPS6KA5,SYK,STAT3,ARHGEF6,CD8A,PIK3AP1,CBLB,PRKCH,ASXL1,ZAP70,CYLD,GRAP2,CD3E,PAG1,CCL5,PRF1,SMPD1,ZEB1,IKBKB,JAK1,PIM1,HCST,CD69,BAD,PPARD,CDKN1B,ITGA4,CASP3,STK17B,SEMA4D,MAPK14,AKT3,TNFAIP8L2,AP3B1,ITGAE,IL13RA1,IL2RB,PIK3R1,RAC2,IKZF1,CCND3,INPP5D,DEF6,ITK,TOX,SLAMF6,NMT2,UPF2,N4BP1,PRKDC,PTGDR,KRAS,ANGPT2,CD8B,RC3H2,ATM,MSN,PPP3CB,SERPINB6,TIGIT,SLA,PDCD1,IRF2,THEMIS,TACC3,TNFAIP3,ARID4B,RUNX3,SOCS1,PTPRC,TNIP1,KMT2E,CD247,SH2D1A,CASP8,ATXN1,XKR8
Lymphocyte homeostasis	1,39E-14	-5,90	IRF1,ICOS,RASGRP4,CDKN2A,FCER1G,HNRNPL,RASGRP1,IL2RG,ELF1,CXCR4,HMGB1,IL7R,LCK,TFAP4,LAT,LAG3,WWP1,SKIL,LTB,S1PR1,IL2,TCF7,SMAD2,CD46,SOCS2,PTGER4,MAF,PRDM1,TAPBP,GBA,ZC3H8,KLRD1,USP15,SIT1,DNAJA3,TRAT1,BATF,BAX,STAT5B,FAS,ETS1,FLT3LG,CBFB,RHOH,SLAMF1,CREB1,IFNG,DOK2,ZBTB16,RICTOR,PTPN22,NDFIP1,ELK4,GPR174,FYN,ITPKB,STAT5A,CD3D,CREBBP,NCOR1,DGKZ,PRDX2,CD27,CCR2,TRAF3IP3,STAT4,NOTCH1,STIM2,BCL11B,CD5,CD3G,EP300,MAP3K14,LY9,PPP3CA,JMJD6,GIMAP1,PARP1,HLA-A,CD40LG,BCL2,SP3,TNFRSF18,PGAP3,CD2,TBX21,DUSP10,ITGAL,ATG5,RBL2,PRKAA1,PRKCQ,CARD9,ELK1,GATA3,MALT1,SYK,STAT3,CD8A,CBLB,PRKCH,ZAP70,PIK3C3,NR4A2,IL18R1,CYLD,GRAP2,CD3E,PAG1,CCL5,DNAJB1,ZEB1,IKBKB,JAK1,PIM1,RORA,CD69,SH2D2A,BAD,ANXA1,BRAF,CDKN1B,DOCK11,CASP3,ITGA4,NKAP,NCAPH2,MAPK14,HECTD1,AP3B1,IL2RB,PIK3R1,CD6,ZFP36L2,IKZF1,INPP5D,DEF6,PPP2R3C,ITK,TOX,SLAMF6,KAT7,NMT2,HSP90AA1,PRKDC,GADD45G,KRAS,CD8B,PREX1,RC3H2,ATM,PPP3CB,SLA,PDCD1,IRF2,THEMIS,TNFAIP3,RUNX3,SOCS1,PTPRC,CD247,SH2D1A
Homeostasis of leukocytes	4,19E-14	-5,90	IRF1,ICOS,RASGRP4,CDKN2A,FCER1G,HNRNPL,RASGRP1,IL2RG,ELF1,CXCR4,HMGB1,IL7R,LCK,TFAP4,LAT,LAG3,WWP1,SKIL,LTB,S1PR1,IL2,TCF7,SMAD2,CD46,SOCS2,PTGER4,MAF,PRDM1,TAPBP,GBA,KLRD1,ZC3H8,USP15,SIT1,DNAJA3,TRAT1,BATF,BAX,STAT5B,FAS,ETS1,FLT3LG,CBFB,RHOH,SLAMF1,CREB1,IFNG,DOK2,ZBTB16,RICTOR,PTPN22,NDFIP1,PDE4B,ELK4,GPR174,FYN,ITPKB,STAT5A,CD3D,CREBBP,NCOR1,DGKZ,PRDX2,CD27,CCR2,TRAF3IP3,STAT4,NOTCH1,STIM2,BCL11B,CD5,CD3G,EP300,MAP3K14,LY9,PPP3CA,JMJD6,GIMAP1,PARP1,HLA-A,CD40LG,BCL2,SP3,TNFRSF18,PGAP3,CD2,TBX21,DUSP10,ITGAL,ATG5,RBL2,PRKAA1,PRKCQ,CARD9,ELK1,GATA3,MALT1,SYK,STAT3,CD8A,CBLB,PRKCH,ZAP70,PIK3C3,NR4A2,IL18R1,CYLD,GRAP2,CD3E,PAG1,CCL5,DNAJB1,ZEB1,IKBKB,JAK1,PIM1,RORA,CD69,SH2D2A,BAD,ANXA1,BRAF,CDKN1B,DOCK11,CASP3,ITGA4,NKAP,NCAPH2,MAPK14,HECTD1,AP3B1,IL2RB,PIK3R1,CD6,ZFP36L2,IKZF1,INPP5D,DEF6,PPP2R3C,ITK,TOX,SLAMF6,KAT7,NMT2,HSP90AA1,PRKDC,GADD45G,KRAS,CD8B,PREX1,RC3H2,ATM,PPP3CB,SLA,PDCD1,IRF2,THEMIS,TNFAIP3,RUNX3,SOCS1,PTPRC,CD247,SH2D1A
Leukocyte migration	4,90E-03	-5,63	ICOS,RASGRP4,MIA3,FCER1G,HNRNPL,IL16,RASGRP1,F2R,CXCR4,HMGB1,CREB3,IL7R,LCK,PRKCB,LAT,LAG3,SIRPG,S1PR1,RAP2A,IL2,TLN1,EZR,ARHGAP1,AKIRIN1,PRDM1,CD47,NRAS,BATF,RAP1B,ETS1,EVL,FAS,STK4,FLT3LG,CBFB,SLAMF1,IFNG,DOK2,ARHGAP18,RICTOR,WIPF1,PDE4B,SLC16A1,AQP3,B4GALT1,LCP1,GPR18,FYN,STAT5A,PLCB3,CCR2,MS4A4A,NOTCH1,GPR132,CD5,CD58,BCL11B,STIM2,CD99,MAP3K14,PTPRO,PIKFYVE,FYB1,ABCC1,PYCARD,CCDC88A,PARP1,CD40LG,BCL2,ARHGEF1,MAPK13,TNFRSF18,XCL1,CD2,TBX21,LYST,ITGAL,PAFAH1B1,PLAU,PRKCQ,CARD9,MYH9,DOCK8,SYK,CD63,STAT3,ARHGEF6,CD8A,FUT8,ANO6,CBLB,CYTIP,ACTN4,ZAP70,PIK3C3,CCL5,CLTC,SMPD1,IKBKB,JAK1,CD69,ANXA1,PPARD,BRAF,SLFN12L,ADGRE5,DOCK11,ITGA4,PPIF,SKAP1,SEMA4D,GSK3B,GYPC,MAPK14,F11R,S1PR4,TNFAIP8L2,ITGAE,SELENOK,TSPAN14,PLA2G6,PIK3R1,RAC2,LY96,ITGA1,C1GALT1,INPP5D,ITK,AIF1,MGAT5,HSP90AA1,PTGDR,HEBP1,SIGIRR,KRAS,ANGPT2,PREX1,TAX1BP1,TNFSF14,NR1H2,PDCD1,CD44,MMP7,RGS1,RUNX3,EPAS1,PTPRC,SH2D1A,CASP8
T cell homeostasis	3,58E-14	-5,58	IRF1,ICOS,RASGRP4,CDKN2A,FCER1G,HNRNPL,RASGRP1,IL2RG,ELF1,CXCR4,HMGB1,IL7R,LCK,TFAP4,LAT,LAG3,WWP1,LTB,S1PR1,IL2,TCF7,SMAD2,CD46,SOCS2,PTGER4,MAF,PRDM1,TAPBP,GBA,ZC3H8,KLRD1,USP15,SIT1,DNAJA3,TRAT1,BATF,BAX,STAT5B,FAS,ETS1,CBFB,RHOH,CREB1,IFNG,DOK2,ZBTB16,RICTOR,NDFIP1,PTPN22,ELK4,GPR174,FYN,ITPKB,STAT5A,CD3D,CREBBP,NCOR1,DGKZ,PRDX2,CD27,CCR2,TRAF3IP3,STAT4,NOTCH1,STIM2,BCL11B,CD5,CD3G,EP300,MAP3K14,LY9,PPP3CA,JMJD6,GIMAP1,PARP1,HLA-A,CD40LG,BCL2,SP3,TNFRSF18,PGAP3,CD2,TBX21,DUSP10,ITGAL,ATG5,RBL2,PRKAA1,PRKCQ,CARD9,ELK1,GATA3,MALT1,SYK,STAT3,CD8A,CBLB,PRKCH,ZAP70,PIK3C3,NR4A2,IL18R1,CYLD,GRAP2,CD3E,PAG1,CCL5,DNAJB1,ZEB1,IKBKB,JAK1,PIM1,RORA,CD69,SH2D2A,BAD,ANXA1,BRAF,CDKN1B,CASP3,ITGA4,NKAP,NCAPH2,MAPK14,AP3B1,IL2RB,PIK3R1,CD6,ZFP36L2,IKZF1,DEF6,PPP2R3C,ITK,TOX,SLAMF6,NMT2,PRKDC,HSP90AA1,GADD45G,KRAS,CD8B,PREX1,RC3H2,ATM,PPP3CB,SLA,PDCD1,IRF2,THEMIS,TNFAIP3,RUNX3,SOCS1,PTPRC,CD247,SH2D1A
T cell development	2,78E-13	-5,55	IRF1,ICOS,RASGRP4,CDKN2A,FCER1G,HNRNPL,RASGRP1,IL2RG,ELF1,CXCR4,HMGB1,IL7R,LCK,TFAP4,LAT,WWP1,LTB,S1PR1,IL2,TCF7,SMAD2,CD46,SOCS2,PTGER4,MAF,PRDM1,TAPBP,GBA,KLRD1,USP15,SIT1,DNAJA3,TRAT1,BATF,BAX,STAT5B,FAS,ETS1,CBFB,RHOH,CREB1,IFNG,DOK2,ZBTB16,RICTOR,NDFIP1,PTPN22,ELK4,GPR174,FYN,ITPKB,STAT5A,CD3D,CREBBP,NCOR1,DGKZ,PRDX2,CD27,CCR2,TRAF3IP3,STAT4,NOTCH1,STIM2,BCL11B,CD5,CD3G,EP300,MAP3K14,LY9,PPP3CA,JMJD6,GIMAP1,PARP1,HLA-A,CD40LG,BCL2,SP3,TNFRSF18,CD2,PGAP3,TBX21,DUSP10,ITGAL,ATG5,RBL2,PRKAA1,PRKCQ,CARD9,ELK1,GATA3,MALT1,SYK,STAT3,CD8A,CBLB,PRKCH,ZAP70,PIK3C3,NR4A2,IL18R1,CYLD,GRAP2,CD3E,PAG1,CCL5,DNAJB1,ZEB1,IKBKB,JAK1,PIM1,CD69,RORA,SH2D2A,BAD,ANXA1,BRAF,CDKN1B,ITGA4,NKAP,NCAPH2,MAPK14,AP3B1,IL2RB,PIK3R1,CD6,ZFP36L2,IKZF1,DEF6,ITK,TOX,SLAMF6,NMT2,PRKDC,HSP90AA1,GADD45G,KRAS,CD8B,PREX1,ATM,PPP3CB,SLA,PDCD1,IRF2,THEMIS,TNFAIP3,RUNX3,SOCS1,PTPRC,CD247,SH2D1A
Cell movement	3,13E-03	-5,53	ICOS,RASGRP4,MIA3,FCER1G,HNRNPL,IL16,RASGRP1,F2R,CXCR4,HMGB1,CREB3,IL7R,LCK,PRKCB,LAT,LAG3,SIRPG,LTB,S1PR1,RAP2A,IL2,TLN1,EZR,ARHGAP1,AKIRIN1,PRDM1,CD47,NRAS,BATF,RAP1B,BAX,ETS1,EVL,FAS,STK4,FLT3LG,CBFB,SLAMF1,IFNG,DOK2,ARHGAP18,RICTOR,WIPF1,PDE4B,SLC16A1,AQP3,B4GALT1,LCP1,GPR18,FYN,STAT5A,PLCB3,CCR2,MS4A4A,NOTCH1,GPR132,STIM2,CD5,CD58,BCL11B,CD99,MAP3K14,PTPRO,PIKFYVE,FYB1,ABCC1,PYCARD,CCDC88A,PARP1,CD40LG,BCL2,ARHGEF1,MAPK13,TNFRSF18,XCL1,CD2,TBX21,LYST,ITGAL,PAFAH1B1,PLAU,PRKCQ,CARD9,MYH9,DOCK8,SYK,CD63,STAT3,ARHGEF6,CD8A,FUT8,ANO6,CBLB,CYTIP,ACTN4,ZAP70,PIK3C3,CCL5,CLTC,SMPD1,IKBKB,JAK1,CD69,ANXA1,PPARD,BRAF,SLFN12L,ADGRE5,DOCK11,ITGA4,PPIF,SKAP1,SEMA4D,GSK3B,GYPC,MAPK14,F11R,S1PR4,TNFAIP8L2,ITGAE,SELENOK,TSPAN14,PLA2G6,PIK3R1,RAC2,LY96,ITGA1,C1GALT1,INPP5D,ITK,AIF1,MGAT5,HSP90AA1,PTGDR,HEBP1,SIGIRR,KRAS,ANGPT2,PREX1,TAX1BP1,TNFSF14,NR1H2,PDCD1,CD44,MMP7,RGS1,RUNX3,PTPRC,EPAS1,SH2D1A,CASP8
Cell movement of leukocytes	8,97E-04	-5,51	ICOS,RASGRP4,FCER1G,HNRNPL,IL16,RASGRP1,F2R,CXCR4,HMGB1,CREB3,IL7R,PRKCB,LAT,LAG3,S1PR1,RAP2A,IL2,TLN1,EZR,ARHGAP1,AKIRIN1,PRDM1,CD47,NRAS,BATF,RAP1B,ETS1,EVL,FAS,STK4,FLT3LG,CBFB,SLAMF1,IFNG,ARHGAP18,RICTOR,WIPF1,PDE4B,AQP3,B4GALT1,LCP1,GPR18,FYN,PLCB3,CCR2,MS4A4A,GPR132,STIM2,CD5,CD58,BCL11B,CD99,MAP3K14,PTPRO,PIKFYVE,FYB1,ABCC1,PYCARD,CCDC88A,PARP1,CD40LG,BCL2,ARHGEF1,MAPK13,XCL1,CD2,TBX21,LYST,ITGAL,PAFAH1B1,PLAU,PRKCQ,MYH9,DOCK8,SYK,CD63,STAT3,CD8A,ANO6,CBLB,CYTIP,ACTN4,ZAP70,CCL5,CLTC,SMPD1,IKBKB,JAK1,CD69,ANXA1,PPARD,BRAF,DOCK11,ITGA4,SKAP1,SEMA4D,GSK3B,MAPK14,F11R,S1PR4,TNFAIP8L2,ITGAE,SELENOK,TSPAN14,PLA2G6,PIK3R1,RAC2,ITGA1,C1GALT1,INPP5D,ITK,AIF1,MGAT5,HSP90AA1,PTGDR,HEBP1,SIGIRR,ANGPT2,PREX1,TNFSF14,NR1H2,PDCD1,CD44,MMP7,RGS1,RUNX3,SH2D1A
Cell movement of mononuclear leukocytes	1,67E-05	-5,24	ITGAL,PLAU,ICOS,PRKCQ,HNRNPL,IL16,RASGRP1,MYH9,DOCK8,F2R,CXCR4,STAT3,CD8A,HMGB1,CREB3,IL7R,ANO6,PRKCB,CBLB,LAT,CYTIP,ACTN4,LAG3,ZAP70,S1PR1,RAP2A,IL2,CCL5,CLTC,SMPD1,EZR,JAK1,CD69,ANXA1,CD47,NRAS,BATF,RAP1B,ITGA4,EVL,FAS,ETS1,STK4,SKAP1,SEMA4D,CBFB,MAPK14,F11R,IFNG,ITGAE,RICTOR,WIPF1,PLA2G6,PIK3R1,RAC2,AQP3,LCP1,FYN,INPP5D,AIF1,ITK,PLCB3,HSP90AA1,PTGDR,HEBP1,CCR2,GPR132,STIM2,BCL11B,CD58,TNFSF14,CD99,MAP3K14,PTPRO,FYB1,NR1H2,ABCC1,PDCD1,CD44,PYCARD,CD40LG,RGS1,BCL2,ARHGEF1,XCL1,SH2D1A,CD2
Cell viability of leukocytes	8,82E-09	-5,24	ICOS,PRKCQ,PRKAA1,FCER1G,IL2RG,NCF2,MALT1,ELF1,DOCK8,SYK,F2R,STAT3,CXCR4,HMGB1,IL7R,PRKCB,LAT,PIK3C3,CYLD,CD3E,SH3KBP1,IL2,TCF7,LAX1,IKBKB,PIM1,PRDM1,CAMK2G,PSMD1,BCL2L1,BAX,FCMR,STAT5B,FAS,YWHAZ,GSK3B,SEMA4D,IFNG,RICTOR,IL2RB,WIPF1,PIK3R1,RAC2,INPP5D,TSC22D3,DEF6,RAD9A,ITPKB,STAT5A,MGAT5,KRAS,STAT4,NOTCH1,BCL11B,ATM,NABP1,MAP3K14,CAMK4,LY9,PDCD1,CD44,GIMAP1,PYCARD,CD40LG,TNFAIP3,NABP2,BCL2,TNFRSF18,CASP8
Quantity of B lymphocytes	2,59E-06	-5,11	AKAP13,ICOS,FCER1G,RASGRP1,IL2RG,CXCR4,IL7R,PRKCB,POLM,TLE4,LTB,S1PR1,IL2,SMAD2,PRDM1,KAT6A,CDKN2B,RAP1B,BCLAF1,BAX,FCMR,STAT5B,FAS,ETS1,STK4,EPS15,FLT3LG,IFNG,PTPN22,WIPF1,IKZF3,FYN,STAT5A,CREBBP,TRIB2,GSTP1,CD27,MS4A1,CCR2,NABP1,EP300,MAP3K14,FUBP1,LY9,MATK,GIMAP1,ARID4A,PARP1,CD40LG,BCL2,ARHGEF1,ORMDL3,ZBTB7A,ITGAL,MALT1,DOCK8,SYK,STAT3,ARHGEF6,PIK3AP1,PRKCH,ASXL1,CYLD,PRF1,IKBKB,JAK1,PIM1,CD69,PPARD,DOCK11,SEMA4D,TNFAIP8L2,IL2RB,PIK3R1,CD6,RAC2,IKZF1,CCND3,INPP5D,ITK,N4BP1,KRAS,ANGPT2,MYBL1,MARK2,ATM,MSN,SLA,PDCD1,IRF2,NABP2,RGS1,ARID4B,UBASH3A,SOCS1,PTPRC,KMT2E,SH2D1A,CASP8
Cytotoxicity of natural killer cells	1,29E-07	-4,94	FLT3LG,ITGAL,IFNG,ICOS,KLRB1,PRKCQ,WIPF1,CD6,RASGRP1,MYH9,DOCK8,SYK,LCK,SLAMF6,LAT,CD27,PTGDR,IL2,STAT4,CD58,PRF1,CD44,CD69,CD96,NCR3,KLRD1,SOCS1,STAT5B,CD247,SH2D1A,SIGLEC7,CD2,TBX21
Migration of mononuclear leukocytes	1,18E-05	-4,88	ITGAL,PLAU,ICOS,PRKCQ,HNRNPL,IL16,RASGRP1,MYH9,DOCK8,STAT3,CXCR4,CD8A,HMGB1,IL7R,CBLB,LAT,CYTIP,LAG3,ZAP70,S1PR1,RAP2A,IL2,CCL5,CLTC,EZR,SMPD1,JAK1,CD69,ANXA1,CD47,NRAS,BATF,RAP1B,ITGA4,ETS1,EVL,FAS,STK4,SKAP1,SEMA4D,MAPK14,F11R,IFNG,ITGAE,RICTOR,PLA2G6,PIK3R1,RAC2,AQP3,LCP1,FYN,INPP5D,ITK,PLCB3,HSP90AA1,CCR2,GPR132,STIM2,CD58,BCL11B,TNFSF14,CD99,MAP3K14,FYB1,NR1H2,ABCC1,PDCD1,CD44,PYCARD,CD40LG,RGS1,ARHGEF1,BCL2,XCL1,SH2D1A,CD2
Cell viability of lymphocytes	5,79E-11	-4,84	ICOS,PRKCQ,IL2RG,NCF2,MALT1,ELF1,DOCK8,SYK,F2R,STAT3,HMGB1,IL7R,PRKCB,LAT,PIK3C3,CYLD,CD3E,IL2,SH3KBP1,TCF7,LAX1,IKBKB,PIM1,PRDM1,CAMK2G,BCL2L1,FCMR,STAT5B,FAS,GSK3B,SEMA4D,IFNG,RICTOR,IL2RB,WIPF1,INPP5D,TSC22D3,DEF6,RAD9A,ITPKB,STAT5A,KRAS,STAT4,NOTCH1,BCL11B,ATM,NABP1,MAP3K14,CAMK4,LY9,PDCD1,CD44,GIMAP1,PYCARD,CD40LG,TNFAIP3,NABP2,BCL2,TNFRSF18,CASP8
Cytotoxicity of lymphocytes	1,25E-08	-4,79	FLT3LG,ITGAL,ATG5,IFNG,ICOS,KLRB1,PRKCQ,WIPF1,CD6,HLA-F,RASGRP1,MYH9,DOCK8,SYK,LCK,FUT8,SLAMF6,CBLB,LAT,CD27,PTGDR,IL2,STAT4,CCL5,NOTCH1,CD58,PRF1,KLRC1,CD44,CD69,CD96,NCR3,KLRD1,CD40LG,HLA-A,GZMA,SOCS1,PTPRC,STAT5B,FAS,CD247,SIGLEC7,SH2D1A,XCL1,CD2,TBX21

Lymphocyte migration	3,48E-06	-4,79	ITGAL,PLAU,ICOS,PRKCQ,HNRNPL,IL16,RASGRP1,MYH9,DOCK8,STAT3,CXCR4,CD8A,HMGB1,IL7R,CBLB,LAT,CYTIP,LAG3,ZAP70,S1PR1,RAP2A,IL2,CCL5,CLTC,EZR,SMPD1,JAK1,CD69,ANXA1,CD47,NRAS,BATF,RAP1B,ITGA4,EVL,ETS1,FAS,STK4,SKAP1,SEMA4D,MAPK14,F11R,IFNG,ITGAE,RICTOR,PIK3R1,RAC2,AQP3,LCP1,FYN,INPP5D,ITK,PLCB3,HSP90AA1,CCR2,GPR132,STIM2,CD58,BCL11B,TNFSF14,MAP3K14,FYB1,NR1H2,ABCC1,PDCD1,CD44,PYCARD,RGS1,ARHGEF1,BCL2,XCL1,SH2D1A,CD2
Cell movement of lymphocytes	5,20E-06	-4,65	ITGAL,PLAU,ICOS,PRKCQ,HNRNPL,IL16,RASGRP1,MYH9,DOCK8,STAT3,CXCR4,CD8A,HMGB1,IL7R,CBLB,LAT,CYTIP,ACTN4,LAG3,ZAP70,S1PR1,RAP2A,IL2,CCL5,CLTC,EZR,SMPD1,JAK1,CD69,ANXA1,CD47,NRAS,BATF,RAP1B,ITGA4,EVL,ETS1,FAS,STK4,SKAP1,SEMA4D,CBFB,MAPK14,F11R,IFNG,ITGAE,RICTOR,WIPF1,PIK3R1,RAC2,AQP3,LCP1,FYN,INPP5D,ITK,PLCB3,HSP90AA1,PTGDR,CCR2,GPR132,STIM2,BCL11B,CD58,TNFSF14,MAP3K14,FYB1,NR1H2,ABCC1,PDCD1,CD44,PYCARD,RGS1,ARHGEF1,BCL2,XCL1,SH2D1A,CD2
Differentiation of T lymphocytes	1,31E-10	-4,56	IRF1,ICOS,CDKN2A,IL2RG,CXCR4,HMGB1,IL7R,LCK,TFAP4,LAT,WWP1,S1PR1,IL2,SMAD2,CD46,SOCS2,PTGER4,MAF,PRDM1,GBA,KLRD1,USP15,SIT1,DNAJA3,BATF,STAT5B,ETS1,RHOH,IFNG,ZBTB16,RICTOR,PTPN22,NDFIP1,ITPKB,STAT5A,CD3D,NCOR1,PRDX2,CCR2,STAT4,NOTCH1,BCL11B,CD3G,MAP3K14,JMJD6,GIMAP1,PARP1,CD40LG,BCL2,SP3,TNFRSF18,PGAP3,TBX21,DUSP10,ITGAL,PRKCQ,CARD9,GATA3,MALT1,SYK,STAT3,CD8A,CBLB,ZAP70,NR4A2,IL18R1,CYLD,GRAP2,CD3E,DNAJB1,CCL5,ZEB1,JAK1,CD69,RORA,SH2D2A,BAD,ANXA1,BRAF,CDKN1B,NCAPH2,NKAP,MAPK14,AP3B1,IL2RB,PIK3R1,CD6,ZFP36L2,IKZF1,DEF6,ITK,TOX,SLAMF6,PRKDC,HSP90AA1,GADD45G,CD8B,PREX1,ATM,PPP3CB,SLA,PDCD1,IRF2,THEMIS,TNFAIP3,SOCS1,PTPRC,CD247,SH2D1A
Maturation of leukocytes	1,58E-03	-4,33	IRF1,RHOH,IFNG,ICOS,CDKN2A,PIK3R1,RASGRP1,PHC1,LCP1,ELF1,STAT3,CXCR4,FYN,INPP5D,HMGB1,STAT5A,IL7R,LCK,ITK,LAT,DGKZ,GSTP1,CCR2,CD3E,IL2,CCL5,NOTCH1,GPR132,TNFSF14,ATM,SRSF2,MAP3K14,PRDM1,CD44,CD47,CD40LG,RUNX3,PTPRC,FAS,CD247,TBX21
Homing of mononuclear leukocytes	8,98E-03	-4,33	ITGAL,ITGAE,WIPF1,PLA2G6,IL16,RAC2,LCP1,F2R,STAT3,CXCR4,FYN,HMGB1,CREB3,AIF1,ITK,ANO6,PRKCB,LAT,PTGDR,HEBP1,S1PR1,CCR2,IL2,CCL5,TNFSF14,PTPRO,JAK1,FYB1,CD44,ANXA1,RGS1,BCL2,BATF,RAP1B,ITGA4,XCL1,STK4
Binding of leukocytes	1,45E-02	-4,27	ITGAL,PAFAH1B1,PLAU,ICOS,PRKCQ,RASGRP1,SYK,F2R,CXCR4,IL7R,LCK,CBLB,IL18R1,RAP2A,IL2,P2RY8,CCL5,TLN1,EZR,IKBKB,JAK1,PTGER4,ANXA1,CD47,ADGRE5,RAP1B,ITGA4,EVL,FAS,NKAP,STK4,SKAP1,SEMA4D,F11R,IFNG,RICTOR,PTPN22,PLA2G6,CD6,RAC2,B4GALT1,FYN,INPP5D,ITK,MGAT5,PLCB3,PRDX2,HSP90AA1,PAK2,ANGPT2,PREX1,CD58,MSN,MAP3K14,FYB1,JMJD6,CD44,CD40LG,RGS1,ARHGEF1,PTPRC,TNIP1,SH2D1A,STX4,CD2
Quantity of hematopoietic progenitor cells	9,15E-12	-4,21	IRF1,AKAP13,CDKN2A,FCER1G,RASGRP1,IL2RG,CXCR4,IL7R,LCK,LAT,LAG3,TLE4,LTB,IL2,KAT6A,SIT1,DNAJA3,RAP1B,BCL2L1,BAX,STAT5B,FAS,ETS1,STK4,EPS15,FLT3LG,CBFB,RHOH,CREB1,IFNG,WIPF1,IKZF3,ELK4,TAP1,FYN,ITPKB,CREBBP,MS4A1,NOTCH1,RAP1GDS1,BCL11B,CD5,CD3G,NABP1,EP300,MAP3K14,FYB1,LY9,MATK,GIMAP1,CD40LG,CD7,BCL2,ARHGEF1,ZBTB7A,PRKCQ,SYK,STAT3,CD8A,PIK3AP1,PRKCH,ASXL1,ZAP70,CYLD,GRAP2,CD3E,PAG1,ZEB1,JAK1,PIM1,CD69,PPARD,CDKN1B,CASP3,STK17B,IL2RB,PIK3R1,IKZF1,CCND3,INPP5D,ITK,TOX,NMT2,PRKDC,KRAS,CD8B,ATM,PPP3CB,THEMIS,NABP2,TACC3,RUNX3,SOCS1,PTPRC,KMT2E,CD247,SH2D1A
Quantity of lymphoid tissue	3,20E-11	-4,20	IRF1,ICOS,CDKN2A,FCER1G,RASGRP1,IL2RG,CXCR4,IL7R,LCK,LAT,LAG3,TLE4,LTB,IL2,PRDM1,KAT6A,SIT1,DNAJA3,RAP1B,BCL2L1,BAX,FCMR,STAT5B,FAS,ETS1,STK4,EPS15,FLT3LG,CBFB,RHOH,CREB1,IFNG,PTPN22,WIPF1,IKZF3,TAP1,ELK4,ITPKB,CREBBP,NOTCH1,RAP1GDS1,BCL11B,CD5,CD3G,EP300,MAP3K14,FYB1,LY9,GIMAP1,CD40LG,CD7,BCL2,ARHGEF1,PRKCQ,MALT1,DOCK8,STAT3,CD8A,PIK3AP1,PRKCH,ASXL1,ZAP70,CYLD,GRAP2,CD3E,PAG1,ZEB1,JAK1,PIM1,PPARD,CDKN1B,DOCK11,CASP3,STK17B,IL2RB,IKZF1,CCND3,INPP5D,ITK,TOX,NMT2,N4BP1,PRKDC,KRAS,CD8B,ATM,PPP3CB,THEMIS,TACC3,RGS1,RUNX3,SOCS1,PTPRC,KMT2E,CD247,SH2D1A,CASP8,XKR8
Adhesion of immune cells	1,51E-02	-4,18	ITGAL,PAFAH1B1,PLAU,ICOS,PRKCQ,RASGRP1,SYK,F2R,CXCR4,IL7R,LCK,CBLB,RAP2A,IL2,P2RY8,CCL5,TLN1,EZR,JAK1,PTGER4,ANXA1,CD47,ADGRE5,RAP1B,ITGA4,FAS,STK4,SKAP1,SEMA4D,F11R,IFNG,RICTOR,PTPN22,PLA2G6,CD6,RAC2,B4GALT1,FYN,INPP5D,ITK,MGAT5,PLCB3,PRDX2,HSP90AA1,PAK2,ANGPT2,PREX1,CD58,MSN,MAP3K14,FYB1,CD44,CD40LG,RGS1,ARHGEF1,PTPRC,TNIP1,SH2D1A,STX4,CD2
Quantity of thymocytes	1,07E-12	-4,07	IRF1,PRKCQ,CDKN2A,FCER1G,RASGRP1,IL2RG,STAT3,CXCR4,CD8A,IL7R,LCK,LAT,PRKCH,ASXL1,LAG3,TLE4,ZAP70,LTB,CYLD,GRAP2,CD3E,IL2,PAG1,ZEB1,JAK1,PIM1,PPARD,KAT6A,SIT1,DNAJA3,CDKN1B,CASP3,BCL2L1,BAX,ETS1,STAT5B,FAS,STK17B,STK4,FLT3LG,CBFB,RHOH,CREB1,IFNG,IL2RB,WIPF1,TAP1,ELK4,IKZF1,CCND3,INPP5D,ITPKB,ITK,TOX,CREBBP,NMT2,PRKDC,NOTCH1,CD8B,RAP1GDS1,CD5,CD3G,BCL11B,ATM,MAP3K14,EP300,PPP3CB,LY9,FYB1,THEMIS,CD40LG,TACC3,CD7,ARHGEF1,BCL2,RUNX3,SOCS1,PTPRC,KMT2E,CD247
Quantity of thymus gland	4,60E-13	-3,91	IRF1,PRKCQ,CDKN2A,FCER1G,RASGRP1,IL2RG,STAT3,CXCR4,CD8A,IL7R,LCK,LAT,PRKCH,ASXL1,LAG3,TLE4,ZAP70,LTB,CYLD,GRAP2,CD3E,IL2,PAG1,ZEB1,JAK1,PIM1,PPARD,KAT6A,SIT1,DNAJA3,CDKN1B,CASP3,BCL2L1,BAX,ETS1,STAT5B,FAS,STK17B,STK4,FLT3LG,RHOH,CBFB,CREB1,IFNG,IL2RB,WIPF1,TAP1,ELK4,IKZF1,CCND3,INPP5D,ITPKB,ITK,TOX,CREBBP,NMT2,PRKDC,NOTCH1,CD8B,RAP1GDS1,CD5,BCL11B,CD3G,ATM,MAP3K14,EP300,PPP3CB,LY9,FYB1,THEMIS,CD40LG,TACC3,CD7,ARHGEF1,BCL2,RUNX3,SOCS1,PTPRC,KMT2E,CD247,XKR8
Quantity of natural killer T lymphocytes	5,61E-11	-3,89	IRF1,RHOH,ITGAL,ATG5,IFNG,ICOS,AP3B1,PRKCQ,RICTOR,IL2RB,RASAL3,RASGRP1,IL2RG,IKZF1,ELF1,IL7R,ITK,TOX,TLE4,S1PR1,IL2,TXNIP,NOTCH1,RC3H2,BCL11B,MAP3K14,LY9,FYB1,TNFAIP3,BATF,SOCS1,PTPRC,BAX,KMT2E,ETS1,SH2D1A,CD247,TBX21
Maturation of mononuclear leukocytes	3,84E-04	-3,86	RHOH,IFNG,ICOS,CDKN2A,RASGRP1,PHC1,LCP1,ELF1,CXCR4,FYN,STAT5A,IL7R,LCK,ITK,DGKZ,LAT,CD3E,IL2,NOTCH1,GPR132,ATM,SRSF2,MAP3K14,PRDM1,RUNX3,PTPRC,CD247,TBX21
Interaction of mononuclear leukocytes	6,26E-04	-3,84	ITGAL,IFNG,PLAU,ICOS,RICTOR,CD6,RAC2,RASGRP1,B4GALT1,CXCR4,IL7R,LCK,ITK,CBLB,PRDX2,HSP90AA1,IL18R1,RAP2A,IL2,ANGPT2,CCL5,CD58,MSN,EZR,JAK1,FYB1,JMJD6,CD44,ANXA1,CD47,CD40LG,RGS1,ARHGEF1,RAP1B,ITGA4,EVL,FAS,SH2D1A,NKAP,CD2,STK4,SKAP1
Differentiation of B lymphocytes	1,15E-03	-3,82	ICOS,IL2RG,PHC1,MALT1,ELF1,SYK,STAT3,CXCR4,PIK3AP1,IL7R,LAT,POLM,CYLD,IL2,TLN1,EZR,JAK1,PRDM1,CD69,BAD,PCID2,DOCK11,ETS1,STAT5B,FLT3LG,IFNG,FOXP1,IL2RB,WIPF1,PIK3R1,IKZF3,LCP1,ZFP36L2,IKZF1,FYN,INPP5D,PPP2R3C,STAT5A,PRKDC,ITM2A,MS4A1,NOTCH1,ATM,EP300,GIMAP1,CD40LG,BCL2,SP3,PTPRC,ZBTB7A
Cell viability of T lymphocytes	3,17E-09	-3,81	GSK3B,ICOS,PRKCQ,RICTOR,IL2RB,IL2RG,ELF1,DOCK8,STAT3,F2R,DEF6,RAD9A,HMGB1,STAT5A,IL7R,LAT,PIK3C3,CYLD,CD3E,IL2,STAT4,NOTCH1,TCF7,BCL11B,ATM,LAX1,CAMK4,PIM1,PDCD1,PYCARD,GIMAP1,BCL2,CAMK2G,BCL2L1,TNFRSF18,STAT5B,FAS,CASP8
Adhesion of T lymphocytes	1,14E-05	-3,79	ITGAL,IFNG,PLAU,ICOS,RICTOR,RAC2,RASGRP1,CXCR4,IL7R,ITK,CBLB,HSP90AA1,IL2,CD58,EZR,JAK1,FYB1,CD44,ANXA1,CD47,ARHGEF1,RAP1B,ITGA4,FAS,SH2D1A,CD2,SKAP1
Interaction of T lymphocytes	2,17E-05	-3,77	ITGAL,PLAU,IFNG,ICOS,RICTOR,CD6,RAC2,RASGRP1,CXCR4,IL7R,LCK,ITK,CBLB,HSP90AA1,IL18R1,IL2,CCL5,CD58,MSN,EZR,JAK1,FYB1,JMJD6,CD44,ANXA1,CD47,ARHGEF1,RAP1B,ITGA4,FAS,EVL,SH2D1A,NKAP,CD2,STK4,SKAP1
Proliferation of lymphocytes	9,98E-18	-3,77	IRF1,AKAP13,ICOS,HNRNPL,RASGRP1,ELF1,CXCR4,HMGB1,PCYT1A,TXLNA,LCK,PRKCB,TRAF1,LAG3,S1PR1,SH3KBP1,IL2,TXNIP,TCF7,CD46,PTGER4,PRDM1,TAPBP,KLRD1,CD47,DNAJA3,BTN3A1,CDKN2B,BCLAF1,PCBP2,FAS,SLAMF1,WBP2,RICTOR,CUL1,PTPN22,NDFIP1,IKZF3,GPR174,DGKZ,PRDX2,CD27,WWP2,CD3G,EP300,PPP3CA,PARP1,CD40LG,BCL2,TNFRSF18,CD2,PGAP3,TBX21,ITGAL,PLAU,RBL2,CD320,RPS6KA5,STAT3,PIK3AP1,ANO6,CBLB,PRKCH,CYTIP,ISG20,CD3E,PRF1,SMPD1,SH2D2A,CDKN1B,STK17B,SEMA4D,MAP2K7,S1PR4,KLRB1,PLA2G6,CD6,IKZF1,CCND3,INPP5D,ITK,TOX,KAT7,EHD4,LST1,MYBL1,MARK2,ATM,TIGIT,PDCD1,CD44,IRF2,THEMIS,NCSTN,ACLY,UBASH3A,SOCS1,SH2D1A,TBC1D10C,NCOR2,FTL,CDKN2A,FCER1G,IL2RG,ZNF335,STING1,JUND,IL7R,RGS2,LAT,POLM,NCK1,SKIL,LTB,TLN1,CCND2,DLG1,EZR,SIT1,BATF,BCL2L1,ETS1,STAT5B,STK4,JUNB,FLT3LG,CBFB,RHOH,MARCHF7,IFNG,CREB1,DOK2,ZBTB16,WIPF1,LCP1,FYN,ISG15,STAT5A,PDE4D,MS4A1,CCR2,RASA2,STAT4,NOTCH1,MBP,STIM2,CD5,NABP1,MAP3K14,LY9,FYB1,GLMN,PYCARD,GIMAP1,ARHGEF1,SP3,XCL1,AHNAK,DUSP10,EIF2AK2,WASHC1,ATG5,PRKCQ,PLEKHA2,PPP2R5C,GATA3,MALT1,LPIN1,DOCK8,SYK,ARHGEF6,CD8A,ZAP70,CYLD,GRAP2,PAG1,CCL5,IKBKB,PIM1,CD69,ANXA1,PPARD,BRAF,CASP3,SKAP1,GSK3B,MAPK14,HECTD1,IL2RB,SELENOK,PIK3R1,RAC2,LY96,TSC22D3,DEF6,AIF1,MGAT5,SLAMF6,SIGIRR,KRAS,RC3H2,TNFSF14,MSN,PPP3CB,NR1H2,NABP2,TNFAIP3,MAP3K2,RUNX3,PTPRC,CD247,CASP8
Proliferation of mononuclear leukocytes	2,86E-17	-3,73	IRF1,AKAP13,ICOS,HNRNPL,RASGRP1,ELF1,CXCR4,HMGB1,PCYT1A,TXLNA,LCK,PRKCB,TRAF1,LAG3,S1PR1,SH3KBP1,IL2,TXNIP,TCF7,CD46,PTGER4,PRDM1,TAPBP,KLRD1,CD47,DNAJA3,BTN3A1,CDKN2B,BCLAF1,PCBP2,FAS,SLAMF1,WBP2,RICTOR,CUL1,PTPN22,NDFIP1,IKZF3,GPR174,DGKZ,PRDX2,WWP2,CD27,CD3G,EP300,PPP3CA,PARP1,CD40LG,BCL2,TNFRSF18,CD2,PGAP3,TBX21,ITGAL,PLAU,RBL2,CD320,RPS6KA5,STAT3,PIK3AP1,ANO6,CBLB,PRKCH,CYTIP,ISG20,CD3E,PRF1,SMPD1,SH2D2A,CDKN1B,STK17B,SEMA4D,MAP2K7,S1PR4,KLRB1,PLA2G6,CD6,IKZF1,CCND3,INPP5D,ITK,TOX,KAT7,EHD4,LST1,MYBL1,MARK2,ATM,TIGIT,PDCD1,CD44,IRF2,THEMIS,NCSTN,ACLY,UBASH3A,SOCS1,SH2D1A,TBC1D10C,NCOR2,FTL,CDKN2A,FCER1G,IL2RG,ZNF335,STING1,JUND,IL7R,RGS2,LAT,POLM,NCK1,SKIL,LTB,TLN1,CCND2,DLG1,EZR,SIT1,BATF,BCL2L1,ETS1,STAT5B,STK4,JUNB,FLT3LG,CBFB,RHOH,MARCHF7,IFNG,CREB1,DOK2,ZBTB16,WIPF1,LCP1,FYN,ISG15,STAT5A,PDE4D,MS4A1,CCR2,RASA2,STAT4,NOTCH1,MBP,STIM2,CD5,NABP1,MAP3K14,LY9,FYB1,GLMN,PYCARD,GIMAP1,ARHGEF1,SP3,XCL1,AHNAK,DUSP10,EIF2AK2,WASHC1,ATG5,PRKCQ,PLEKHA2,PPP2R5C,GATA3,MALT1,LPIN1,DOCK8,SYK,ARHGEF6,CD8A,ZAP70,CYLD,GRAP2,PAG1,CCL5,IKBKB,PIM1,CD69,ANXA1,PPARD,BRAF,CASP3,SKAP1,GSK3B,MAPK14,HECTD1,IL2RB,SELENOK,PIK3R1,RAC2,LY96,TSC22D3,DEF6,AIF1,MGAT5,SLAMF6,SIGIRR,KRAS,RC3H2,TNFSF14,MSN,PPP3CB,NR1H2,SENP1,NABP2,TNFAIP3,MAP3K2,RUNX3,PTPRC,CD247,CASP8
Binding of mononuclear leukocytes	2,13E-04	-3,72	ITGAL,PLAU,IFNG,ICOS,RICTOR,CD6,RAC2,RASGRP1,B4GALT1,CXCR4,IL7R,ITK,CBLB,PRDX2,HSP90AA1,IL18R1,RAP2A,IL2,ANGPT2,CCL5,CD58,MSN,EZR,JAK1,FYB1,JMJD6,CD44,ANXA1,CD47,CD40LG,RGS1,ARHGEF1,RAP1B,ITGA4,EVL,FAS,SH2D1A,NKAP,CD2,STK4,SKAP1
Interaction of lymphocytes	3,55E-04	-3,71	ITGAL,PLAU,IFNG,ICOS,RICTOR,CD6,RAC2,RASGRP1,CXCR4,IL7R,LCK,ITK,CBLB,HSP90AA1,IL18R1,RAP2A,IL2,CCL5,CD58,MSN,EZR,JAK1,FYB1,JMJD6,CD44,ANXA1,CD47,RGS1,ARHGEF1,RAP1B,ITGA4,EVL,FAS,SH2D1A,NKAP,CD2,STK4,SKAP1
Binding of T lymphocytes	2,82E-06	-3,66	ITGAL,PLAU,IFNG,ICOS,RICTOR,CD6,RAC2,RASGRP1,CXCR4,IL7R,ITK,CBLB,HSP90AA1,IL18R1,IL2,CCL5,CD58,MSN,EZR,JAK1,FYB1,JMJD6,CD44,ANXA1,CD47,ARHGEF1,RAP1B,ITGA4,FAS,EVL,SH2D1A,NKAP,CD2,STK4,SKAP1
Maturation of lymphocytes	1,49E-03	-3,63	RHOH,ICOS,CDKN2A,RASGRP1,PHC1,LCP1,ELF1,CXCR4,FYN,IL7R,LCK,ITK,DGKZ,LAT,CD3E,IL2,NOTCH1,ATM,SRSF2,MAP3K14,PRDM1,RUNX3,PTPRC,CD247,TBX21
Adhesion of lymphocytes	1,16E-04	-3,62	ITGAL,PLAU,IFNG,ICOS,RICTOR,RAC2,RASGRP1,CXCR4,IL7R,ITK,CBLB,HSP90AA1,RAP2A,IL2,CD58,EZR,JAK1,FYB1,CD44,ANXA1,CD47,RGS1,ARHGEF1,RAP1B,ITGA4,FAS,SH2D1A,CD2,SKAP1
Proliferation of immune cells	3,92E-16	-3,61	IRF1,AKAP13,ICOS,HNRNPL,RASGRP1,ELF1,CXCR4,HMGB1,PCYT1A,TXLNA,LCK,PRKCB,TRAF1,LAG3,S1PR1,SH3KBP1,IL2,TXNIP,TCF7,CD46,PTGER4,PRDM1,TAPBP,KLRD1,CD47,DNAJA3,BTN3A1,CDKN2B,BCLAF1,PCBP2,FAS,SLAMF1,WBP2,RICTOR,CUL1,NBEAL2,PTPN22,NDFIP1,IKZF3,GPR174,DGKZ,PRDX2,GSTP1,WWP2,CD27,CD3G,EP300,PPP3CA,PARP1,CD40LG,BCL2,TNFRSF18,CD2,PGAP3,TBX21,ITGAL,PLAU,RBL2,CD320,RPS6KA5,STAT3,PIK3AP1,ANO6,CBLB,PRKCH,CYTIP,ISG20,CD3E,PRF1,SMPD1,SH2D2A,CDKN1B,STK17B,SEMA4D,MAP2K7,S1PR4,KLRB1,PLA2G6,CD6,IKZF1,CCND3,INPP5D,ITK,TOX,KAT7,EHD4,LST1,MYBL1,MARK2,ATM,TIGIT,PDCD1,CD44,IRF2,THEMIS,NCSTN,BST2,ACLY,UBASH3A,SOCS1,SH2D1A,TBC1D10C,NCOR2,FTL,CDKN2A,FCER1G,IL2RG,ZNF335,STING1,JUND,IL7R,RGS2,LAT,POLM,NCK1,SKIL,LTB,TLN1,CCND2,DLG1,EZR,SIT1,BATF,BCL2L1,ETS1,STAT5B,STK4,JUNB,FLT3LG,CBFB,RHOH,MARCHF7,IFNG,CREB1,DOK2,ZBTB16,WIPF1,LCP1,FYN,ISG15,STAT5A,PDE4D,MS4A1,CCR2,RASA2,STAT4,NOTCH1,MBP,STIM2,CD5,NABP1,MAP3K14,LY9,FYB1,GLMN,PYCARD,GIMAP1,ARHGEF1,SP3,XCL1,AHNAK,DUSP10,EIF2AK2,WASHC1,ATG5,PRKCQ,PRKAA1,PLEKHA2,PPP2R5C,GATA3,MALT1,LPIN1,DOCK8,SYK,ARHGEF6,CD8A,ZAP70,CYLD,GRAP2,PAG1,CCL5,IKBKB,PIM1,CD69,ANXA1,PPARD,BRAF,CASP3,SKAP1,GSK3B,MAPK14,HECTD1,IL2RB,SELENOK,PIK3R1,RAC2,LY96,TSC22D3,DEF6,AIF1,MGAT5,SLAMF6,SIGIRR,KRAS,RC3H2,TNFSF14,MSN,PPP3CB,NR1H2,SENP1,NABP2,TNFAIP3,MAP3K2,RUNX3,PTPRC,CD247,CASP8

Binding of lymphocytes	7.54E-05	-3.60	ITGAL,PLAU,IFNG,ICOS,RICTOR,CD6,RAC2,RASGRP1,CXCR4,IL7R,ITK,CBLB,HSP90AA1,IL18R1,RAP2A,IL2,CCL5,CD58,MSN,EZR,JAK1,FYB1,JMJD6,CD44,ANXA1,CD47,RGS1,ARHGEF1,RAP1B,ITGA4,FAS,EVL,SH2D1A,NKAP,CD2,STK4,SKAP1
Adhesion of mononuclear leukocytes	2.08E-04	-3.59	ITGAL,PLAU,IFNG,ICOS,RICTOR,RAC2,RASGRP1,CXCR4,IL7R,ITK,CBLB,PRDX2,HSP90AA1,RAP2A,IL2,ANGPT2,CD58,EZR,JAK1,FYB1,CD44,ANXA1,CD47,CD40LG,RGS1,ARHGEF1,RAP1B,ITGA4,FAS,SH2D1A,CD2,SKAP1
Cell cycle progression	5.12E-07	-3.58	ITGAL,IFNG,ATG5,CDKN2A,CD320,IKZF3,IKZF1,STAT3,INPP5D,STAT5A,IL7R,LCK,LAG3,ZAP70,SKIL,KRAS,IL2,STAT4,ATM,NABP1,IKBKB,CD40LG,NABP2,CDKN1B,BCL2,SOCS1,PTPRC,BCL2L1,STAT5B
Cell viability of B lymphocytes	3.70E-04	-3.52	SEMA4D,RICTOR,WIPF1,MALT1,SYK,INPP5D,TSC22D3,ITPKB,STAT5A,IL7R,PRKCB,KRAS,SH3KBP1,NABP1,MAP3K14,LAX1,IKBKB,LY9,PRDM1,CD44,GIMAP1,CD40LG,NABP2,BCL2,FCMR,BCL2L1,STAT5B
T cell migration	2.88E-04	-3.51	ITGAL,IFNG,PLAU,ICOS,PRKCQ,ITGAE,HNRNPL,PIK3R1,IL16,RAC2,AQP3,LCP1,MYH9,DOCK8,STAT3,CXCR4,FYN,INPP5D,ITK,PLCB3,LAT,LAG3,HSP90AA1,ZAP70,S1PR1,CCR2,IL2,CCL5,BCL11B,TNFSF14,MAP3K14,EZR,SMPD1,JAK1,FYB1,NR1H2,ABCC1,PDCD1,CD69,CD44,CD47,RGS1,BCL2,BATF,ITGA4,XCL1,STK4
Quantity of CD8+ T lymphocyte	6.75E-11	-3.50	IRF1,PRKCQ,P2RX5,CDKN2A,PRKACB,RASGRP1,IL2RG,MALT1,DOCK8,STING1,ARHGEF6,CD8A,LCK,PRKCH,LAG3,CYLD,GRAP2,CD3E,IL2,TXNIP,SMPD1,PRF1,ZEB1,HCST,TAPBP,PPARD,SIT1,BATF,GNLY,BCLAF1,ETS1,STAT5B,STK4,RHOH,IFNG,IL2RB,PTPN22,RAC2,TAP1,ELK4,GPR18,STAT5A,CD3D,ITK,TOX,TRIB2,KRAS,CCR2,CD8B,CD5,BCL11B,MAP3K14,KLRC1,PDCD1,THEMIS,HLA-A,BCL2,PTPRC,TNFRSF18,CD247,SH2D1A,CASP8
Cell proliferation of T lymphocytes	6.73E-16	-3.49	IRF1,NCOR2,AKAP13,FTL,ICOS,CDKN2A,FCER1G,HNRNPL,RASGRP1,IL2RG,ELF1,STING1,CXCR4,JUND,HMGB1,IL7R,LCK,RGS2,LAT,TRAF1,LAG3,NCK1,SKIL,LTB,IL2,TXNIP,TLN1,DLG1,TCF7,CD46,PTGER4,PRDM1,KLRD1,CD47,SIT1,DNAJA3,BTN3A1,BATF,BCLAF1,PCBP2,BCL2L1,ETS1,STAT5B,FAS,STK4,CBFB,RHOH,MARCHF7,SLAMF1,WBP2,IFNG,CREB1,DOK2,ZBTB16,RICTOR,CUL1,PTPN22,NDFIP1,WIPF1,IKZF3,LCP1,GPR174,FYN,STAT5A,PDE4D,DGKZ,PRDX2,WWP2,CD27,CCR2,RASA2,STAT4,NOTCH1,MBP,CD5,CD3G,MAP3K14,LY9,GLMN,FYB1,PPP3CA,PYCARD,PARP1,CD40LG,BCL2,ARHGEF1,TNFRSF18,XCL1,CD2,PGAP3,AHNAK,DUSP10,EIF2AK2,ITGAL,WASHC1,ATG5,PLAU,RBL2,PRKCQ,PPP2R5C,GATA3,MALT1,DOCK8,RPS6KA5,SYK,STAT3,ARHGEF6,CD8A,ANO6,CBLB,PRKCH,CYTIP,ZAP70,ISG20,CYLD,GRAP2,CD3E,PAG1,CCL5,PRF1,SMPD1,IKBKB,PIM1,CD69,SH2D2A,ANXA1,PPARD,BRAF,CDKN1B,CASP3,STK17B,SKAP1,GSK3B,MAP2K7,MAPK14,S1PR4,KLRB1,IL2RB,SELENOK,PIK3R1,CD6,RAC2,IKZF1,CCND3,INPP5D,TSC22D3,DEF6,ITK,AIF1,MGAT5,TOX,EHD4,RC3H2,TNFSF14,ATM,MSN,PPP3CB,TIGIT,NR1H2,PDCD1,CD44,IRF2,THEMIS,NCSTN,TNFAIP3,MAP3K2,RUNX3,UBASH3A,SOCS1,PTPRC,CD247,SH2D1A,CASP8,TBC1D10C
Activation of natural killer cells	8.04E-03	-3.49	FLT3LG,IL2,ITGAL,STAT4,TXNIP,IFNG,KLRB1,PRF1,SOCS2,RASGRP1,NCR3,MALT1,HLA-A,KLRC2,STAT5B,SH2D1A,CASP8,LYST,HEBP1,IL18R1
Differentiation of helper T lymphocytes	1.19E-04	-3.34	IRF1,MAPK14,ITGAL,IFNG,ICOS,PRKCQ,RICTOR,IL2RB,NDFIP1,CARD9,CD6,MALT1,GATA3,STAT3,DEF6,HMGB1,STAT5A,ITK,CBLB,GADD45G,IL18R1,NR4A2,IL2,STAT4,DNAJB1,CCL5,NOTCH1,SMAD2,MAP3K14,SOCS2,PTGER4,PRDM1,MAF,CD69,RORA,SH2D2A,ANXA1,KLRD1,USP15,SIT1,CD40LG,CDKN1B,BATF,ETS1,TNFRSF18,SH2D1A,PGAP3,TBX21
Transmigration of mononuclear leukocytes	1.07E-02	-3.29	CCR2,F11R,ITGAL,IFNG,CCL5,CD99,RAC2,CD44,AQP3,MYH9,CD47,CD40LG,ITK,ITGA4,HSP90AA1,ZAP70
NK cell homeostasis	3.67E-07	-3.24	FLT3LG,IRF1,SLAMF1,HECTD1,IL2RB,PRDM1,TAPBP,IL2RG,IRF2,IKZF1,GATA3,STAT3,INPP5D,STAT5A,SP3,TOX,SLAMF6,KAT7,PTPRC,STAT5B,ETS1,TBX21
Expansion of lymphocytes	7.36E-07	-3.21	IRF1,ICOS,SYK,STING1,STAT3,HMGB1,IL7R,LAT,LAG3,ZAP70,S1PR1,GRAP2,CD3E,IL2,CCND2,PRF1,IKBKB,PIM1,CD69,BRAF,CDKN1B,BCL2L1,ETS1,STAT5B,FAS,CREB1,IFNG,ZBTB16,IL2RB,PTPN22,LCP1,IKZF1,CCND3,DEF6,STAT5A,ITK,TOX,CD27,SIGIRR,STAT4,MYBL1,LY9,NR1H2,PDCD1,IRF2,PARP1,CD40LG,TNFAIP3,BCL2,SOCS1,PTPRC,TNFRSF18
Flux of Ca2+	1.01E-03	-3.20	ICOS,PIK3R1,RAC2,SYK,CXCR4,INPP5D,ITK,PLCB3,NMT2,DGKZ,LAT,BACE1,HEBP1,CD3E,PAG1,CCL5,GPR132,DLG1,STIM2,CD5,EZR,WDR37,LAX1,ANXA1,PARP1,FAS,JUNB
Maturation of T lymphocytes	2.88E-05	-3.19	RHOH,IL2,CD3E,NOTCH1,ATM,SRSF2,CDKN2A,RASGRP1,ELF1,CXCR4,FYN,IL7R,LCK,ITK,RUNX3,PTPRC,DGKZ,LAT,CD247,TBX21
Generation of helper T lymphocytes	2.39E-03	-3.19	IL2,STAT4,IFNG,ICOS,CD5,SMAD2,PRKCQ,PRKAA1,MAP3K14,PPP3CA,RORA,GATA3,STAT3,STAT5A,ITK,TBX21,S1PR1
Expansion of T lymphocytes	5.27E-05	-3.16	IRF1,CREB1,IFNG,ICOS,ZBTB16,PTPN22,IKZF1,LCP1,SYK,STAT3,STING1,DEF6,HMGB1,IL7R,ITK,TOX,LAT,LAG3,CD27,ZAP70,GRAP2,CD3E,IL2,STAT4,PRF1,IKBKB,LY9,NR1H2,PIM1,PDCD1,CD69,IRF2,PARP1,BRAF,CD40LG,CDKN1B,BCL2,TNFRSF18,STAT5B,FAS
Activation of leukocytes	2.97E-09	-3.13	NCOR2,MTND5,ICOS,NCF1,FCER1G,IL16,RASGRP1,IL2RG,STARD7,STING1,HMGB1,TXLNA,LCK,PRKCB,LAT,TRAF1,LAG3,SIRPG,NCK1,LTB,SH3KBP1,IL2,TXNIP,TLN1,DLG1,CD46,LAX1,SOCS2,PTGER4,PRDM1,GBA,PTGER2,KLRD1,CD47,SIT1,BTN3A1,GNLY,AZI2,BCLAF1,BCL2L1,BAX,ETS1,STAT5B,FAS,FLT3LG,RHOH,SLAMF1,IFNG,FOXP1,PTPN22,NDFIP1,RAB6A,FYN,ITPKB,STAT5A,DGKZ,SLA2,CD27,MS4A1,CCR2,STAT4,NOTCH1,MBP,CD5,CD58,BCL11B,CD3G,CAMK4,KLRC1,PPP3CA,JMJD6,PYCARD,HLAA,CD40LG,CD7,GZMA,BCL2,VTI1B,RAB27A,TNFRSF18,CD2,LYST,AHNAK,ITGAL,ATG5,PRKCQ,PRKAA1,PLEKHA2,CARD9,RAB29,MALT1,SYK,STAT3,FUT8,ANO6,CBLB,ZAP70,IL18R1,NR4A2,CD3E,PAG1,CCL5,PRF1,SMPD1,IKBKB,MGST1,RORA,RAB8B,SH2D2A,ANXA1,RAB5B,CDKN1B,HSPH1,SEMA4D,MAPK14,PIK3IP1,TNFAIP8L2,KLRB1,AP3B1,IL2RB,TAB2,CD6,GNPAT,LY96,PILRB,ITGA1,INPP5D,CLEC2D,DEF6,KLRC2,PPP2R3C,ITK,MGAT5,UPF2,PTGDR,HEBP1,PAK2,ANGPT2,PREX1,TNFSF14,ATM,RAB34,PPP3CB,TIGIT,NR1H2,PDCD1,CD44,NCR3,TNFAIP3,SOCS1,PTPRC,KMT2E,CD247,SH2D1A,CASP8,TBC1D10C
Quantity of double-positive thymocyte	7.88E-09	-3.13	IRF1,RHOH,PRKCQ,IL2RB,CDKN2A,IL2RG,IKZF1,CCND3,CXCR4,CD8A,ITPKB,IL7R,LCK,ITK,NMT2,PRKCH,LAT,PRKDC,TLE4,ZAP70,CYLD,CD3E,GRAP2,IL2,CD5,BCL11B,ATM,ZEB1,THEMIS,PPARD,CD40LG,SIT1,CDKN1B,CD7,BCL2,PTPRC,KMT2E,ETS1,CD247,STK17B
Clonal expansion of lymphocytes	3.78E-04	-3.12	GRAP2,IL2,PARP1,SYK,STAT4,HMGB1,IFNG,LAT,CD27,ZAP70,S1PR1
Clonal expansion of T lymphocytes	6.12E-04	-3.12	GRAP2,IL2,PARP1,SYK,STAT4,HMGB1,IFNG,LAT,CD27,ZAP70
Activation of mononuclear leukocytes	4.92E-13	-3.10	NCOR2,ICOS,NCF1,FCER1G,RASGRP1,IL2RG,STING1,HMGB1,TXLNA,LCK,PRKCB,LAT,TRAF1,SIRPG,LAG3,NCK1,IL2,SH3KBP1,TXNIP,TLN1,DLG1,CD46,LAX1,SOCS2,PTGER4,PRDM1,KLRD1,CD47,SIT1,BTN3A1,BCLAF1,AZI2,BCL2L1,BAX,STAT5B,FAS,ETS1,FLT3LG,SLAMF1,IFNG,FOXP1,PTPN22,NDFIP1,FYN,ITPKB,STAT5A,DGKZ,SLA2,CD27,MS4A1,CCR2,STAT4,MBP,NOTCH1,BCL11B,CD5,CD58,CD3G,CAMK4,KLRC1,PPP3CA,PYCARD,HLAA,CD40LG,CD7,GZMA,BCL2,VTI1B,TNFRSF18,CD2,LYST,AHNAK,ITGAL,ATG5,PRKAA1,PLEKHA2,PRKCQ,RAB29,MALT1,SYK,STAT3,ANO6,FUT8,CBLB,ZAP70,NR4A2,IL18R1,CD3E,PAG1,CCL5,PRF1,SMPD1,IKBKB,MGST1,SH2D2A,ANXA1,CDKN1B,HSPH1,SEMA4D,MAPK14,PIK3IP1,TNFAIP8L2,KLRB1,IL2RB,TAB2,CD6,LY96,ITGA1,INPP5D,DEF6,CLEC2D,KLRC2,PPP2R3C,ITK,MGAT5,HEBP1,PAK2,TNFSF14,PPP3CB,TIGIT,PDCD1,CD44,NCR3,TNFAIP3,SOCS1,PTPRC,CD247,SH2D1A,CASP8,TBC1D10C
Expansion of mononuclear leukocytes	5.10E-07	-3.07	IRF1,ICOS,SYK,STING1,STAT3,HMGB1,IL7R,LAT,LAG3,ZAP70,S1PR1,GRAP2,CD3E,IL2,CCND2,PRF1,IKBKB,PIM1,CD69,BRAF,CDKN1B,BCL2L1,STAT5B,ETS1,FAS,FLT3LG,IFNG,CREB1,ZBTB16,IL2RB,PTPN22,LCP1,IKZF1,CCND3,DEF6,STAT5A,ITK,TOX,CD27,SIGIRR,STAT4,MYBL1,LY9,NR1H2,SENP1,PDCD1,IRF2,PARP1,CD40LG,TNFAIP3,BCL2,SOCS1,PTPRC,TNFRSF18
Development of B lymphocytes	2.43E-03	-2.99	FLT3LG,ITGAL,IFNG,ICOS,PLEKHA2,RICTOR,TAB2,PIK3R1,IL2RG,PHC1,CCND3,DOCK8,SYK,CXCR4,INPP5D,FYN,ITPKB,STAT5A,IL7R,FUT8,LTB,IL2,EP300,IKBKB,PRDM1,CD69,RORA,NCSTN,CD40LG,BCL2,ITGA4,BCL2L1
Transmigration of T lymphocytes	1.66E-02	-2.98	CD47,CCL5,IFNG,ITK,HSP90AA1,RAC2,CD44,AQP3,ZAP70,MYH9
NK cell development	1.18E-06	-2.95	FLT3LG,IRF1,SLAMF1,HECTD1,IL2RB,TAPBP,IL2RG,IRF2,IKZF1,GATA3,STAT3,INPP5D,STAT5A,SP3,TOX,SLAMF6,KAT7,PTPRC,STAT5B,ETS1,TBX21

Development of innate lymphoid cells	1.61E-07	-2.93	FLT3LG,IRF1,SLAMF1,ZBTB16,HECTD1,IL2RB,TAPBP,IL2RG,IRF2,IKZF1,GATA3,STAT3,INPP5D,STAT5A,SP3,TOX,SLAMF6,KAT7,PTPRC,STAT5B,ETS1,SH2D1A,TBX21
Cell movement of T lymphocytes	5.23E-04	-2.93	IFNG,ICOS,ITGAE,HNRNPL,PIK3R1,IL16,RAC2,AQP3,LCP1,MYH9,DOCK8,STAT3,CXCR4,FYN,INPP5D,ITK,PLCB3,LAT,LAG3,HSP90AA1,ZAP70,S1PR1,CCR2,IL2,CCL5,BCL11B,TNFSF14,SMPD1,JAK1,FYB1,NR1H2,PDCD1,CD69,CD44,CD47,RGS1,BCL2,BATF,ITGA4,XCL1,STK4
Cell viability of hematopoietic progenitor cells	4.55E-03	-2.93	CD3E,IL2,TCF7,ATM,NABP1,GIMAP1,NABP2,RAD9A,BCL2,STAT5A,IL7R,PSMD1,BCL2L1,STAT5B
Quantity of invariant natural killer T cells	6.53E-04	-2.92	NOTCH1,ICOS,ATG5,BCL11B,RICTOR,FYB1,LY9,RASGRP1,ELF1,TNFAIP3,IL7R,BAX,ETS1
Activation of lymphocytes	3.62E-13	-2.88	NCOR2,ICOS,NCF1,FCER1G,RASGRP1,IL2RG,STING1,HMGB1,TXLNA,LCK,PRKCB,LAT,TRAF1,SIRPG,LAG3,NCK1,SH3KBP1,IL2,TXNIP,TLN1,DLG1,CD46,LAX1,SOCS2,PTGER4,PRDM1,KLRD1,CD47,SIT1,BTN3A1,BCLAF1,AZI2,BCL2L1,BAX,STAT5B,FAS,ETS1,FLT3LG,SLAMF1,IFNG,PTPN22,NDFIP1,FYN,ITPKB,STAT5A,DGKZ,SLA2,CD27,MS4A1,CCR2,STAT4,MBP,NOTCH1,BCL11B,CD5,CD58,CD3G,CAMK4,KLRC1,PPP3CA,PYCARD,HLAA,CD40LG,CD7,GZMA,BCL2,VTI1B,TNFRSF18,CD2,LYST,AHNAK,ITGAL,ATG5,PRKAA1,PLEKHA2,PRKCQ,RAB29,MALT1,SYK,STAT3,ANO6,FUT8,CBLB,ZAP70,NR4A2,IL18R1,CD3E,PAG1,CCL5,PRF1,SMPD1,IKBKB,MGST1,SH2D2A,ANXA1,CDKN1B,HSPH1,SEMA4D,MAPK14,PIK3IP1,TNFAIP8L2,KLRB1,IL2RB,TAB2,CD6,INPP5D,DEF6,CLEC2D,KLRC2,PPP2R3C,ITK,MGAT5,HEBP1,PAK2,TNFSF14,PPP3CB,TIGIT,PDCD1,CD44,NCR3,TNFAIP3,SOCS1,PTPRC,CD247,SH2D1A,CASP8,TBC1D10C
Cell death of immune cells	1.54E-10	-2.88	IRF1,ICOS,CDKN2A,FCER1G,IL2RG,ELF1,F2R,CXCR4,STING1,HMGB1,IL7R,LCK,PRKCB,LAT,TRAF1,LAG3,TLE4,SKIL,LTB,S1PR1,IL2,SH3KBP1,TLN1,TCF7,EZR,LAX1,PTGER4,PRDM1,ZC3H8,CD47,DNAJA3,CAMK2G,BIRC6,BCL2L1,FCMR,BAX,STAT5B,FAS,ETS1,YWHAZ,STK4,FLT3LG,RHOH,SLC6A6,CREB1,IFNG,FOXP1,ZBTB16,RICTOR,PTPN22,WIPF1,H12,FYN,RAD9A,ITPKB,ISG15,STAT5A,WWP2,CD27,MS4A1,CASP10,STAT4,NOTCH1,GPR132,RAP1GDS1,BCL11B,CD5,CD99,NABP1,EP300,MAP3K14,CAMK4,LY9,OGT,KLRC1,PYCARD,GIMAP1,PARP1,CD40LG,SOAT1,GZMA,BCL2,ARHGEF1,TNFRSF18,ORMDL3,XCL1,CD2,ANKZF1,EIF2AK2,ITGAL,ATG5,PRKCQ,PRKAA1,NCF2,MALT1,DOCK8,SYK,STAT3,NOC2L,PIK3AP1,DUSP4,ZAP70,PIK3C3,CYLD,GRAP2,CD3E,CCL5,SMPD1,PRF1,IKBKB,PIM1,SH2D2A,BAD,ANXA1,CDKN1B,PSMD1,CASP3,STK17B,SEMA4D,GSK3B,GIMAP4,MAPK14,TNFAIP8L2,IL2RB,PIK3R1,CD6,RAC2,INPP5D,TSC22D3,DEF6,ITK,MGAT5,PRKDC,GADD45G,KRAS,ANGPT2,ATM,DFFA,PPP3CB,PDCD1,CD44,CASP6,NABP2,TNFAIP3,MAP3K2,SOCS1,PPP2R2B,PTPRC,CD247,SH2D1A,CASP8,XKR8
Differentiation of Th1 cells	7.17E-03	-2.86	IRF1,IL2,ITGAL,STAT4,IFNG,ICOS,PRKCQ,CD6,MAF,SH2D2A,ANXA1,KLRD1,CD40LG,SIT1,HMGB1,TBX21,GADD45G,IL18R1
Homing of T lymphocytes	2.86E-03	-2.84	IL2,CCR2,CCL5,TNFSF14,ITGAE,JAK1,FYB1,IL16,RAC2,CD44,LCP1,STAT3,CXCR4,FYN,RGS1,BCL2,ITK,BATF,ITGA4,LAT,XCL1,STK4,S1PR1
Response of lymphocytes	9.03E-05	-2.82	FLT3LG,MAPK14,IFNG,ICOS,SIPA1,KLRB1,PIK3R1,HSDL1,MALT1,DOCK8,STAT3,FYN,ITPKB,STAT5A,IL7R,ITK,CBLB,CD27,LTB,CD3E,IL2,CD5,SMAD2,LAX1,IKBKB,TIGIT,PDCD1,NCR3,PYCARD,PARP1,CD40LG,CDKN1B,CASP3,PTPRC,TNFRSF18,STAT5B,ETS1,SH2D1A,PGAP3,TBX21,CASP8
Stimulation of leukocytes	7.72E-03	-2.78	FLT3LG,SLAMF1,IFNG,ICOS,CD320,PIK3R1,FYN,TXLNA,LCK,ITK,IL18R1,CD3E,GRAP2,IL2,PAK2,MBP,CD5,TNFSF14,SMAD2,KLRC1,PDCD1,PYCARD,CD40LG,TNFAIP3,CDKN2B,PTPRC,FAS,CD2
Maintenance of leukocytes	2.32E-03	-2.78	GATA3,IL2,CXCR4,ICOS,IL7R,ITK,PRDM1,PIK3C3
Development of helper T lymphocytes	2.39E-03	-2.77	IL2,STAT4,NOTCH1,IFNG,ICOS,CD5,SMAD2,PRKCQ,PRKAA1,MAP3K14,PPP3CA,RORA,GATA3,STAT3,STAT5A,ITK,TBX21
Chemotaxis of PBMCs	2.45E-02	-2.75	CCR2,IL2,CXCR4,F2R,CCL5,PLA2G6,ANXA1,LCP1
Quantity of natural killer cells	4.06E-06	-2.71	FLT3LG,IRF1,RHOH,IFNG,P2RX5,IL2RB,FCER1G,SPPL3,IL2RG,IKZF1,GATA3,CXCR4,INPP5D,ISG15,STAT5A,TOX,PRKDC,LTB,IL2,ANGPT2,TXNIP,PRF1,HCST,PYCARD,TNFAIP3,BCL2,PTPRC,ETS1,KMT2E,STAT5B,CD247,TBX21
Mobilization of Ca2+	8.39E-03	-2.71	PRKCQ,WIPF1,PIK3R1,CD6,SLC9A3R1,CXCR4,FYN,DEF6,PIK3AP1,LCK,ITK,MGAT5,CBLB,LAT,PTGDR,ZAP70,HEBP1,MS4A1,GRAP2,CD3E,CCL5,CD5,SH2D2A,NCR3,PTPRC,CD2
Response of mononuclear leukocytes	1.22E-04	-2.70	FLT3LG,MAPK14,IFNG,ICOS,SIPA1,KLRB1,PIK3R1,HSDL1,MALT1,DOCK8,STAT3,FYN,ITPKB,STAT5A,IL7R,ITK,CBLB,SLA2,CD27,LTB,CD3E,IL2,CD5,SMAD2,LAX1,IKBKB,TIGIT,PDCD1,TAPBP,CD69,NCR3,PYCARD,PARP1,CD40LG,CDKN1B,CASP3,PTPRC,BCL2L1,BAX,STAT5B,ETS1,TNFRSF18,FAS,SH2D1A,PGAP3,TBX21,CASP8
Quantity of pro-B lymphocytes	8.99E-04	-2.68	FLT3LG,CYLD,KRAS,IL2,NABP1,PIK3R1,PIM1,IL2RG,IKZF1,SYK,CXCR4,INPP5D,NABP2,BCL2,IL7R,RAP1B,KMT2E,ZBTB7A,STAT5B,ETS1
Quantity of pre-B lymphocytes	9.77E-05	-2.65	FLT3LG,AKAP13,PIK3R1,IKZF3,IL2RG,IKZF1,CCND3,SYK,CXCR4,INPP5D,FYN,PIK3AP1,IL7R,ASXL1,CYLD,MS4A1,KRAS,IL2,ATM,EP300,MAP3K14,NABP1,LY9,PIM1,MATK,CD69,GIMAP1,NABP2,BCL2,RAP1B,KMT2E,ZBTB7A,STAT5B,ETS1,SH2D1A,EPS15
Contact growth inhibition	3.50E-05	-2.63	IFNG,CDKN1B,LCK,PTPRC,IKZF3,ZAP70,IKZF1
Cell movement of B lymphocytes	3.18E-03	-2.63	CCR2,IL2,ITGAL,ICOS,MAP3K14,WIPF1,NR1H2,PIK3R1,RAC2,LCP1,CXCR4,INPP5D,RGS1,HMGB1,ARHGEF1,RAP1B,STK4,ZAP70,RAP2A,S1PR1
T cell response	1.07E-07	-2.63	FLT3LG,MAPK14,IFNG,ICOS,SIPA1,PIK3R1,HSDL1,MALT1,DOCK8,STAT3,FYN,STAT5A,IL7R,ITK,CBLB,CD27,LTB,CD3E,IL2,CD5,SMAD2,LAX1,IKBKB,TIGIT,PDCD1,PYCARD,PARP1,CD40LG,CDKN1B,CASP3,PTPRC,TNFRSF18,ETS1,STAT5B,SH2D1A,PGAP3,TBX21,CASP8
Differentiation of gamma-delta T lymphocytes	1.27E-05	-2.63	IL2,SYK,NOTCH1,IL7R,LCK,STAT5A,LAT,PTPRC,STAT5B,IL2RG
Development of hematopoietic system	2.06E-08	-2.62	IRF1,ITGAL,ICOS,RASGRP4,RASGRP1,IL2RG,GATA3,SYK,CXCR4,IL7R,LCK,FUT8,LAT,ZAP70,LTB,GRAP2,CD3E,IL2,TCF7,IKBKB,PIM1,PBXIP1,SIT1,CDKN1B,TRAT1,BAX,ETS1,FLT3LG,CBFB,CREB1,IFNG,RICTOR,IL2RB,PIK3R1,IKZF1,CCND3,FYN,STAT5A,CD3D,ITK,NCOR1,TRAF3IP3,NOTCH1,CD5,CD3G,SENP1,PDCD1,THEMIS,BCL2,RUNX3,SOCS1,PTPRC
Maintenance of T lymphocytes	5.39E-03	-2.60	GATA3,IL2,CXCR4,ICOS,IL7R,ITK,PIK3C3
Cell death of antigen presenting cells	7.00E-03	-2.60	GSK3B,EIF2AK2,FLT3LG,IRF1,MAPK14,IFNG,CDKN2A,STAT3,STING1,INPP5D,PIK3AP1,HMGB1,ISG15,IL2,CCL5,GPR132,SMPD1,DFFA,IKBKB,PTGER4,OGT,PYCARD,PARP1,CD40LG,SOAT1,TNFAIP3,CDKN1B,BCL2,CAMK2G,CASP3,BCL2L1,FAS,CASP8,STK4
Apoptosis of antigen presenting cells	7.01E-03	-2.55	GSK3B,EIF2AK2,FLT3LG,IRF1,MAPK14,IFNG,CDKN2A,STAT3,INPP5D,PIK3AP1,HMGB1,ISG15,IL2,CCL5,GPR132,SMPD1,DFFA,PTGER4,CD40LG,SOAT1,TNFAIP3,CDKN1B,BCL2,CAMK2G,CASP3,BCL2L1,FAS,CASP8

Proliferation of PBMCs	1.39E-02	-2.53	KRAS,SH3KBP1,IL2,LST1,IL2RB,PLA2G6,PIK3R1,CD6,PDCD1,ANXA1,CD40LG,FYN,BRAF,BCL2,ITK,BATF
Development of hematopoietic progenitor cells	4.45E-08	-2.50	IRF1,ITGAL,ICOS,RASGRP4,RASGRP1,IL2RG,GATA3,SYK,CXCR4,IL7R,LCK,FUT8,LAT,ZAP70,CD3E,GRAP2,IL2,TCF7,IKBKB,PIM1,PBXIP1,SIT1,CDKN1B,TRAT1,BAX,ETS1,FLT3LG,CBFB,CREB1,IFNG,RICTOR,IL2RB,PIK3R1,IKZF1,CCND3,FYN,CD3D,STAT5A,ITK,NCOR1,TRAF3IP3,NOTCH1,CD3G,CD5,PDCD1,THEMIS,BCL2,SOCS1,PTPRC
Contact growth inhibition of lymphocytes	2.10E-05	-2.45	CDKN1B,LCK,PTPRC,IKZF3,ZAP70,IKZF1
Transendothelial migration of T lymphocytes	9.99E-03	-2.43	CD47,ITK,RAC2,AQP3,CD44,ZAP70,MYH9
Proliferation of Th1 cells	1.71E-03	-2.43	KLRD1,IL2,PARP1,CD40LG,ICOS,IFNG,CD6
Maturation of thymocytes	5.98E-03	-2.42	CD3E,CXCR4,NOTCH1,LCK,DGKZ,PTPRC,CD247,RASGRP1
Maturation of hematopoietic progenitor cells	1.84E-02	-2.42	CD3E,CXCR4,NOTCH1,LCK,PTPRC,DGKZ,CD247,RASGRP1,PHC1
Cytolysis	2.45E-02	-2.41	HLA-A,LCK,KLRB1,GNLY,PRF1,PTPRC,SH2D1A,CD69
Quantity of regulatory T lymphocytes	5.67E-05	-2.40	MAPK14,AKT3,TOB1,IFNG,ICOS,PRKCQ,RICTOR,IL2RB,PTPN22,MALT1,STAT3,STAT5A,IL7R,CD27,PTGDR,LTB,CYLD,CCR2,IL2,TRAF3IP3,TXNIP,MBP,TLN1,CD5,MAP3K14,IKBKB,CAMK4,PIKFYVE,PDCD1,CD69,THEMIS,PARP1,CD40LG,BATF,STAT5B,XCL1,STK4
Differentiation of effector T lymphocytes	6.76E-04	-2.40	IL2,STAT3,STAT4,DEF6,ICOS,CDKN1B,RICTOR,IL2RB,CD69
Stimulation of lymphocytes	1.92E-03	-2.37	SLAMF1,IFNG,ICOS,CD320,PIK3R1,FYN,TXLNA,LCK,ITK,IL18R1,GRAP2,CD3E,IL2,PAK2,MBP,CD5,TNFSF14,SMAD2,KLRC1,PDCD1,PYCARD,CD40LG,TNFAIP3,CDKN2B,PTPRC,FAS,CD2
Differentiation of naive lymphocytes	8.91E-03	-2.37	IL2,STAT4,IFNG,ICOS,PRKCQ,MAF,GATA3,MALT1,SYK,STAT3,CD40LG,DEF6,BATF,TBX21
Cell cycle progression of leukocytes	3.82E-05	-2.36	KRAS,IL2,ITGAL,ATG5,ATM,NABP1,CDKN2A,IKBKB,CD320,STAT3,CD40LG,INPP5D,NABP2,CDKN1B,BCL2,STAT5A,SOCS1,BCL2L1,STAT5B,LAG3,SKIL
Cell movement of peripheral blood lymphocytes	7.85E-03	-2.31	CD47,IL2,CXCR4,RGS1,CCL5,CLTC,PLCB3,FYB1,LCP1
Quantity of TREG cells	5.44E-03	-2.30	CCR2,IL2,IFNG,TLN1,ICOS,PRKCQ,RICTOR,IL2RB,CAMK4,PTPN22,PIKFYVE,CD69,MALT1,STAT3,IL7R,BATF,CD27,STK4
Immune response of leukocytes	2.65E-03	-2.30	FLT3LG,ITGAL,IFNG,PLAU,ICOS,PRKAA1,KLRB1,FCER1G,HSDL1,MALT1,DOCK8,SYK,F2R,STAT3,GPR18,HMGB1,ISG15,IL7R,ITK,SLA2,CD27,ABCA7,BRD2,IL2,BRD3,IKBKB,TIGIT,PDCD1,TAPBP,CD69,CD44,NCR3,PYCARD,IRF2,CD47,PARP1,CD40LG,CDKN1B,ITGA4,BCL2L1,BAX,ETS1,FAS,SH2D1A,ERN1,PGAP3,TBX21,CASP8
NK cell proliferation	8.58E-07	-2.28	FLT3LG,IRF1,SLAMF1,ICOS,KLRB1,HECTD1,IL2RB,IL2RG,IKZF1,GATA3,STAT3,INPP5D,STAT5A,TOX,KAT7,SLAMF6,CD27,IL2,TCF7,PRF1,TAPBP,IRF2,TNFAIP3,SP3,SOCS1,PTPRC,ETS1,STAT5B,TBX21
Proliferation of CD4+ T-lymphocytes	3.24E-03	-2.24	IL2,S1PR4,WBP2,ATG5,IFNG,TLN1,CD5,RICTOR,NDFIP1,FYB1,TIGIT,CD6,PDCD1,CD69,PPARD,STAT3,PARP1,BTN3A1,IL7R,ITK,BCLAF1,PTPRC,CYTIP,EHD4
Polarization of T lymphocytes	1.84E-02	-2.24	GSK3B,SLAMF1,STAT4,IFNG,ICOS,MSN,SMPD1,WIPF1,CD6,LCP1,MYH9,CXCR4,FYN
Migration of mast cells	2.12E-02	-2.21	MS4A4A,FYN,CCL5,PIK3R1,RAC2
Recruitment of lymphocytes	1.48E-02	-2.21	CCR2,IL2,MAPK14,GPR132,IFNG,CCL5,PREX1,ITGAE,IL16,PYCARD,CD47,NRAS,CXCR4,CD8A,HMGB1,STAT5A,ETS1,FAS,S1PR1
Trafficking of leukocytes	1.66E-02	-2.21	CCR2,CCL5,IFNG,TNFSF14,RAP1B,ITGA4,EVL,AQP3,ANXA1,S1PR1
Quantity of Single positive thymocytes	9.37E-03	-2.21	RHOH,GRAP2,IL2,ATM,PPP3CB,ELK4,IL2RG,THEMIS,BCL2,IL7R,LCK,ITK,NMT2,LAG3,CD247,PRKDC,STK4
Homeostasis of regulatory T lymphocytes	2.25E-03	-2.20	GATA3,IL2,ICOS,LAG3,TBX21
Cell death of phagocytes	1.00E-02	-2.20	GSK3B,EIF2AK2,FLT3LG,IRF1,MAPK14,IFNG,CDKN2A,FCER1G,SYK,STING1,STAT3,CXCR4,INPP5D,PIK3AP1,HMGB1,ISG15,STAT5A,IL2,ANGPT2,CCL5,GPR132,SMPD1,DFFA,IKBKB,PTGER4,OGT,CD44,ANXA1,PYCARD,PARP1,SOAT1,CD40LG,TNFAIP3,CDKN1B,BCL2,CAMK2G,CASP3,SOCS1,BCL2L1,BAX,FAS,CASP8,STK4,XKR8
Differentiation of cytotoxic T cells	1.25E-02	-2.19	IL2,STAT3,CD8A,IL2RB,PRDM1,HSP90AA1
Cell viability of thymocytes	2.45E-02	-2.18	IL2,CD3E,TCF7,RAD9A,BCL2,ATM,BCL2L1,GIMAP1
Stimulation of T lymphocytes	2.50E-03	-2.17	SLAMF1,IFNG,ICOS,PIK3R1,FYN,LCK,ITK,IL18R1,CD3E,GRAP2,IL2,PAK2,MBP,CD5,TNFSF14,SMAD2,KLRC1,PDCD1,PYCARD,CD40LG,TNFAIP3,CDKN2B,PTPRC,FAS,CD2

Expansion of helper T lymphocytes	3.61E-03	-2.16	IL2,PARP1,STAT3,CD40LG,ICOS,IFNG,IL7R,CD69,LCP1
Quantity of B-1 lymphocytes	1.87E-02	-2.11	SEMA4D,CYLD,MS4A1,FLT3LG,KRAS,SMAD2,LY9,CD6,PDCD1,RAC2,IKZF1,GIMAP1,MALT1,INPP5D,PIK3AP1,ARHGEF1,IL7R,PRKCB,FCMR,ORMDL3,FAS
Influx of Ca2+	3.56E-03	-2.06	PARP1,CXCR4,INPP5D,CCL5,DLG1,STIM2,CD5,ITK,LAT,NMT2,FAS,JUNB
Cell movement of mast cells	1.38E-02	-2.04	SEMA4D,GSK3B,MS4A4A,FYN,CCL5,PLAU,RICTOR,PIK3R1,IL16,RAC2
Proliferation of hematopoietic progenitor cells	7.64E-05	-2.03	ATG5,ZBTB16,RICTOR,CDKN2A,HNRNPL,RASGRP1,IL2RG,IKZF1,LPIN1,SYK,FYN,IL7R,LCK,ITK,TOX,ZAP70,LTB,S1PR1,CD3E,GRAP2,IL2,PAG1,NOTCH1,MAP3K14,IKBKB,LY9,TNFAIP3,DNAJA3,CDKN1B,BCL2,SOCS1,BCL2L1,CD2
Cell cycle progression of lymphocytes	6.03E-05	-2.01	KRAS,IL2,ITGAL,ATG5,ATM,NABP1,CDKN2A,IKBKB,CD320,CD40LG,INPP5D,NABP2,CDKN1B,BCL2,STAT5A,BCL2L1,STAT5B,LAG3,SKIL
Proliferation of peripheral blood lymphocytes	1.12E-02	-2.00	SH3KBP1,IL2,BRAF,FYN,BCL2,BATF,ITK,PLA2G6,PIK3R1,CD6,PDCD1,ANXA1
Activation of thymocytes	5.25E-06	-2.00	CD3E,IL2,FYN,ITPKB,CD58,LCK,PTPN22,LAT,CD247,CD2,IL2RG
Differentiation of natural killer T lymphocytes	4.18E-03	-2.00	ZBTB16,AP3B1,ITK,SLAMF6,SH2D1A,IKZF1
Chemotaxis of peripheral blood lymphocytes	1.04E-02	-2.00	IL2,CXCR4,CCL5,LCP1
Differentiation of effector helper T lymphocyte	1.70E-02	-2.00	IL2,ICOS,RICTOR,IL2RB
Proliferation of thymocytes	9.16E-06	-1.99	ZBTB16,RICTOR,HNRNPL,RASGRP1,IL2RG,IKZF1,SYK,FYN,IL7R,LCK,ITK,TOX,ZAP70,LTB,IL2,CD3E,GRAP2,PAG1,NOTCH1,MAP3K14,IKBKB,LY9,TNFAIP3,DNAJA3,CDKN1B,SOCS1,CD2
Apoptosis of phagocytes	1.39E-02	-1.99	GSK3B,EIF2AK2,FLT3LG,IRF1,MAPK14,IFNG,CDKN2A,FCER1G,SYK,STAT3,CXCR4,INPP5D,PIK3AP1,HMGB1,ISG15,STAT5A,IL2,CCL5,GPR132,SMPD1,DFFA,PTGER4,ANXA1,SOAT1,CD40LG,TNFAIP3,CDKN1B,CAMK2G,BCL2,CASP3,SOCS1,BCL2L1,BAX,FAS,CASP8,XKR8
Clearance of cells	1.08E-03	-1.98	PPARD,CCR2,HMGB1,GPR132,IFNG,CD44,XKR8,ANXA1
Polarization of Th1 cells	1.04E-02	-1.98	STAT4,ICOS,IFNG,CD6
Homeostasis of TREG cells	1.04E-02	-1.98	GATA3,IL2,ICOS,TBX21
Survival of natural killer T lymphocytes	1.70E-02	-1.96	CYLD,ELF1,BCL11B,IL2RG
Proliferation of B lymphocytes	5.32E-06	-1.96	RBL2,PLEKHA2,CDKN2A,CD320,RASGRP1,IL2RG,MALT1,LPIN1,SYK,ARHGEF6,PIK3AP1,PCYT1A,TXLNA,IL7R,PRKCB,CBLB,POLM,LAG3,S1PR1,CD3E,IL2,SH3KBP1,CCND2,EZR,IKBKB,PIM1,PRDM1,CDKN1B,CASP3,BCL2L1,STAT5B,ETS1,FAS,JUNB,FLT3LG,SEMA4D,IFNG,CREB1,IL2RB,WIPF1,PIK3R1,CD6,IKZF3,LCP1,CCND3,LY96,FYN,INPP5D,STAT5A,MS4A1,SIGIRR,KRAS,MYBL1,STIM2,MARK2,ATM,MAP3K14,NABP1,EP300,LY9,PDCD1,CD44,GIMAP1,IRF2,CD40LG,NABP2,TNFAIP3,ARHGEF1,BCL2,ACLY,PTPRC,PGAP3,CASP8
Cytotoxicity of T lymphocytes	5.62E-04	-1.96	IL2,STAT4,NOTCH1,IFNG,CCL5,ATG5,PRF1,KLRC1,HLA-F,KLRD1,HLA-A,CD40LG,GZMA,FUT8,CBLB,PTPRC,FAS,XCL1,CD27
Development of body trunk	1.33E-09	-1.93	IRF1,CBFB,ITGAL,CREB1,RASGRP4,RICTOR,IL2RB,PLA2G6,RASGRP1,IL2RG,IKZF1,GATA3,SYK,CXCR4,CD3D,STAT5A,IL7R,LCK,ITK,NCOR1,LAT,ZAP70,GRAP2,CD3E,IL2,TRAF3IP3,NOTCH1,CD3G,CD5,IKBKB,PIM1,PDCD1,THEMIS,SIT1,CDKN1B,TRAT1,BCL2,SOCS1,PTPRC,BAX,ETS1
Cell movement of hematopoietic progenitor cells	1.98E-02	-1.90	CCR2,DOCK8,CXCR4,RGS1,CCL5,HNRNPL,CD69,STK4,S1PR1,LCP1
Inhibition of leukocytes	2.29E-06	-1.87	IL2,AKT3,IFNG,KLRB1,EP300,SMPD1,PTGER4,CD6,PIM1,PDCD1,GATA3,PARP1,STAT3,HLA-A,FKBP5,CLEC2D,ITGA4,CBLB,DGKZ,TNFRSF18,TBX21
Quantity of CD4+ T-lymphocytes	4.33E-10	-1.83	IRF1,ITGAL,TOB1,ICOS,PRKCQ,CDKN2A,FCER1G,PRKACB,RASGRP1,STARD7,MALT1,DOCK8,STING1,STAT3,ARHGEF6,PIK3AP1,LCK,PRKCB,LAT,PRKCH,ASXL1,LAG3,ZAP70,CYLD,GRAP2,CD3E,IL2,CCL5,PRF1,IKBKB,CD69,PPARD,SIT1,BATF,GNLY,ETS1,STK17B,STK4,SEMA4D,RHOH,IFNG,ZBTB20,IL13RA1,IL2RB,PTPN22,NDFIP1,RAC2,ELK4,FYN,CD3D,ITK,TOX,SLAMF6,PTGDR,KRAS,CCR2,GPR132,CD5,ATM,MSN,MAP3K14,SLA,PDCD1,PYCARD,THEMIS,PARP1,HLA-A,CD40LG,BCL2,PTPRC,TNIP1,CD247,SH2D1A,TBX21,CASP8
Cell movement of peripheral T lymphocyte	5.39E-03	-1.82	CD47,CXCR4,RGS1,CCL5,PLCB3,FYB1,LCP1
Apoptosis of macrophages	1.28E-02	-1.75	EIF2AK2,IRF1,MAPK14,CCL5,GPR132,IFNG,DFFA,CDKN2A,STAT3,INPP5D,SOAT1,PIK3AP1,HMGB1,BCL2,ISG15,CAMK2G,CASP3,BCL2L1,FAS,CASP8
Activation of T lymphocytes	2.52E-13	-1.74	NCOR2,ICOS,NCF1,FCER1G,RASGRP1,IL2RG,HMGB1,LCK,PRKCB,LAT,TRAF1,SIRPG,LAG3,NCK1,IL2,TLN1,DLG1,CD46,LAX1,PTGER4,PRDM1,KLRD1,CD47,SIT1,BTN3A1,BCLAF1,AZI2,BCL2L1,BAX,STAT5B,FAS,ETS1,FLT3LG,SLAMF1,IFNG,PTPN22,NDFIP1,FYN,ITPKB,STAT5A,DGKZ,SLA2,CD27,CCR2,STAT4,MBP,NOTCH1,BCL11B,CD5,CD58,CD3G,CAMK4,KLRC1,PPP3CA,PYCARD,HLAA,CD40LG,CD7,GZMA,BCL2,VTI1B,TNFRSF18,CD2,AHNAK,ITGAL,ATG5,PRKAA1,PRKCQ,RAB29,MALT1,STAT3,ANO6,FUT8,CBLB,ZAP70,NR4A2,CD3E,PAG1,CCL5,PRF1,SMPD1,IKBKB,MGST1,SH2D2A,ANXA1,CDKN1B,HSPH1,SEMA4D,MAPK14,PIK3IP1,TNFAIP8L2,IL2RB,CD6,DEF6,ITK,MGAT5,PAK2,TNFSF14,PPP3CB,TIGIT,PDCD1,CD44,SOCS1,PTPRC,CD247,CASP8,TBC1D10C

Production of lymphocytes	1.98E-02	-1.68	ARID4A,GATA3,CD40LG,FYN,NOTCH1,RBL2,IL2RB,ITGA4,ETS1,TBX21
Production of leukocytes	1.55E-02	-1.68	ARID4A,FLT3LG,GATA3,CD40LG,INPP5D,FYN,NOTCH1,RBL2,IL2RB,ITGA4,ETS1,TBX21
Binding of B lymphocytes	1.14E-02	-1.67	CD47,IL2,CXCR4,ITGAL,RGS1,RAP1B,ITGA4,SH2D1A,JMJD6,RAP2A
Proliferation of alpha-beta T lymphocytes	1.25E-02	-1.67	CD3E,CCR2,SYK,CBLB,PTPRC,ZAP70
Quantity of follicular B lymphocytes	7.72E-03	-1.67	ICOS,PTPN22,WIPF1,IKZF3,IKZF1,MALT1,DOCK8,STAT3,PIK3AP1,ITK,N4BP1,TLE4,LTB,CYLD,KRAS,IL2,MAP3K14,EP300,PRDM1,GIMAP1,CD40LG,RGS1,ARHGEF1,BCL2,DOCK11,RAP1B,FCMR,FAS,SH2D1A,CASP8,STK4,EPS15
Migration of peripheral blood lymphocytes	1.31E-02	-1.66	CD47,CXCR4,RGS1,CCL5,CLTC,PLCB3,LCP1
Inhibition of lymphocytes	2.12E-05	-1.65	IL2,AKT3,KLRB1,SMPD1,EP300,PTGER4,PIM1,CD6,PDCD1,GATA3,PARP1,HLA-A,CLEC2D,CBLB,DGKZ,TNFRSF18,TBX21
Selection of T lymphocytes	5.81E-07	-1.64	RHOH,CD3E,TRAF3IP3,ATG5,PRKCQ,ELK4,ELK1,THEMIS,GATA3,ELF1,SIT1,ITPKB,TRAT1,CD3D,LCK,ITK,TOX,PTPRC,PRKCH,FAS,ZAP70
Migration of effector memory T lymphocytes	2.10E-05	-1.63	ICOS,IFNG,ITGA4,ZAP70,MYH9,S1PR1
Activation of PBMCs	1.68E-02	-1.63	IL2,LY96,CLEC2D,IFNG,SOCS1,CD6
Inflammatory response of leukocytes	1.69E-02	-1.63	STAT3,F2R,IFNG,BRD3,ETS1,ERN1,BRD2
Formation of lymphoid tissue	8.23E-09	-1.63	NCOR2,IRF1,CBFB,ITGAL,CREB1,RASGRP4,RICTOR,IL2RB,RASGRP1,IL2RG,IKZF1,GATA3,DOCK8,SYK,CXCR4,CD3D,IL7R,LCK,ITK,NCOR1,LAT,ZAP70,CD3E,GRAP2,IL2,TRAF3IP3,NOTCH1,CD3G,CD5,IKBKB,PIM1,PDCD1,THEMIS,NCSTN,SIT1,CDKN1B,TRAT1,BCL2,SOCS1,PTPRC,BAX,ETS1
Formation of thymocytes	1.49E-09	-1.60	IRF1,CBFB,ITGAL,CREB1,RASGRP4,RICTOR,IL2RB,RASGRP1,IL2RG,IKZF1,GATA3,SYK,CXCR4,CD3D,IL7R,LCK,ITK,NCOR1,LAT,ZAP70,CD3E,GRAP2,IL2,TRAF3IP3,NOTCH1,CD3G,CD5,IKBKB,PIM1,PDCD1,THEMIS,SIT1,CDKN1B,TRAT1,BCL2,SOCS1,PTPRC,BAX,ETS1
Activation of cytotoxic T cells	2.33E-04	-1.57	MALT1,CCR2,CD40LG,IFNG,GZMA,PRF1,SMPD1,FUT8,VTI1B,FAS,CD247,ZAP70
Suppression of leukocytes	2.46E-05	-1.56	IL2,AKT3,IFNG,SMPD1,EP300,PIM1,CD6,PDCD1,GATA3,PARP1,STAT3,FKBP5,CBLB,DGKZ,TNFRSF18,TBX21
Cell movement of CD4+ T-lymphocytes	9.88E-03	-1.55	STAT3,CXCR4,BATF,ITK,NR1H2,LAG3,PDCD1,RAC2,AQP3
Inflammation of organ	2.33E-03	-1.53	NCOR2,IFNG,ZNF331,SELENOK,FCER1G,PDE4B,HSF2,STAT3,CXCR4,CD8A,HLA-DRB5,ITK,ISG20,NR4A2,PPP2CA,S1PR1,STAT4,RRN3,MBP,NOTCH1,STIM2,SMAD2,EP300,IKBKB,PRDM1,PDCD1,RABGAP1L,DUSP2,CD40LG,TNFAIP3,PHF1,BCL2,FAS,TBX21,AHNAK,JUNB
Cell death of macrophages	1.48E-02	-1.51	EIF2AK2,IRF1,MAPK14,IFNG,CDKN2A,STAT3,STING1,INPP5D,PIK3AP1,HMGB1,ISG15,CCL5,GPR132,DFFA,OGT,PYCARD,PARP1,SOAT1,TNFAIP3,BCL2,CAMK2G,CASP3,BCL2L1,FAS,CASP8,STK4
Quantity of follicular T helper cells	7.85E-03	-1.49	MALT1,IL2,STAT3,IFNG,ICOS,PRDM1,MAF,TIGIT,SH2D1A
Proliferation of activated T lymphocytes	6.77E-03	-1.49	IL2,SLAMF1,ITGAL,NOTCH1,PRKCQ,PRDM1,PYCARD,FYN,HMGB1,BTN3A1,CDKN1B,BCL2,STAT5A,TRAF1,STAT5B
Cell-cell contact	2.11E-02	-1.48	F11R,ITGAL,CCL5,ICOS,MAP3K14,CD44,CXCR4,SYK,CD40LG,ADGRE5,ITGA4,TNIP1,PTPRC
Proliferation of helper T lymphocytes	8.91E-03	-1.46	IL2,IFNG,ICOS,PTPN22,CD6,CD69,KLRD1,GATA3,PARP1,STAT3,CD40LG,IL7R,SOCS1,STAT5B
Response of bone marrow-derived macrophages	1.62E-02	-1.43	F2R,IFNG,BRD3,PRKAA1,PLCB3,SOCS1,ERN1,BRD2
Accumulation of leukocytes	1.49E-02	-1.41	IRF1,ITGAL,PAFAH1B1,PLAU,ICOS,PRKCQ,NCF1,CARD9,RASGRP1,IL2RG,DOCK8,STAT3,PIK3AP1,HMGB1,PNPLA2,LAT,S1PR1,IL2,CCL5,TLN1,IKBKB,SH2D2A,BAD,PPARD,BRAF,ADGRE5,ITGA4,FAS,STK4,FLT3LG,IFNG,TNFAIP8L2,IL2RB,ITGA1,INPP5D,TSC22D3,DEF6,STAT5A,CD27,KRAS,CCR2,ANGPT2,NOTCH1,CD99,CD44,MMP7,IP6K1,BCL2,MAPK13,RUNX3,TNFRSF18,CASP8
Cell survival of T lymphocytes	2.74E-05	-1.41	IL2,DEF6,PIM1,IL2RG,PYCARD
Differentiation of alpha-beta T lymphocytes	5.98E-03	-1.40	SYK,BRAF,NOTCH1,ITPKB,BCL11B,NKAP,ZAP70,ANXA1

Migration of peripheral T lymphocyte	4.18E-03	-1.39	CD47,CXCR4,RGS1,CCL5,PLCB3,LCP1
Hematologic cancer of cells	6.11E-03	-1.39	GSK3B,MS4A1,CD3E,PAG1,NOTCH1,BUB3,PIK3R1,PIM1,PDCD1,PARP1,BTG2,NRAS,CD40LG,HIPK3,BCL2,STAT5A,NIBAN1,FCMR,BCL2L1,STAT5B,FAS,CASP8
Movement of CD4+ T-lymphocytes	1.18E-02	-1.38	IFNG,CCL5,NR1H2,PDCD1,RAC2,AQP3,CD69,LCP1,STAT3,CXCR4,ITK,BATF,LAG3
Selection of thymocytes	2.86E-07	-1.37	CD3E,RHOH,TRAF3IP3,ATG5,PRKCQ,ELK4,ELK1,THEMIS,GATA3,SIT1,TRAT1,ITPKB,LCK,CD3D,ITK,TOX,PTPRC,PRKCH,FAS,ZAP70
Apoptosis of hematopoietic progenitor cells	3.24E-03	-1.35	IFNG,CDKN2A,H1-2,FYN,TSC22D3,LCK,PRKDC,TLE4,DUSP4,LTB,CYLD,CD3E,IL2,NOTCH1,RAP1GDS1,BCL11B,ATM,DFFA,IKBKB,BAD,ZC3H8,PARP1,MAP3K2,GZMA,BCL2,ARHGEF1,BIRC6,CASP3,PTPRC,BAX,BCL2L1,ETS1,FAS,CASP8,XKR8
Shape change of lymphocytes	1.51E-02	-1.35	SEMA4D,ITGAL,INPP5D,ICOS,SMPD1,RAP1B,FYB1,CD44,MYH9
Survival of CD4+ T-lymphocytes	2.25E-03	-1.34	PIM1,CASP8,IL2RG,GIMAP1,PYCARD
Expression of protein	8.99E-03	-1.34	IL2,SYK,IFNG,LCK,PRKAA1,FAS
Positive selection of thymocytes	1.05E-07	-1.32	RHOH,TRAF3IP3,PRKCQ,ELK4,ELK1,THEMIS,SIT1,TRAT1,LCK,ITK,TOX,PRKCH,PTPRC
Cytotoxic T lymphocyte response	3.62E-05	-1.31	IL2,PARP1,CD8A,IFNG,BAX,SH2D1A,HSDL1,PYCARD
Inhibition of mononuclear leukocytes	4.24E-06	-1.31	IL2,AKT3,IFNG,KLRB1,EP300,SMPD1,PTGER4,CD6,PIM1,PDCD1,GATA3,PARP1,HLA-A,CLEC2D,ITGA4,CBLB,DGKZ,TNFRSF18,TBX21
Activation of B lymphocytes	2.76E-03	-1.30	SEMA4D,FLT3LG,TNFAIP8L2,IFNG,PLEKHA2,IL2RB,TAB2,RASGRP1,MALT1,SYK,STING1,INPP5D,CLEC2D,PPP2R3C,TXLNA,PRKCB,ZAP70,MS4A1,SH3KBP1,IL2,LAX1,PDCD1,CD40LG,TNFAIP3,FAS,CASP8,TBC1D10C
Differentiation of Th2 cells	2.28E-03	-1.28	IRF1,IL2,MAPK14,STAT4,IFNG,CCL5,ICOS,PRKCQ,RICTOR,SOCS2,GATA3,CDKN1B,STAT5A,ITK,BATF,CBLB,SH2D1A,PGAP3,TBX21
Transcription of RNA	3.61E-03	-1.28	IL2,INPP5D,IFNG,MAP3K14,GTF2I,IKBKB,CREBBP,MAF,ZAP70
Inhibition of T lymphocytes	7.63E-05	-1.26	IL2,AKT3,SMPD1,EP300,PTGER4,PIM1,CD6,PDCD1,GATA3,PARP1,HLA-A,CBLB,DGKZ,TNFRSF18,TBX21
Interaction of CD4+ T-lymphocytes	6.68E-03	-1.25	CXCR4,IL7R,FYB1,ANXA1,SKAP1
Emigration of T lymphocytes	2.56E-02	-1.21	CXCR4,IFNG,STK4,S1PR1
Quantity of intraepithelial T lymphocytes	8.59E-05	-1.20	IRF1,LCK,IL7R,PTPRC,TAP1,IL2RG,IKZF1
Maturation of natural killer T lymphocytes	1.03E-02	-1.19	ELF1,IL7R,ITK,DGKZ,TBX21
Differentiation of regulatory T lymphocytes	2.54E-02	-1.18	DUSP10,CCR2,IL2,MAPK14,ICOS,SMAD2,PRKCQ,CD46,MAF,CD69,STAT3,ITK,SOCS1,NCOR1,TNFRSF18,S1PR1
Immune response of T lymphocytes	3.54E-03	-1.16	FLT3LG,IFNG,ICOS,HSDL1,MALT1,DOCK8,STAT3,IL7R,ITK,CD27,IL2,IKBKB,TIGIT,PDCD1,TAPBP,CD69,PYCARD,PARP1,CDKN1B,BAX,BCL2L1,ETS1,SH2D1A,PGAP3,TBX21,CASP8
Mitogenesis of lymphocytes	1.03E-02	-1.15	STAT3,STAT4,IL7R,CDKN2A,IKBKB
Exposure of phosphatidylserine	2.56E-02	-1.15	HMGB1,ANO6,FAS,XKR8
Nephritis	4.25E-02	-1.13	NCOR2,CD40LG,CD8A,IFNG,BCL2,EP300,FAS,RABGAP1L,PPP2CA,ISG20
Glomerulonephritis	4.92E-02	-1.13	NCOR2,CD40LG,IFNG,BCL2,EP300,FAS,RABGAP1L,ISG20,PPP2CA
Expansion of B lymphocytes	8.60E-04	-1.13	SIGIRR,CCND2,MYBL1,LY9,LCP1,CCND3,TNFAIP3,BCL2,STAT5A,IL7R,BCL2L1,STAT5B,LAG3,S1PR1

Quantity of plasma cells	1.58E-02	-1.11	FLT3LG,IL2,ANGPT2,IFNG,ICOS,IL2RB,PRDM1,PDCD1,RAC2,RASGRP1,IL2RG,RGS1,IL7R,PRKCB,FCMR,ETS1
Cell spreading of lymphocytes	1.31E-02	-1.09	ITGAL,INPP5D,ICOS,SMPD1,RAP1B,FYB1,CD44
Accumulation of dendritic cells	1.25E-02	-1.09	FLT3LG,DOCK8,CCR2,DEF6,CD99,RUNX3
Degranulation of leukocytes	3.66E-03	-1.08	FTL,RASGRP4,FCER1G,RASGRP1,STK10,STING1,HMGB1,LAT,LAG3,A1BG,QSOX1,AGPAT2,IL2,LAX1,CD47,AGA,NRAS,SVIP,ARMC8,COPB1,RAP1B,STAT5B,ETS1,CYBA,SPTAN1,JUNB,NBEAL2,PTPN22,WIPF1,RAB6A,B4GALT1,PTPN4,FYN,HUWE1,HSPA8,STAT5A,DGKZ,GSTP1,ATP8A1,PYGL,CD58,GYG1,PYCARD,VTI1B,RAB27A,ORMDL3,IQGAP1,ITGAL,PLAU,HSPA6,ARHGAP45,DDX3X,LPIN1,SYK,CD63,ANO6,GPI,RAB37,DYNC1H1,GHDC,PRF1,MGST1,TMEM30A,ANXA1,DNAJC3,RAB5B,ADGRE5,PSMD1,MAPK14,CAB39,VAMP2,KLRB1,TSPAN14,RAC2,TMC6,UBR4,INPP5D,CPNE1,ITK,SLAMF6,PSMD2,HSP90AA1,PTGDR,PAFAH1B2,PAK2,SERPINB6,AP1M1,PDCD1,CD44,NCR3,NCSTN,BST2,TNFAIP3,ACLY,PTPRC,CAND1,TBC1D10C
Accumulation of mononuclear leukocytes	8.22E-04	-1.08	ITGAL,TNFAIP8L2,IFNG,ICOS,PRKCQ,IL2RB,CARD9,RASGRP1,IL2RG,ITGA1,STAT3,TSC22D3,DEF6,PIK3AP1,STAT5A,LAT,CD27,S1PR1,KRAS,CCR2,IL2,CCL5,NOTCH1,TLN1,CD99,IKBKB,SH2D2A,BAD,PPARD,BRAF,BCL2,RUNX3,TNFRSF18,FAS,CASP8,STK4
Killing of leukocytes	1.40E-03	-1.08	IL2,SLAMF1,MAPK14,IFNG,PRF1,LY9,CD47,HLA-A,SLAMF6,BAX,RAB27A,FAS,SH2D1A
Egression of T lymphocytes	1.70E-02	-1.07	CCR2,ITGAL,RAC2,S1PR1
Glycolysis	7.89E-03	-1.06	IL2,TRAF3IP3,CREM,CRBN,IFNG,PLEKHA2,EPAS1,PIM1,GSTP1,PIK3R1,PDCD1,ANXA1
Differentiation of CD4+ T-lymphocytes	3.66E-03	-1.03	MAPK14,IFNG,ICOS,PRKCQ,NDFIP1,CARD9,CD6,MALT1,STAT3,CD8A,DEF6,STAT5A,ITK,TOX,NR4A2,CCR2,IL2,STAT4,DNAJB1,SMAD2,MAP3K14,SOCS2,MAF,PRDM1,CD69,RORA,SH2D2A,USP15,BATF,TNFRSF18,ETS1,TBX21
Activation of peripheral blood lymphocytes	1.70E-02	-1.00	IL2,CLEC2D,SOCS1,CD6
Inflammatory response of bone marrow-derived macrophages	2.56E-02	-1.00	F2R,BRD3,ERN1,BRD2
Activation-induced cell death of T lymphocytes	2.12E-05	-0.99	IL2,SLC6A6,IFNG,CREB1,CD5,PRF1,RAC2,CD44,DNAJA3,TSC22D3,MAP3K2,BCL2,ITK,CASP3,BCL2L1,FAS,CASP8
Lymphocytic cancer	6.16E-03	-0.97	GSK3B,PIK3R1,BTG2,HIPK3,STAT5A,NIBAN1,CD27,MS4A1,CD3E,PAG1,NOTCH1,BUB3,PRF1,PIM1,PDCD1,CD47,PARP1,NRAS,CD40LG,BCL2,CASP3,FCMR,BCL2L1,STAT5B,FAS,CASP8
Suppression of T lymphocytes	3.72E-04	-0.95	IL2,AKT3,SMPD1,EP300,PIM1,CD6,PDCD1,GATA3,PARP1,CBLB,DGKZ,TNFRSF18,TBX21
Expansion of hematopoietic progenitor cells	5.98E-03	-0.94	SYK,ZBTB16,TOX,BCL2L1,LY9,ZAP70,S1PR1,IKZF1
Activation of CD4+ T-lymphocytes	1.54E-03	-0.94	IL2,IFNG,TLN1,ATG5,CD46,CAMK4,NDFIP1,PTPN22,CD6,PRDM1,STAT3,HLA-A,PRKCB,AZI2,BCLAF1,DGKZ,CD27,CASP8
Killing of lymphocytes	7.54E-03	-0.93	CD47,IL2,SLAMF1,HLA-A,PRF1,SLAMF6,RAB27A,LY9,FAS,SH2D1A
Expression of RNA	1.33E-03	-0.90	CD47,IL2,INPP5D,IFNG,BATF,MAP3K14,GTF2I,IKBKB,CREBBP,MAF,ZAP70
Accumulation of lymphocytes	1.23E-03	-0.89	ITGAL,TNFAIP8L2,IFNG,ICOS,PRKCQ,IL2RB,CARD9,RASGRP1,IL2RG,STAT3,TSC22D3,DEF6,STAT5A,LAT,CD27,S1PR1,KRAS,IL2,CCL5,NOTCH1,TLN1,CD99,IKBKB,SH2D2A,BAD,PPARD,BRAF,BCL2,RUNX3,TNFRSF18,FAS,CASP8,STK4
Maturation of invariant natural killer T cells	1.04E-02	-0.88	ELF1,IL7R,ITK,DGKZ
Survival of CD8+ T lymphocyte	2.25E-03	-0.88	IL7R,FAS,CASP8,PIK3C3,GIMAP1
Function of leukocytes	7.14E-08	-0.87	IRF1,AKAP13,ICOS,CBX5,RASGRP4,FCER1G,IL16,RASGRP1,IL2RG,STK10,LAT,LAG3,S1PR1,IL2,TXNIP,DLG1,LAX1,PTGER4,DUSP2,TAPBP,PTGER2,AZI2,BCL2L1,FCMR,STAT5B,NTAN1,ETS1,STK4,FLT3LG,SLAMF1,CREB1,IFNG,DOK2,PTPN22,WIPF1,TAP1,B4GALT1,LCP1,FYN,STAT5A,DGKZ,SLA2,CD27,CCR2,STAT4,CD3G,FYB1,ABCC1,PYCARD,PARP1,HLAA,CD40LG,GZMA,BCL2,ARHGEF1,PGAP3,TBX21,AHNAK,ITGAL,PLAU,PRKCQ,CARD9,MCPH1,MALT1,DOCK8,STAT3,ARHGEF6,CD8A,CBLB,DUSP4,PIK3C3,IL18R1,CYLD,CD3E,CCL5,PRF1,SMPD1,PIM1,PSTPIP1,HCST,CD69,BAD,GSK3B,GIMAP4,F11R,TNFAIP8L2,KLRB1,IL13RA1,SELENOK,PIK3R1,LY96,INPP5D,ITK,SLAMF6,N4BP1,PRKDC,HSP90AA1,PTGDR,GADD45G,SIGIRR,CD8B,TAX1BP1,TNFSF14,ATM,PPP3CB,SLA,PDCD1,CD44,CD96,MMP7,RGS1,SOCS1,PTPRC,CD247,SH2D1A,CASP8
Hematologic cancer	7.01E-03	-0.86	GSK3B,CDKN2A,PIK3R1,STAT3,BTG2,HIPK3,STAT5A,NIBAN1,CD27,MS4A1,CD3E,PAG1,NOTCH1,BUB3,PRF1,PIM1,PDCD1,CD47,PARP1,NRAS,CD40LG,BCL2,CASP3,FCMR,BCL2L1,STAT5B,FAS,CASP8
Accumulation of T lymphocytes	2.43E-04	-0.85	ITGAL,IFNG,ICOS,PRKCQ,IL2RB,CARD9,RASGRP1,IL2RG,STAT3,DEF6,STAT5A,LAT,CD27,S1PR1,KRAS,IL2,CCL5,NOTCH1,CD99,IKBKB,SH2D2A,PPARD,BRAF,BCL2,RUNX3,FAS,CASP8,STK4
Synthesis of carbohydrate	8.99E-03	-0.85	INPP5D,PCYT1A,PTPRC,PLA2G6,PIK3R1,ANXA1
Synthesis of phosphatidic acid	1.51E-02	-0.85	INPP5D,PCYT1A,PLA2G6,PIK3R1,ANXA1
Cell death of hematopoietic progenitor cells	5.52E-03	-0.84	IFNG,CDKN2A,H1-2,FYN,TSC22D3,LCK,PRKDC,TLE4,DUSP4,LTB,CYLD,CD3E,IL2,NOTCH1,RAP1GDS1,BCL11B,ATM,MAP3K14,DFFA,IKBKB,BAD,ZC3H8,PARP1,DNAJA3,MAP3K2,GZMA,ARHGEF1,BCL2,BIRC6,CASP3,PTPRC,BCL2L1,BAX,ETS1,FAS,CASP8,XKR8

Cell cycle progression of T lymphocytes	2.75E-04	-0.83	KRAS,IL2,ITGAL,ATG5,ATM,CDKN2A,CDKN1B,BCL2,STAT5A,BCL2L1,STAT5B,LAG3,SKIL
Glycolysis of cells	1.04E-02	-0.82	IL2,TRAF3IP3,CREM,CRBN,IFNG,PLEKHA2,EPAS1,PIM1,GSTP1,PDCD1,ANXA1
Cell viability of mast cells	1.62E-02	-0.79	GSK3B,INPP5D,LAX1,LAT,FCER1G,PIK3R1,YWHAZ,RAC2
Killing of cells	5.15E-03	-0.79	IL2,SLAMF1,MAPK14,IFNG,PRF1,LY9,CARD9,CD47,LY96,HLA-A,PRKCB,SLAMF6,RAB27A,BAX,FAS,SH2D1A
Cancer of cells	8.39E-03	-0.73	GSK3B,PIK3R1,BTG2,HIPK3,STAT5A,NIBAN1,CD27,MS4A1,CD3E,PAG1,NOTCH1,BUB3,PRF1,PIM1,PDCD1,CD47,PARP1,NRAS,CD40LG,BCL2,CASP3,FCMR,BCL2L1,STAT5B,FAS,CASP8
Apoptosis of thymocytes	2.01E-03	-0.71	CDKN2A,H1-2,FYN,LCK,PRKDC,TLE4,LTB,CYLD,CD3E,IL2,NOTCH1,RAP1GDS1,BCL11B,ATM,DFFA,IKBKB,ZC3H8,PARP1,MAP3K2,GZMA,ARHGEF1,BCL2,BIRC6,CASP3,PTPRC,BAX,BCL2L1,ETS1,FAS,CASP8,XKR8
Selection of lymphocytes	4.84E-08	-0.70	RHOH,CD3E,TRAF3IP3,ATG5,PRKCQ,ELK4,TAPBP,ELK1,THEMIS,GATA3,ELF1,SYK,SIT1,ITPKB,TRAT1,CD3D,LCK,ITK,TOX,PTPRC,BAX,PRKCH,FAS,ZAP70
Expansion of thymocytes	8.99E-03	-0.64	SYK,ZBTB16,TOX,LY9,ZAP70,IKZF1
Solid tumor	1.02E-03	-0.64	IFITM1,GNB1,EIF4A2,PIK3R1,ATP6AP1,LCP1,SNRNP200,BTG2,HIPK3,STAT5A,LCK,SRRM1,NIBAN1,DDX5,NONO,CD3E,PAG1,BUB3,SRSF2,EP300,PIM1,ANXA1,CALCOCO2,PARP1,NRAS,CD40LG,PTPRC,STAT5B,CASP8
Function of TREG cells	2.56E-02	-0.63	DLG1,LAT,TBX21,S1PR1
Lymphatic system tumor	1.15E-03	-0.57	GSK3B,CDKN2A,PIK3R1,BTG2,INPP5D,HIPK3,STAT5A,IL7R,LCK,NIBAN1,CD27,MS4A1,CD3E,PAG1,NOTCH1,BUB3,PRF1,PIM1,PDCD1,CD47,PARP1,NRAS,CD40LG,BCL2,CASP3,FCMR,BCL2L1,STAT5B,FAS,CASP8
Lymphocytic neoplasm	1.31E-03	-0.57	GSK3B,CDKN2A,PIK3R1,BTG2,INPP5D,HIPK3,STAT5A,IL7R,NIBAN1,CD27,MS4A1,CD3E,PAG1,NOTCH1,BUB3,PRF1,PIM1,PDCD1,CD47,PARP1,NRAS,CD40LG,BCL2,CASP3,FCMR,BCL2L1,STAT5B,FAS,CASP8
Myeloid or lymphoid neoplasm	2.01E-03	-0.57	GSK3B,CDKN2A,PIK3R1,CD63,STAT3,BTG2,INPP5D,HIPK3,STAT5A,IL7R,NIBAN1,CD27,MS4A1,CD3E,PAG1,NOTCH1,BUB3,PRF1,PIM1,PDCD1,CD47,PARP1,NRAS,CD40LG,BCL2,CASP3,FCMR,BCL2L1,STAT5B,FAS,CASP8
Neoplasia of leukocytes	2.06E-03	-0.57	GSK3B,CDKN2A,PIK3R1,CD63,BTG2,INPP5D,HIPK3,STAT5A,IL7R,NIBAN1,CD27,MS4A1,CD3E,PAG1,NOTCH1,BUB3,PRF1,PIM1,PDCD1,CD47,PARP1,NRAS,CD40LG,BCL2,CASP3,FCMR,BCL2L1,STAT5B,FAS,CASP8
Malignant solid tumor	2.76E-03	-0.57	IFITM1,GNB1,EIF4A2,PIK3R1,ATP6AP1,LCP1,SNRNP200,BTG2,HIPK3,STAT5A,SRRM1,NIBAN1,DDX5,NONO,CD3E,PAG1,BUB3,SRSF2,EP300,PIM1,CALCOCO2,PARP1,NRAS,CD40LG,PTPRC,STAT5B,CASP8
Neoplasia of blood cells	3.09E-03	-0.57	GSK3B,CDKN2A,PIK3R1,CD63,STAT3,BTG2,INPP5D,HIPK3,STAT5A,IL7R,NIBAN1,CD27,MS4A1,CD3E,PAG1,NOTCH1,BUB3,PRF1,PIM1,PDCD1,CD47,PARP1,NRAS,CD40LG,BCL2,CASP3,FCMR,BCL2L1,STAT5B,FAS,CASP8
Proliferation of naive T lymphocytes	9.94E-04	-0.55	CD3E,IL2,STAT3,CD40LG,DEF6,CDKN1B,TNFSF14,SOCS1,IKBKB,PIM1,CD69
Expansion of natural killer cells	1.51E-02	-0.53	IRF1,IL2,IL2RB,SOCS1,PTPRC
Hematological or lymphatic system tumor	2.43E-03	-0.51	GSK3B,CDKN2A,PIK3R1,CD63,STAT3,BTG2,INPP5D,HIPK3,STAT5A,IL7R,LCK,NIBAN1,CD27,MS4A1,CD3E,PAG1,NOTCH1,BUB3,PRF1,PIM1,PDCD1,CD47,PARP1,NRAS,CD40LG,BCL2,CASP3,FCMR,BCL2L1,STAT5B,FAS,CASP8
Quantity of naive lymphocytes	1.71E-03	-0.49	DOCK8,IL2,ITGAL,ICOS,CDKN1B,ITK,IKZF3
Tumorigenesis of lymphocytes	2.17E-03	-0.48	GSK3B,CDKN2A,PIK3R1,BTG2,INPP5D,HIPK3,STAT5A,IL7R,NIBAN1,CD27,MS4A1,CD3E,PAG1,BUB3,PRF1,PIM1,PDCD1,CD47,PARP1,NRAS,CD40LG,BCL2,CASP3,FCMR,BCL2L1,STAT5B,CASP8
Cancer	1.07E-03	-0.45	GSK3B,IFITM1,GNB1,CDKN2A,EIF4A2,PIK3R1,ATP6AP1,LCP1,SNRNP200,STAT3,BTG2,HIPK3,STAT5A,SRRM1,NIBAN1,DDX5,CD27,NONO,MS4A1,CD3E,PAG1,NOTCH1,BUB3,SRSF2,PRF1,EP300,PIM1,PDCD1,CD47,CALCOCO2,PARP1,NRAS,CD40LG,BCL2,CASP3,PTPRC,BCL2L1,FCMR,STAT5B,FAS,CASP8
Hyperplasia of hematopoietic progenitor cells	1.70E-02	-0.45	MAP2K7,PIK3R1,RAC2,IKZF1
Transmembrane potential of mitochondria	1.68E-02	-0.45	CD47,MAPK14,BCL2,BCL2L1,FAS,CD2
Positive selection of lymphocytes	3.70E-09	-0.42	RHOH,TRAF3IP3,PRKCQ,ELK4,ELK1,THEMIS,SYK,SIT1,TRAT1,LCK,ITK,TOX,PRKCH,PTPRC,ZAP70
Degranulation of natural killer cells	2.18E-03	-0.41	IL2,TNFAIP3,KLRB1,PRF1,SLAMF6,RAB27A,PTPRC,DGKZ,ETS1,NCR3

Anergy of lymphocytes	2.14E-02	-0.39	CD3E,IL2,PAG1,INPP5D,CD5,PRKCQ,CBLB
Extrapancreatic malignant tumor	8.46E-04	-0.37	GSK3B,IFITM1,GNB1,CDKN2A,EIF4A2,PIK3R1,ATP6AP1,LCP1,SNRNP200,STAT3,BTG2,HIPK3,STAT5A,SRRM1,NIBAN1,DDX5,CD27,NONO,MS4A1,CD3E,PAG1,NOTCH1,BUB3,SRSF2,PRF1,PIM1,PDCD1,CD47,CALCOCO2,PARP1,NRAS,CD40LG,BCL2,CASP3,FCMR,BCL2L1,STAT5B,FAS,CASP8
Apoptosis of effector T lymphocytes	3.50E-05	-0.34	IL2,TLN1,IFNG,CDKN1B,PTPN22,FAS,PDCD1
Cell-mediated response	6.29E-04	-0.30	FLT3LG,IL2,IFNG,ICOS,PRKAA1,TIGIT,PDCD1,PYCARD,HSDL1,MALT1,PARP1,STAT3,CD8A,CDKN1B,IL7R,ITK,BAX,SH2D1A,PGAP3,CASP8
Cell death of cytotoxic T cells	4.05E-03	-0.28	IL2,GZMA,PRKAA1,PRF1,FAS
Conversion of T lymphocytes	2.21E-02	-0.28	IL2,PARP1,STAT4,TNFAIP3,IFNG,BCL11B
Accumulation of B lymphocytes	2.21E-02	-0.27	TLN1,DEF6,TSC22D3,ICOS,BCL2,CD99,IKBKB,FAS,BAD
Cell death of thymocytes	2.27E-03	-0.26	CDKN2A,H1-2,FYN,LCK,PRKDC,TLE4,LTB,CYLD,CD3E,IL2,NOTCH1,RAP1GDS1,BCL11B,ATM,MAP3K14,DFFA,IKBKB,BAD,ZC3H8,PARP1,DNAJA3,MAP3K2,GZMA,ARHGEF1,BCL2,BIRC6,CASP3,PTPRC,BAX,BCL2L1,ETS1,FAS,CASP8,XKR8
Survival of organism	8.63E-03	-0.22	NRAS,TNFAIP3,ATG5,IFNG,BCL2,STAT5A,IL7R,NCF1,BCL2L1,STAT5B,CASP8
Suppression of TREG cells	4.48E-04	-0.20	GATA3,IL2,PARP1,SMPD1,EP300,DGKZ,CD6,PIM1,PDCD1
Proliferation of double-negative T lymphocyte	4.34E-04	-0.16	IL2,TNFAIP3,CDKN1B,MAP3K14,RICTOR,HNRNPL,RASGRP1,LTB
Proliferation of granulocytes	1.02E-02	-0.14	STAT3,MAPK14,ATG5,STAT5A,PTPRC,STAT5B,LAG3,SENP1
Quantity of naive T lymphocytes	1.51E-02	-0.13	DOCK8,IL2,ITGAL,CDKN1B,ITK
Quantity of germinal center B lymphocytes	4.11E-03	-0.11	IL2,ICOS,MAP3K14,PRDM1,MALT1,STAT3,CD40LG,ARHGEF1,ITK,FCMR,FAS,N4BP1,SH2D1A
Function of lymphocytes	5.38E-09	-0.10	IRF1,ITGAL,AKAP13,CBX5,ICOS,PRKCQ,FCER1G,IL16,IL2RG,STK10,MCPH1,ARHGEF6,CD8A,CBLB,LAT,LAG3,PIK3C3,IL18R1,S1PR1,CD3E,IL2,TXNIP,DLG1,PRF1,LAX1,HCST,PSTPIP1,TAPBP,CD69,BAD,BCL2L1,NTAN1,STAT5B,ETS1,STK4,GSK3B,FLT3LG,GIMAP4,SLAMF1,IFNG,CREB1,TNFAIP8L2,KLRB1,IL13RA1,SELENOK,PTPN22,WIPF1,PIK3R1,TAP1,FYN,INPP5D,STAT5A,ITK,SLAMF6,PRKDC,CD27,GADD45G,CCR2,STAT4,CD8B,TAX1BP1,CD3G,TNFSF14,ATM,PPP3CB,FYB1,PDCD1,CD44,CD96,HLA-A,CD40LG,ARHGEF1,BCL2,SOCS1,PTPRC,CD247,SH2D1A,PGAP3,TBX21,AHNAK
Extracranial solid tumor	3.92E-03	-0.09	PAG1,IFITM1,SRSF2,EP300,GNB1,EIF4A2,PIK3R1,ATP6AP1,LCP1,ANXA1,SNRNP200,CALCOCO2,LCK,SRRM1,PTPRC,DDX5,NONO
Apoptosis of naive T lymphocytes	1.01E-03	-0.07	IL2,PRF1,FAS,IL2RG
Lymphoproliferative disorder	1.29E-03	0.00	GSK3B,CDKN2A,PIK3R1,BTG2,INPP5D,HIPK3,STAT5A,IL7R,NIBAN1,LAT,CD27,MS4A1,CD3E,PAG1,NOTCH1,BUB3,PRF1,PIM1,PDCD1,CD47,PARP1,NRAS,CD40LG,BCL2,CASP3,FCMR,BCL2L1,STAT5B,FAS,CASP8
Suppression of regulatory T lymphocytes	6.12E-04	0.10	GATA3,IL2,PARP1,SMPD1,EP300,DGKZ,TNFRSF18,PIM1,CD6,PDCD1
Quantity of effector memory T lymphocytes	1.31E-02	0.11	MALT1,FLT3LG,ITGAL,ANGPT2,ICOS,SOCS1,SERPINB6
Apoptosis of CD4+ T-lymphocytes	9.99E-03	0.11	IL2,IFNG,CD5,PRKCQ,FAS,CD6,GIMAP1
Apoptosis	1.15E-08	0.11	IRF1,ICOS,CDKN2A,FCER1G,IL2RG,CXCR4,STING1,HMGB1,IL7R,LCK,PRKCB,TRAF1,TLE4,SKIL,LTB,S1PR1,IL2,TLN1,EZR,PTGER4,ZC3H8,CD47,DNAJA3,CAMK2G,BIRC6,FCMR,BAX,BCL2L1,STAT5B,FAS,ETS1,FLT3LG,RHOH,SLC6A6,CREB1,IFNG,FOXP1,ZBTB16,PTPN22,H12,FYN,STAT5A,ISG15,CD27,WWP2,CASP10,NOTCH1,GPR132,RAP1GDS1,BCL11B,CD5,EP300,OGT,KLRC1,GIMAP1,PARP1,CD40LG,SOAT1,GZMA,BCL2,ARHGEF1,ORMDL3,TNFRSF18,XCL1,CD2,ANKZF1,EIF2AK2,ATG5,PRKCQ,MALT1,DOCK8,SYK,STAT3,NOC2L,PIK3AP1,DUSP4,PIK3C3,CYLD,GRAP2,CD3E,CCL5,SMPD1,PRF1,IKBKB,PIM1,BAD,ANXA1,CDKN1B,CASP3,STK17B,NCAPH2,GSK3B,MAPK14,TNFAIP8L2,PIK3R1,CD6,RAC2,INPP5D,TSC22D3,ITK,PRKDC,GADD45G,KRAS,ATM,DFFA,PPP3CB,PDCD1,CD44,CASP6,TNFAIP3,MAP3K2,SOCS1,PPP2R2B,PTPRC,CD247,CASP8,XKR8
Conversion of leukocytes	1.31E-02	0.13	IL2,PARP1,STAT4,TNFAIP3,BACH1,IFNG,BCL11B
Apoptosis of leukocytes	1.46E-08	0.19	IRF1,ICOS,CDKN2A,FCER1G,IL2RG,CXCR4,STING1,HMGB1,IL7R,LCK,PRKCB,TRAF1,TLE4,SKIL,LTB,S1PR1,IL2,TLN1,EZR,PTGER4,ZC3H8,CD47,DNAJA3,CAMK2G,BIRC6,BCL2L1,BAX,FCMR,STAT5B,FAS,ETS1,FLT3LG,RHOH,SLC6A6,CREB1,IFNG,FOXP1,ZBTB16,PTPN22,H12,FYN,STAT5A,ISG15,WWP2,CD27,CASP10,NOTCH1,GPR132,RAP1GDS1,BCL11B,CD5,EP300,OGT,KLRC1,GIMAP1,PARP1,CD40LG,SOAT1,GZMA,BCL2,ARHGEF1,TNFRSF18,ORMDL3,XCL1,CD2,ANKZF1,EIF2AK2,ATG5,PRKCQ,MALT1,DOCK8,SYK,STAT3,NOC2L,PIK3AP1,DUSP4,PIK3C3,CYLD,GRAP2,CD3E,CCL5,PRF1,SMPD1,IKBKB,PIM1,BAD,ANXA1,CDKN1B,CASP3,STK17B,GSK3B,MAPK14,TNFAIP8L2,PIK3R1,CD6,RAC2,INPP5D,TSC22D3,ITK,PRKDC,GADD45G,KRAS,ATM,DFFA,PPP3CB,PDCD1,CD44,CASP6,TNFAIP3,MAP3K2,SOCS1,PPP2R2B,PTPRC,CD247,CASP8,XKR8
Quantity of central memory T lymphocyte	1.04E-02	0.19	MALT1,ITGAL,CD40LG,ICOS
Cell-mediated response of T lymphocytes	2.89E-03	0.21	FLT3LG,IFNG,ICOS,TIGIT,PDCD1,PYCARD,HSDL1,MALT1,PARP1,STAT3,CDKN1B,IL7R,ITK,PGAP3,CASP8

Killing of antigen presenting cells	7.43E-03	0.22	MAPK14,SLAMF1,IFNG,SLAMF6,BAX,LY9,SH2D1A
Killing of phagocytes	7.43E-03	0.22	MAPK14,SLAMF1,IFNG,SLAMF6,BAX,LY9,SH2D1A
Renal cancer	1.33E-03	0.30	SNRNP200,CALCOCO2,IFITM1,SRSF2,SRRM1,GNB1,DDX5,EIF4A2,ATP6AP1,NONO,LCP1
Cellular infiltration by leukocytes	2.45E-02	0.34	CBFB,MAPK14,IFNG,CCL5,ICOS,BCL11B,NR1H2,PIK3R1,PRDM1,IL16,PDCD1,CD44,CD47,STAT3,BRAF,LAG3,TBX21
Function of T lymphocytes	4.55E-08	0.38	ITGAL,CBX5,ICOS,PRKCQ,FCER1G,IL16,STK10,MCPH1,ARHGEF6,CD8A,CBLB,LAT,LAG3,PIK3C3,IL18R1,S1PR1,CD3E,IL2,DLG1,PRF1,LAX1,HCST,PSTPIP1,TAPBP,CD69,BAD,BCL2L1,NTAN1,STAT5B,STK4,GSK3B,FLT3LG,GIMAP4,SLAMF1,CREB1,IFNG,TNFAIP8L2,IL13RA1,SELENOK,PTPN22,WIPF1,FYN,INPP5D,STAT5A,ITK,SLAMF6,PRKDC,CD27,GADD45G,CCR2,STAT4,CD8B,TAX1BP1,CD3G,TNFSF14,ATM,PPP3CB,FYB1,PDCD1,CD44,HLA-A,CD40LG,BCL2,PTPRC,CD247,SH2D1A,PGAP3,TBX21,AHNAK
Quantity of gamma-delta T lymphocytes	1.83E-03	0.38	RHOH,IL2,ITGAL,RICTOR,IKZF1,GPR18,SIT1,CD8A,CDKN1B,BCL2,IL7R,LCK,ITK,SOCS1,TNIP1,PTPRC
Differentiation of TREG cells	2.21E-02	0.38	CCR2,IL2,MAPK14,ITK,CD69,S1PR1
Cell death of effector T lymphocytes	2.73E-06	0.44	IL2,TLN1,IFNG,CDKN1B,PRKAA1,SMPD1,PTPN22,FAS,PDCD1
Quantity of memory T lymphocytes	5.89E-05	0.53	FLT3LG,IL2,GRAP2,ITGAL,ANGPT2,ICOS,IL2RB,PTPN22,SERPINB6,PIK3R1,IRF2,THEMIS,MALT1,CD40LG,ITK,SOCS1,BCL2L1,PRKCH,ETS1,CD27,STK17B,TBX21,LTB
Exhaustion of T lymphocytes	8.59E-05	0.54	DOCK8,IFNG,ANO6,IL2RB,TNFRSF18,CD27,PDCD1
Quantity of effector T lymphocytes	4.75E-03	0.55	MALT1,FLT3LG,IL2,ITGAL,ANGPT2,ICOS,CDKN1B,SOCS1,SERPINB6
Suppressive capacity of TREG cells	6.39E-04	0.57	PARP1,SMPD1,EP300,DGKZ,CD6,PIM1,PDCD1
Quantity of double-negative T lymphocyte	1.38E-06	0.57	FLT3LG,RHOH,PRKCQ,TAP1,IL2RG,CCND3,IL7R,LCK,ITK,LAT,PRKCH,LAG3,ASXL1,PRKDC,GRAP2,IL2,CD3E,BCL11B,ATM,JAK1,PPARD,KAT6A,BCL2,PTPRC,KMT2E,ETS1,FAS,CD247
Cell death of PBMCs	2.14E-02	0.58	GIMAP4,CD40LG,EZR,PTPRC,FAS,ZAP70,GIMAP1
Metabolism of DNA	2.60E-03	0.59	IL2,PARP1,IFNG,CAMK2G,DFFA,CASP3,FAS
Fragmentation of DNA	2.66E-03	0.59	IL2,PARP1,IFNG,DFFA,CASP3,FAS
Quantity of helper T lymphocytes	2.25E-03	0.64	IRF1,IFNG,ICOS,TOB1,PRKCQ,IL13RA1,NDFIP1,TAP1,RASGRP1,GATA3,MALT1,STAT3,PIK3AP1,LCK,CCR2,IL2,CCL5,IKBKB,TIGIT,SERPINB6,PRDM1,MAF,CD69,PYCARD,BATF,TNIP1,TNFRSF18,SH2D1A,TBX21,ATXN1,XKR8
Cell death of effector memory T lymphocytes	1.11E-03	0.64	IL2,IFNG,PRKAA1,SMPD1,FAS
Hyperplasia of leukocytes	4.98E-03	0.65	CYLD,MAP2K7,MAPK14,MAP3K14,CDKN2A,PIK3R1,RAC2,IKZF1,CD40LG,BAX,LAT,TNFRSF18,DUSP4
Organismal death	6.77E-03	0.68	CD3E,IFNG,MSN,EP300,GNB1,CDKN2A,PRDM1,ATP6AP1,LCP1,TNFAIP3,SOCS1,EIF4A1,DDX5,CASP8,BRD2
Activation of memory T lymphocytes	4.18E-03	0.71	MALT1,IL2,HLA-A,CDKN1B,MGST1,ZAP70
Apoptosis of T lymphocytes	4.28E-13	0.73	ATG5,ICOS,PRKCQ,CDKN2A,IL2RG,DOCK8,STING1,STAT3,CXCR4,IL7R,LCK,TRAF1,TLE4,PIK3C3,LTB,CYLD,GRAP2,CD3E,IL2,CCL5,TLN1,EZR,PRF1,IKBKB,ZC3H8,CD47,DNAJA3,CDKN1B,BIRC6,CASP3,BCL2L1,BAX,FCMR,STAT5B,ETS1,FAS,STK17B,GSK3B,RHOH,SLC6A6,IFNG,CREB1,TNFAIP8L2,ZBTB16,PTPN22,PIK3R1,CD6,RAC2,H1-2,FYN,TSC22D3,STAT5A,ITK,PRKDC,WWP2,CD27,KRAS,CASP10,NOTCH1,GPR132,RAP1GDS1,CD5,BCL11B,ATM,EP300,PPP3CB,DFFA,OGT,KLRC1,PDCD1,CD44,GIMAP1,CASP6,PARP1,MAP3K2,GZMA,ARHGEF1,BCL2,PPP2R2B,SOCS1,PTPRC,TNFRSF18,CD247,XCL1,CD2,CASP8,XKR8,ANKZF1
Apoptosis of TREG cells	1.48E-04	0.75	IL2,TLN1,EP300,PTPN22,PDCD1
Killing of natural killer cells	1.68E-02	0.83	SLAMF1,HLA-A,PRF1,SLAMF6,RAB27A,LY9
Apoptosis of regulatory T lymphocytes	4.00E-07	0.94	IL2,TLN1,STAT5A,EP300,PTPN22,FAS,STAT5B,PDCD1
Cell death of naive T lymphocytes	7.54E-05	0.98	IL2,FYN,LCK,IL7R,PRF1,CASP3,FAS,IL2RG
Hyperplasia of T lymphocytes	1.08E-03	1.00	MAP2K7,MAPK14,MAP3K14,LAT,BAX,TNFRSF18,DUSP4,IKZF1

B-cell neoplasm	2.48E-03	1.00	MS4A1,GSK3B,PAG1,BUB3,PRF1,CDKN2A,PIM1,PDCD1,CD47,PARP1,BTG2,CD40LG,INPP5D,HIPK3,BCL2,IL7R,CASP3,NIBAN1,FCMR,BCL2L1,CD27,CASP8
Lifespan of blood cells	2.12E-02	1.00	SEMA4D,DOCK8,IFNG,CDKN2A,IKBKB
Development of regulatory T lymphocytes	2.39E-03	1.04	IL2,STAT4,IFNG,ICOS,STIM2,PRKCQ,IL2RB,GPR174,PARP1,STAT3,CREBBP,CBLB,ETS1,TBX21,S1PR1
Apoptosis of helper T lymphocytes	1.03E-02	1.08	IL2,CREB1,BCL2,FAS,CD6
Proliferation of CD8+ T lymphocyte	7.72E-03	1.11	IL2,RASA2,STAT4,IFNG,CD5,PDCD1,CD8A,TNFAIP3,CDKN1B,STAT5A,LCK,ANO6,BATF,UBASH3A,SOCS1,CBLB,PCBP2,PTPRC,CYTIP,DGKZ,TNFRSF18,STAT5B
Cell death of T lymphocytes	4.68E-15	1.13	ICOS,CDKN2A,IL2RG,STING1,CXCR4,IL7R,LCK,LAT,TRAF1,LAG3,TLE4,LTB,IL2,TLN1,EZR,PRDM1,ZC3H8,CD47,DNAJA3,BIRC6,BCL2L1,BAX,FCMR,STAT5B,ETS1,FAS,RHOH,SLC6A6,CREB1,IFNG,ZBTB16,PTPN22,H12,FYN,STAT5A,WWP2,CD27,CASP10,NOTCH1,GPR132,RAP1GDS1,CD5,BCL11B,CD99,MAP3K14,EP300,OGT,KLRC1,GIMAP1,PARP1,GZMA,ARHGEF1,BCL2,TNFRSF18,XCL1,CD2,ANKZF1,ATG5,PRKCQ,PRKAA1,DOCK8,STAT3,PIK3C3,CYLD,GRAP2,CD3E,CCL5,PRF1,SMPD1,IKBKB,BAD,SH2D2A,CDKN1B,CASP3,STK17B,GSK3B,GIMAP4,MAPK14,TNFAIP8L2,PIK3R1,CD6,RAC2,TSC22D3,DEF6,ITK,PRKDC,KRAS,ATM,DFFA,PPP3CB,PDCD1,CD44,CASP6,MAP3K2,PPP2R2B,SOCS1,PTPRC,CD247,SH2D1A,CASP8,XKR8
Cell death of B lymphocytes	2.61E-03	1.13	IFNG,FOXP1,CDKN2A,PIK3R1,MALT1,STAT3,STING1,INPP5D,NOC2L,TSC22D3,PRKCB,CD27,DUSP4,SKIL,S1PR1,MS4A1,IL2,CD5,SMPD1,PRF1,PDCD1,BAD,CD40LG,BCL2,PTPRC,FCMR,BAX,BCL2L1,ORMDL3,ETS1,FAS,CASP8,ANKZF1
Apoptosis of mononuclear leukocytes	1.62E-10	1.20	ICOS,CDKN2A,IL2RG,CXCR4,STING1,IL7R,LCK,PRKCB,TRAF1,TLE4,SKIL,LTB,S1PR1,IL2,TLN1,EZR,ZC3H8,CD47,DNAJA3,BIRC6,BCL2L1,BAX,FCMR,STAT5B,FAS,ETS1,RHOH,SLC6A6,CREB1,IFNG,FOXP1,ZBTB16,PTPN22,H12,FYN,STAT5A,WWP2,CD27,CASP10,NOTCH1,GPR132,RAP1GDS1,BCL11B,CD5,EP300,OGT,KLRC1,GIMAP1,PARP1,CD40LG,GZMA,BCL2,ARHGEF1,TNFRSF18,ORMDL3,XCL1,CD2,ANKZF1,ATG5,PRKCQ,MALT1,DOCK8,STAT3,NOC2L,DUSP4,PIK3C3,CYLD,GRAP2,CD3E,CCL5,PRF1,SMPD1,IKBKB,BAD,CDKN1B,CASP3,STK17B,GSK3B,TNFAIP8L2,PIK3R1,CD6,RAC2,INPP5D,TSC22D3,ITK,PRKDC,GADD45G,KRAS,ATM,DFFA,PPP3CB,PDCD1,CD44,CASP6,MAP3K2,SOCS1,PPP2R2B,PTPRC,CD247,CASP8,XKR8
Cell death of mononuclear leukocytes	1.39E-12	1.27	ICOS,CDKN2A,IL2RG,CXCR4,STING1,IL7R,LCK,PRKCB,LAT,TRAF1,LAG3,TLE4,SKIL,LTB,S1PR1,IL2,TLN1,EZR,PRDM1,ZC3H8,CD47,DNAJA3,BIRC6,BCL2L1,BAX,FCMR,STAT5B,FAS,ETS1,RHOH,SLC6A6,CREB1,IFNG,FOXP1,ZBTB16,PTPN22,H12,FYN,STAT5A,CD27,WWP2,MS4A1,CASP10,NOTCH1,GPR132,RAP1GDS1,BCL11B,CD5,CD99,EP300,MAP3K14,OGT,KLRC1,GIMAP1,PARP1,CD40LG,GZMA,BCL2,ARHGEF1,TNFRSF18,ORMDL3,XCL1,CD2,ANKZF1,ITGAL,ATG5,PRKAA1,PRKCQ,MALT1,DOCK8,STAT3,NOC2L,DUSP4,ZAP70,PIK3C3,CYLD,GRAP2,CD3E,CCL5,PRF1,SMPD1,IKBKB,SH2D2A,BAD,CDKN1B,CASP3,STK17B,GSK3B,GIMAP4,MAPK14,TNFAIP8L2,PIK3R1,CD6,RAC2,INPP5D,DEF6,TSC22D3,ITK,PRKDC,GADD45G,KRAS,ATM,PPP3CB,DFFA,PDCD1,CD44,CASP6,TNFAIP3,MAP3K2,SOCS1,PPP2R2B,PTPRC,CD247,SH2D1A,CASP8,XKR8
Apoptosis of lymphocytes	3.39E-11	1.29	ICOS,CDKN2A,IL2RG,CXCR4,STING1,IL7R,LCK,PRKCB,TRAF1,TLE4,SKIL,LTB,S1PR1,IL2,TLN1,EZR,ZC3H8,CD47,DNAJA3,BIRC6,BCL2L1,BAX,FCMR,STAT5B,FAS,ETS1,RHOH,SLC6A6,CREB1,IFNG,FOXP1,ZBTB16,PTPN22,H12,FYN,STAT5A,WWP2,CD27,CASP10,NOTCH1,GPR132,RAP1GDS1,BCL11B,CD5,EP300,OGT,KLRC1,GIMAP1,PARP1,CD40LG,GZMA,BCL2,ARHGEF1,TNFRSF18,ORMDL3,XCL1,CD2,ANKZF1,ATG5,PRKCQ,MALT1,DOCK8,STAT3,NOC2L,DUSP4,PIK3C3,CYLD,GRAP2,CD3E,CCL5,PRF1,SMPD1,IKBKB,BAD,CDKN1B,CASP3,STK17B,GSK3B,TNFAIP8L2,PIK3R1,CD6,RAC2,INPP5D,TSC22D3,ITK,PRKDC,KRAS,ATM,DFFA,PPP3CB,PDCD1,CD44,CASP6,MAP3K2,SOCS1,PPP2R2B,PTPRC,CD247,CASP8,XKR8
Frequency of TREG cells	8.99E-03	1.36	IL2,PARP1,MAPK14,SMPD1,PTPN22,CD6
Cell death of helper T lymphocytes	1.71E-03	1.40	IL2,CREB1,BCL2,SMPD1,CASP3,FAS,CD6
Lymphoid hyperplasia	7.54E-03	1.43	CYLD,MAP2K7,MAPK14,CD40LG,MAP3K14,BAX,LAT,TNFRSF18,DUSP4,IKZF1
Cell death of lymphocytes	6.35E-13	1.44	ICOS,CDKN2A,IL2RG,CXCR4,STING1,IL7R,LCK,PRKCB,LAT,TRAF1,LAG3,TLE4,SKIL,LTB,S1PR1,IL2,TLN1,EZR,PRDM1,ZC3H8,CD47,DNAJA3,BIRC6,BCL2L1,BAX,FCMR,STAT5B,FAS,ETS1,RHOH,SLC6A6,CREB1,IFNG,FOXP1,ZBTB16,PTPN22,H12,FYN,STAT5A,WWP2,CD27,MS4A1,CASP10,NOTCH1,GPR132,RAP1GDS1,BCL11B,CD5,CD99,EP300,MAP3K14,OGT,KLRC1,GIMAP1,PARP1,CD40LG,GZMA,BCL2,ARHGEF1,TNFRSF18,ORMDL3,XCL1,CD2,ANKZF1,ITGAL,ATG5,PRKAA1,PRKCQ,MALT1,DOCK8,STAT3,NOC2L,DUSP4,PIK3C3,CYLD,GRAP2,CD3E,CCL5,PRF1,SMPD1,IKBKB,SH2D2A,BAD,CDKN1B,CASP3,STK17B,GSK3B,GIMAP4,MAPK14,TNFAIP8L2,PIK3R1,CD6,RAC2,INPP5D,DEF6,TSC22D3,ITK,PRKDC,KRAS,ATM,DFFA,PPP3CB,PDCD1,CD44,CASP6,TNFAIP3,MAP3K2,SOCS1,PPP2R2B,PTPRC,CD247,SH2D1A,CASP8,XKR8
Apoptosis of B lymphocytes	6.10E-03	1.51	IFNG,FOXP1,CDKN2A,PIK3R1,MALT1,STAT3,NOC2L,TSC22D3,PRKCB,CD27,DUSP4,SKIL,S1PR1,IL2,CD5,SMPD1,PRF1,BAD,CD40LG,BCL2,PTPRC,BAX,BCL2L1,FCMR,ORMDL3,ETS1,FAS,CASP8,ANKZF1
Frequency of regulatory T lymphocytes	1.29E-02	1.64	CYLD,IL2,PARP1,MAPK14,SMPD1,RICTOR,PTPN22,CD6
Number of activated T lymphocyte	9.99E-03	2.61	DUSP10,IL2,GRAP2,PRF1,IL2RB,SOCS1,N4BP1
Morphology of lymphocytes	2.78E-11		IRF1,PRKCQ,FCER1G,RASGRP1,IL2RG,MALT1,SYK,CD8A,PIK3AP1,IL7R,LCK,CBLB,LAT,PRKCH,CYTIP,LTB,CYLD,GRAP2,CD3E,IL2,TXNIP,CD46,PRDM1,BATF,CDKN2B,BCL2L1,FCMR,BAX,ETS1,STAT5B,FAS,STK4,EPS15,FLT3LG,SEMA4D,RHOH,IFNG,IL2RB,PTPN22,TAP1,LCP1,IKZF1,INPP5D,ITPKB,ITK,TOX,CREBBP,PRKDC,TRAF3IP3,CD8B,BCL11B,TNFSF14,MARK2,PPP3CB,LY9,SLA,THEMIS,HLA-A,BCL2,RUNX3,SOCS1,PTPRC,CD247,SH2D1A,ERN1,PGAP3,TBX21,LYST
Abnormal morphology of lymphocytes	4.10E-11		IRF1,PRKCQ,FCER1G,RASGRP1,IL2RG,MALT1,SYK,CD8A,PIK3AP1,IL7R,LCK,CBLB,LAT,CYTIP,PRKCH,LTB,CYLD,GRAP2,CD3E,IL2,TXNIP,PRDM1,BATF,CDKN2B,FCMR,BAX,ETS1,STAT5B,FAS,STK4,EPS15,FLT3LG,SEMA4D,RHOH,IFNG,IL2RB,PTPN22,TAP1,LCP1,IKZF1,INPP5D,ITPKB,ITK,TOX,CREBBP,PRKDC,CD8B,BCL11B,TNFSF14,MARK2,PPP3CB,LY9,SLA,THEMIS,HLA-A,BCL2,RUNX3,SOCS1,PTPRC,CD247,SH2D1A,ERN1,PGAP3,TBX21
Abnormal morphology of mononuclear leukocytes	5.93E-11		IRF1,FTL,PRKCQ,FCER1G,RASGRP1,IL2RG,MALT1,SYK,CD8A,PIK3AP1,IL7R,LCK,CBLB,LAT,CYTIP,PRKCH,LTB,CYLD,GRAP2,CD3E,IL2,TXNIP,PRDM1,BATF,CDKN2B,BAX,FCMR,ETS1,STAT5B,FAS,STK4,EPS15,FLT3LG,SEMA4D,RHOH,IFNG,IL2RB,PTPN22,TAP1,LCP1,IKZF1,INPP5D,ITPKB,ITK,TOX,CREBBP,PRKDC,CD8B,BCL11B,TNFSF14,MARK2,PPP3CB,LY9,SLA,THEMIS,HLA-A,BCL2,RUNX3,SOCS1,PTPRC,CD247,SH2D1A,ERN1,TBX21,PGAP3
Morphology of T lymphocytes	6.31E-11		IRF1,RHOH,PRKCQ,PTPN22,FCER1G,TAP1,RASGRP1,IL2RG,MALT1,INPP5D,CD8A,ITPKB,IL7R,LCK,ITK,TOX,CBLB,PRKCH,CYTIP,LAT,CD3E,GRAP2,IL2,TRAF3IP3,CD8B,BCL11B,MARK2,TNFSF14,CD46,PPP3CB,LY9,SLA,THEMIS,HLA-A,BCL2,RUNX3,SOCS1,PTPRC,BAX,BCL2L1,STAT5B,ETS1,FAS,SH2D1A,CD247,PGAP3,TBX21,LYST
Morphology of mononuclear leukocytes	6.55E-11		IRF1,FTL,PRKCQ,FCER1G,RASGRP1,IL2RG,MALT1,SYK,CD8A,PIK3AP1,IL7R,LCK,CBLB,LAT,CYTIP,PRKCH,LTB,CYLD,GRAP2,CD3E,IL2,TXNIP,CD46,PRDM1,BATF,CDKN2B,BCL2L1,FCMR,BAX,ETS1,STAT5B,FAS,STK4,EPS15,FLT3LG,SEMA4D,RHOH,IFNG,IL2RB,PTPN22,TAP1,LCP1,IKZF1,INPP5D,ITPKB,ITK,TOX,CREBBP,PRKDC,CCR2,TRAF3IP3,CD8B,BCL11B,TNFSF14,MARK2,PPP3CB,LY9,SLA,THEMIS,HLA-A,BCL2,RUNX3,SOCS1,PTPRC,CD247,SH2D1A,ERN1,PGAP3,TBX21,LYST
Abnormal morphology of leukocytes	1.44E-10		IRF1,PML,FTL,PRKCQ,FCER1G,RASGRP1,IL2RG,GATA3,MALT1,SYK,CD8A,PIK3AP1,IL7R,LCK,CBLB,LAT,PRKCH,CYTIP,ASXL1,LTB,CYLD,GRAP2,CD3E,IL2,TXNIP,SMPD1,PRDM1,PSTPIP1,BAD,BATF,CDKN2B,BAX,FCMR,ETS1,STAT5B,FAS,STK4,EPS15,FLT3LG,SEMA4D,RHOH,IFNG,TNFAIP8L2,IL2RB,PTPN22,TAP1,LCP1,IKZF1,INPP5D,ITPKB,ITK,TOX,CREBBP,PRKDC,CD8B,BCL11B,ATM,MARK2,TNFSF14,PPP3CB,LY9,SLA,THEMIS,HLA-A,BCL2,RUNX3,SOCS1,PTPRC,CD247,SH2D1A,ERN1,PGAP3,TBX21,CASP8
Abnormal morphology of T lymphocytes	1.48E-09		IRF1,RHOH,PRKCQ,PTPN22,FCER1G,TAP1,RASGRP1,IL2RG,MALT1,CD8A,ITPKB,IL7R,LCK,ITK,TOX,CBLB,CYTIP,PRKCH,LAT,CD3E,GRAP2,IL2,CD8B,BCL11B,MARK2,TNFSF14,PPP3CB,LY9,SLA,THEMIS,HLA-A,BCL2,RUNX3,SOCS1,PTPRC,BAX,STAT5B,FAS,ETS1,CD247,SH2D1A,PGAP3,TBX21
Abnormal morphology of immune system	2.13E-08		IRF1,PML,FTL,PRKCQ,FCER1G,RASGRP1,IL2RG,MALT1,GATA3,SYK,CD8A,PIK3AP1,IL7R,LCK,CBLB,LAT,CYTIP,PRKCH,ASXL1,LTB,CYLD,GRAP2,CD3E,IL2,TXNIP,SMPD1,PRDM1,PSTPIP1,GBA,BAD,DOCK11,BATF,CDKN2B,BAX,FCMR,STAT5B,ETS1,FAS,STK4,EPS15,FLT3LG,SEMA4D,RHOH,IFNG,TNFAIP8L2,IL2RB,PTPN22,TAP1,LCP1,IKZF1,INPP5D,ITPKB,STAT5A,ITK,TOX,CREBBP,PRKDC,PTGDR,CCR2,CD8B,BCL11B,TNFSF14,MARK2,ATM,PPP3CB,LY9,NR1H2,SLA,THEMIS,HLA-A,SOAT1,BCL2,RUNX3,SOCS1,PTPRC,CD247,SH2D1A,ERN1,PGAP3,TBX21,CASP8
Development of natural killer T lymphocytes	2.68E-08		CYLD,IRF1,KRAS,NOTCH1,STIM2,ZBTB16,PRKCQ,RICTOR,IKBKB,MALT1,FYN,TNFAIP3,ITK,DGKZ,ETS1,SH2D1A,PIK3C3

Morphology of lymphoid tissue	5.11E-08	RHOH,CD3E,GRAP2,EP300,IL2RB,PTPN22,SLA,RASGRP1,LCP1,MALT1,CD8A,BCL2,LCK,BATF,PTPRC,STAT5B,SH2D1A,CD247,CASP8,STK4,EPS15,PIK3C3,LTB
Lack of lymphocytes	9.17E-08	IL2RB,FCER1G,TAP1,IL2RG,IKZF1,MALT1,SYK,CD8A,PIK3AP1,ITPKB,IL7R,LCK,CREBBP,LAT,PRKDC,LTB,CD3E,BCL11B,PRDM1,HLA-A,BATF,ETS1,SH2D1A,CD247,TBX21
Lymphopenia	1.57E-07	ICOS,IL2RB,FCER1G,TAP1,IL2RG,IKZF1,MALT1,SYK,CD8A,PIK3AP1,ITPKB,IL7R,LCK,CREBBP,LAT,PRKDC,LTB,CD3E,BCL11B,PRDM1,HLA-A,BATF,ETS1,CD247,SH2D1A,TBX21
Morphology of leukocytes	1.77E-07	IRF1,PML,FTL,PRKCQ,CDKN2A,FCER1G,RASGRP1,IL2RG,GATA3,MALT1,SYK,CD8A,PIK3AP1,IL7R,LCK,CBLB,LAT,PRKCH,CYTIP,ASXL1,LTB,CYLD,GRAP2,CD3E,IL2,TXNIP,SMPD1,CD46,PRDM1,PSTPIP1,GBA,BAD,NRAS,DOCK11,BATF,CDKN2B,BCL2L1,BAX,FCMR,STAT5B,FAS,ETS1,STK4,EPS15,FLT3LG,SEMA4D,RHOH,TNFAIP8L2,IFNG,IL2RB,PTPN22,TAP1,CTCF,LCP1,IKZF1,INPP5D,ITPKB,STAT5A,ITK,TOX,CREBBP,PRKDC,PTGDR,CCR2,TRAF3IP3,CD8B,BCL11B,TNFSF14,MARK2,ATM,PPP3CB,LY9,NR1H2,SLA,THEMIS,HLA-A,SOAT1,BCL2,RUNX3,SOCS1,PTPRC,CD247,ERN1,SH2D1A,PGAP3,TBX21,CASP8,LYST
Lack of T lymphocytes	5.05E-06	CD3E,BCL11B,FCER1G,TAP1,IL2RG,HLA-A,CD8A,ITPKB,IL7R,LAT,SH2D1A,CD247,TBX21
Function of natural killer cells	1.09E-05	TXNIP,STAT4,ICOS,IFNG,KLRB1,PRF1,FCER1G,TAPBP,CD96,FYN,PTPRC,ETS1,LAG3,SH2D1A,CD247,TBX21,GADD45G
Abnormal morphology of lymphoid organ	3.06E-05	GRAP2,CD3E,RHOH,EP300,PTPN22,SLA,RASGRP1,MALT1,CD8A,BCL2,LCK,STAT5B,CD247,CASP8
Abnormal morphology of hematopoietic progenitor cells	5.32E-05	RHOH,CD3E,GRAP2,PTPN22,SLA,RASGRP1,IKZF1,MALT1,SYK,CD8A,LCK,CREBBP,PTPRC,STAT5B,CD247,ERN1,PRKDC
Abnormal morphology of follicular B lymphocytes	7.54E-05	BATF,IL2RB,PTPRC,SH2D1A,EPS15,STK4,LTB,LCP1
Abnormal morphology of thoracic cavity	7.63E-05	MALT1,GRAP2,CD3E,RHOH,CD8A,LCK,PTPN22,STAT5B,CD247,SLA,CD69,RASGRP1
Immune mediated inflammatory disease	9.33E-05	EIF2AK2,NCOR2,RAB1B,ICOS,TNRC6B,FCER1G,GATA3,RNF14,TMF1,CXCR4,CCDC59,STING1,STAT3,HLADRB5,MLLT6,TRAF1,ZNF326,DTX3L,NR4A2,S1PR1,ISG20,DNAJA4,IL2,TRIM22,RRN3,CCL5,WNK1,PRDM1,DUSP2,ADSS2,CD69,USP15,PHF1,RNF149,CDKN1B,ADGRE5,GNLY,BCL2L1,BAX,STK17B,RCSD1,JUNB,F11R,IFNG,ZNF331,SELENOK,PDE4B,HSF2,LCP1,IKZF1,CCND3,INPP5D,FKBP5,DEDD2,HSPA8,SLAMF6,EHD4,SAFB,NONO,PPP2CA,DCUN1D1,EP300,LY9,RABGAP1L,PDCD1,NUMB,BST2,TNFAIP3,RGS1,MAP3K2,GZMA,BCL2,SOCS1,PTPRC,TBRG1,CASP8,TAGAP,AHNAK,LDHB
Abnormal morphology of B lymphocytes	1.04E-04	SEMA4D,FLT3LG,CYLD,CD3E,IL2RB,PRDM1,IL2RG,IKZF1,LCP1,MALT1,SYK,PIK3AP1,BATF,CREBBP,PTPRC,FCMR,BAX,ETS1,SH2D1A,ERN1,PRKDC,STK4,EPS15,LTB
Abnormal morphology of body cavity	1.04E-04	GRAP2,CD3E,RHOH,EP300,PTPN22,SLA,CD69,RASGRP1,MALT1,CD8A,BCL2,LCK,STAT5B,CD247,CASP8
Morphology of lymph follicle	1.04E-04	BATF,IL2RB,PTPRC,SH2D1A,STK4,PIK3C3,EPS15,LTB,LCP1
Abnormal morphology of thymus gland	1.20E-04	MALT1,CD3E,GRAP2,RHOH,CD8A,LCK,PTPN22,STAT5B,CD247,SLA,RASGRP1
Differentiation of natural killer cells	1.44E-04	FLT3LG,IRF1,SLAMF1,HECTD1,IKZF1,INPP5D,STAT5A,SP3,TOX,SLAMF6,KAT7,PTPRC,STAT5B
Abnormal quantity of lymphocytes	1.55E-04	MS4A1,CYLD,IRF1,RHOH,IL2,ICOS,IL2RG,SYK,SIT1,ARHGEF6,CD8A,ARHGEF1,LCK,TOX,PTPRC,STAT5B,POLM,SH2D1A,CD247,PRKDC
Abnormal morphology of CD8+ T lymphocyte	1.87E-04	CD3E,IL2,CD8B,PPP3CB,TAP1,HLA-A,CD8A,ITPKB,BCL2,RUNX3,TOX,LAT,CYTIP,ETS1,TBX21
Apoptosis of effector T regulatory cells	2.24E-04	IL2,TLN1,PTPN22,PDCD1
Arrest in differentiation of T lymphocytes	2.63E-04	GRAP2,CD3E,RHOH,ITPKB,IL7R,CD3D,PTPRC,LAT,NCOR1,PRKDC,ZAP70
Rheumatic Disease	2.77E-04	EIF2AK2,NCOR2,RAB1B,ICOS,TNRC6B,SIPA1,NCF1,LPIN1,RNF14,TMF1,CXCR4,CCDC59,STING1,STAT3,PRKCB,MLLT6,TRAF1,ZNF326,DTX3L,NR4A2,S1PR1,ISG20,DNAJA4,CD3E,IL2,TRIM22,CCL5,TCF7,WNK1,IKBKB,PRDM1,DUSP2,ADSS2,CD69,USP15,RNF149,CDKN1B,ADGRE5,GNLY,BCL2L1,BAX,STK17B,RCSD1,F11R,IFNG,LCP1,IKZF1,CCND3,METTL9,INPP5D,FKBP5,DEDD2,HSPA8,SLAMF6,EHD4,SAFB,CD27,MAP3K3,NONO,SIGIRR,DCUN1D1,LY9,RABGAP1L,NUMB,BST2,TNFAIP3,RGS1,MAP3K2,GZMA,BCL2,RUNX3,SOCS1,PTPRC,TBRG1,TAGAP,LDHB
Lack of CD8+ T lymphocyte	4.52E-04	CD3E,HLA-A,CD8A,ITPKB,LAT,TAP1
Eosinophilia	5.31E-04	CXCR4,STAT3,SYK,CD40LG,IFNG,CD7,CD3G,CD5,IKBKB,PPP3CA,CD247
Systemic autoimmune syndrome	5.51E-04	EIF2AK2,NCOR2,RAB1B,TNRC6B,ICOS,RNF14,TMF1,CCDC59,STING1,STAT3,CXCR4,MLLT6,TRAF1,DTX3L,ZNF326,NR4A2,ISG20,S1PR1,DNAJA4,TRIM22,IL2,CCL5,WNK1,PRDM1,DUSP2,CD69,ADSS2,USP15,CDKN1B,RNF149,ADGRE5,GNLY,BCL2L1,BAX,FAS,RCSD1,STK17B,F11R,IFNG,LCP1,IKZF1,CCND3,FKBP5,DEDD2,HSPA8,SLAMF6,EHD4,SAFB,NONO,DCUN1D1,LY9,RABGAP1L,PDCD1,NUMB,BST2,CD40LG,TNFAIP3,MAP3K2,GZMA,BCL2,SOCS1,PTPRC,TBRG1,CASP8,TAGAP,LDHB
Function of CD4+ T-lymphocytes	6.81E-04	IL2,SLAMF1,ITGAL,TNFAIP8L2,ICOS,PRKCQ,PPP3CB,PTPN22,FCER1G,CD44,CD69,FYN,INPP5D,LAG3,SH2D1A,CD27,PGAP3,TBX21,STK4
Abnormal morphology of double-negative T lymphocyte	6.98E-04	MALT1,CD3E,RHOH,GRAP2,CD8A,LCK,STAT5B,CD247
Abnormal morphology of gamma-delta T lymphocytes	6.98E-04	IL2,IL7R,TNFSF14,ITK,LAT,FCER1G,CD247,IL2RG

Function of cytotoxic T cells	8.60E-04	GSK3B,IL2,ITGAL,CD8B,IFNG,TNFAIP8L2,TNFSF14,PRF1,TAPBP,HLA-A,CD8A,SH2D1A,CD27,TBX21
Clearance of neutrophils	8.92E-04	CCR2,GPR132,HMGB1,CD44,XKR8,ANXA1
Exhaustion of CD8+ T lymphocyte	1.01E-03	ANO6,IL2RB,TNFRSF18,CD27
Familial autoimmune disease	1.01E-03	CD40LG,ICOS,FAS,PDCD1
Arrest in differentiation of leukocytes	1.07E-03	RHOH,GRAP2,CD3E,JAK1,PIK3R1,IKZF3,IL2RG,SYK,FYN,ITPKB,CD3D,IL7R,PTPRC,NCOR1,LAT,PRKDC,CASP8,ZAP70
Lack of gamma-delta T lymphocytes	1.11E-03	IL7R,FCER1G,LAT,CD247,IL2RG
Systemic lupus erythematosus	1.40E-03	EIF2AK2,NCOR2,TRIM22,IFNG,ICOS,LY9,RABGAP1L,CCND3,BST2,STAT3,CDKN1B,BCL2,SLAMF6,BAX,BCL2L1,DTX3L,ISG20
Inflammation of joint	1.76E-03	RAB1B,TNRC6B,ICOS,SIPA1,NCF1,RNF14,LPIN1,TMF1,CCDC59,STING1,STAT3,CXCR4,MLLT6,PRKCB,TRAF1,ZNF326,NR4A2,S1PR1,DNAJA4,CD3E,IL2,CCL5,TCF7,WNK1,IKBKB,PRDM1,DUSP2,CD69,ADSS2,USP15,RNF149,ADGRE5,GNLY,RCSD1,STK17B,F11R,IFNG,LCP1,IKZF1,CCND3,METTL9,FKBP5,DEDD2,HSPA8,EHD4,SAFB,CD27<br>,NONO,MAP3K3,SIGIRR,DCUN1D1,NUMB,TNFAIP3,MAP3K2,GZMA,RUNX3,SOCS1,PTPRC,TBRG1,TAGAP,LDHB
Cell survival of CD4+ T-lymphocytes	1.83E-03	PIM1,IL2RG,PYCARD
Contact growth inhibition of T lymphocytes	1.83E-03	LCK,PTPRC,ZAP70
Generation of CD8+ T lymphocyte	1.83E-03	ITGAL,HLA-A,IFNG
Insulin-dependent diabetes mellitus secretory diarrhea syndrome	1.83E-03	CD40LG,ICOS,PDCD1
Selection of natural killer T lymphocytes	1.83E-03	ELF1,ELK4,ELK1
Hypereosinophilia	1.97E-03	CXCR4,STAT3,CD40LG,CD7,CD3G,CD5,IKBKB,PPP3CA,CD247
Arrest in differentiation of lymphocytes	2.39E-03	RHOH,GRAP2,CD3E,JAK1,IKZF3,PIK3R1,IL2RG,SYK,FYN,ITPKB,CD3D,IL7R,PTPRC,NCOR1,LAT,PRKDC,ZAP70
Clearance of leukocytes	2.60E-03	PPARD,CCR2,HMGB1,GPR132,CD44,XKR8,ANXA1
Abnormal morphology of natural killer T lymphocytes	2.74E-03	ITK,SH2D1A,TBX21,IL2RG
Deficiency of lymphocytes	2.74E-03	BCL2,EP300,PTPRC,GIMAP1
Lack of B-1a lymphocytes	2.74E-03	MALT1,PIK3AP1,ETS1,IKZF1
Lymphoid immunodeficiency	2.74E-03	BCL2,EP300,FAS,GIMAP1
Cell-cell adhesion of leukocytes	2.88E-03	SYK,ITGAL,F11R,CD40LG,CCL5,ICOS,ADGRE5,ITGA4,TNIP1,PTPRC
Abnormal quantity of leukocytes	3.23E-03	MS4A1,CYLD,IRF1,RHOH,IL2,ICOS,IL2RG,IKZF1,SYK,SIT1,CD8A,ARHGEF6,ARHGEF1,LCK,TOX,PTPRC,STAT5B,POLM,SH2D1A,CD247,PRKDC
Abnormal morphology of natural killer cells	3.25E-03	INPP5D,TXNIP,IFNG,LCK,IL2RB,CD247,IL2RG,IKZF1
Rett Syndrome	3.26E-03	CCL5,MBP,IFNG,NKG7,YBX3,ZNF131,TAF9B,FCER1G,CD6,SLC16A1,NCR3,CASP6,CXCR6,PITPNC1,HMBOX1,BTN3A1,ABLIM1,DNAJB6,XCL1,TBX21,DUSP4,ERAP2
Insulin-dependent diabetes mellitus	4.05E-03	CD40LG,ICOS,IFNG,PTPRC,PDCD1

Function of mast cells	4.11E-03	RASGRP4,FCER1G,PIM1,ABCC1,DUSP2,RASGRP1,IL2RG,STAT5A,CBLB,DGKZ,LAT,STAT5B,PTGDR
Abnormal number of leukocytes	4.13E-03	MS4A1,CYLD,IRF1,ICOS,IL2RG,IKZF1,SYK,ARHGEF6,CD8A,LCK,TOX,PTPRC,POLM,CD247,PRKDC
Development of gamma-delta T lymphocytes	4.18E-03	ITGAL,NOTCH1,CD3G,CD3D,SH2D1A,IL2RG
Atopic dermatitis	4.55E-03	RRN3,ZNF331,SELENOK,FCER1G,PDE4B,DUSP2,HSF2,CXCR4,TNFAIP3,PHF1,HLA-DRB5,AHNAK,JUNB,NR4A2
Dysfunction of lymphocytes	4.82E-03	MS4A1,CYLD,TNFAIP8L2,IFNG,TLN1,ICOS,POLH,MAP3K14,LAX1,PIK3R1,CD6,GBA,BAD,ELF1,CXCR4,INPP5D,ARHGEF6,RGS1,PTPRC,STK4,TBC1D10C
Development of invariant natural killer T cells	5.39E-03	KRAS,STIM2,ITK,RICTOR,IKBKB,DGKZ,PIK3C3
Glucose metabolism disorder	5.39E-03	PPARD,CD40LG,CREB1,ICOS,IFNG,PTPRC,PDCD1
Lack of B lymphocytes	5.76E-03	MALT1,SYK,PIK3AP1,BATF,CREBBP,ETS1,PRDM1,PRKDC,IL2RG,LTB,IKZF1
Abnormal morphology of marginal-zone B lymphocytes	5.77E-03	IL2RB,EPS15,STK4,LCP1
Activation of effector T lymphocytes	5.77E-03	MALT1,DEF6,BAX,ZAP70
Humoral immunodeficiency	5.77E-03	IL2,BCL2,EP300,GIMAP1
Development of pre-T lymphocytes	5.98E-03	CBFB,IL2,CXCR4,CD3G,RICTOR,IL2RB,RASGRP1,IL2RG
Allergy	6.01E-03	RRN3,ZNF331,SELENOK,FCER1G,PDE4B,PDCD1,DUSP2,HSF2,GATA3,CXCR4,TNFAIP3,PHF1,HLA-DRB5,AHNAK,JUNB,NR4A2
Binding of interferon-gamma activated sequence	6.66E-03	IL2,IFNG,CD2
Binding of keratinocytes	6.66E-03	ITGAL,IFNG,CD44
Cell death of effector memory helper T cells	6.66E-03	IL2,SMPD1,FAS
Cell-cell adhesion of granulocytes	6.66E-03	CD40LG,ICOS,ADGRE5
Contact growth inhibition of pre-B lymphocytes	6.66E-03	CDKN1B,IKZF3,IKZF1
Deficiency of B lymphocytes	6.66E-03	BCL2,EP300,GIMAP1
Dysfunction of T lymphocytes	6.66E-03	ICOS,TLN1,GBA
Influx of T lymphocytes	6.66E-03	IFNG,STIM2,ITK
Lack of natural killer T lymphocytes	6.66E-03	SH2D1A,TBX21,IL2RG
Maintenance of regulatory T lymphocytes	6.66E-03	IL2,ICOS,PIK3C3
Number of iNKT2 cells	6.66E-03	TNFAIP3,BCL11B,LY9
Survival of gamma-delta T lymphocytes	6.66E-03	IL2,BCL2,IL2RG

Abnormal morphology of CD4+ T-lymphocytes	7.54E-03	GRAP2,CD3E,ITPKB,BCL2,MARK2,PPP3CB,PTPRC,LAT,RASGRP1,IL2RG
B cell cancer	1.04E-02	MS4A1,GSK3B,PAG1,BUB3,PRF1,PIM1,PDCD1,CD47,BTG2,PARP1,CD40LG,HIPK3,BCL2,CASP3,NIBAN1,FCMR,BCL2L1,CD27,CASP8
Cell death of peripheral T lymphocyte	1.04E-02	GIMAP4,EZR,FAS,GIMAP1
Condensation of chromatin	1.04E-02	IFNG,DFFA,FAS,NCAPH2
Development of alpha-beta T lymphocytes	1.04E-02	FYN,NOTCH1,CD3G,LCK
Development of intraepithelial lymphocytes	1.04E-02	IRF1,STIM2,LCK,PTPRC
Selection of B lymphocytes	1.04E-02	SYK,BAX,FAS,ZAP70
Development of Th1 cells	1.25E-02	GATA3,IL2,STAT4,IFNG,PRKCQ,PRKAA1
Development of pro-T lymphocytes	1.31E-02	CBFB,IL2,CXCR4,CD3G,RICTOR,IL2RB,RASGRP1
Abnormal function of B lymphocytes	1.32E-02	MS4A1,CYLD,TNFAIP8L2,IFNG,ICOS,POLH,MAP3K14,LAX1,PIK3R1,CD6,BAD,ELF1,CXCR4,INPP5D,ARHGEF6,RGS1,PTPRC,STK4,TBC1D10C
Homeostasis of B lymphocytes	1.38E-02	TNFAIP3,ICOS,RC3H2,PPP2R3C,BCL2,DOCK11,RICTOR,CASP3,IKBKB,BAX
Chronic inflammatory disorder	1.49E-02	RAB1B,TNRC6B,ICOS,RNF14,TMF1,CCDC59,STING1,STAT3,CXCR4,MLLT6,TRAF1,ZNF326,NR4A2,S1PR1,DNAJA4,IL2,CCL5,WNK1,PRDM1,DUSP2,CD69,ADSS2,USP15,RNF149,ADGRE5,GNLY,RCSD1,STK17B,F11R,IFNG,LCP1,IKZF1,FKBP5,DEDD2,HSPA8,EHD4,SAFB,NONO,DCUN1D1,BCL11B,PDCD1,NUMB,TNFAIP3,MAP3K2,GZMA,S<br>OCS1,PTPRC,TBRG1,TAGAP,LDHB
Accumulation of naive T lymphocytes	1.50E-02	BCL2,IL2RG
Accumulation of reactive oxygen species	1.50E-02	IL2,PIK3C3
Activation of effector memory cytotoxic T cells	1.50E-02	MALT1,ZAP70
Activation of protein	1.50E-02	SOCS1,PTPRC
Adhesion of keratinocytes	1.50E-02	IFNG,CD44
Apoptosis of Th17 cells	1.50E-02	IL2,CD6
Apoptosis of central Treg cells	1.50E-02	TLN1,PTPN22
Apoptosis of conventional dendritic cells	1.50E-02	FLT3LG,IL2
Apoptosis of large granular lymphocytes	1.50E-02	STAT3,FAS
Arrest in development of alpha-beta T lymphocytes	1.50E-02	FYN,LCK
Autophagy of T lymphocytes	1.50E-02	ATG5,PIK3C3
Cell division of memory T lymphocytes	1.50E-02	IL2,IL2RB
Cell spreading of natural killer cells	1.50E-02	ITGAL,RAP1B

Clonal expansion of thymocytes	1.50E-02	SYK,ZAP70
Cytolysis of intraepithelial lymphocytes	1.50E-02	LCK,PTPRC
Cytotoxicity of memory T lymphocytes	1.50E-02	IFNG,CD27
Degranulation of CD8+ T lymphocyte	1.50E-02	LAG3,PDCD1
Development of memory natural killer cells	1.50E-02	STAT3,IL2RB
Development of pro T2 thymocytes	1.50E-02	IL2,IL2RB
Development of type II natural killer T cells	1.50E-02	ZBTB16,SH2D1A
Distension of endoplasmic reticulum	1.50E-02	FAS,CD2
Exocytosis of tertiary granules	1.50E-02	VAMP2,STX4
Expansion of CD56bright natural killer cells	1.50E-02	IL2,IL2RB
Fragmentation of nucleus	1.50E-02	DFFA,CASP3
Function of bone marrow-derived mast cells	1.50E-02	RASGRP4,RASGRP1
Hyperplasia of regulatory T lymphocytes	1.50E-02	MAP3K14,TNFRSF18
Hyperplasia of thymocytes	1.50E-02	MAP2K7,IKZF1
Immunostimulatory potential of conventional dendritic cells	1.50E-02	FLT3LG,IL2
Influx of CD4+ T-lymphocytes	1.50E-02	IFNG,STIM2
Influx of eosinophils	1.50E-02	LY96,ITK
Inhibition of lymphokine activated killer cells	1.50E-02	CLEC2D,KLRB1
Maturation of inflammatory monocytes	1.50E-02	IFNG,GPR132
Maturation of phagolysosome	1.50E-02	SLAMF1,FAS
Neck neoplasm	1.50E-02	PTPRC,ANXA1
Phagocytosis of bone marrow-derived neutrophils	1.50E-02	CD47,HMGB1
Positive selection of B lymphocytes	1.50E-02	SYK,ZAP70
Positive selection of natural killer T lymphocytes	1.50E-02	ELK4,ELK1
Quantity of lymphoid dendritic cells	1.50E-02	FLT3LG,IKZF1

Rejection of lymphokine activated killer cells	1.50E-02	IL2,KLRB1
Release of B lymphocytes	1.50E-02	ARHGEF1,S1PR1
Release of prostaglandin E2	1.50E-02	IFNG,IRF2
Replication of Dengue virus 2	1.50E-02	STING1,HMGB1
Repopulation by blood cells	1.50E-02	FLT3LG,DOK2
Switching of T lymphocytes	1.50E-02	IL2,CD46
T-cell-independent type-2 extrafollicular antibody response	1.50E-02	SH3KBP1,IKBKB
Transendothelial migration of effector memory T lymphocytes	1.50E-02	ZAP70,MYH9
Turnover of T lymphocytes	1.50E-02	IL2,GRAP2
Abnormal morphology of B-1 lymphocytes	1.51E-02	MALT1,SEMA4D,PIK3AP1,ETS1,IKZF1
Abnormal morphology of alpha-beta intraepithelial T cell	1.51E-02	IL2,LCK,PTPRC,CD247,IL2RG
Arrest in cell cycle progression of T lymphocytes	1.51E-02	ATM,CDKN2A,BCL2L1,LAG3,SKIL
Abnormal morphology of regulatory T lymphocytes	1.51E-02	THEMIS,IL2,TOX
Aneuploidy of thymocytes	1.51E-02	BCL2,ATM,BAX
Cellular infiltration by CD8+ T lymphocyte	1.51E-02	NR1H2,LAG3,PDCD1
Conversion of induced regulatory T-lymphocyte	1.51E-02	IL2,PARP1,BCL11B
Modification of genomic DNA	1.51E-02	IRF1,PARP1,ATRX
Phospholipid flip-flop of phosphatidylserine	1.51E-02	IFNG,FAS,CD2
Quantity of central memory cytotoxic T cells	1.51E-02	MALT1,CD40LG,ICOS
Survival of intraepithelial lymphocytes	1.51E-02	BCL2,IL2RB,BCL2L1
Immunodeficiency	1.68E-02	IL2,BCL2,EP300,FAS,CASP8,GIMAP1
Abnormal morphology of effector T lymphocytes	1.70E-02	PRKCQ,PRKCH,FCER1G,CD247
Degranulation of T lymphocytes	1.70E-02	VTI1B,RAB27A,LAG3,PDCD1
Immune response of CD8+ T lymphocyte	1.70E-02	PARP1,CD27,TBX21,HSDL1
Lack of natural killer cells	1.70E-02	LCK,IL2RB,IL2RG,IKZF1

Neoplastic cell transformation of B lymphocytes	1.70E-02	INPP5D,IL7R,CDKN2A,BCL2L1
Proliferation of plasma cells	1.70E-02	CD40LG,TNFAIP3,PRDM1,FAS
Size of T lymphocytes	1.70E-02	IL2,TRAF3IP3,BCL2L1,IL2RG
Psoriatic arthritis	1.81E-02	CCND3,SIGIRR,METTL9,LPIN1,STAT3,TCF7,SIPA1,RUNX3,PRKCB,IKBKB,CD27,MAP3K3
Signaling of T lymphocytes	2.12E-02	IL2,CD5,IL2RB,PTPRC,IL2RG
Transformation of B lymphocytes	2.12E-02	INPP5D,IL7R,CDKN2A,BCL2L1,JUNB
Rheumatoid arthritis	2.39E-02	RAB1B,F11R,IFNG,TNRC6B,ICOS,LCP1,IKZF1,RNF14,TMF1,CCDC59,STING1,CXCR4,FKBP5,DEDD2,HSPA8,MLLT6,TRAF1,ZNF326,EHD4,SAFB,NONO,NR4A2,S1PR1,DNAJA4,IL2,DCUN1D1,CCL5,WNK1,PRDM1,DUSP2,CD69,ADSS2,NUMB,USP15,TNFAIP3,MAP3K2,GZMA,RNF149,ADGRE5,GNLY,SOCS1,PTPRC,TBRG1,RCSD1,STK17B,TAGAP,LDHB
Development of Single positive thymocytes	2.56E-02	THEMIS,CD3G,RICTOR,IKBKB
Differentiation of naive B cells	2.56E-02	IL2,STAT3,SYK,CD40LG
Expansion of Th1 cells	0.0256	PARP1,CD40LG,ICOS,IFNG
Function of natural killer T lymphocytes	0.0256	FCER1G,HCST,SH2D1A,PRKDC

Supplementary Table 5. List of Canonical Pathways deregulated in cluster 0 compared with cluster 1.

Ingenuity Canonical Pathways	-log(p-value)	zScore	Ratio	Molecules
Insulin Secretion Signaling Pathway	1,43	-6,08	0,16	CAMK2G,CREB1,CREB3,CREBBP,EIF4A1,EIF4A2,EIF4G1,EIF4G2,EIF4G3,FYN,GOSR2,JAK1,LCK,MAPK13,MAPK14,PAIP1,PIK3C2B,PIK3C3,PIK3R1,PIK3R4,PLCB3,PRKACB,PRKAR2A,PRKCB,PRKCH,PRKCQ,PRKD3,RAP1B,RPS6KA5,SRP54,STAT3,STAT4,STAT5A,STAT5B,STX16,VAMP2,VTI1B
Thrombin Signaling	2,39	-5,57	0,19	AKT3,ARHGEF1,ARHGEF3,ARHGEF6,CAMK2G,CAMK4,CREB1,ELK1,F2R,FNBP1,GATA3,GNB1,IKBKB,KRAS,MAPK13,MAPK14,MPRIP,NRAS,PIK3C2B,PIK3C3,PIK3R1,PIK3R4,PLCB3,PPP1R12A,PRKCB,PRKCH,PRKCQ,PRKD3,RAC2,RAP1B,RAP2A,RASD1,RHOH,RHOT1,RHOT2,TBP
Phospholipase C Signaling	2,92	-5,52	0,19	AHNAK,ARHGEF1,ARHGEF3,ARHGEF6,CALM1,CAMK4,CD247,CD3D,CD3E,CD3G,CREB1,CREB3,CREBBP,EP300,FCER1G,FNBP1,FYN,GNB1,GRAP2,HDAC8,ITGA1,ITGA4,ITGAE,ITGAL,ITK,KRAS,LAT,LCK,MPRIP,NRAS,PLA2G6,PLCB3,PPP1R12A,PPP3CA,PPP3CB,PRKCB,PRKCH,PRKCQ,PRKD3,RAC2,RAP1B,RAP2A,RASD1,RHOH,RHOT1,RHOT2,SYK,TRGC2,ZAP70
Senescence Pathway	3,73	-5,46	0,20	AKT3,ANAPC1,ASXL1,ASXL2,ATM,BRAF,CALM1,CAMK4,CAPN2,CBX7,CCND2,CCND3,CDKN1B,CDKN2A,CDKN2B,CREBBP,ELF1,EP300,ETS1,GADD45G,HIPK2,IKBKB,ITSN2,KRAS,MAP2K7,MAP3K7,MAPK14,NRAS,PARP1,PCGF6,PDK3,PHF1,PIK3C2B,PIK3C3,PIK3R1,PIK3R4,PML,PPP2CA,PPP2R1B,PPP2R2B,PPP2R5A,PPP2R5C,PPP2R5D,PPP2R5E,PPP3CA,PPP3CB,RAP
Natural Killer Cell Signaling	6,95	-5,40	0,26	AKT3,CD247,CFL2,FCER1G,FYN,HCST,HLAA,HLAF,HSPA1L,HSPA6,HSPA8,IFNG,IL18R1,IL2,IL2RB,ITGAL,KLRB1,KLRC1,KLRC2,KLRD1,KRAS,LAT,LCK,MAP3K14,MAP3K2,MAP3K3,MAP3K7,MAPK13,MAPK14,NCK1,NCR3,NRAS,PAK2,PIK3C2B,PIK3C3,PIK3R1,PIK3R4,PRKCQ,PXN,RAC2,RAP1B,RAP2A,RASD1,SH2D1A,SIGLEC7,STAT4,SYK,WIPF1,ZAP70
Superpathway of Inositol Phosphate Compounds	3,29	-5,40	0,21	ACP1,DUSP10,DUSP2,DUSP4,INPP5D,IP6K1,ITPKB,MINPP1,MTMR6,NT5C,PDCD1,PI4KB,PIK3C2B,PIK3C3,PIK3R1,PIK3R4,PIKFYVE,PIP4K2A,PLCB3,PPFIBP2,PPIP5K1,PPP1R10,PPP1R12A,PPP1R16B,PPP2CA,PPP2R1B,PPP2R2B,PPP2R5A,PPP2R5C,PPP2R5D,PPP2R5E,PPP3CA,PPP3CB,PPP4R1,PRUNE1,PTPN22,PTPN7,PTPRC,PTPRO,SACM1L,SSH2,STYX
T Cell Receptor Signaling	7,40	-5,37	0,26	AKT3,CALM1,CAMK4,CD247,CD3D,CD3E,CD3G,CD8A,CD8B,CDKN1B,ELK1,FCER1G,FYB1,FYN,GRAP2,GSK3B,HLAA,HLADRB5,HLAF,ICOS,IFNG,IKBKB,IL2,ITGAL,ITK,KRAS,LAT,LCK,MALT1,MAP2K7,MAPK13,MAPK14,NCK1,NRAS,PAG1,PIK3C2B,PIK3C3,PIK3R1,PIK3R4,PPP3CA,PPP3CB,PRKCQ,PTPN22,PTPN7,PTPRC,RAP1B,RAP2A,RASD1,RASGRP1,SKAP1,TCF7,TRGC2,ZA
PKCθ Signaling in T Lymphocytes	3,02	-5,30	0,21	CAMK2G,CD247,CD3D,CD3E,CD3G,FCER1G,FYN,GRAP2,HLAA,HLADRB5,IKBKB,IL2,KRAS,LAT,LCK,MALT1,MAP3K14,MAP3K2,MAP3K3,MAP3K7,NRAS,PIK3C2B,PIK3C3,PIK3R1,PIK3R4,PPP3CA,PPP3CB,PRKCQ,RAC2,RAP1B,RAP2A,RASD1,TRGC2,ZAP70
Oxytocin Signaling Pathway	1,46	-5,19	0,17	AKT3,CALM1,CAMK4,CCL5,CREB1,CREB3,CREBBP,GNB1,GSK3B,KRAS,MAPK13,MAPK14,MPRIP,MYH9,NRAS,NT5C,PIK3C2B,PIK3C3,PIK3R1,PIK3R4,PLA2G6,PLCB3,PNPLA2,PPARD,PPP1R12A,PPP3CA,PPP3CB,PRKAA1,PRKACB,PRKAR2A,PRKCB,PRKCH,PRKCQ,PRKD3,RAP1B,RAP2A,RASD1,SMARCC2
Integrin Signaling	1,72	-5,11	0,18	ACTN4,AKT3,ARPC5L,BRAF,CAPN2,FNBP1,FYN,GSK3B,ITGA1,ITGA4,ITGAE,ITGAL,KRAS,MPRIP,NCK1,NRAS,PAK2,PIK3C2B,PIK3C3,PIK3R1,PIK3R4,PIKFYVE,PPP1R12A,PXN,RAC2,RAP1B,RAP2A,RASD1,RHOH,RHOT1,RHOT2,TLN1,WIPF1
GNRH Signaling	1,52	-5,10	0,18	CALM1,CAMK2G,CAMK4,CREB1,CREB3,CREBBP,ELK1,GNB1,KRAS,MAP2K7,MAP3K14,MAP3K2,MAP3K3,MAP3K7,MAPK13,MAPK14,NRAS,PAK2,PLCB3,PRKACB,PRKAR2A,PRKCB,PRKCH,PRKCQ,PRKD3,PXN,RAP1B,RAP2A,RASD1
Systemic Lupus Erythematosus In B Cell Signaling Pathway	3,17	-5,09	0,19	AKT3,BAD,BCL2,BCL2L1,CALM1,CAMK4,CCND2,CCND3,CD40LG,CD5,FYN,GSK3B,IFNG,IL2,INPP5D,IRF9,ISG15,ISG20,JAK1,KRAS,LCK,LTB,MALT1,MAP3K14,MAP3K7,NRAS,PAG1,PDCD1,PIK3AP1,PIK3C2B,PIK3C3,PIK3R1,PIK3R4,PLAAT4,PPP3CA,PPP3CB,PRKCB,PRKCH,PRKCQ,PRKD3,RAC2,RAP1B,RAP2A,RASD1,RASGRP1,STAT3,STING1,SYK,TNFSF14,TRAF1
3-phosphoinositide Biosynthesis	3,46	-5,01	0,21	ACP1,DUSP10,DUSP2,DUSP4,INPP5D,MINPP1,MTMR6,NT5C,PDCD1,PI4KB,PIK3C2B,PIK3C3,PIK3R1,PIK3R4,PIKFYVE,PIP4K2A,PPFIBP2,PPIP5K1,PPP1R10,PPP1R12A,PPP1R16B,PPP2CA,PPP2R1B,PPP2R2B,PPP2R5A,PPP2R5C,PPP2R5D,PPP2R5E,PPP3CA,PPP3CB,PPP4R1,PRUNE1,PTPN22,PTPN7,PTPRC,PTPRO,SACM1L,SSH2,STYX
ICOS-ICOSL Signaling in T Helper Cells	6,08	-4,81	0,29	AKT3,BAD,CALM1,CAMK2G,CAMK4,CD247,CD3D,CD3E,CD3G,CD40LG,FCER1G,GRAP2,HLAA,HLADRB5,ICOS,IKBKB,IL2,IL2RB,IL2RG,INPP5D,ITK,LAT,LCK,PIK3C2B,PIK3C3,PIK3R1,PIK3R4,PLEKHA2,PPP3CA,PPP3CB,PRKCQ,PTPRC,TRAT1,TRGC2,ZAP70
NF-κB Signaling	3,77	-4,80	0,22	AKT3,AZI2,BRAF,CASP8,CD247,CD3D,CD3E,CD3G,CD40LG,CREBBP,EIF2AK2,EP300,FCER1G,GSK3B,IKBKB,KRAS,LCK,MALT1,MAP2K7,MAP3K14,MAP3K3,MAP3K7,NRAS,PIK3C2B,PIK3C3,PIK3R1,PIK3R4,PRKACB,PRKCB,PRKCQ,RAP1B,RAP2A,RASD1,SIGIRR,TAB2,TNFAIP3,TNIP1,TRGC2,UBE2V1,ZAP70
CXCR4 Signaling	1,38	-4,80	0,18	AKT3,CXCR4,ELK1,FNBP1,GNB1,KRAS,NRAS,PAK2,PIK3C2B,PIK3C3,PIK3R1,PIK3R4,PLCB3,PRKCB,PRKCH,PRKCQ,PRKD3,PXN,RAC2,RAP1B,RAP2A,RASD1,RHOH,RHOT1,RHOT2
IL-8 Signaling	1,43	-4,75	0,17	AKT3,ANGPT2,BAX,BCL2,BCL2L1,BRAF,CCND2,CCND3,FNBP1,GNB1,IKBKB,IQGAP1,KRAS,NCF1,NCF2,NRAS,PAK2,PIK3C2B,PIK3C3,PIK3R1,PIK3R4,PRKCB,PRKCH,PRKCQ,PRKD3,RAC2,RAP1B,RAP2A,RASD1,RHOH,RHOT1,RHOT2
HMGB1 Signaling	1,53	-4,69	0,18	AKT3,CD40LG,ELK1,FNBP1,HMGB1,IFNG,IL2,KAT6A,KAT7,KRAS,LTB,MAP2K7,MAPK13,MAPK14,NRAS,PIK3C2B,PIK3C3,PIK3R1,PIK3R4,RAC2,RAP1B,RAP2A,RASD1,RHOH,RHOT1,RHOT2,TNFSF14
CD28 Signaling in T Helper Cells	3,22	-4,69	0,23	AKT3,ARPC5L,CALM1,CAMK4,CD247,CD3D,CD3E,CD3G,FCER1G,FYN,GRAP2,HLAA,HLADRB5,IKBKB,IL2,ITK,LAT,LCK,MALT1,PIK3C2B,PIK3C3,PIK3R1,PIK3R4,PPP3CA,PPP3CB,PRKCQ,PTPRC,SYK,TRGC2,ZAP70
B Cell Receptor Signaling	3,72	-4,64	0,22	AKT3,BAD,BCL2L1,CALM1,CAMK2G,CAMK4,CFL2,CREB1,CREB3,CREBBP,ELK1,ETS1,GSK3B,IKBKB,INPP5D,KRAS,MALT1,MAP2K7,MAP3K14,MAP3K2,MAP3K3,MAP3K7,MAPK13,MAPK14,NRAS,PAG1,PIK3AP1,PIK3C2B,PIK3C3,PIK3R1,PIK3R4,PPP3CA,PPP3CB,PRKCB,PRKCQ,PTPRC,RAC2,RAP1B,RAP2A,RASD1,SYK
TEC Kinase Signaling	2,37	-4,60	0,19	CD247,CD3D,CD3E,CD3G,FAS,FCER1G,FNBP1,FYN,GNB1,GTF2I,ITGA1,ITGA4,ITGAE,ITGAL,ITK,JAK1,LCK,PAK2,PIK3C2B,PIK3C3,PIK3R1,PIK3R4,PRKCB,PRKCH,PRKCQ,PRKD3,RAC2,RHOH,RHOT1,RHOT2,STAT3,STAT4,STAT5A,STAT5B,TRGC2
3-phosphoinositide Degradation	2,60	-4,60	0,20	ACP1,DUSP10,DUSP2,DUSP4,INPP4B,INPP5D,MINPP1,MTMR6,NT5C,PDCD1,PIKFYVE,PPFIBP2,PPIP5K1,PPP1R10,PPP1R12A,PPP1R16B,PPP2CA,PPP2R1B,PPP2R2B,PPP2R5A,PPP2R5C,PPP2R5D,PPP2R5E,PPP3CA,PPP3CB,PPP4R1,PRUNE1,PTPN22,PTPN7,PTPRC,PTPRO,SACM1L,SSH2,STYX
D-myo-inositol-5-phosphate Metabolism	2,79	-4,60	0,20	ACP1,DUSP10,DUSP2,DUSP4,INPP5D,MINPP1,MTMR6,NT5C,PDCD1,PIKFYVE,PIP4K2A,PLCB3,PPFIBP2,PPIP5K1,PPP1R10,PPP1R12A,PPP1R16B,PPP2CA,PPP2R1B,PPP2R2B,PPP2R5A,PPP2R5C,PPP2R5D,PPP2R5E,PPP3CA,PPP3CB,PPP4R1,PRUNE1,PTPN22,PTPN7,PTPRC,PTPRO,SACM1L,SSH2,STYX
NAD Signaling Pathway	2,83	-4,60	0,22	AKT3,ERN1,ETFA,GSK3B,H1-10,H1-2,H1-3,H1-4,LDHB,NMNAT1,NMRK1,PARP1,PARP10,PARP4,PARP6,PARP8,PIK3C2B,PIK3C3,PIK3R1,PIK3R4,POLR2F,POLR2H,POLR2I,PRKAA1,SIRT3,SLC36A4,TIPARP,TNKS2
Role of NFAT in Regulation of the Immune Response	3,27	-4,60	0,21	AKT3,CABIN1,CALM1,CAMK4,CD247,CD3D,CD3E,CD3G,CSNK1G3,FCER1G,FYN,GNB1,GSK3B,HLAA,HLADRB5,IKBKB,ITK,KPNA1,KPNA4,KPNA5,KPNA6,KRAS,LAT,LCK,NRAS,PIK3C2B,PIK3C3,PIK3R1,PIK3R4,PLCB3,PPP3CA,PPP3CB,PRKCQ,RAP1B,RAP2A,RASD1,SYK,TNPO1,TRGC2,ZAP70
NGF Signaling	1,62	-4,58	0,19	AKT3,BAX,CREB1,CREB3,CREBBP,ELK1,IKBKB,KRAS,MAP3K14,MAP3K2,MAP3K3,MAP3K7,NRAS,PIK3C2B,PIK3C3,PIK3R1,PIK3R4,RAP1B,RAP2A,RASD1,RPS6KA5,SMPD1
IL-15 Signaling	2,68	-4,58	0,24	AKT3,BCL2L1,IL2RB,IL2RG,JAK1,KRAS,LCK,MAPK13,MAPK14,NRAS,PIK3C2B,PIK3C3,PIK3R1,PIK3R4,RAP1B,RAP2A,RASD1,STAT3,STAT5A,STAT5B,SYK
LPS-stimulated MAPK Signaling	3,16	-4,47	0,26	ATF1,CREB1,ELK1,IKBKB,KRAS,MAP3K14,MAP3K7,MAPK13,MAPK14,NRAS,PIK3C2B,PIK3C3,PIK3R1,PIK3R4,PRKCB,PRKCH,PRKCQ,PRKD3,RAP1B,RAP2A,RASD1
Autophagy	2,08	-4,44	0,18	AKT3,ATG12,ATG5,ATM,BAD,BCL2,BIRC6,CALM1,CAMK4,CDKN1B,CREB1,CREB3,CREBBP,EPG5,ERN1,GORASP2,GSK3B,MAP2K7,OPTN,PIK3C2B,PIK3C3,PIK3R1,PIK3R4,PPP2CA,PPP2R1B,PPP2R2B,PPP2R5A,PPP2R5C,PPP2R5D,PPP2R5E,PPP3CA,PPP3CB,PRKAA1,PRKACB,PRKAR2A,PRKCQ,VPS11
D-myo-inositol (1,4,5,6)-Tetrakisphosphate Biosynthesis	2,81	-4,38	0,21	ACP1,DUSP10,DUSP2,DUSP4,INPP5D,MINPP1,MTMR6,NT5C,PDCD1,PIKFYVE,PPFIBP2,PPIP5K1,PPP1R10,PPP1R12A,PPP1R16B,PPP2CA,PPP2R1B,PPP2R2B,PPP2R5A,PPP2R5C,PPP2R5D,PPP2R5E,PPP3CA,PPP3CB,PPP4R1,PRUNE1,PTPN22,PTPN7,PTPRC,PTPRO,SACM1L,SSH2,STYX
D-myo-inositol (3,4,5,6)-tetrakisphosphate Biosynthesis	2,81	-4,38	0,21	ACP1,DUSP10,DUSP2,DUSP4,INPP5D,MINPP1,MTMR6,NT5C,PDCD1,PIKFYVE,PPFIBP2,PPIP5K1,PPP1R10,PPP1R12A,PPP1R16B,PPP2CA,PPP2R1B,PPP2R2B,PPP2R5A,PPP2R5C,PPP2R5D,PPP2R5E,PPP3CA,PPP3CB,PPP4R1,PRUNE1,PTPN22,PTPN7,PTPRC,PTPRO,SACM1L,SSH2,STYX
HGF Signaling	3,34	-4,38	0,23	AKT3,CDKN2A,ELF1,ELK1,ETS1,ITGA1,ITGA4,ITGAE,ITGAL,KRAS,MAP2K7,MAP3K14,MAP3K2,MAP3K3,MAP3K7,NRAS,PIK3C2B,PIK3C3,PIK3R1,PIK3R4,PRKCB,PRKCH,PRKCQ,PRKD3,PXN,RAP1B,RAP2A,RASD1,STAT3
Fcγ Receptor-mediated Phagocytosis in Macrophages and Monocytes	1,88	-4,36	0,21	AKT3,ARPC5L,EZR,FYB1,FYN,INPP5D,NCF1,NCK1,PIK3R1,PLA2G6,PRKCB,PRKCH,PRKCQ,PRKD3,PXN,RAB11B,RAC2,SYK,TLN1
Renin-Angiotensin Signaling	1,92	-4,36	0,20	CCL5,ELK1,KRAS,MAPK13,MAPK14,NRAS,PAK2,PIK3C2B,PIK3C3,PIK3R1,PIK3R4,PRKACB,PRKAR2A,PRKCB,PRKCH,PRKCQ,PRKD3,PTGER2,RAP1B,RAP2A,RASD1,STAT3
RANK Signaling in Osteoclasts	1,93	-4,36	0,21	AKT3,CALM1 (includes others),CAMK4,ELK1,IKBKB,MAP2K7,MAP3K14,MAP3K2,MAP3K3,MAP3K7,MAPK13,MAPK14,PIK3C2B,PIK3C3,PIK3R1,PIK3R4,PPP3CA,PPP3CB,TAB2
Signaling by Rho Family GTPases	1,33	-4,27	0,16	ARHGEF1,ARHGEF3,ARHGEF6,ARPC5L,CDC42EP3,CFL2,ELK1,EZR,FNBP1,GNB1,IQGAP1,ITGA1,ITGA4,ITGAE,ITGAL,MAP2K7,MSN,NCF1,NCF2,PAK2,PIK3C2B,PIK3C3,PIK3R1,PIK3R4,PIKFYVE,PIP4K2A,PPP1R12A,RAC2,RHOH,RHOT1,RHOT2,SEPTIN1,SEPTIN6,SEPTIN7,SEPTIN9,WIPF1
fMLP Signaling in Neutrophils	1,87	-4,26	0,20	ARPC5L,CALM1,CAMK4,ELK1,GNB1,KRAS,NCF1,NCF2,NRAS,PIK3C2B,PIK3C3,PIK3R1,PIK3R4,PLCB3,PPP3CA,PPP3CB,PRKCB,PRKCH,PRKCQ,PRKD3,RAC2,RAP1B,RAP2A,RASD1
NF-κB Activation by Viruses	2,86	-4,24	0,25	AKT3,EIF2AK2,IKBKB,KRAS,LCK,MAP3K14,NRAS,PIK3C2B,PIK3C3,PIK3R1,PIK3R4,PRKCB,PRKCH,PRKCQ,PRKD3,RAP1B,RAP2A,RASD1,TBP
PI3K Signaling in B Lymphocytes	2,27	-4,20	0,20	AKT3,ATF1,ATF6,ATF6B,CALM1,CAMK2G,CAMK4,CREB1,ELK1,FYN,IKBKB,INPP5D,KRAS,MALT1,NRAS,PIK3AP1,PIK3R1,PLCB3,PLEKHA2,PPP3CA,PPP3CB,PRKCB,PTPRC,RAP1B,RAP2A,RASD1,SYK
RAC Signaling	2,61	-4,15	0,21	ARPC5L,CD44,CFL2,CYFIP2,ELK1,ELK4,IQGAP1,ITGA1,ITGA4,ITGAE,ITGAL,KRAS,MAP2K7,NCF1,NCF2,NRAS,PAK2,PIK3C2B,PIK3C3,PIK3R1,PIK3R4,PIKFYVE,PIP4K2A,RAC2,RAP1B,RAP2A,RASD1
Paxillin Signaling	2,10	-4,12	0,21	ACTN4,ARHGEF6,ITGA1,ITGA4,ITGAE,ITGAL,KRAS,MAPK13,MAPK14,NCK1,NRAS,PAK2,PIK3C2B,PIK3C3,PIK3R1,PIK3R4,PXN,RAP1B,RAP2A,RASD1,TLN1
CCR3 Signaling in Eosinophils	2,14	-4,12	0,21	CALM1,CAMK4,CFL2,GNB1,KRAS,MAPK13,MAPK14,MPRIP,NRAS,PAK2,PIK3C2B,PIK3C3,PIK3R1,PIK3R4,PLA2G6,PLCB3,PPP1R12A,PRKCB,PRKCH,PRKCQ,PRKD3,RAP1B,RAP2A,RASD1
Dopamine-DARPP32 Feedback in cAMP Signaling	1,50	-4,08	0,18	ATF1,ATP2A2,CALM1,CAMK4,CREB1,CREB3,CREBBP,CREM,CSNK1G3,PLCB3,PPP1R10,PPP1R12A,PPP2CA,PPP2R1B,PPP2R2B,PPP2R5A,PPP2R5C,PPP2R5D,PPP2R5E,PPP3CA,PPP3CB,PRKACB,PRKAR2A,PRKCB,PRKCH,PRKCQ,PRKD3
Regulation of IL-2 Expression in Activated and Anergic T Lymphocytes	4,00	-4,04	0,26	CALM1,CAMK4,CD247,CD3D,CD3E,CD3G,ELK1,FCER1G,FYN,IKBKB,IL2,KRAS,LAT,LCK,MALT1,MAP2K7,NRAS,PABPC4,PPP3CA,PPP3CB,PRKCQ,RAP1B,RAP2A,RASD1,SMAD2,TOB1,ZAP70
Leukocyte Extravasation Signaling	1,82	-4,02	0,18	ACTN4,ARHGAP1,CD44,CD99,CXCR4,CYBA,EZR,F11R,ITGA4,ITGAL,ITK,MAPK13,MAPK14,MMP7,MSN,NCF1,NCF2,PIK3C2B,PIK3C3,PIK3R1,PIK3R4,PRKCB,PRKCH,PRKCQ,PRKD3,PXN,RAC2,RAP1B,RASGRP1,RHOH,SIPA1,WIPF1
Melanocyte Development and Pigmentation Signaling	1,31	-4,00	0,19	BCL2,CREB1,CREB3,CREBBP,EP300,KRAS,NRAS,PIK3C2B,PIK3C3,PIK3R1,PIK3R4,PRKACB,PRKAR2A,RAP1B,RAP2A,RASD1,RPS6KA5
Glioma Signaling	2,09	-4,00	0,20	AKT3,CALM1,CAMK2G,CAMK4,CCND2,CCND3,CDKN2A,CDKN2B,HDAC8,KRAS,NRAS,PIK3C2B,PIK3C3,PIK3R1,PIK3R4,PRKCB,PRKCH,PRKCQ,PRKD3,RAP1B,RAP2A,RASD1,RBL2,SIN3A
Fc Epsilon RI Signaling	2,83	-3,96	0,23	AKT3,FCER1G,FYN,GRAP2,INPP5D,KRAS,LAT,MAP2K7,MAPK13,MAPK14,NRAS,PIK3C2B,PIK3C3,PIK3R1,PIK3R4,PLA2G6,PRKCB,PRKCH,PRKCQ,PRKD3,RAC2,RAP1B,RAP2A,RASD1,SYK
FLT3 Signaling in Hematopoietic Progenitor Cells	4,83	-3,96	0,30	AKT3,BAD,CREB1,CREB3,CREBBP,ELK1,FLT3LG,INPP5D,KRAS,MAPK13,MAPK14,NRAS,PIK3C2B,PIK3C3,PIK3R1,PIK3R4,RAP1B,RAP2A,RASD1,RPS6KA5,STAT3,STAT4,STAT5A,STAT5B
Th1 Pathway	4,08	-3,92	0,25	CD247,CD3D,CD3E,CD3G,CD40LG,CD8A,GATA3,HLA-A,HLA-DRB5,ICOS,IFNG,IL18R1,IL2,IRF1,JAK1,KLRC1,KLRD1,NCSTN,NOTCH1,PIK3C2B,PIK3C3,PIK3R1,PIK3R4,PRKCQ,RUNX3,SOCS1,STAT3,STAT4,TBX21
Death Receptor Signaling	2,26	-3,90	0,22	BCL2,CASP10,CASP3,CASP6,CASP8,DAXX,DFFA,FAS,HTRA2,IKBKB,MAP2K7,MAP3K14,PARP1,PARP10,PARP4,PARP6,PARP8,SPTAN1,TIPARP,TNKS2
IL-2 Signaling	4,90	-3,90	0,34	AKT3,ELK1,IL2,IL2RB,IL2RG,JAK1,KRAS,LCK,NRAS,PIK3C2B,PIK3C3,PIK3R1,PIK3R4,RAP1B,RAP2A,RASD1,SOCS1,STAT5A,STAT5B,SYK
Estrogen-Dependent Breast Cancer Signaling	2,03	-3,87	0,23	AKT3,CREB1,CREB3,CREBBP,ELK1,KRAS,NRAS,PIK3C2B,PIK3C3,PIK3R1,PIK3R4,RAP1B,RAP2A,RASD1,STAT5A,STAT5B
Thrombopoietin Signaling	2,74	-3,87	0,27	KRAS,NRAS,PIK3C2B,PIK3C3,PIK3R1,PIK3R4,PRKCB,PRKCH,PRKCQ,PRKD3,RAP1B,RAP2A,RASD1,STAT3,STAT5A,STAT5B
Spliceosomal Cycle	4,33	-3,87	0,36	BCAS2,CASC3,CWC25,DDX23,DDX39B,DHX16,HSPA8,MAGOHB,PRPF18,SF1,SF3A3,SF3B1,SF3B3,SNRNP200,U2AF1/U2AF1L5,U2AF1L4
IL-3 Signaling	4,62	-3,84	0,30	AKT3,BAD,ELK1,INPP5D,JAK1,KRAS,NRAS,PIK3C2B,PIK3C3,PIK3R1,PIK3R4,PPP3CA,PPP3CB,PRKCB,PRKCH,PRKCQ,PRKD3,RAP1B,RAP2A,RASD1,STAT3,STAT5A,STAT5B
HIF1α Signaling	1,96	-3,77	0,18	AKT3,BRAF,CAMK2G,CAMK4,CREBBP,CUL2,EP300,GPI,HSP90AA1,HSPA1L,HSPA6,HSPA8,KRAS,LDHB,MAP2K7,MMP7,NCF1,NCF2,NRAS,PIK3C2B,PIK3C3,PIK3R1,PIK3R4,PPP3CA,PPP3CB,PRKCB,PRKCH,PRKCQ,PRKD3,RAC2,RAP1B,RAP2A,RASD1,RBX1,STAT3
ERBB Signaling	2,20	-3,77	0,22	ELK1,GSK3B,KRAS,MAPK13,MAPK14,NCK1,NRAS,PAK2,PIK3C2B,PIK3C3,PIK3R1,PIK3R4,PRKCB,PRKCH,PRKCQ,PRKD3,RAP1B,RAP2A,RASD1
VEGF Family Ligand-Receptor Interactions	1,35	-3,74	0,20	AKT3,KRAS,NRAS,PIK3C2B,PIK3C3,PIK3R1,PIK3R4,PLA2G6,PRKCB,PRKCH,PRKCQ,PRKD3,RAP1B,RAP2A,RASD1
PAK Signaling	1,44	-3,74	0,19	ARHGEF6,CASP3,CFL2,ITGA1,ITGA4,ITGAE,ITGAL,KRAS,NCK1,NRAS,PAK2,PIK3C2B,PIK3C3,PIK3R1,PIK3R4,PXN,RAP1B,RAP2A,RASD1
Synaptic Long Term Potentiation	1,85	-3,71	0,20	CALM1,CAMK2G,CAMK4,CREB1,CREB3,CREBBP,EP300,KRAS,NRAS,PLCB3,PPP1R10,PPP1R12A,PPP3CA,PPP3CB,PRKACB,PRKAR2A,PRKCB,PRKCH,PRKCQ,PRKD3,RAP1B,RAP2A,RASD1
Endocannabinoid Developing Neuron Pathway	2,09	-3,71	0,20	AKT3,BRAF,CDKN1B,CREB1,CREB3,CREBBP,GNB1,GSK3B,KRAS,MAP2K7,MAPK13,MAPK14,NRAS,PIK3C2B,PIK3C3,PIK3R1,PIK3R4,PRKACB,PRKAR2A,RAC2,RAP1B,RAP2A,RASD1,STAT3
Crosstalk between Dendritic Cells and Natural Killer Cells	1,56	-3,61	0,20	CAMK2G,CD40LG,CD69,FAS,HLA-A,HLA-DRB5,HLA-F,IFNG,IL2,IL2RB,IL2RG,ITGAL,KLRD1,LTB,NCR3,PRF1,TLN1
PEDF Signaling	3,24	-3,58	0,26	AKT3,BCL2,BCL2L1,CASP8,ELK1,FAS,IKBKB,KRAS,MAPK13,MAPK14,NRAS,PIK3C2B,PIK3C3,PIK3R1,PIK3R4,PNPLA2,RAP1B,RAP2A,RASD1,TCF7,ZEB1
Prolactin Signaling	4,05	-3,58	0,28	CREBBP,EP300,FYN,IRF1,KRAS,NRAS,PIK3C2B,PIK3C3,PIK3R1,PIK3R4,PRKCB,PRKCH,PRKCQ,PRKD3,RAP1B,RAP2A,RASD1,SOCS1,SOCS2,STAT3,STAT5A,STAT5B,TCF7
Th2 Pathway	3,39	-3,53	0,23	CD247,CD3D,CD3E,CD3G,CXCR4,CXCR6,GATA3,HLA-A,HLA-DRB5,ICOS,IFNG,IKZF1,IL2,IL2RB,IL2RG,JAK1,MAF,NCSTN,NOTCH1,PIK3C2B,PIK3C3,PIK3R1,PIK3R4,PRKCQ,RUNX3,S1PR1,STAT4,STAT5A,STAT5B,TBX21
Actin Cytoskeleton Signaling	1,54	-3,53	0,17	ACTN4,ARHGEF1,ARHGEF6,ARPC5L,CFL2,CYFIP2,DIAPH2,EZR,F2R,FGD3,IQGAP1,ITGA1,ITGA4,ITGAE,ITGAL,KRAS,MATK,MPRIP,MSN,MYH9,NRAS,PAK2,PIK3C2B,PIK3C3,PIK3R1,PIK3R4,PPP1R12A,PXN,RAC2,RAP1B,RAP2A,RASD1,SSH2,TLN1
Xenobiotic Metabolism General Signaling Pathway	4,27	-3,53	0,26	AKT3,CREBBP,EP300,FTL,GSTP1,KRAS,MAF,MAP2K7,MAP3K14,MAP3K2,MAP3K3,MAP3K7,MAPK13,MAPK14,MGST1,MGST2,NR1H2,NRAS,PIK3C2B,PIK3C3,PIK3R1,PIK3R4,PRKCB,PRKCH,PRKCQ,PRKD3,RAP1B,RAP2A,RASD1,RBX1
GM-CSF Signaling	3,91	-3,50	0,29	AKT3,BCL2L1,CAMK2G,ELK1,ETS1,KRAS,NRAS,PIK3C2B,PIK3C3,PIK3R1,PIK3R4,PIM1,PPP3CA,PPP3CB,PRKCB,RAP1B,RAP2A,RASD1,STAT3,STAT5B
Aldosterone Signaling in Epithelial Cells	1,48	-3,46	0,18	DNAJA1,DNAJB1,DNAJB12,DNAJB6,DNAJC17,DNAJC21,DNAJC3,DNAJC7,HSP90AA1,HSPA1L,HSPA6,HSPA8,HSPH1,KRAS,PIK3C2B,PIK3C3,PIK3R1,PIK3R4,PIKFYVE,PIP4K2A,PLCB3,PRKCB,PRKCH,PRKCQ,PRKD3
ERBB4 Signaling	1,86	-3,46	0,23	KRAS,NCSTN,NRAS,PIK3C2B,PIK3C3,PIK3R1,PIK3R4,PRKCB,PRKCH,PRKCQ,PRKD3,RAP1B,RAP2A,RASD1
UVB-Induced MAPK Signaling	1,95	-3,46	0,25	BAD,MAPK13,MAPK14,PIK3C2B,PIK3C3,PIK3R1,PIK3R4,PRKCB,PRKCH,PRKCQ,PRKD3,RPS6KA5
UVC-Induced MAPK Signaling	2,30	-3,46	0,27	BRAF,KRAS,MAPK13,MAPK14,NRAS,PRKCB,PRKCH,PRKCQ,PRKD3,RAP1B,RAP2A,RASD1,SMPD1
GP6 Signaling Pathway	2,22	-3,44	0,21	AKT3,CALM1 (includes others),CAMK4,CERT1,FCER1G,FYB1,FYN,GRAP2,GSK3B,ITK,LAT,NCK1,PIK3C2B,PIK3C3,PIK3R1,PIK3R4,PRKCB,PRKCH,PRKCQ,PRKD3,RAP1B,SYK,TLN1
Calcium-induced T Lymphocyte Apoptosis	4,22	-3,44	0,29	ATP2A2,CABIN1,CALM1,CAMK4,CAPN2,CD247,CD3D,CD3E,CD3G,EP300,FCER1G,HLA-A,HLA-DRB5,LCK,PPP3CA,PPP3CB,PRKCB,PRKCH,PRKCQ,PRKD3,TRGC2,ZAP70
PDGF Signaling	1,50	-3,36	0,20	ACP1,EIF2AK2,ELK1,INPP5D,JAK1,KRAS,NRAS,PIK3C2B,PIK3C3,PIK3R1,PIK3R4,PRKCB,RAP1B,RAP2A,RASD1,STAT3
Thyroid Cancer Signaling	1,58	-3,36	0,21	AKT3,BRAF,CXCR4,ELK1,GSK3B,KRAS,NRAS,PIK3C2B,PIK3C3,PIK3R1,PIK3R4,RAP1B,RAP2A,RASD1,TCF7
CD40 Signaling	1,97	-3,36	0,23	ATF1,CD40LG,IKBKB,MAP2K7,MAP3K14,MAP3K7,MAPK13,MAPK14,PIK3C2B,PIK3C3,PIK3R1,PIK3R4,STAT3,TNFAIP3,TRAF1
ERK5 Signaling	2,27	-3,36	0,24	BAD,CREB1,CREB3,CREBBP,ELK4,KRAS,MAP3K2,MAP3K3,NRAS,RAP1B,RAP2A,RASD1,RPS6KA5,SH2D2A,WNK1,YWHAQ,YWHAZ
Glioma Invasiveness Signaling	2,70	-3,36	0,26	CD44,F2R,FNBP1,KRAS,NRAS,PIK3C2B,PIK3C3,PIK3R1,PIK3R4,PLAU,RAC2,RAP1B,RAP2A,RASD1,RHOH,RHOT1,RHOT2
Retinoic acid Mediated Apoptosis Signaling	1,49	-3,32	0,22	CASP3,CASP8,IFNG,IRF1,PARP1,PARP10,PARP4,PARP6,PARP8,TIPARP,TNKS2
CNTF Signaling	1,62	-3,32	0,23	JAK1,KRAS,NRAS,PIK3C2B,PIK3C3,PIK3R1,PIK3R4,RAP1B,RAP2A,RASD1,RPS6KA5,STAT3
Renal Cell Carcinoma Signaling	2,15	-3,32	0,23	AKT3,CREBBP,CUL2,EP300,ETS1,KRAS,NRAS,PAK2,PIK3C2B,PIK3C3,PIK3R1,PIK3R4,RAP1B,RAP2A,RASD1,RBX1,UBC
Lymphotoxin β Receptor Signaling	2,42	-3,32	0,26	AKT3,BCL2L1,CASP3,CREBBP,EP300,IKBKB,LTB,MAP3K14,PIK3C2B,PIK3C3,PIK3R1,PIK3R4,TNFSF14,TRAF1
SAPK/JNK Signaling	3,05	-3,27	0,23	CD247,CD3D,CD3E,CD3G,DAXX,DUSP10,DUSP4,ELK1,FCER1G,GNB1,KRAS,LCK,MAP2K7,MAP3K2,MAP3K3,MAP3K7,NRAS,PIK3C2B,PIK3C3,PIK3R1,PIK3R4,RAC2,RAP1B,RAP2A,RASD1,SH2D2A,TRGC2
UVA-Induced MAPK Signaling	3,74	-3,16	0,26	ATM,BCL2L1,CASP3,KRAS,MAPK13,MAPK14,NRAS,PARP1,PARP10,PARP4,PARP6,PARP8,PIK3C2B,PIK3C3,PIK3R1,PIK3R4,PLCB3,RAP1B,RAP2A,RASD1,RPS6KA5,SMPD1,TIPARP,TNKS2
Mouse Embryonic Stem Cell Pluripotency	1,60	-3,15	0,20	AKT3,CREBBP,DVL3,GSK3B,JAK1,KRAS,MAP3K7,MAPK13,MAPK14,NRAS,PIK3C2B,PIK3C3,PIK3R1,PIK3R4,RAP1B,RAP2A,RASD1,STAT3,TCF7
Acute Myeloid Leukemia Signaling	2,38	-3,15	0,23	AKT3,BAD,BRAF,FLT3LG,KRAS,MAP2K7,NRAS,PIK3C2B,PIK3C3,PIK3R1,PIK3R4,PIM1,PML,RAP1B,RAP2A,RASD1,STAT3,STAT5A,STAT5B,TCF7
14-3-3-mediated Signaling	2,77	-3,13	0,23	AKT3,BAD,BAX,CDKN1B,ELK1,GSK3B,KRAS,NRAS,PDCD6IP,PIK3C2B,PIK3C3,PIK3R1,PIK3R4,PLCB3,PRKCB,PRKCH,PRKCQ,PRKD3,RAP1B,RAP2A,RASD1,STRADA,TUBA4A,YWHAQ,YWHAZ
Macropinocytosis Signaling	1,74	-3,05	0,22	ACTN4,KRAS,NRAS,PIK3C2B,PIK3C3,PIK3R1,PIK3R4,PRKCB,PRKCH,PRKCQ,PRKD3,RAB34,RAP1B,RAP2A,RASD1
IL-9 Signaling	2,22	-3,00	0,29	IL2RG,JAK1,PIK3C2B,PIK3C3,PIK3R1,PIK3R4,SOCS2,STAT3,STAT5A,STAT5B

Role of MAPK Signaling in Promoting the Pathogenesis of Influenza	1.32	-2.98	0.18	ATP6AP1,ATP6V0D1,ATP6V0E2,BAX,BCL2,BRAF,CASP3,KRAS,MAP2K7,MAPK13,MAPK14,NRAS,NUP153,PLA2G6,PLAAT4,PNPLA8,RAP1B,RAP2A,RASD1
NRF2-mediated Oxidative Stress Response	3.78	-2.98	0.22	ABCC1,BACH1,CREBBP,DNAJA1,DNAJA3,DNAJA4,DNAJB1,DNAJB12,DNAJB6,DNAJC17,DNAJC21,DNAJC3,DNAJC7,EP300,FKBP5,FTL,GSK3B,GSTP1,HSP90AA1,JUNB,JUND,KRAS,MAF,MAP2K7,MAP3K7,MAPK14,MGST1,MGST2,NRAS,PIK3C2B,PIK3C3,PIK3R1,PIK3R4,PRKCB,PRKCH,PRKCQ,PRKD3,RAP1B,RAP2A,RASD1,RBX1,UBE2E3
Melatonin Signaling	1.31	-2.89	0.20	BRAF,CALM1,CAMK2G,CAMK4,MAP2K7,PLCB3,PRKACB,PRKAR2A,PRKCB,PRKCH,PRKCQ,PRKD3,RORA
Antiproliferative Role of Somatostatin Receptor 2	1.80	-2.89	0.22	BRAF,CDKN1B,ELK1,GNB1,KRAS,NRAS,NT5C,PIK3C2B,PIK3C3,PIK3R1,PIK3R4,RAP1B,RAP2A,RASD1,SMARCC2
Chemokine Signaling	1.80	-2.84	0.22	CALM1,CAMK2G,CAMK4,CCL5,CXCR4,KRAS,MAPK13,MAPK14,MPRIP,NRAS,PLCB3,PPP1R12A,PRKCB,RAP1B,RAP2A,RASD1
Growth Hormone Signaling	2.04	-2.84	0.23	ELK1,PIK3C2B,PIK3C3,PIK3R1,PIK3R4,PRKCB,PRKCH,PRKCQ,PRKD3,RPS6KA5,SOCS1,SOCS2,STAT3,STAT5A,STAT5B
IGF-1 Signaling	2.10	-2.83	0.21	AKT3,BAD,ELK1,JAK1,KRAS,NRAS,PIK3C2B,PIK3C3,PIK3R1,PIK3R4,PRKACB,PRKAR2A,PXN,RAP1B,RAP2A,RASD1,SOCS1,SOCS2,STAT3,YWHAQ,YWHAZ
JAK/STAT Signaling	2.51	-2.83	0.24	AKT3,BCL2L1,JAK1,KRAS,NRAS,PIAS1,PIK3C2B,PIK3C3,PIK3R1,PIK3R4,RAP1B,RAP2A,RASD1,SOCS1,SOCS2,STAT3,STAT4,STAT5A,STAT5B
Protein Kinase A Signaling	1.98	-2.83	0.17	ACP1,ADD3,AKAP11,AKAP13,AKAP7,AKAP9,ANAPC1,ATF1,BAD,BRAF,CALM1,CAMK2G,CAMK4,CREB1,CREB3,CREBBP,CREM,DUSP10,DUSP2,DUSP4,ELK1,GNB1,GSK3B,H1-10,H1-2,H1-3,H1-
ERK/MAPK Signaling	5.33	-2.72	0.24	ATF1,BAD,BRAF,CREB1,CREB3,CREBBP,DUSP2,DUSP4,ELF1,ELK1,ETS1,FYN,ITGA1,ITGA4,ITGAE,ITGAL,KRAS,NRAS,PAK2,PIK3C2B,PIK3C3,PIK3R1,PIK3R4,PLA2G6,PPP1R10,PPP1R12A,PPP2CA,PPP2R1B,PPP2R2B,PPP2R5A,PPP2R5C,PPP2R5D,PPP2R5E,PRKACB,PRKAR2A,PRKCB,PXN,RAC2,RAP1B,RAP2A,RASD1,RPS6KA5,STAT3,TLN1,YWHAQ,YWHAZ
Androgen Signaling	1.47	-2.71	0.18	CALM1,CAMK4,CCNH,CREBBP,DNAJB1,EP300,GNB1,GTF2B,GTF2H3,HSP90AA1,KAT7,MNAT1,POLR2F,POLR2H,POLR2I,PRKACB,PRKAR2A,PRKCB,PRKCH,PRKCQ,PRKD3,TAF1,TAF13,TAF15,TAF9B,TBP
Endometrial Cancer Signaling	1.68	-2.71	0.22	AKT3,BAD,ELK1,GSK3B,KRAS,NRAS,PIK3C2B,PIK3C3,PIK3R1,PIK3R4,RAP1B,RAP2A,RASD1
Actin Nucleation by ARP-WASP Complex	1.87	-2.71	0.21	ARPC5L,FNBP1,ITGA1,ITGA4,ITGAE,ITGAL,KRAS,NCK1,NRAS,PPP1R12A,RAC2,RAP1B,RAP2A,RASD1,RHOH,RHOT1,RHOT2,WIPF1
Melanoma Signaling	2.39	-2.71	0.27	AKT3,BAD,BRAF,CDKN2A,KRAS,NRAS,PIK3C2B,PIK3C3,PIK3R1,PIK3R4,RAP1B,RAP2A,RASD1
Oncostatin M Signaling	2.96	-2.71	0.31	ELK1,EPAS1,JAK1,KRAS,MT2A,NRAS,PLAU,RAP1B,RAP2A,RASD1,STAT3,STAT5A,STAT5B
ERB2-ERBB3 Signaling	2.91	-2.67	0.28	BAD,CDKN1B,ELK1,GSK3B,KRAS,NRAS,PIK3C2B,PIK3C3,PIK3R1,PIK3R4,RAP1B,RAP2A,RASD1,STAT3,STAT5A,STAT5B
Production of Nitric Oxide and Reactive Oxygen Species in Macrophages	4.11	-2.67	0.23	AKT3,CREBBP,CYBA,FNBP1,IFNG,IKBKB,IRF1,JAK1,MAP2K7,MAP3K14,MAP3K2,MAP3K3,MAP3K7,MAPK13,MAPK14,NCF1,NCF2,PIK3C2B,PIK3C3,PIK3R1,PIK3R4,PPP1R10,PPP1R12A,PPP2CA,PPP2R1B,PPP2R2B,PPP2R5A,PPP2R5C,PPP2R5D,PPP2R5E,PRKCB,PRKCH,PRKCQ,PRKD3,RAC2,RAP1B,RHOH,RHOT1,RHOT2
IL-22 Signaling	1.67	-2.65	0.29	AKT3,JAK1,MAPK13,MAPK14,STAT3,STAT5A,STAT5B
Regulation of Cellular Mechanics by Calpain Protease	1.76	-2.65	0.21	ACTN4,CAPN2,CCND2,CCND3,CDKN1B,EZR,ITGA1,ITGA4,ITGAE,ITGAL,KRAS,NRAS,PXN,RAP1B,RAP2A,RASD1,TLN1
RAN Signaling	2.77	-2.65	0.44	KPNA1,KPNA4,KPNA5,KPNA6,RANBP2,RCC1,TNPO1
mTOR Signaling	2.88	-2.65	0.20	AKT3,DGKZ,EIF3A,EIF3B,EIF4A1,EIF4A2,EIF4G1,EIF4G2,EIF4G3,FNBP1,KRAS,NRAS,PIK3C2B,PIK3C3,PIK3R1,PIK3R4,PPP2CA,PPP2R1B,PPP2R2B,PPP2R5A,PPP2R5C,PPP2R5D,PPP2R5E,PRKAA1,PRKCB,PRKCH,PRKCQ,PRKD3,PRR5,RAC2,RAP1B,RAP2A,RASD1,RHOH,RHOT1,RHOT2,RICTOR,RPS6KA5
Insulin Receptor Signaling	1.59	-2.56	0.19	ACLY,AKT3,BAD,FYN,GSK3B,INPP5D,JAK1,KRAS,NCK1,NRAS,PDE3B,PIK3C2B,PIK3C3,PIK3R1,PIK3R4,PPP1R10,PPP1R12A,PRKACB,PRKAR2A,RAP1B,RAP2A,RASD1,STX4,VAMP2
Erythropoietin Signaling Pathway	1.47	-2.50	0.18	AKT3,BAD,BCL2L1,CCND2,CD40LG,ELK1,GSK3B,IFNG,IL2,KRAS,LTB,NRAS,PIK3C2B,PIK3C3,PIK3R1,PIK3R4,PRKCB,PRKCH,PRKCQ,PRKD3,RAC2,RAP1B,RAP2A,RASD1,STAT5A,STAT5B,TNFSF14
Neuregulin Signaling	2.11	-2.50	0.21	AKT3,BAD,CDKN1B,ELK1,HSP90AA1,ITGA1,ITGA4,ITGAE,ITGAL,KRAS,MATK,NRAS,PIK3R1,PRKCB,PRKCH,PRKCQ,PRKD3,RAP1B,RAP2A,RASD1,STAT5A,STAT5B
SPINK1 General Cancer Pathway	2.68	-2.50	0.28	AKT3,GZMA,JAK1,KRAS,MT2A,NRAS,PIK3C2B,PIK3C3,PIK3R1,PIK3R4,RAP1B,RAP2A,RASD1,STAT3
Role of PKR in Interferon Induction and Antiviral Response	1.61	-2.40	0.19	BAX,CASP3,CASP8,DNAJC3,EIF2AK2,FAS,HMGB1,HSP90AA1,HSPA1L,HSPA6,HSPA8,IFNG,IKBKB,IRF1,IRF9,JAK1,MAP2K7,MAP3K7,MAPK13,MAPK14,PYCARD,STAT3,TAB2
Type I Diabetes Mellitus Signaling	2.83	-2.40	0.22	BCL2,CASP3,CASP8,CD247,CD3D,CD3E,CD3G,FAS,FCER1G,HLA-A,HLA-DRB5,HLA-F,IFNG,IKBKB,IL2,IRF1,JAK1,MAP2K7,MAP3K14,MAP3K7,MAPK13,MAPK14,PIAS1,PRF1,SOCS1,SOCS2,TRGC2
NUR77 Signaling in T Lymphocytes	3.27	-2.40	0.24	BCL2,CABIN1,CALM1 (includes others),CAMK4,CASP3,CD247,CD3D,CD3E,CD3G,EP300,FCER1G,HLA-A,HLA-DRB5,HLA-F,MAP3K2,MAP3K3,PCNA,PPP3CA,PPP3CB,PRKCB,PRKCH,PRKCQ,PRKD3,SIN3A,TRGC2
CDC42 Signaling	1.84	-2.36	0.19	ARHGEF6,ARPC5L,CD247,CD3D,CD3E,CD3G,CFL2,EXOC1,EXOC6,FCER1G,FGD3,GSK3B,HLA-A,HLA-DRB5,HLA-F,IQGAP1,ITGA1,ITGA4,ITGAE,ITGAL,ITK,MAPK13,MAPK14,MPRIP,PAK2,PPP1R12A,TRGC2,WIPF1
ILK Signaling	1.74	-2.35	0.18	ACTN4,AKT3,ARHGEF6,CASP3,CFL2,CREB1,CREB3,CREBBP,FNBP1,GSK3B,MYH9,PIK3C2B,PIK3C3,PIK3R1,PIK3R4,PPP1R12A,PPP2CA,PPP2R1B,PPP2R2B,PPP2R5A,PPP2R5C,PPP2R5D,PPP2R5E,PXN,RAC2,RHOH,RHOT1,RHOT2,RICTOR,RPS6KA5
Role of CHK Proteins in Cell Cycle Checkpoint Control	1.32	-2.33	0.21	ATM,PCNA,PPP2CA,PPP2R1B,PPP2R2B,PPP2R5A,PPP2R5C,PPP2R5D,PPP2R5E,RAD9A,TLK1
Assembly of RNA Polymerase II Complex	1.88	-2.31	0.25	CCNH,GTF2B,GTF2H3,MNAT1,POLR2F,POLR2H,POLR2I,TAF1,TAF13,TAF15,TAF9B,TBP
Systemic Lupus Erythematosus In T Cell Signaling Pathway	7.00	-2.26	0.25	AKT3,CAMK4,CASP10,CASP3,CASP6,CASP8,CD247,CD3D,CD3E,CD3G,CD40LG,CD44,CREB1,CREB3,CREBBP,CREM,DNMT1,ELF1,EZR,FAS,FCER1G,FNBP1,HLA-A,HLA-DRB5,HLA-F,ICOS,IL2,ITGAL,KRAS,LAT,MAP2K7,MSN,NRAS,PIK3C2B,PIK3C3,PIK3R1,PIK3R4,PPP2CA,PPP2R1B,PPP2R2B,PPP2R5A,PPP2R5C,PPP2R5D,PPP2R5E,PPP3CA,PPP3CB,RAC2,RAP1B,R
nNOS Signaling in Neurons	1.50	-2.24	0.23	CALM1 (includes others),CAMK4,CAPN2,PPP3CA,PPP3CB,PRKCB,PRKCH,PRKCQ,PRKD3,RASD1
p70S6K Signaling	3.87	-2.20	0.25	AKT3,BAD,F2R,IL2RG,JAK1,KRAS,NRAS,PIK3C2B,PIK3C3,PIK3R1,PIK3R4,PLCB3,PPP2CA,PPP2R1B,PPP2R2B,PPP2R5A,PPP2R5C,PPP2R5D,PPP2R5E,PRKCB,PRKCH,PRKCQ,PRKD3,RAP1B,RAP2A,RASD1,SYK,YWHAQ,YWHAZ
Pyroptosis Signaling Pathway	1.61	-2.18	0.20	BAX,CASP3,CASP8,DHX9,GBP3,GBP4,GBP5,GZMA,HMGB1,IRF2,MAPK13,MAPK14,PRKACB,PRKAR2A,PTGER4,PYCARD,TXNIP
Induction of Apoptosis by HIV1	2.04	-2.14	0.23	BAX,BCL2,BCL2L1,CASP3,CASP8,CXCR4,DAXX,DFFA,FAS,HTRA2,IKBKB,MAP2K7,MAP3K14,SLC25A6,TRAF1
CSDE1 Signaling Pathway	1.49	-2.11	0.22	AKT3,CCND3,CSDE1,DCP1A,DCP2,DDX6,FUS,PABPC4,PAIP1,PTBP1,PUM1
Non-Small Cell Lung Cancer Signaling	1.58	-2.11	0.20	AKT3,BAD,CCND2,CCND3,CDKN2A,HDAC8,KRAS,NRAS,PIK3C2B,PIK3C3,PIK3R1,PIK3R4,RAP1B,RAP2A,RASD1,RBL2,SIN3A,STK4
CLEAR Signaling Pathway	1.59	-2.11	0.16	AKT3,ATP6AP1,ATP6V0D1,ATP6V0E2,CD63,CREB1,CREB3,CREBBP,CTNS,DEPDC5,EP300,GBA,GSK3B,IKBKB,KRAS,MAPK13,MAPK14,NAGPA,NRAS,PML,PPP2CA,PPP2R1B,PPP2R2B,PPP2R5A,PPP2R5C,PPP2R5D,PPP2R5E,PPP3CA,PPP3CB,PRKAA1,PRKCB,PRKCH,PRKCQ,PRKD3,RAP1B,RAP2A,RASD1,RRAGB,SEH1L,STK4,VPS11,VPS26A,YWHAQ,YWHAZ
IL-7 Signaling Pathway	4.01	-1.96	0.29	AKT3,BAD,BAX,BCL2,CCND3,CDKN1B,FYN,GSK3B,IFNG,IL2RG,IL7R,JAK1,MAPK13,MAPK14,PIK3C2B,PIK3C3,PIK3R1,PIK3R4,SOCS1,STAT5A,STAT5B
Telomerase Signaling	4.96	-1.96	0.29	AKT3,ELF1,ETS1,HDAC8,HSP90AA1,IL2,IL2RB,IL2RG,KRAS,NRAS,PIK3C2B,PIK3C3,PIK3R1,PIK3R4,POT1,PPP2CA,PPP2R1B,PPP2R2B,PPP2R5A,PPP2R5C,PPP2R5D,PPP2R5E,RAP1B,RAP2A,RASD1,TERF1,TERF2IP,TINF2
Tumoricidal Function of Hepatic Natural Killer Cells	2.22	-1.89	0.33	BAX,CASP3,CASP6,CASP8,DFFA,FAS,ITGAL,PRF1
Granzyme B Signaling	3.59	-1.89	0.50	CASP3,CASP8,DFFA,LMNB1,NUMA1,PARP1,PRF1,PRKDC
Unfolded protein response	3.20	-1.73	0.25	ATF6,BCL2,CD82,CEBPZ,DNAJA1,DNAJA3,DNAJA4,DNAJB1,DNAJB12,DNAJB6,DNAJC17,DNAJC21,DNAJC3,DNAJC7,ERN1,HSPA1L,HSPA6,HSPA8,HSPH1,MAP2K7,P4HB,UBXN4
Cytotoxic T Lymphocyte-mediated Apoptosis of Target Cells	3.85	-1.73	0.34	BCL2,CASP3,CASP6,CASP8,CD247,CD3D,CD3E,CD3G,DFFA,FAS,FCER1G,HLA-A,HLA-F,PRF1,TRGC2
Coronavirus Replication Pathway	1.44	-1.67	0.24	ARCN1,COPA,COPB1,COPG1,GSK3B,IFITM1,IFITM3,PI4KB,TUBA4A
Ceramide Signaling	3.43	-1.61	0.26	AKT3,BAD,BCL2,KRAS,NRAS,PIK3C2B,PIK3C3,PIK3R1,PIK3R4,PPP2CA,PPP2R1B,PPP2R2B,PPP2R5A,PPP2R5C,PPP2R5D,PPP2R5E,RAP1B,RAP2A,RASD1,S1PR1,S1PR4,SMPD1
Angiopoietin Signaling	3.34	-1.60	0.28	AKT3,ANGPT2,BAD,DOK2,IKBKB,KRAS,NCK1,NRAS,PAK2,PIK3C2B,PIK3C3,PIK3R1,PIK3R4,RAP1B,RAP2A,RASD1,STAT5A,STAT5B,TNIP1
MYC Mediated Apoptosis Signaling	1.49	-1.51	0.22	BAX,BCL2,BCL2L1,CASP3,CASP8,CDKN2A,CREBBP,FAS,IKBKB,PRKACB,PRKAR2A
T Cell Exhaustion Signaling Pathway	5.34	-1.13	0.25	AKT3,BATF,CD247,CD3D,CD3E,CD3G,FCER1G,FOXP1,HLA-A,HLA-DRB5,HLA-F,IFNG,IRF9,JAK1,KRAS,LAG3,MGAT5,NRAS,PDCD1,PIK3C2B,PIK3C3,PIK3R1,PIK3R4,PPP2CA,PPP2R1B,PPP2R2B,PPP2R5A,PPP2R5C,PPP2R5D,PPP2R5E,PRDM1,PRKCQ,RAP1B,RAP2A,RASD1,SMAD2,STAT3,STAT4,TBX21,TCF7,TRGC2,ZAP70
Huntington's Disease Signaling	3.13	-1.04	0.19	AKT3,BAX,BCL2L1,CAPN2,CASP10,CASP3,CASP6,CASP8,CLTC,CREB1,CREB3,CREBBP,DNAJB1,EP300,GNB1,GOSR2,HDAC8,HIP1,HSPA1L,HSPA6,HSPA8,MAP2K7,NCOR1,NCOR2,PIK3C2B,PIK3C3,PIK3R1,PIK3R4,PLCB3,POLR2F,POLR2H,POLR2I,PRKCB,PRKCH,PRKCQ,PRKD3,PSMC6,PSMD1,PSMD10,PSMD2,PSMD9,PSME4,PSMF1,SIN3A,STX16,TAF9B,TBP,UBC,VAMP2,VTI1B
Regulation of eIF4 and p70S6K Signaling	2.21	-1.00	0.19	AKT3,EIF3A,EIF3B,EIF4A1,EIF4A2,EIF4G1,EIF4G2,EIF4G3,ITGA1,ITGA4,ITGAE,ITGAL,KRAS,MAPK13,MAPK14,NRAS,PAIP1,PIK3C2B,PIK3C3,PIK3R1,PIK3R4,PPP2CA,PPP2R1B,PPP2R2B,PPP2R5A,PPP2R5C,PPP2R5D,PPP2R5E,RAP1B,RAP2A,RASD1
Interferon Signaling	3.22	-0.90	0.34	BAX,BCL2,IFITM1,IFITM3,IFNG,IRF1,IRF9,ISG15,JAK1,PIAS1,SOCS1,TAP1
PI3K/AKT Signaling	2.01	-0.82	0.18	AKT3,BAD,BCL2,BCL2L1,CDKN1B,GSK3B,HSP90AA1,IKBKB,IL13RA1,IL18R1,IL2RB,IL2RG,IL7R,INPP5D,ITGA1,ITGA4,ITGAE,ITGAL,JAK1,KRAS,NRAS,PIK3R1,PPP2CA,PPP2R1B,PPP2R2B,PPP2R5A,PPP2R5C,PPP2R5D,PPP2R5E,RAP1B,RAP2A,RASD1,YWHAQ,YWHAZ
p53 Signaling	3.39	-0.47	0.26	AKT3,ATM,BAX,BCL2,BCL2L1,CASP6,CCND2,CDKN2A,EP300,FAS,GADD45G,GSK3B,HIPK2,MAPK14,MDM4,PCNA,PIAS1,PIK3C2B,PIK3C3,PIK3R1,PIK3R4,PML,PRKDC
Apoptosis Signaling	3.15	0.00	0.24	BAD,BAX,BCL2,BCL2L1,BIRC6,CAPN2,CASP10,CASP3,CASP6,CASP8,DFFA,FAS,HTRA2,IKBKB,KRAS,MAP2K7,MAP3K14,NRAS,PARP1,PRKCQ,RAP1B,RAP2A,RASD1,SPTAN1
Cell Cycle: G2/M DNA Damage Checkpoint Regulation	1.56	0.33	0.23	ATM,CDKN2A,CUL1,EP300,HIPK2,MDM4,PRKDC,TOP2B,TRIP12,YWHAQ,YWHAZ
ATM Signaling	4.18	1.15	0.28	ATF1,ATM,BRAT1,CBX5,CREB1,CREB3,CREBBP,GADD45G,HERC2,HP1BP3,MAPK13,MAPK14,MDM4,PPP2CA,PPP2R1B,PPP2R2B,PPP2R5A,PPP2R5C,PPP2R5D,PPP2R5E,RAD9A,RNF168,TLK1,TRRAP,ZEB1
Inhibition of ARE-Mediated mRNA Degradation Pathway	6.02	1.18	0.27	AKT3,CD40LG,CNOT11,CNOT2,CNOT7,CNOT8,CNOT9,DCP1A,DCP2,DDX6,EXOSC1,EXOSC10,EXOSC6,EXOSC7,LTB,MAP3K7,MAPK13,MAPK14,PAPOLA,PPP2CA,PPP2R1B,PPP2R2B,PPP2R5A,PPP2R5C,PPP2R5D,PPP2R5E,PRKACB,PRKAR2A,PSMC6,PSMD1,PSMD10,PSMD2,PSMD9,PSME4,PSMF1,TNFSF14,YWHAQ,YWHAZ,ZFP36L2,ZNF331
Sumoylation Pathway	2.97	1.21	0.24	CREBBP,DAXX,EP300,ETS1,FAS,FNBP1,ISG20,PCNA,PIAS1,PML,RAC2,RANBP2,RCC1,RHOH,RHOT1,RHOT2,SENP1,SENP6,SENP7,SP100,SP3,UBA2,ZEB1
Antiproliferative Role of TOB in T Cell Signaling	2.02	1.26	0.26	CD247,CD3D,CD3E,CD3G,CDKN1B,CUL1,FCER1G,IL2,PABPC4,SMAD2,TOB1,TRGC2
Epithelial Adherens Junction Signaling	1.35	1.40	0.17	AKT3,ARPC5L,CFL2,FYN,IQGAP1,KRAS,NF2,NOTCH1,NRAS,PAK2,PPP2CA,PPP2R1B,PPP2R2B,PPP2R5A,PPP2R5C,PPP2R5D,PPP2R5E,PRKAA1,RAP1B,RAP2A,RASD1,STK4,TCF7,YWHAQ,YWHAZ
Semaphorin Neuronal Repulsive Signaling Pathway	1.33	1.53	0.18	AKT3,CD44,CFL2,FYN,GSK3B,ITGA1,ITGA4,ITGAE,ITGAL,MAP2K7,MPRIP,PAK2,PDE4B,PDE4D,PIK3C2B,PIK3C3,PIK3R1,PIK3R4,PPP1R12A,PRKACB,PRKAR2A,SEMA4D
VDR/RXR Activation	1.62	1.73	0.22	CCL5,CDKN1B,EP300,GTF2B,IFNG,IL2,NCOA3,NCOR1,NCOR2,PPARD,PRKCB,PRKCH,PRKCQ,PRKD3
HIPPO signaling	2.33	1.73	0.23	CD44,CUL1,DLG1,NF2,PATJ,PPP1R10,PPP1R12A,PPP2CA,PPP2R1B,PPP2R2B,PPP2R5A,PPP2R5C,PPP2R5D,PPP2R5E,SMAD2,STK4,YWHAQ,YWHAZ
Cyclins and Cell Cycle Regulation	2.57	2.14	0.24	ATM,CCND2,CCND3,CCNH,CDKN1B,CDKN2A,CDKN2B,CUL1,GSK3B,HDAC8,PPP2CA,PPP2R1B,PPP2R2B,PPP2R5A,PPP2R5C,PPP2R5D,PPP2R5E,RBL2,SIN3A
PD-1, PD-L1 cancer immunotherapy pathway	4.87	3.40	0.28	AKT3,BCL2L1,CBLB,CD247,CDKN1B,ELK1,GSK3B,HLA-A,HLA-DRB5,HLA-F,IFNG,IL2,IL2RB,IL2RG,JAK1,LAT,LCK,PDCD1,PDCD4,PIK3C2B,PIK3C3,PIK3R1,PIK3R4,PRKCQ,RASGRP1,STAT5A,STAT5B,ZAP70
Cleavage and Polyadenylation of Pre-mRNA	1.31		0.33	CPSF1,CSTF1,PAPOLA,WDR33
Tight Junction Signaling	1.35		0.17	AKT3,CPSF1,CSTF1,EPB41,F11R,GOSR2,MARK2,MYH9,PATJ,PPP2CA,PPP2R1B,PPP2R2B,PPP2R5A,PPP2R5C,PPP2R5D,PPP2R5E,PRKACB,PRKAR2A,SAFB,SPTAN1,STX16,STX4,VAMP2,VTI1B,YBX3
Allograft Rejection Signaling	1.37		0.20	CD247,CD3D,CD3E,CD3G,CD40LG,FAS,FCER1G,HLA-A,HLA-DRB5,HLA-F,IFNG,IL2,PRF1,TRGC2
Autoimmune Thyroid Disease Signaling	1.45		0.21	CD247,CD3D,CD3E,CD3G,CD40LG,FAS,FCER1G,HLA-A,HLA-DRB5,HLA-F,IL2,PRF1,TRGC2
RAR Activation	1.46		0.17	AKT3,ARID1A,CCNH,CREBBP,EP300,GTF2H3,HLTF,MAPK13,MAPK14,MNAT1,NCOR1,NCOR2,NSD1,PARP1,PIK3R1,PML,PRKACB,PRKAR2A,PRKCB,PRKCH,PRKCQ,PRKD3,SMAD2,SMARCA2,SMARCC2,SNW1,STAT5A,STAT5B,TNIP1,ZBTB16
Graft-versus-Host Disease Signaling	1.51		0.21	CD247,CD3D,CD3E,CD3G,FAS,FCER1G,HLA-A,HLA-DRB5,HLA-F,IFNG,IL2,PRF1,TRGC2
IL-4 Signaling	1.52		0.20	AKT3,HLA-A,HLA-DRB5,IL13RA1,IL2RG,INPP5D,JAK1,KRAS,NRAS,PIK3C2B,PIK3C3,PIK3R1,PIK3R4,RAP1B,RAP2A,RASD1,SOCS1
Telomere Extension by Telomerase	1.54		0.33	POT1,TERF1,TERF2IP,TINF2,TNKS2
Phagosome Maturation	1.55		0.18	ATP6AP1,ATP6V0D1,ATP6V0E2,CTSW,DYNC1H1,DYNC1LI2,GOSR2,HLA-A,HLA-DRB5,HLA-F,NCF1,NCF2,PIK3C3,PIKFYVE,PRDX2,RAB5B,RAC2,STX16,TAP1,TUBA4A,VAMP2,VPS11,VPS37A,VPS37B,VTI1B
Germ Cell-Sertoli Cell Junction Signaling	1.56		0.18	ACTN4,CFL2,FNBP1,IQGAP1,KRAS,MAP2K7,MAP3K14,MAP3K2,MAP3K3,MAP3K7,MAPK14,NRAS,PAK2,PIK3C2B,PIK3C3,PIK3R1,PIK3R4,PXN,RAB8B,RAC2,RAP1B,RAP2A,RASD1,RHOH,RHOT1,RHOT2,TUBA4A
Role of JAK family kinases in IL-6-type Cytokine Signaling	1.57		0.28	JAK1,MAPK13,MAPK14,SOCS1,STAT3,STAT5A,STAT5B
DNA Methylation and Transcriptional Repression Signaling	1.61		0.27	ARID4B,CHD3,DNMT1,H4C3,MECP2,RBBP4,SAP30,SIN3A
Protein Ubiquitination Pathway	1.61		0.17	ANAPC1,BIRC6,CUL1,CUL2,DNAJA1,DNAJB1,DNAJB12,DNAJB6,DNAJC17,DNAJC21,DNAJC3,DNAJC7,HLAA,HLAF,HSP90AA1,HSPA1L,HSPA6,HSPA8,HSPH1,IFNG,PSMC6,PSMD1,PSMD10,PSMD2,PSMD9,RBX1,TAP1,UBC,UBE2D4,UBE2E3,UBE2G1,UBE2V1,UBE3A,USP10,USP15,USP3,USP34,USP36,USP39,USP48,USP53,USP9Y
T Helper Cell Differentiation	1.72		0.21	CD247,CD3D,CD3E,CD3G,CD40LG,FCER1G,GATA3,HLA-A,HLA-DRB5,ICOS,IFNG,IL18R1,IL2,IL2RG,STAT3,STAT4,TBX21,TRGC2
Role of Tissue Factor in Cancer	1.73		0.20	AKT3,BCL2L1,CASP3,CFL2,FYN,KRAS,LCK,MAPK13,MAPK14,NRAS,P4HB,PIK3C2B,PIK3C3,PIK3R1,PIK3R4,RAP1B,RAP2A,RASD1,RPS6KA5,STAT5A,STAT5B
Nucleotide Excision Repair Pathway	1.74		0.27	CCNH,ERCC5,GTF2H3,MNAT1,POLR2F,POLR2H,POLR2I,RPA2,XPC
Prostate Cancer Signaling	1.92		0.20	AKT3,BAD,BCL2,CDKN1B,CREB1,CREB3,CREBBP,GSK3B,GSTP1,HDAC8,HSP90AA1,KRAS,NRAS,PIK3C2B,PIK3C3,PIK3R1,PIK3R4,RAP1B,RAP2A,RASD1,RBL2,SIN3A
Primary Immunodeficiency Signaling	2.02		0.26	CD3D,CD3E,CD40LG,CD8A,ICOS,IL2RG,IL7R,LCK,PTPRC,RFX5,TAP1,ZAP70
Role of MAPK Signaling in the Pathogenesis of Influenza	2.09		0.23	AKT3,BAX,BCL2,CASP3,CCL5,IFNG,KRAS,MAP2K7,MAPK13,MAPK14,NRAS,PLA2G6,PLAAT4,PNPLA8,RAP1B,RAP2A,RASD1
Chronic Myeloid Leukemia Signaling	2.21		0.21	AKT3,BAD,BCL2L1,CCND2,CCND3,CDKN1B,CDKN2A,HDAC8,IKBKB,KRAS,NRAS,PIK3C2B,PIK3C3,PIK3R1,PIK3R4,RAP1B,RAP2A,RASD1,RBL2,SIN3A,STAT5A,STAT5B
Cell Cycle Regulation by BTG Family Proteins	2.22		0.29	BTG1,BTG2,CNOT7,PPP2CA,PPP2R1B,PPP2R2B,PPP2R5A,PPP2R5C,PPP2R5D,PPP2R5E
Glucocorticoid Receptor Signaling	2.26		0.16	AKT3,ANXA1,ARID1A,BCL2,BCL2L1,CCL5,CCNH,CD247,CD3D,CD3E,CD3G,CREB1,CREBBP,ELK1,EP300,FKBP5,GTF2B,GTF2H3,HLAA,HLADRB5,HLAF,HLTF,HMGB1,HSP90AA1,HSPA1L,HSPA6,HSPA8,IFNG,IKBKB,IL13RA1,IL18R1,IL2,IL2RB,IL2RG,IL7R,JAK1,KRAS,MAP2K7,MAP3K14,MAP3K7,MAPK13,MAPK14,MNAT1,MTND5,NCOA3,NCOR1,NCOR2,NDUFA10,NDUFS8,NRAS,PIK3C2B,PIK3C3,PIK3R1,PIK3R4,PLA2G6,PLAU,POLR2F,POLR2H,POLR2I,PPP3CA,PPP3CB,PRKAA1,PRKACB,RAP1B,RAP2A,RASD1,RPS6KA5,SMAD2,SMARCA2,SMARCC2,STAT3,STAT5A,STAT5B,TAF1,TAF13,TAF15,TAF9B,TBP,TSC22D3
Systemic Lupus Erythematosus Signaling	2.39		0.19	AKT3,CAMK4,CD247,CD3D,CD3E,CD3G,CD40LG,CREM,FCER1G,HLAA,HLAF,IL2,INPP5D,KRAS,LAT,LCK,LSM8,NRAS,PIK3C2B,PIK3C3,PIK3R1,PIK3R4,PRPF18,PRPF38B,PRPF39,PRPF40A,PTPRC,RAP1B,RAP2A,RASD1,SNRNP200,SNRNP27,SNRNP70,SNRPB2,SNRPN,TRGC2
Virus Entry via Endocytic Pathways	2.43		0.23	AP1G2,AP1M1,AP3B1,CLTC,FYN,HLA-A,HLA-F,KRAS,NRAS,PIK3C2B,PIK3C3,PIK3R1,PIK3R4,PRKCB,PRKCH,PRKCQ,PRKD3,RAC2,RAP1B,RAP2A,RASD1
Molecular Mechanisms of Cancer	2.63		0.17	AKT3,ARHGEF1,ARHGEF3,ARHGEF6,ATM,BAD,BAX,BCL2,BCL2L1,BRAF,CAMK2G,CASP10,CASP3,CASP6,CASP8,CCND2,CCND3,CDK3,CDKN1B,CDKN2A,CDKN2B,CREBBP,DAXX,ELK1,EP300,FAS,FNBP1,FYN,GNB1,GSK3B,HDAC8,HIPK2,ITGA1,ITGA4,ITGAE,ITGAL,JAK1,KRAS,MAP3K7,MAPK13,MAPK14,NCSTN,NOTCH1,NRAS,PAK2,PIK3C2B,PIK3C3,PIK3R1,PIK3R4,PLCB3,PRKACB,PRKAR2A,PRKCB,PRKCH,PRKCQ,PRKD3,PRKDC,RAC2,RAP1B,RAP2A,RASD1,RASGRP1,RBL2,RHOH,RHOT1,RHOT2,SIN3A,SMAD2,TAB2
Hereditary Breast Cancer Signaling	2.67		0.21	AKT3,ARID1A,ATM,BARD1,CCND2,CCND3,CREBBP,EP300,FANCG,GADD45G,HDAC8,HLTF,KRAS,NRAS,PIK3C2B,PIK3C3,PIK3R1,PIK3R4,POLR2F,POLR2H,POLR2I,RAP1B,RAP2A,RASD1,SMARCA2,SMARCC2,UBC,XPC
Xenobiotic Metabolism Signaling	2.74		0.19	ANKRA2,CAMK2G,CAMK4,CHST11,CHST12,CREBBP,DNAJC7,EP300,FTL,GSTP1,HSP90AA1,KRAS,MAF,MAP2K7,MAP3K14,MAP3K2,MAP3K3,MAP3K7,MAPK13,MAPK14,MGST1,MGST2,NCOR2,NRAS,PIK3C2B,PIK3C3,PIK3R1,PIK3R4,PPP2CA,PPP2R1B,PPP2R2B,PPP2R5A,PPP2R5C,PPP2R5D,PPP2R5E,PRKCB,PRKCH,PRKCQ,PRKD3,RAP1B,RAP2A,RASD1,RBX1,SNW1
Docosahexaenoic Acid (DHA) Signaling	3.09		0.33	AKT3,BAD,BAX,BCL2,BCL2L1,CASP3,GSK3B,PIK3C2B,PIK3C3,PIK3R1,PIK3R4,PNPLA2
Role of JAK1 and JAK3 in γc Cytokine Signaling	3.61		0.29	IL2,IL2RB,IL2RG,IL7R,JAK1,KRAS,NRAS,PIK3C2B,PIK3C3,PIK3R1,PIK3R4,RAP1B,RAP2A,RASD1,SOCS1,STAT3,STAT5A,STAT5B,SYK
Th1 and Th2 Activation Pathway	4.23		0.23	CD247,CD3D,CD3E,CD3G,CD40LG,CD8A,CXCR4,CXCR6,GATA3,HLAA,HLADRB5,ICOS,IFNG,IKZF1,IL18R1,IL2,IL2RB,IL2RG,IRF1,JAK1,KLRC1,KLRD1,MAF,NCSTN,NOTCH1,PIK3C2B,PIK3C3,PIK3R1,PIK3R4,PRKCQ,RUNX3,S1PR1,SOCS1,STAT3,STAT4,STAT5A,STAT5B,TBX21
Granzyme A Signaling	4.82		0.60	CREBBP,EP300,GZMA,H1-10,H1-2,H1-3,H1-4,HMGB2,PRF1
CTLA4 Signaling in Cytotoxic T Lymphocytes	8.51		0.36	AKT3,AP1G2,AP1M1,CD247,CD3D,CD3E,CD3G,CD8A,CD8B,CLTC,FCER1G,FYN,GRAP2,HLAA,HLAF,LAT,LCK,PIK3C2B,PIK3C3,PIK3R1,PIK3R4,PPP2CA,PPP2R1B,PPP2R2B,PPP2R5A,PPP2R5C,PPP2R5D,PPP2R5E,PTPN22,SYK,TRAT1,TRGC2,ZAP70

Supplementary Table 6. List of DEGs in cluster 0 and cluster 1, related to *Cytoskeletal rearrangement, cell motility and cell-cell interactions*.

Geni	log2FC cluster 0 vs cluster 1	adj p value
CCL5	-3,32	2,78E-32
IFNG	-2,78	2,56E-26
GNLY	-2,62	4,81E-28
CD3D	-2,54	1,47E-35
CD69	-2,51	2,09E-27
CD3E	-2,37	4,89E-37
IL7R	-2,34	8,55E-36
XCL1	-2,28	1,14E-36
CD3G	-2,16	1,63E-38
CD2	-2,16	4,70E-31
IL2	-2,07	1,69E-27
LCK	-2,05	1,60E-35
CD8A	-2,01	1,76E-34
ETS1	-1,97	4,04E-35
PIK3R1	-1,94	8,34E-20
HLA-DRB5	-1,87	8,55E-06
BCL11B	-1,78	3,27E-36
LAT	-1,77	5,24E-35
PTGDR	-1,71	1,47E-34
CD40LG	-1,69	1,19E-32
GATA3	-1,68	8,34E-30
ITK	-1,63	5,79E-35
KLRG1	-1,61	8,55E-20
PDCD1	-1,60	6,01E-31
BATF	-1,59	2,73E-35
STAT4	-1,57	1,45E-27
FYN	-1,57	1,16E-31
SIRPG	-1,56	5,36E-33
ITGA1	-1,55	4,45E-27
PRDM1	-1,53	2,43E-22
F2R	-1,52	9,14E-21
SH2D1A	-1,52	5,32E-28
ADGRE5	-1,52	4,88E-20
STK4	-1,50	1,55E-23
ZAP70	-1,49	3,16E-34
C1GALT1	-1,48	1,13E-24
SLFN12L	-1,46	1,14E-30
ITGA4	-1,46	3,21E-22
PIKFYVE	-1,44	6,66E-18
RICTOR	-1,44	1,74E-10
CXCR4	-1,43	1,32E-15
SMPD1	-1,42	9,71E-25
PTPRC	-1,42	5,40E-21
MIA3	-1,40	2,89E-14
LTB	-1,39	2,79E-20
CD247	-1,39	6,29E-29
ORMDL3	-1,37	5,19E-13
P2RY8	-1,36	1,84E-25
RUNX3	-1,36	1,27E-18
MPRIP	-1,36	1,65E-04
PARP1	-1,35	7,22E-20
CBFB	-1,33	2,25E-16
CYFIP2	-1,33	2,65E-29
CASP3	-1,32	6,63E-13
PIK3R4	-1,32	8,10E-27
PPP2R5C	-1,32	2,71E-27
ICOS	-1,31	7,35E-20
PXN	-1,31	1,49E-21
SKAP1	-1,31	5,92E-28

PPP2R5E	-1,30	1,37E-13
RHOT1	-1,30	3,80E-15
BCL2	-1,29	2,09E-13
DAXX	-1,29	8,64E-15
PTPN22	-1,26	1,51E-14
ARHGEF6	-1,25	2,35E-20
CD47	-1,24	1,54E-18
PIP4K2A	-1,23	1,13E-09
RASGRP1	-1,21	3,93E-17
MATK	-1,21	1,09E-17
CBLB	-1,20	1,38E-15
CCND2	-1,19	1,37E-14
S1PR1	-1,19	2,19E-18
FLT3LG	-1,18	3,37E-18
CD58	-1,17	1,13E-09
HLA-F	-1,17	1,86E-19
BRAF	-1,17	4,07E-14
MMP7	-1,15	1,96E-03
BAD	-1,14	8,78E-19
TNFSF14	-1,14	1,82E-09
MAP2K7	-1,13	2,86E-14
F11R	-1,13	6,42E-09
PLA2G6	-1,12	2,15E-16
GYPC	-1,12	9,89E-10
CREB3	-1,12	2,26E-15
IL18R1	-1,12	2,63E-09
SLAMF1	-1,11	3,41E-15
CD5	-1,11	3,35E-19
PTGER4	-1,10	3,72E-14
ITGAL	-1,09	7,64E-08
CYTIP	-1,08	4,76E-21
STAT5B	-1,07	3,40E-14
PRKCQ	-1,06	5,91E-19
KRAS	-1,05	4,99E-05
FNBP1	-1,05	1,22E-10
IKBKB	-1,04	2,05E-18
FAS	-1,04	6,74E-11
PPP2R5D	-1,03	2,53E-11
CREBBP	-1,02	5,93E-08
FUT8	-1,02	2,15E-09
MGAT5	-1,01	1,44E-13
SOCS1	-1,00	1,78E-15
PIK3C2B	-1,00	1,53E-11
RASD1	-0,99	1,78E-11
OGT	-0,99	2,32E-11
JAK1	-0,99	3,30E-09
CAPN2	-0,99	3,67E-08
NRAS	-0,99	4,73E-12
NCK1	-0,99	9,54E-10
S1PR4	-0,99	4,52E-15
PLCB3	-0,98	4,63E-10
CD44	-0,98	5,67E-12
HMGB1	-0,97	2,10E-16
CCND3	-0,96	6,26E-18
PRKCH	-0,96	4,18E-07
DIAPH2	-0,96	1,96E-09
CREB1	-0,96	1,04E-13
LAG3	-0,95	3,40E-15
MYH9	-0,94	6,84E-16
EVL	-0,94	7,43E-22
TSPAN14	-0,94	3,75E-16

PRKAR2A	-0,94	1,46E-07
PDE4B	-0,94	3,93E-06
HEBP1	-0,93	4,16E-06
DOCK11	-0,93	3,80E-05
YWHAQ	-0,93	4,53E-21
CASP8	-0,93	2,67E-07
AKIRIN1	-0,92	1,77E-10
PRKACB	-0,92	4,18E-09
EZR	-0,91	7,27E-17
PREX1	-0,91	1,22E-19
WIPF1	-0,91	3,41E-18
TNFRSF18	-0,90	3,66E-09
SEMA4D	-0,90	1,99E-12
PLEKHA2	-0,90	5,95E-09
PPP2R2B	-0,90	6,01E-11
FGD3	-0,89	2,73E-04
MAPK13	-0,88	5,00E-10
ACTN4	-0,88	2,46E-15
ANGPT2	-0,88	1,67E-06
AQP3	-0,88	5,92E-13
CD6	-0,87	7,31E-09
GPR132	-0,87	8,86E-04
CLTC	-0,87	6,44E-04
ANO6	-0,87	3,90E-05
RAC2	-0,86	2,16E-17
GSK3B	-0,85	7,23E-04
MAP3K14	-0,85	1,19E-06
ARPC5L	-0,84	2,64E-10
RPS6KA5	-0,84	5,28E-12
STIM2	-0,84	2,13E-07
IQGAP1	-0,84	2,80E-10
LY96	0,85	3,11E-12
TNFAIP8L2	0,87	1,12E-05
RASGRP4	0,92	4,90E-03
CARD9	0,97	5,19E-04
AIF1	1,00	3,18E-11
SIGLEC7	1,00	1,52E-03
MS4A4A	1,07	1,08E-03
PPIF	1,09	5,38E-03
NCF2	1,19	4,28E-05
RAB34	1,28	3,32E-08
FCER1G	1,46	1,29E-15
PLAU	1,71	3,58E-03

Supplementary Table 7. List of DEGs in cluster 0 and cluster 1, related to *Activation state* .

Geni	lnFC cluster 0 vs 1	adj p value
KLRB1	-2,31	1,36E-33
CCL5	-2,31	2,78E-32
IL32	-2,12	1,52E-35
IFNG	-1,93	2,56E-26
GZMA	-1,91	9,39E-37
GNLY	-1,82	4,81E-28
KLRC1	-1,78	1,66E-30
CD3D	-1,76	1,47E-35
CD69	-1,74	2,09E-27
CD3E	-1,64	4,89E-37
IL7R	-1,63	8,55E-36
XCL1	-1,58	1,14E-36
CD52	-1,52	6,31E-28
TIGIT	-1,51	6,15E-35
CD3G	-1,50	1,63E-38
CD2	-1,50	4,70E-31
IL2	-1,44	1,69E-27
CD27	-1,43	2,23E-36
RORA	-1,42	6,85E-37
LCK	-1,42	1,60E-35
CD8A	-1,40	1,76E-34
KLRD1	-1,37	3,21E-28
ETS1	-1,37	4,04E-35
TOB1	-1,36	4,29E-29
DUSP4	-1,36	7,21E-18
PIK3R1	-1,35	8,34E-20
CLEC2D	-1,33	1,23E-32
CD7	-1,32	2,83E-26
HLA-DRB5	-1,30	8,55E-06
IKZF3	-1,24	8,21E-28
BCL11B	-1,23	3,27E-36
GPR174	-1,23	1,01E-30
LAT	-1,23	5,24E-35
EML4	-1,19	3,28E-27
PTGDR	-1,19	1,47E-34
PDE4D	-1,18	2,36E-34
CD40LG	-1,17	1,19E-32
CAMK4	-1,16	1,41E-37
GATA3	-1,16	8,34E-30
PARP8	-1,16	4,20E-26
PIK3IP1	-1,15	5,17E-21
PRF1	-1,15	5,70E-36
RBL2	-1,14	9,00E-30

SIT1	-1,14	3,86E-34
ITK	-1,13	5,79E-35
PDCD1	-1,11	6,01E-31
BATF	-1,11	2,73E-35
STAT4	-1,09	1,45E-27
GLMN	-1,09	6,22E-24
THEMIS	-1,09	6,78E-35
FYN	-1,09	1,16E-31
PTPN4	-1,09	3,66E-30
SH2D2A	-1,08	2,19E-35
SIRPG	-1,08	5,36E-33
ITGA1	-1,08	4,45E-27
PDE7A	-1,07	8,73E-28
PTPN7	-1,07	4,36E-23
PRDM1	-1,06	2,43E-22
TBP	-1,06	1,89E-26
IP6K1	-1,06	1,94E-26
F2R	-1,06	9,14E-21
SH2D1A	-1,06	5,32E-28
CTCF	-1,05	2,94E-14
STK4	-1,04	1,55E-23
BCLAF1	-1,04	7,24E-19
PPP1R10	-1,04	4,06E-14
ZAP70	-1,04	3,16E-34
RAB27A	-1,01	2,64E-25
ITGA4	-1,01	3,21E-22
PDCD4	-1,01	7,50E-22
TBC1D10C	-1,01	7,11E-27
STYX	-1,01	8,77E-16
MYBL1	-1,00	8,10E-14
PIKFYVE	-1,00	6,66E-18
RICTOR	-1,00	1,74E-10
SLAMF6	-0,99	1,38E-20
CXCR4	-0,99	1,32E-15
CDKN2A	-0,99	2,28E-27
SMPD1	-0,99	9,71E-25
PTPRC	-0,98	5,40E-21
TMC6	-0,98	1,86E-22
ERN1	-0,97	1,46E-16
AKAP11	-0,97	4,23E-20
CABIN1	-0,97	9,25E-21
MAF	-0,97	4,30E-17
SLA2	-0,97	1,61E-26
LTB	-0,96	2,79E-20
CD247	-0,96	6,29E-29

BTG1	-0,96	3,35E-21
SNTB1	-0,96	5,23E-32
KAT7	-0,96	6,85E-15
TSC22D3	-0,95	1,41E-13
RUNX3	-0,94	1,27E-18
MPRIP	-0,94	1,65E-04
TAB2	-0,94	3,70E-16
NMNAT1	-0,94	9,41E-27
PARP1	-0,94	7,22E-20
MS4A1	-0,93	8,97E-18
RCAN3	-0,93	1,44E-16
CBFB	-0,92	2,25E-16
CASP3	-0,92	6,63E-13
PIK3R4	-0,92	8,10E-27
PPP2R5C	-0,91	2,71E-27
EIF4G1	-0,91	5,26E-11
PXN	-0,91	1,49E-21
TRAF1	-0,91	3,73E-13
SKAP1	-0,91	5,92E-28
ISG20	-0,91	3,80E-15
PPP2R5E	-0,90	1,37E-13
RHOT1	-0,90	3,80E-15
BCL2	-0,90	2,09E-13
PNPLA2	-0,90	3,65E-10
DAXX	-0,89	8,64E-15
SIRT3	-0,89	1,09E-21
SMAD2	-0,88	8,14E-17
BACE1	-0,88	3,27E-29
LY9	-0,88	2,08E-20
PTPN22	-0,88	1,51E-14
IL2RB	-0,87	1,78E-32
HECTD1	-0,87	2,26E-18
ARHGEF6	-0,87	2,35E-20
ITPKB	-0,86	5,56E-19
LAX1	-0,86	3,29E-18
KPNA1	-0,86	2,56E-12
CD47	-0,86	1,54E-18
TXNIP	-0,86	4,28E-10
SLC36A4	-0,85	5,63E-10
PIP4K2A	-0,85	1,13E-09
SACM1L	-0,84	4,61E-18
RASGRP1	-0,84	3,93E-17
PRUNE1	-0,84	2,74E-13
MARK2	-0,84	2,44E-16
CBLB	-0,84	1,38E-15

PIAS1	-0,83	5,70E-16
KPNA4	-0,83	1,96E-11
CCND2	-0,83	1,37E-14
S1PR1	-0,82	2,19E-18
CAMK2G	-0,82	5,18E-13
DNAJC17	-0,82	2,21E-11
FLT3LG	-0,82	3,37E-18
ATM	-0,82	3,35E-15
ZEB1	-0,82	3,79E-11
CD58	-0,82	1,13E-09
NMT2	-0,82	3,64E-12
HLA-F	-0,81	1,86E-19
BRAF	-0,81	4,07E-14
POLR2I	-0,81	8,58E-20
PIM1	-0,81	9,37E-10
RAB5B	-0,81	1,03E-13
PYGL	-0,81	3,65E-12
VAMP4	-0,80	2,63E-08
CD82	-0,80	1,62E-09
HSPH1	-0,80	2,85E-04
POLM	-0,80	3,07E-17
MMP7	-0,80	1,96E-03
TCF7	-0,80	3,18E-09
RANBP2	-0,79	2,78E-09
ATG5	-0,79	2,12E-12
MT2A	-0,79	1,34E-08
BAD	-0,79	8,78E-19
TNFSF14	-0,79	1,82E-09
NCOA3	-0,79	1,17E-12
MAP2K7	-0,79	2,86E-14
DNAJA3	-0,79	7,81E-17
WNK1	-0,78	9,00E-19
DNAJC3	-0,78	8,98E-14
PLA2G6	-0,78	2,15E-16
CREB3	-0,78	2,26E-15
IL18R1	-0,78	2,63E-09
NCOR2	-0,77	1,04E-11
SLAMF1	-0,77	3,41E-15
CD5	-0,77	3,35E-19
IL2RG	-0,77	3,84E-11
PTGER4	-0,76	3,72E-14
ADAT2	-0,76	2,39E-10
GADD45G	-0,76	7,20E-12
GBA	-0,76	2,93E-12
ITGAL	-0,76	7,64E-08

AKAP13	-0,76	2,10E-15
CYTIP	-0,75	4,76E-21
DNAJB12	-0,75	3,53E-15
STAT5B	-0,75	3,40E-14
PML	-0,74	7,99E-11
FOXP1	-0,74	1,13E-21
PRKCQ	-0,74	5,91E-19
CYLD	-0,74	2,05E-20
PTGER2	-0,73	7,52E-08
ELF1	-0,73	6,23E-11
KRAS	-0,73	4,99E-05
PPP2R3C	-0,73	1,63E-11
FNBP1	-0,73	1,22E-10
CREM	-0,73	1,99E-05
IKBKB	-0,73	2,05E-18
RPA2	-0,72	1,61E-09
JUNB	-0,72	2,81E-09
FAS	-0,72	6,74E-11
PPP2R5D	-0,72	2,53E-11
AKAP9	-0,72	1,19E-16
CREBBP	-0,71	5,93E-08
DNAJA1	-0,71	2,19E-15
DNAJB1	-0,71	2,64E-06
AKAP7	-0,71	1,56E-10
MGAT5	-0,70	1,44E-13
SKIL	-0,70	7,92E-09
TNKS2	-0,70	1,17E-13
SOCS1	-0,70	1,78E-15
PIK3C2B	-0,70	1,53E-11
GPI	-0,70	1,42E-16
MALT1	-0,70	1,05E-07
RASD1	-0,69	1,78E-11
IRF1	-0,69	8,45E-19
JAK1	-0,69	3,30E-09
PPP3CA	-0,69	5,69E-07
NRAS	-0,69	4,73E-12
NCK1	-0,69	9,54E-10
RAB6A	-0,69	5,48E-15
S1PR4	-0,69	4,52E-15
MTMR6	-0,68	1,35E-08
PLCB3	-0,68	4,63E-10
KLF9	-0,68	1,61E-05
CD44	-0,68	5,67E-12
RC3H2	-0,68	1,88E-08
HMGB1	-0,68	2,10E-16

RAB8B	-0,68	5,56E-08
DNAJB14	-0,67	6,21E-09
NABP1	-0,67	7,70E-09
PHKG2	-0,67	7,83E-05
CSNK1G3	-0,67	6,78E-03
CCND3	-0,67	6,26E-18
RAB29	-0,67	2,29E-06
PRKCH	-0,67	4,18E-07
CREB1	-0,66	1,04E-13
UBASH3A	-0,66	6,56E-14
KMT2E	-0,66	1,36E-09
RGS2	-0,66	8,71E-05
LAG3	-0,66	3,40E-15
PRKAR2A	-0,65	1,46E-07
DNAJA4	-0,65	7,04E-05
PDE4B	-0,65	3,93E-06
HEBP1	-0,65	4,16E-06
ARCN1	-0,65	1,13E-07
YWHAQ	-0,65	4,53E-21
ADD3	-0,64	4,28E-17
CASP8	-0,64	2,67E-07
PRR5	-0,64	1,41E-12
PPARD	-0,64	1,53E-07
PRKACB	-0,64	4,18E-09
IRF2	-0,64	7,12E-07
JUND	-0,64	3,15E-18
ZNF335	-0,63	1,12E-15
EZR	-0,63	7,27E-17
PREX1	-0,63	1,22E-19
DEF6	-0,63	1,60E-13
GRAP2	-0,63	6,47E-12
WIPF1	-0,63	3,41E-18
NCSTN	-0,63	1,15E-07
TNFRSF18	-0,63	3,66E-09
SEMA4D	-0,63	1,99E-12
PPP2R2B	-0,62	6,01E-11
ISG15	-0,62	2,86E-06
PPP1R16B	-0,62	1,06E-09
EP300	-0,62	3,41E-05
PGAP3	-0,62	2,95E-07
MAPK13	-0,61	5,00E-10
PARP4	-0,61	8,42E-06
ANGPT2	-0,61	1,67E-06
CD6	-0,61	7,31E-09
IKZF1	-0,61	7,53E-12

EIF2AK2	-0,61	8,36E-08
GIMAP1	-0,60	1,64E-06
GPR132	-0,60	8,86E-04
EIF3A	-0,60	2,96E-11
ACLY	-0,60	2,90E-03
TOX	-0,60	1,80E-08
ANO6	-0,60	3,90E-05
KPNA6	-0,60	3,67E-09
EIF4G3	-0,60	1,05E-08
LBR	-0,60	1,74E-08
EIF4A2	-0,60	2,55E-14
RAC2	-0,60	2,16E-17
KIDINS220	-0,59	4,81E-07
GSK3B	-0,59	7,23E-04
MAP3K14	-0,59	1,19E-06
EIF3B	-0,59	1,88E-08
DNAJB6	-0,59	1,82E-07
NR4A2	-0,59	2,17E-03
PPP4R1	-0,58	4,67E-07
NABP2	-0,58	3,27E-09
HSPA8	-0,58	4,87E-11
RPS6KA5	-0,58	5,28E-12
STIM2	-0,58	2,13E-07
LY96	0,59	3,11E-12
TNFAIP8L2	0,61	1,12E-05
CARD9	0,68	5,19E-04
AIF1	0,69	3,18E-11
MS4A4A	0,74	1,08E-03
POLR2H	0,81	4,84E-07
NCF2	0,83	4,28E-05
RAB34	0,89	3,32E-08
MGST1	0,89	1,62E-09
FTL	0,92	8,00E-08
PIK3AP1	0,95	1,70E-07
FCER1G	1,02	1,29E-15
PLAU	1,19	3,58E-03

Supplementary Table 8. List of Diseases and Functions deregulated in cluster 2 compared with cluster 0.

Diseases or Functions Annotation	p-Value	Activation z-score	Molecules
Organismal death	1,75E-03	-3,16	VIM,MYL6,HDAC2,CFL1,PTEN,ACTG1,GNB1,ATP5MF,DDX5,KRR1
Leukocyte migration	1,59E-02	-2,38	FASTK,GAS6,CORO1A,HSPA1A/HSPA1B,PTEN,ALOX5AP,RASGRP4,CD48,FCER1G,DEPTOR,NFKBIA,PRDX1,MAPKAPK2,MYH9,CD63,CXCR4,TPT1,S100A10,LRP1,TGFBR1,LAMTOR2,PILRA,SMPD2,JAK2,CD14,NINJ1,EZR,PTPN1,TIMP2,GNB2,EIF2AK4,IL4R,STK4,CTSZ,JUN,SERPINB1,HCLS1,CLEC4E,LGALS1,SOCS3,CSF2RA,G6PC3,LITAF,FCER1A,MTND1,MYO1G,BSG,LY96,GNAI2,GPSM3,CD302,PFN1,CD1D,CD37,ROCK1,UCP2,MPP1,S100A8,GLRX,VASP,CD99,S100A9,FYB1,GNAS,RGS1,UBE2I,CAST,PTPRC,DDT,RAC1
Cell movement of leukocytes	1,98E-02	-2,29	CORO1A,PTEN,RASGRP4,CD48,FCER1G,DEPTOR,PRDX1,MAPKAPK2,MYH9,CD63,CXCR4,S100A10,LRP1,TGFBR1,PILRA,SMPD2,JAK2,CD14,NINJ1,EZR,PTPN1,TIMP2,GNB2,IL4R,STK4,CTSZ,JUN,SERPINB1,HCLS1,LGALS1,SOCS3,CSF2RA,G6PC3,LITAF,FCER1A,MT-ND1,MYO1G,BSG,GNAI2,GPSM3,CD302,PFN1,CD1D,CD37,ROCK1,MPP1,S100A8,VASP,CD99,S100A9,FYB1,GNAS,RGS1,UBE2I,CAST,DDT,RAC1
Cell movement	1,20E-02	-2,23	GAS6,FASTK,CORO1A,HSPA1A/HSPA1B,PTEN,ALOX5AP,RASGRP4,CD48,FCER1G,DEPTOR,NFKBIA,PRDX1,MAPKAPK2,MYH9,CD63,CXCR4,TPT1,S100A10,LRP1,TGFBR1,LAMTOR2,PILRA,SMPD2,JAK2,CD14,NINJ1,EZR,PTPN1,TIMP2,GNB2,EIF2AK4,IL4R,BAX,STK4,CTSZ,JUN,SERPINB1,HCLS1,CLEC4E,LGALS1,SOCS3,CSF2RA,G6PC3,LITAF,FCER1A,MTND1,MYO1G,BSG,LY96,GNAI2,GPSM3,CD302,PFN1,CD1D,CD37,ROCK1,UCP2,MPP1,S100A8,GLRX,VASP,CD99,S100A9,FYB1,GNAS,RGS1,UBE2I,CAST,PTPRC,DDT,RAC1
Binding of leukocytes	8,54E-03	-1,91	GAS6,CORO1A,LGALS1,FCGR1A,TNFSF12,CD48,DEPTOR,PKM,MYO1G,GNAI2,CXCR4,CD37,ROCK1,PRDX2,TGFBR1,PILRA,IL18R1,ADAM9,S100A8,MFNG,GLRX,JAK2,CD14,VASP,NINJ1,S100A9,EZR,FYB1,CALR,RGS1,UBE2I,PTPRC,RAC1,STK4
Adhesion of immune cells	1,20E-02	-1,88	GAS6,CORO1A,FCGR1A,LGALS1,TNFSF12,CD48,PKM,DEPTOR,MYO1G,GNAI2,CXCR4,CD37,ROCK1,TGFBR1,PRDX2,PILRA,ADAM9,S100A8,GLRX,JAK2,CD14,NINJ1,S100A9,EZR,FYB1,CALR,RGS1,UBE2I,PTPRC,RAC1,STK4
Renal cancer	7,96E-03	-1,63	LILRB4,HCLS1,DHRS7,GNB1,ATP5MF,DDX5
Extracranial solid tumor	3,56E-02	-1,63	LILRB4,HCLS1,PAG1,DHRS7,GNB1,ATP5MF,PTPRC,DDX5
Concentration of lipid	3,82E-02	-1,09	S100A8,G6PC3,S100A9,CS,ACAT1,ROCK1,IL4R,PKM
Interaction of mononuclear leukocytes	3,34E-02	-0,85	MFNG,JAK2,CD14,VASP,EZR,CD48,FYB1,DEPTOR,MYO1G,CALR,GNAI2,CXCR4,RGS1,UBE2I,PRDX2,RAC1,STK4,IL18R1
Binding of lymphocytes	1,29E-02	-0,78	MFNG,JAK2,VASP,CD48,EZR,FYB1,DEPTOR,MYO1G,CALR,GNAI2,CXCR4,RGS1,UBE2I,RAC1,STK4,IL18R1
Activation of leukocytes	3,81E-02	-0,70	CORO1A,HSPA1A/HSPA1B,HPS1,PTEN,CD300LF,FCGR1A,CD48,FCER1G,NFKBIA,PRDX1,LILRB4,SLC33A1,TPT1,LRP1,IL18R1,ADAM9,PAG1,TXNIP,CD14,LAPTM5,MGST1,RAB8B,HAVCR2,CALR,EIF2AK4,IFI35,IL4R,BCLAF1,CD163,BAX,PSMB9,PSMB8,MT-CYB,RAB33A,LGALS1,TNFSF12,FKBP1A,PSME2,IRAK3,LY96,DUSP1,CD1D,CD37,CD1E,UPF2,HDAC9,S100A8,NFKB1,MFNG,VAMP8,MBP,OSTM1,DUSP22,CRY1,PHB2,PTPRC
Binding of mononuclear leukocytes	3,13E-02	-0,63	MFNG,JAK2,VASP,CD48,EZR,FYB1,DEPTOR,MYO1G,CALR,GNAI2,CXCR4,RGS1,UBE2I,PRDX2,RAC1,STK4,IL18R1
Activation of mononuclear leukocytes	2,71E-02	-0,63	CORO1A,HSPA1A/HSPA1B,HPS1,PTEN,LGALS1,TNFSF12,CD48,FKBP1A,FCER1G,NFKBIA,PRDX1,IRAK3,LILRB4,LY96,SLC33A1,DUSP1,TPT1,CD1D,CD1E,HDAC9,IL18R1,ADAM9,PAG1,NFKB1,VAMP8,MFNG,TXNIP,MBP,CD14,LAPTM5,MGST1,HAVCR2,DUSP22,CRY1,CALR,EIF2AK4,PHB2,BCLAF1,IL4R,PTPRC,CD163,BAX
Proliferation of immune cells	2,15E-02	-0,61	CORO1A,ITPR2,HSPA1A/HSPA1B,FTL,PTEN,CD48,FCER1G,GADD45B,RABL3,NFKBIA,LILRB4,CXCR4,TPT1,RGS2,TGFBR1,LAMTOR2,ZNHIT1,CD300C,RELT,ELOB,PAG1,TXNIP,JAK2,CD14,LAPTM5,EZR,HAVCR2,CALR,EIF2AK4,IL4R,BCLAF1,ELAVL1,TALDO1,STK17B,SLC39A10,POU2F2,STK4,PMAIP1,CTSZ,JUNB,HCLS1,MARCHF7,LGALS1,SOCS3,FKBP1A,RNF7,BSG,VIM,LY96,GNAI2,TSC22D3,DUSP1,CD1D,ISG15,CD37,ARHGDIA,PRDX2,NFKB1,MFNG,MBP,RPS6KA3,S100A9,FYB1,LAMTOR1,DUSP22,NDUFB10,RHOG,BST2,GNAS,VSIG4,GAPT,PTPRC
Proliferation of mononuclear leukocytes	4,08E-02	-0,50	CORO1A,ITPR2,HSPA1A/HSPA1B,FTL,PTEN,CD48,FCER1G,GADD45B,RABL3,NFKBIA,LILRB4,CXCR4,TPT1,RGS2,TGFBR1,ZNHIT1,CD300C,RELT,ELOB,PAG1,TXNIP,CD14,LAPTM5,EZR,HAVCR2,CALR,EIF2AK4,IL4R,BCLAF1,ELAVL1,TALDO1,STK17B,SLC39A10,POU2F2,STK4,PMAIP1,CTSZ,JUNB,HCLS1,MARCHF7,LGALS1,SOCS3,FKBP1A,RNF7,BSG,VIM,LY96,GNAI2,TSC22D3,DUSP1,CD1D,ISG15,CD37,ARHGDIA,PRDX2,NFKB1,MFNG,MBP,FYB1,LAMTOR1,DUSP22,NDUFB10,RHOG,GNAS,VSIG4,GAPT,PTPRC
Proliferation of lymphatic system cells	3,35E-02	-0,40	CORO1A,ITPR2,HSPA1A/HSPA1B,FTL,PTEN,CD48,FCER1G,GADD45B,RABL3,NFKBIA,LILRB4,CXCR4,TPT1,RGS2,TGFBR1,ZNHIT1,CD300C,RELT,ELOB,PAG1,TXNIP,JAK2,CD14,LAPTM5,EZR,HAVCR2,CALR,EIF2AK4,IL4R,BCLAF1,ELAVL1,STK17B,SLC39A10,POU2F2,STK4,PMAIP1,CTSZ,JUNB,HCLS1,MARCHF7,LGALS1,SOCS3,FKBP1A,RNF7,BSG,VIM,LY96,GNAI2,TSC22D3,DUSP1,CD1D,ISG15,CD37,ARHGDIA,PRDX2,NFKB1,MFNG,MBP,FYB1,LAMTOR1,DUSP22,NDUFB10,RHOG,GNAS,VSIG4,GAPT,PTPRC
Proliferation of lymphocytes	4,20E-02	-0,40	CORO1A,ITPR2,HSPA1A/HSPA1B,FTL,PTEN,CD48,FCER1G,GADD45B,RABL3,NFKBIA,LILRB4,CXCR4,TPT1,RGS2,TGFBR1,ZNHIT1,CD300C,RELT,ELOB,PAG1,TXNIP,CD14,LAPTM5,EZR,HAVCR2,CALR,EIF2AK4,IL4R,BCLAF1,ELAVL1,STK17B,SLC39A10,POU2F2,STK4,PMAIP1,CTSZ,JUNB,HCLS1,MARCHF7,LGALS1,SOCS3,FKBP1A,RNF7,BSG,VIM,LY96,GNAI2,TSC22D3,DUSP1,CD1D,ISG15,CD37,ARHGDIA,PRDX2,NFKB1,MFNG,MBP,FYB1,LAMTOR1,DUSP22,NDUFB10,RHOG,GNAS,VSIG4,GAPT,PTPRC
Binding of T lymphocytes	9,75E-03	-0,35	MFNG,JAK2,VASP,CD48,EZR,FYB1,DEPTOR,CALR,GNAI2,CXCR4,UBE2I,RAC1,STK4,IL18R1
Degranulation of myeloid cells	1,94E-09	-0,14	PEBP1,CORO1A,HSPA1A/HSPA1B,FTL,PTEN,PGAM1,FCGR1A,RASGRP4,FCER1G,PKM,HSPA6,LILRB4,CD63,CYSTM1,LAMTOR2,FABP5,RAP2C,CTSD,A1BG,VPS35L,CD14,CSTB,MGST1,HAVCR2,TIMP2,CYB5R3,ARMC8,VAMP7,IL4R,HLA-C,CYBA,JUNB,CTSZ,DYNLL1,SERPINB1,CD59,PRDX4,APAF1,VAMP2,FCER1A,PSMD11,PSMB1,RAB5C,DUSP1,ROCK1,S100A11,CREG1,S100A8,NFKB1,VAMP8,TUBB,CSNK2B,TRAPPC1,S100A9,LAMTOR1,ACAA1,SERPINB6,RHOG,PDAP1,BST2,DYNLT1,CORO1B,PSMD13,PTPRC,CFD,COTL1,RAC1,ARHGAP9
Degranulation of phagocytes	2,88E-09	-0,14	PEBP1,CORO1A,HSPA1A/HSPA1B,FTL,PTEN,PGAM1,FCGR1A,RASGRP4,FCER1G,PKM,HSPA6,LILRB4,CD63,CYSTM1,LAMTOR2,FABP5,RAP2C,CTSD,A1BG,VPS35L,CD14,CSTB,MGST1,HAVCR2,TIMP2,CYB5R3,ARMC8,VAMP7,IL4R,HLA-C,CYBA,JUNB,CTSZ,DYNLL1,SERPINB1,CD59,PRDX4,APAF1,FCER1A,PSMD11,PSMB1,RAB5C,DUSP1,ROCK1,S100A11,CREG1,S100A8,NFKB1,VAMP8,TUBB,CSNK2B,TRAPPC1,S100A9,LAMTOR1,ACAA1,SERPINB6,RHOG,PDAP1,BST2,DYNLT1,CORO1B,PSMD13,PTPRC,CFD,COTL1,RAC1,ARHGAP9
Degranulation of mast cells	5,14E-03	-0,14	PEBP1,CORO1A,VAMP8,PTEN,FCGR1A,RASGRP4,FCER1G,FCER1A,HAVCR2,LILRB4,CD63,DUSP1,CORO1B,IL4R,PTPRC,JUNB
Inflammation of organ	3,66E-04	0,00	JUN,MBP,IFI30,JPT1,LGALS1,SOCS3,RBPJ,H3-3A/H3-3B,FCER1G,LSM1,NFKBIA,FCER1A,CXCR4,DUSP1,PHF1,IFI44,HLA-DRB5,S100A10,ELAVL1,TGFBR1,S100A11,JUNB
Degranulation	2,73E-09	0,04	GAS6,PEBP1,CORO1A,HSPA1A/HSPA1B,FTL,PTEN,PGAM1,FCGR1A,RASGRP4,FCER1G,PKM,HSPA6,LILRB4,CD63,CYSTM1,LAMTOR2,FABP5,RAP2C,CTSD,A1BG,VPS35L,CD14,CSTB,MGST1,HAVCR2,TIMP2,CYB5R3,ARMC8,VAMP7,IL4R,HLA-C,CYBA,JUNB,CTSZ,DYNLL1,SERPINB1,CD59,PRDX4,APAF1,VAMP2,FCER1A,PSMD11,PSMB1,RAB5C,DUSP1,ROCK1,S100A11,CREG1,S100A8,NFKB1,VAMP8,GLRX,TUBB,CSNK2B,TRAPPC1,S100A9,LAMTOR1,ACAA1,SERPINB6,RHOG,PDAP1,BST2,DYNLT1,CORO1B,PSMD13,PTPRC,CFD,COTL1,RAC1,ARHGAP9
Degranulation of leukocytes	4,62E-09	0,11	GAS6,PEBP1,CORO1A,HSPA1A/HSPA1B,FTL,PTEN,PGAM1,FCGR1A,RASGRP4,FCER1G,PKM,HSPA6,LILRB4,CD63,CYSTM1,LAMTOR2,FABP5,RAP2C,CTSD,A1BG,VPS35L,CD14,CSTB,MGST1,HAVCR2,TIMP2,CYB5R3,ARMC8,VAMP7,IL4R,HLA-C,CYBA,JUNB,CTSZ,DYNLL1,SERPINB1,CD59,PRDX4,APAF1,VAMP2,FCER1A,PSMD11,PSMB1,RAB5C,DUSP1,ROCK1,S100A11,CREG1,S100A8,NFKB1,VAMP8,TUBB,CSNK2B,TRAPPC1,S100A9,LAMTOR1,ACAA1,SERPINB6,RHOG,PDAP1,BST2,DYNLT1,CORO1B,PSMD13,PTPRC,CFD,COTL1,RAC1,ARHGAP9

Accumulation of B lymphocytes	3.94E-02	0.15	NFKB1,TSC22D3,CD99,BAD,PTPN1
Viral Infection	5.69E-03	0.43	CLEC4E,GLUL,LY6E,S100A9,NFKBIA,ARL4C,TIMP2,HAVCR2,ZYX,EIF1AY,GSTO1,CD63,CXCR4,EIF2AK4,KPNA4,PTPRC,TALDO1,GAPDH,BAX,ZNF175,MTX1,HERPUD1,CTSZ
Activation of monocytes	5.80E-03	0.45	ADAM9,LILRB4,LY96,CD14,TNFSF12,CD163
Quantity of granulocytes	2.23E-02	0.74	SERPINB1,FASTK,GFUS,CLEC4E,PTEN,DIDO1,LGALS1,SOCS3,G6PC3,IL13RA1,PLCE1,NFKBIA,FCER1A,IRAK3,C1QA,GNAI2,SLC33A1,DUSP1,CD1D,ARHGDIA,PILRA,CD300C,MPP1,S100A8,NFKB1,STK3,MFNG,JAK2,S100A9,LAMTOR1,TIMP2,ARID4A,GNAS,CEBPG,STK4
Quantity of leukocytes	1.77E-02	0.97	FASTK,CORO1A,ITPR2,PTEN,CD300LF,FCGR1A,CD48,PLCE1,FCER1G,TSPO,NFKBIA,PRKACB,PRDX1,MAPKAPK2,WWOX,C1QA,LILRB4,CXCR4,SLC33A1,AFF1,FABP5,TGFBR1,PILRA,CTSD,CD300C,PAG1,TXNIP,JAK2,HDAC3,HAVCR2,TIMP2,BAD,CEBPG,IL4R,BCLAF1,ELAVL1,RGS10,BAX,STK17B,PSMB9,SLC39A10,POU2F2,STK4,PSMB8,SERPINB1,GFUS,HCLS1,CLEC4E,DIDO1,COMT,DNMT1,LGALS1,SOCS3,TNFSF12,G6PC3,IL13RA1,FKBP1A,LITAF,FCER1A,IRAK3,MYO1G,BSG,GNAI2,CD302,DUSP1,CD1D,ISG15,ARHGDIA,PARL,UPF2,N4BP1,CENPX,MPP1,S100A8,NFKB1,STK3,MFNG,MBP,RPS6KA3,MPG,RBPJ,S100A9,FYB1,LAMTOR1,SERPINB6,ARID4A,GNAS,RGS1,VSIG4,GAPT,ACAT1,PTPRC,CSNK2A1,RAC1
Quantity of cells	1.42E-02	1.06	FASTK,CORO1A,ITPR2,PTEN,CD300LF,FCGR1A,CD48,PLCE1,FCER1G,TSPO,NFKBIA,PRKACB,PRDX1,MAPKAPK2,WWOX,C1QA,LILRB4,CXCR4,SLC33A1,AFF1,FABP5,TGFBR1,PILRA,CTSD,CD300C,PAG1,TXNIP,JAK2,HDAC3,HAVCR2,TIMP2,BAD,CEBPG,IL4R,BCLAF1,ELAVL1,RGS10,BAX,STK17B,PSMB9,SLC39A10,POU2F2,STK4,PSMB8,JUNB,SERPINB1,GFUS,HCLS1,CLEC4E,DIDO1,COMT,DNMT1,LGALS1,SOCS3,TNFSF12,G6PC3,IL13RA1,FKBP1A,LITAF,FCER1A,IRAK3,MYO1G,BSG,GNAI2,CD302,DUSP1,CD1D,ISG15,ARHGDIA,PARL,UPF2,N4BP1,CENPX,MPP1,S100A8,NFKB1,STK3,MFNG,MBP,RPS6KA3,MPG,RBPJ,S100A9,FYB1,LAMTOR1,SERPINB6,ARID4A,GNAS,RGS1,VSIG4,GAPT,ACAT1,PTPRC,CSNK2A1,RAC1
Degranulation of granulocytes	1.97E-08		HSPA1A/HSPA1B,FTL,PGAM1,FCER1G,PKM,HSPA6,CD63,CYSTM1,LAMTOR2,FABP5,RAP2C,CTSD,A1BG,VPS35L,CD14,CSTB,MGST1,TIMP2,CYB5R3,ARMC8,VAMP7,HLA-C,CYBA,CTSZ,DYNLL1,SERPINB1,CD59,PRDX4,APAF1,VAMP2,PSMD11,PSMB1,RAB5C,ROCK1,S100A11,CREG1,S100A8,NFKB1,VAMP8,TUBB,CSNK2B,TRAPPC1,S100A9,LAMTOR1,ACAA1,SERPINB6,RHOG,PDAP1,BST2,DYNLT1,PSMD13,PTPRC,CFD,COTL1,RAC1,ARHGAP9
Degranulation of neutrophils	2.34E-08		HSPA1A/HSPA1B,FTL,PGAM1,FCER1G,PKM,HSPA6,CD63,CYSTM1,LAMTOR2,FABP5,RAP2C,CTSD,A1BG,VPS35L,CD14,CSTB,MGST1,TIMP2,CYB5R3,ARMC8,VAMP7,HLAC,CYBA,CTSZ,DYNLL1,SERPINB1,CD59,PRDX4,APAF1,PSMD11,PSMB1,RAB5C,ROCK1,S100A11,CREG1,S100A8,NFKB1,VAMP8,TUBB,CSNK2B,TRAPPC1,S100A9,LAMTOR1,ACAA1,SERPINB6,RHOG,PDAP1,BST2,DYNLT1,PSMD13,PTPRC,CFD,COTL1,RAC1,ARHGAP9
Hypersensitive reaction	1.23E-06		JUN,IFI30,JPT1,LGALS1,H3-3A/H3-3B,FCER1G,FCER1A,CXCR4,DUSP1,PHF1,HLA-DRB5,IL4R,TGFBR1,S100A11,JUNB
Allergy	2.29E-06		JUN,IFI30,JPT1,LGALS1,H3-3A/H3-3B,FCER1G,FCER1A,CXCR4,DUSP1,PHF1,HLA-DRB5,IL4R,TGFBR1,S100A11,JUNB
Dermatitis	2.95E-06		JUN,IFI30,JPT1,LGALS1,H3-3A/H3-3B,FCER1G,FCER1A,CXCR4,DUSP1,PHF1,HLA-DRB5,TGFBR1,S100A11,JUNB
Immediate hypersensitivity	3.64E-06		JUN,IFI30,JPT1,LGALS1,H3-3A/H3-3B,FCER1G,FCER1A,CXCR4,DUSP1,PHF1,HLA-DRB5,IL4R,S100A11,JUNB
Atopic dermatitis	4.58E-06		JUN,IFI30,JPT1,LGALS1,H3-3A/H3-3B,FCER1G,FCER1A,CXCR4,DUSP1,PHF1,HLA-DRB5,S100A11,JUNB
Immune mediated inflammatory disease	1.50E-05		JUN,CLEC4E,GLUL,HSPA1A/HSPA1B,TNRC6B,LGALS1,LY6E,DNAJC4,FCER1G,GADD45B,IL1R2,NFKBIA,PRDX1,FCER1A,VIM,CXCR4,FKBP5,DUSP1,HLADRB5,S100A10,ZNF326,S100A11,ST6GALNAC4,S100A8,IFI30,JPT1,S100A9,H3-3A/H3-3B,CHCHD2,NDUFB10,GNB2,ARF1,UQCRC2,BST2,RGS1,PHF1,IFI44,CEBPG,PTPRC,TALDO1,BAX,TBRG1,STK17B,JUNB,DYNLL1
Rheumatoid arthritis	2.23E-04		CLEC4E,GLUL,HSPA1A/HSPA1B,TNRC6B,LGALS1,DNAJC4,GADD45B,IL1R2,NFKBIA,PRDX1,VIM,CXCR4,FKBP5,DUSP1,S100A10,ZNF326,ST6GALNAC4,S100A8,S100A9,H3-3A/H3-3B,CHCHD2,NDUFB10,GNB2,ARF1,UQCRC2,CEBPG,PTPRC,TALDO1,TBRG1,STK17B,DYNLL1
Inflammation of joint	5.57E-04		CLEC4E,GLUL,HSPA1A/HSPA1B,APAF1,TNRC6B,LGALS1,DNAJC4,GADD45B,IL1R2,NFKBIA,PRDX1,VIM,GNAI2,CXCR4,FKBP5,DUSP1,S100A10,ZNF326,ST6GALNAC4,S100A8,S100A9,H3-3A/H3-3B,CHCHD2,NDUFB10,GNB2,ARF1,UQCRC2,CEBPG,PHB2,PTPRC,TALDO1,TBRG1,STK17B,DYNLL1
Systemic autoimmune syndrome	6.94E-04		CLEC4E,GLUL,HSPA1A/HSPA1B,TNRC6B,LY6E,LGALS1,DNAJC4,GADD45B,IL1R2,PRDX1,NFKBIA,VIM,CXCR4,FKBP5,DUSP1,S100A10,ZNF326,ST6GALNAC4,S100A8,S100A9,H3-3A/H3-3B,CHCHD2,NDUFB10,GNB2,ARF1,UQCRC2,BST2,IFI44,CEBPG,PTPRC,TALDO1,BAX,TBRG1,STK17B,DYNLL1
Rheumatic Disease	7.74E-04		CLEC4E,GLUL,HSPA1A/HSPA1B,APAF1,TNRC6B,LGALS1,LY6E,DNAJC4,GADD45B,IL1R2,PRDX1,NFKBIA,VIM,GNAI2,CXCR4,FKBP5,DUSP1,S100A10,ZNF326,ST6GALNAC4,S100A8,S100A9,H3-3A/H3-3B,CHCHD2,NDUFB10,GNB2,ARF1,UQCRC2,BST2,RGS1,IFI44,CEBPG,PHB2,PTPRC,TALDO1,BAX,TBRG1,STK17B,DYNLL1
Severe acute respiratory syndrome	2.74E-03		GLUL,CLEC4E,S100A9,NFKBIA,ARL4C,TIMP2,ZYX,EIF1AY,CD63,GSTO1,GAPDH,TALDO1,MTX1,CTSZ
Emigration of thymocytes	2.83E-03		CXCR4,IL4R,STK4
Cytotoxicity of peripheral blood natural killer cells	4.75E-03		VASP,CD48,HAVCR2
Degranulation of lysosome	7.30E-03		CORO1A,GLRX,CORO1B
Deficiency of T lymphocytes	8.54E-03		RBPJ,PTPRC
Chemotaxis of hematopoietic progenitor cells	1.44E-02		CXCR4,GNAI2,RGS1,STK4
Polycythemia	1.44E-02		CALR,GNAI2,CD63,JAK2
Autoimmune response	1.44E-02		PTEN,BAX,PTPN1
Cell proliferation of Langerhans cells	1.65E-02		RPS6KA3,LAMTOR2
Distribution of phosphatidylserine	1.65E-02		LGALS1,PTPRC

Emigration of single positive thymocytes	1.65E-02	IL4R,STK4
Generation of filament barbed ends	1.65E-02	VASP,RAC1
Phagocytic capacity of peritoneal macrophages	1.65E-02	G6PC3,ISG15
Presence of thymocytes	1.65E-02	CXCR4,BAX
Juvenile rheumatoid arthritis	1.85E-02	S100A8,HSPA1A/HSPA1B,LGALS1,S100A9,DNAJC4,GADD45B,NFKBIA,NDUFB10,CEBPG,S100A10,PTPRC,TALDO1,ZNF326,TBRG1,DYNLL1
Interaction of CD4+ T-lymphocytes	1.90E-02	CXCR4,UBE2I,FYB1
Sepsis	1.90E-02	NFKB1,CD14,NFKBIA
Adhesion of peripheral T lymphocyte	2.44E-02	CXCR4,FYB1,RAC1
Chemokinesis of leukocytes	2.44E-02	S100A9,CAST,RAC1
Accumulation of Single positive thymocytes	2.65E-02	IL4R,STK4
Concentration of cholesterol ester	2.65E-02	ACAT1,PKM
Ingestion of cells	2.65E-02	CALR,LRP1
Myeloproliferative neoplasm	2.65E-02	CALR,JAK2
Polarization of M1 macrophages	2.65E-02	SOCS3,RBPJ
Proliferation of CD3 positive T-lymphocytes	2.65E-02	LILRB4,PTPRC
Synthesis of 5-hydroxyeicosatetraenoic acid	2.65E-02	ALOX5AP,COTL1
Activation of peripheral blood monocytes	3.83E-02	LY96,CD14
Antibody-dependent cell-mediated cytotoxicity	3.83E-02	CD59,VASP
Chemokinesis of neutrophils	3.83E-02	S100A9,CAST
G2 phase of T lymphocytes	3.83E-02	LGALS1,IL4R
G2/M phase	3.83E-02	LGALS1,IL4R
Transition of double-positive thymocyte	3.83E-02	HDAC2,PTEN
Abnormal function of neutrophils	3.84E-02	SERPINB1,HSPB1,FASTK,MPP1,GNAI2,CD14,G6PC3,RASGRP4,PILRA
Migration of hematopoietic progenitor cells	3.87E-02	GNAI2,CXCR4,IL4R,STK4
Atherosclerosis	3.94E-02	JUN,ACAT1,PKM,MAPKAPK2,JUNB
Function of immune system	4.33E-02	LILRB4,GNAI2,CD300LF,FCGR1A,RASGRP4,FCER1G,FCER1A
Morphology of dendritic cells	4.38E-02	RPS6KA3,LAMTOR2,TGFBR1,STK4,CTSZ
Function of mast cells	4.46E-02	LILRB4,CD300LF,FCGR1A,RASGRP4,FCER1G,FCER1A

Supplementary Table 9. List of Cananonical Pathways deregulated in cluster 2 compared with cluster 0.

Ingenuity Canonical Pathways	-log(p-value)	zScore	Ratio	Molecules
Oxidative Phosphorylation	18,20	-5,49	0,34	ATP5F1C,ATP5MC1,ATP5MC3,ATP5MF,ATP5PO,COX4I1,COX5A,COX7A2,COX7A2L,COX7C,COX8A,CYB5A,MTATP6,MTCYB,MTND1,NDUFA13,NDUFA2,NDUFA4,NDUFA9,NDUFB10,NDUFB11,NDUFB2,NDUFB7,NDUFB8,NDUFB9,NDUFS3,NDUFS5,NDUFS7,NDUFS8,UQCR10,UQCR11,UQCRC2,UQCRH,UQCRQ
fMLP Signaling in Neutrophils	2,56	-3,61	0,12	ARPC1A,ARPC1B,ARPC2,ARPC4,CALM1 (includes others),GNA15,GNAI2,GNAS,GNB1,GNB2,GNG5,ITPR2,NFKB1,NFKBIA,RAC1
Fcγ Receptor-mediated Phagocytosis in Macrophages and Monocytes	3,42	-3,21	0,15	ACTG1,ARPC1A,ARPC1B,ARPC2,ARPC4,EZR,FCGR1A,FYB1,PLD3,PTEN,RAB11A,RAC1,RPS6KB2,VASP
Gαq Signaling	2,09	-2,89	0,11	CALM1 (includes others),GNA15,GNAI2,GNAS,GNB1,GNB2,GNG5,ITPR2,NFKB1,NFKBIA,PLD3,RAC1,RGS18,RGS2,RHOG,ROCK1
Fatty Acid β-oxidation I	2,17	-2,45	0,19	ACAA1,ACAA2,ECHS1,ECI2,HADHB,HSD17B10
Signaling by Rho Family GTPases	2,02	-2,32	0,09	ACTG1,ARPC1A,ARPC1B,ARPC2,ARPC4,CFL1,EZR,GNA15,GNAI2,GNAS,GNB1,GNB2,GNG5,JUN,MYL6,NFKB1,PIP4K2A,RAC1,RHOG,ROCK1,VIM
Remodeling of Epithelial Adherens Junctions	1,77	-2,24	0,13	ACTG1,ARPC1A,ARPC1B,ARPC2,ARPC4,RAB5C,TUBB,ZYX
Opioid Signaling Pathway	1,94	-2,24	0,09	AP2S1,BAD,CALM1 (includes others),CREB3L4,CREB5,GNA15,GNAI2,GNAS,GNB1,GNB2,GNG5,GRINA,ITPR2,NFKB1,NFKBIA,PRKACB,RAC1,RGS1,RGS10,RGS18,RPS6KA3,RPS6KB2
Glycolysis I	2,24	-2,24	0,23	GAPDH,PGAM1,PGK1,PKM,TPI1
Necroptosis Signaling Pathway	1,65	-2,14	0,10	CAPNS1,FKBP1A,GLUL,RBCK1,SLC25A3,SLC25A6,TIMM13,TIMM17B,TIMM23,TIMM8B,TOMM34,TSPO,UBC,VDAC2
Regulation of Actin-based Motility by Rho	2,31	-2,11	0,13	ARHGDIA,ARPC1A,ARPC1B,ARPC2,ARPC4,CFL1,MYL6,PFN1,PIP4K2A,RAC1,RHOG,ROCK1
Ephrin Receptor Signaling	2,26	-2,06	0,10	ARPC1A,ARPC1B,ARPC2,ARPC4,CFL1,CREB3L4,CREB5,CXCR4,GNA15,GNAI2,GNAS,GNB1,GNB2,GNG5,GRINA,ITSN1,JAK2,RAC1,ROCK1
Gluconeogenesis I	1,61	-2,00	0,19	GAPDH,MDH2,PGAM1,PGK1
Tryptophan Degradation III (Eukaryotic)	1,86	-2,00	0,19	ACAA2,ACAT1,HADHB,HSD17B10,PARK7
Glutaryl-CoA Degradation	2,44	-2,00	0,25	ACAA2,ACAT1,HADHB,HSD17B10,PARK7
Isoleucine Degradation I	2,65	-2,00	0,28	ACAA2,ACAT1,ECHS1,HADHB,HSD17B10
CD28 Signaling in T Helper Cells	1,54	-1,90	0,10	ARPC1A,ARPC1B,ARPC2,ARPC4,CALM1 (includes others),FCER1G,HLA-DRB5,ITPR2,JUN,NFKB1,NFKBIA,PTPRC,RAC1
Synaptogenesis Signaling Pathway	2,12	-1,88	0,09	AFDN,AP2S1,ARPC1A,ARPC1B,ARPC2,ARPC4,BAD,CALM1 (includes others),CDK5,CFL1,CLASP2,CREB3L4,CREB5,GPAA1,GRINA,ITSN1,LRP1,PRKACB,RAB5C,RAC1,RPS6KB2,SGTA,STXBP5,VAMP2
Estrogen Receptor Signaling	7,95	-1,62	0,13	ATP5F1C,ATP5MC1,BAD,CFL1,CREB3L4,CREB5,DDX5,EIF2B2,GNA15,GNAI2,GNAS,GNB1,GNB2,GNG5,HDAC3,JAK2,JUN,MED6,MTATP6,MTCYB,MTND1,MYL6,NCOR1,NDUFA13,NDUFA2,NDUFA4,NDUFA9,NDUFB10,NDUFB11,NDUFB2,NDUFB7,NDUFB8,NDUFB9,NDUFS3,NDUFS5,NDUFS7,NDUFS8,NFKB1,PCNA,PELP1,PLCE1,PRKAB1,PRKACB,PTEN,ROCK1,RPS6KB2,UQCRC2
RHOA Signaling	3,04	-1,60	0,14	ACTG1,ARHGAP4,ARHGAP5,ARHGAP9,ARPC1A,ARPC1B,ARPC2,ARPC4,CFL1,EZR,MYL6,PFN1,PIP4K2A,ROCK1
CXCR4 Signaling	1,32	-1,51	0,09	CXCR4,GNA15,GNAI2,GNAS,GNB1,GNB2,GNG5,ITPR2,JUN,MYL6,RAC1,RHOG,ROCK1
Integrin Signaling	1,58	-1,50	0,09	ACTG1,ARF1,ARF3,ARHGAP5,ARPC1A,ARPC1B,ARPC2,ARPC4,CAPNS1,PARVB,PFN1,PTEN,RAC1,RHOG,ROCK1,VASP,ZYX
GNRH Signaling	1,43	-1,39	0,09	CALM1 (includes others),CREB3L4,CREB5,GNA15,GNAI2,GNAS,GNB1,GNB2,GNG5,ITPR2,JUN,MAP3K13,NFKB1,PRKACB,RAC1
IL-8 Signaling	1,81	-1,39	0,09	BAX,CSTB,GNA15,GNAI2,GNAS,GNB1,GNB2,GNG5,IRAK3,JUN,LASP1,NFKB1,NFKBIA,PLD3,RAC1,RHOG,ROCK1,VASP
Interferon Signaling	1,40	-1,34	0,14	BAX,IFI35,ISG15,JAK2,PSMB8
α-Adrenergic Signaling	1,53	-1,34	0,11	CALM1 (includes others),GNA15,GNAI2,GNAS,GNB1,GNB2,GNG5,ITPR2,PRKACB,SLC8A1
Apelin Adipocyte Signaling Pathway	1,33	-1,13	0,11	GNAI2,GPX4,GSTK1,MGST1,MGST2,PRKAB1,PRKACB,RAC1
Inhibition of ARE-Mediated mRNA Degradation Pathway	1,80	-1,13	0,10	CNOT11,CNOT4,MAPKAPK2,PPP2R5E,PRKACB,PSMA1,PSMA7,PSMB1,PSMB8,PSMB9,PSMD11,PSMD13,PSMD4,PSME2,TNFSF12
Glutathione Redox Reactions I	1,68	-1,00	0,20	GPX4,GSTK1,MGST1,MGST2
Glutathione-mediated Detoxification	1,76	-1,00	0,21	GSTK1,GSTO1,MGST1,MGST2
Folate Transformations I	3,06	-1,00	0,44	MTHFD2,MTHFD2L,MTHFS,MTR
Role of NFAT in Regulation of the Immune Response	2,05	-0,90	0,10	CALM1 (includes others),FCER1A,FCER1G,FCGR1A,GNA15,GNAI2,GNAS,GNB1,GNB2,GNG5,HLA-DRB5,ITPR2,JUN,KPNA1,KPNA4,MEF2A,NFKB1,NFKBIA,TNPO1
Cardiac β-adrenergic Signaling	1,56	-0,82	0,10	AKAP9,ATP2A2,GNA15,GNAI2,GNAS,GNB1,GNB2,GNG5,PDE6D,PPP1CA,PPP1R10,PPP2R5E,PRKACB,SLC8A1
Phospholipase C Signaling	1,36	-0,77	0,08	CALM1 (includes others),CREB3L4,CREB5,FCER1G,GNAS,GNB1,GNB2,GNG5,HDAC2,HDAC3,HDAC9,ITPR2,MEF2A,MYL6,NFKB1,PEBP1,PLCE1,PLD3,RAC1,RHOG,RPS6KA3
Apelin Endothelial Signaling Pathway	1,34	-0,71	0,09	CALM1 (includes others),GNA15,GNAI2,GNAS,GNB1,GNB2,GNG5,JUN,MEF2A,NFKB1,PRKAB1,RPS6KB2
Huntington's Disease Signaling	7,62	-0,63	0,15	APAF1,ATP5F1C,ATP5MC1,BAX,CAPNS1,CDK5,CREB3L4,CREB5,CTSD,DNAJB1,GNA15,GNB1,GNB2,GNG5,GPAA1,HDAC2,HDAC3,HDAC9,HSPA1A/HSPA1B,HSPA6,JUN,NCOR1,POLR2C,POLR2F,POLR2H,POLR2J,PSMA1,PSMA7,PSMB1,PSMB8,PSMB9,PSMD11,PSMD13,PSMD4,PSME2,RCOR3,UBC,VAMP2
D-myo-inositol-5-phosphate Metabolism	1,32	-0,58	0,09	ACP5,DUSP1,DUSP23,G6PC3,NUDT14,PIP4K2A,PLCE1,PPP1CA,PPP1R10,PPP2R5E,PTEN,PTPN1,PTPRC,SOCS3,WBP11
Dopamine-DARPP32 Feedback in cAMP Signaling	1,41	-0,58	0,09	ATP2A2,CALM1 (includes others),CDK5,CREB3L4,CREB5,GNAI2,GNAS,GRINA,ITPR2,PLCE1,PPP1CA,PPP1R10,PPP2R5E,PRKACB
NER (Nucleotide Excision Repair, Enhanced Pathway)	3,65	-0,53	0,16	CCNH,COPS4,H3-3A/H3-3B,PCNA,POLE4,POLK,POLR2C,POLR2F,POLR2H,POLR2J,PRIM1,RAD23B,RPA2,RPA3,UBE2I
Cardiac Hypertrophy Signaling	1,77	-0,47	0,09	CALM1 (includes others),EIF2B2,GNA15,GNAI2,GNAS,GNB1,GNB2,GNG5,HSPB1,JUN,MAP3K13,MAPKAPK2,MEF2A,MYL6,PLCE1,PRKACB,RAC1,RHOG,ROCK1,TGFBR1
Ephrin B Signaling	3,10	-0,38	0,17	CFL1,CXCR4,GNA15,GNAI2,GNAS,GNB1,GNB2,GNG5,ITSN1,RAC1,ROCK1
Calcium Signaling	1,73	-0,33	0,10	ATP2A2,CALM1 (includes others),CALR,CREB3L4,CREB5,GRINA,HDAC2,HDAC3,HDAC9,ITPR2,MEF2A,MYH9,MYL6,PRKACB,SLC8A1,TPM4
Protein Kinase A Signaling	1,89	-0,21	0,08	AKAP9,ANAPC11,BAD,CALM1 (includes others),CREB3L4,CREB5,DUSP1,DUSP22,GNAI2,GNAS,GNB1,GNB2,GNG5,H3-3A/H3-3B,ITPR2,MYL6,NFKB1,NFKBIA,PDE6D,PLCE1,PPP1CA,PPP1R10,PRKACB,PTEN,PTPN1,PTPRC,ROCK1,TGFBR1,VASP
Ferroptosis Signaling Pathway	1,76	0,00	0,11	ARF1,ARF3,FTL,GPX4,HMGCR,HSPB1,NCOA4,NFS1,PEBP1,PRKAB1,SLC38A1,VDAC2
Sumoylation Pathway	2,69	0,00	0,14	ARHGDIA,HDAC2,JUN,NFKB1,NFKBIA,PCNA,RAC1,RAN,RHOG,SP100,SUMO1,SUMO3,UBE2I
iNOS Signaling	2,45	0,38	0,17	CALM1 (includes others),CD14,IRAK3,JAK2,JUN,LY96,NFKB1,NFKBIA
Androgen Signaling	3,86	0,38	0,14	CALM1 (includes others),CALR,CCNH,DNAJB1,GNA15,GNAI2,GNAS,GNB1,GNB2,GNG5,H3-3A/H3-3B,ITPR2,JUN,NCOA4,NFKB1,POLR2C,POLR2F,POLR2H,POLR2J,PRKACB
Hypoxia Signaling in the Cardiovascular System	1,77	0,45	0,12	CREB3L4,CREB5,JUN,NFKBIA,PTEN,SUMO1,UBE2I,UBE2J1,UBE2V1
Epithelial Adherens Junction Signaling	1,60	0,53	0,10	AFDN,ARPC1A,ARPC1B,ARPC2,ARPC4,CFL1,PPP2R5E,PRKAB1,PTEN,RAC1,ROCK1,STK3,STK4,TGFBR1
p38 MAPK Signaling	1,85	0,63	0,11	CREB3L4,CREB5,DUSP1,H3-3A/H3-3B,HSPB1,IL1R2,IRAK3,MAPKAPK2,MEF2A,RPS6KA3,RPS6KB2,TGFBR1
IL-1 Signaling	2,13	0,82	0,13	GNA15,GNAI2,GNAS,GNB1,GNB2,GNG5,IRAK3,JUN,NFKB1,NFKBIA,PRKACB
RAN Signaling	2,91	1,34	0,31	KPNA1,KPNA4,RAN,RANBP1,TNPO1
BAG2 Signaling Pathway	3,51	1,34	0,17	HSPA1A/HSPA1B,HSPA6,MAPKAPK2,NFKB1,PSMA1,PSMA7,PSMB1,PSMB8,PSMB9,PSMD11,PSMD13,PSMD4,PSME2
p53 Signaling	1,62	1,41	0,11	APAF1,BAX,GADD45B,HDAC9,HIPK2,JUN,PCNA,PLAGL1,PMAIP1,PTEN
HER-2 Signaling in Breast Cancer	1,42	1,70	0,09	ARF1,ARF3,BAD,COX4I1,COX5A,COX7A2,COX7A2L,COX7C,COX8A,CYB5A,FCER1A,FCER1G,JUN,NFKB1,NFKBIA,NUP133,PTEN,RPS6KB2
RHOGDI Signaling	4,27	1,70	0,14	ACTG1,ARHGAP4,ARHGAP5,ARHGAP9,ARHGDIA,ARPC1A,ARPC1B,ARPC2,ARPC4,CFL1,EZR,GNA15,GNAI2,GNAS,GNB1,GNB2,GNG5,MYH9,MYL6,PIP4K2A,RAC1,RHOG,ROCK1
Sirtuin Signaling Pathway	8,42	3,13	0,15	ATG14,ATP5F1C,ATP5MC1,BAX,GADD45B,H3-3A/H3-3B,JUN,MTATP6,MTCYB,MTND1,NDUFA13,NDUFA2,NDUFA4,NDUFA9,NDUFB10,NDUFB11,NDUFB2,NDUFB7,NDUFB8,NDUFB9,NDUFS3,NDUFS5,NDUFS7,NDUFS8,NFKB1,PGAM1,PGK1,POLR2F,SIRT2,SLC25A6,TIMM13,TIMM17B,TIMM23,TIMM8B,TOMM34,TSPO,UCP2,UQCRC2,VDAC2
Antigen Presentation Pathway	1,31		0,14	CALR,HLA-C,HLA-DRB5,PSMB8,PSMB9
GABA Receptor Signaling	1,37		0,10	AP2S1,GNA15,GNAI2,GNAS,GNB1,GNB2,GNG5,ITPR2,UBC,UBQLN1
Chondroitin and Dermatan Biosynthesis	1,42		0,33	CSGALNACT1,CSGALNACT2
Histidine Degradation III	1,42		0,33	MTHFD2,MTHFD2L
IL-10 Signaling	1,45		0,11	BLVRB,CD14,IL1R2,IL4R,JUN,NFKB1,NFKBIA,SOCS3
Tetrahydrofolate Salvage from 5,10-methenyltetrahydrofolate	1,58		0,40	MTHFD2,MTHFD2L
Clathrin-mediated Endocytosis Signaling	1,60		0,09	ACTG1,AP1S1,AP2S1,AP3S1,ARPC1A,ARPC1B,ARPC2,ARPC4,CSNK2A1,CSNK2B,RAB11A,RAB5C,RAC1,S100A8,UBC
Ketogenesis	1,72		0,27	ACAA2,ACAT1,HADHB
Lysine Degradation II	1,78		0,50	AASDH,AASDHPPT
Lysine Degradation V	1,78		0,50	AASDH,AASDHPPT
Tight Junction Signaling	1,93		0,10	ACTG1,AFDN,CPSF6,GPAA1,JUN,MYH9,MYL6,NFKB1,PPP2R5E,PRKACB,PTEN,RAC1,TGFBR1,VAMP2,VASP
Ascorbate Recycling (Cytosolic)	2,07		0,67	GLRX,GSTO1
Glutamate Receptor Signaling	2,13		0,15	CALM1 (includes others),GLUL,GNB1,GNB2,GNG5,GRINA,PICK1,SLC38A1
Superpathway of Geranylgeranyldiphosphate Biosynthesis I (via Mevalona	2,14		0,27	ACAA2,ACAT1,HADHB,HMGCR
Ketolysis	2,14		0,38	ACAA2,ACAT1,HADHB
Iron homeostasis signaling pathway	2,22		0,12	ATP6V0B,ATP6V0D1,ATP6V1F,ATP6V1G1,CD163,CIAO1,FTL,HAMP,ISCU,JAK2,LRP1,MMS19,NFS1,NUBP2
Apelin Muscle Signaling Pathway	2,28		0,18	GNA15,GNAI2,GNAS,GNB1,GNB2,GNG5,PRKAB1
DNA Methylation and Transcriptional Repression Signaling	2,31		0,20	CHD4,DNMT1,H3-3A/H3-3B,HDAC2,SAP18,SAP30
Mevalonate Pathway I	2,52		0,33	ACAA2,ACAT1,HADHB,HMGCR
G Protein Signaling Mediated by Tubby	2,54		0,19	GNA15,GNAI2,GNAS,GNB1,GNB2,GNG5,JAK2
FAT10 Signaling Pathway	2,69		0,17	PSMA1,PSMA7,PSMB1,PSMB8,PSMB9,PSMD11,PSMD13,PSMD4,PSME2
Protein Ubiquitination Pathway	3,20		0,11	ANAPC11,CUL2,DNAJB1,DNAJB4,DNAJC4,ELOB,ELOC,HLA-C,HSPA1A/HSPA1B,HSPA6,HSPB1,HSPE1,PSMA1,PSMA7,PSMB1,PSMB8,PSMB9,PSMD11,PSMD13,PSMD4,PSME2,TRAP1,UBC,UBE2I,UBE2J1,UBE2V1,USP48
Nucleotide Excision Repair Pathway	3,42		0,24	CCNH,POLR2C,POLR2F,POLR2H,POLR2J,RAD23B,RPA2,RPA3
Phagosome Maturation	4,25		0,15	ATP6V0B,ATP6V0D1,ATP6V1F,ATP6V1G1,CALR,CTSD,CTSZ,DCTN4,DYNLL1,DYNLT1,GPAA1,HLA-C,HLA-DRB5,PRDX1,PRDX2,PRDX5,RAB5C,RAC1,TUBB,VAMP2
Glucocorticoid Receptor Signaling	5,26		0,11	ATP5F1C,ATP5MC1,CCNH,CD163,DUSP1,FCGR1A,FKBP5,H3-3A/H3-3B,HLAC,HLADRB5,HSPA1A/HSPA1B,HSPA6,IL13RA1,IL18R1,IL1R2,IL4R,JAK2,JUN,MTATP6,MTCYB,MTND1,NCOR1,NDUFA13,NDUFA2,NDUFA4,NDUFA9,NDUFB10,NDUFB11,NDUFB2,NDUFB7,NDUFB8,NDUFB9,NDUFS3,NDUFS5,NDUFS7,NDUFS8,NFKB1,NFKBIA,POLR2C,POLR2F,POLR2H,POLR2J,PRKAB1,PRKACB,RAC1,SMARCA2,SUMO1,TGFBR1,TSC22D3,UBE2I,UQCRC2
Mitochondrial Dysfunction	19,90		0,29	ATP5F1C,ATP5MC1,ATP5MC3,ATP5MF,ATP5PO,COX4I1,COX5A,COX7A2,COX7A2L,COX7C,COX8A,CYB5A,CYB5R3,FIS1,GPX4,HSD17B10,MTATP6,MTCYB,MTND1,NDUFA13,NDUFA2,NDUFA4,NDUFA9,NDUFB10,NDUFB11,NDUFB2,NDUFB7,NDUFB8,NDUFB9,NDUFS3,NDUFS5,NDUFS7,NDUFS8,PARK7,PRDX3,PRDX5,TXN2,UCP2,UQCR10,UQCR11,UQCRC2,UQCRH,UQCRQ,VDAC2

Supplementary Table 10. List of DEGs in cluster 0 and cluster 2, related to *Cytoskeletal rearrangement, cell motility and cell-cell interactions* and *Activation state* .

geni	log2FC cluster 0 vs cluster 2	adj p value
HSPA1B	-1,95	2,46E-16
FCER1A	-1,91	2,66E-09
HSPA1A	-1,86	4,05E-13
CXCR4	-1,42	2,02E-17
RGS1	-1,41	2,82E-15
DEPTOR	-1,35	1,44E-12
EIF2AK4	-1,35	2,90E-06
CLEC4E	-1,31	4,66E-03
AFDN	-1,25	1,01E-05
PTEN	-1,21	4,93E-04
ITSN1	-1,13	1,46E-06
FER	-1,11	8,27E-10
CD302	-1,10	7,59E-12
JAK2	-1,07	4,63E-06
PTPRC	-1,06	2,18E-15
IL18R1	-1,04	8,71E-08
ROCK1	-1,02	1,15E-05
STXBP5	-1,01	3,95E-09
CSF2RA	-1,01	7,34E-08
PRKACB	-0,94	6,32E-03
HCLS1	-0,90	2,08E-11
CALM1	0,55	3,80E-04
RPS6KB2	0,84	1,05E-10
ARPC1A	0,85	4,03E-04
SMPD2	0,85	2,40E-03
BAD	0,85	7,47E-08
RAC1	0,86	2,39E-03
AP2S1	0,87	2,16E-11
ACTG1	0,87	5,10E-04
FYB1	0,88	1,18E-05
ARPC1B	0,89	6,12E-08
PKM	0,90	1,50E-05
CAST	0,90	1,67E-04
CREB3L4	0,90	4,86E-03
ADAM9	0,92	1,65E-03
LITAF	0,92	3,94E-04
GAS6	0,92	7,70E-03
RASGRP4	0,92	3,83E-03
PFN1	0,93	4,35E-07
SIGLEC7	0,94	7,51E-03
MT-ND1	0,94	9,28E-08
SOCS3	0,95	9,91E-04
PTPN1	0,96	8,27E-03
PLD3	0,97	5,51E-04
PILRA	0,97	1,13E-04
GPAA1	0,97	2,75E-03
MPP1	0,99	3,78E-04
TGFBR1	0,99	5,74E-03
MFNG	0,99	2,52E-06
CDK5	0,99	9,25E-05
MAPKAPK2	1,00	1,65E-03
TNFSF12	1,02	2,28E-03
SGTA	1,04	9,96E-06
FCER1G	1,04	1,83E-07
TIMP2	1,06	6,54E-06
LRP1	1,08	7,73E-05
IL4R	1,12	2,88E-07
UCP2	1,13	5,62E-03
CALR	1,14	5,34E-06
G6PC3	1,14	4,84E-06
CD63	1,17	1,23E-09
CTSZ	1,22	1,67E-08
MYO1G	1,24	5,39E-04
CALM3	1,32	9,00E-14
ALOX5AP	1,39	5,49E-08
NINJ1	1,43	1,11E-06
PRDX1	1,55	2,16E-12
S100A10	1,58	7,37E-06
FCGR1A	1,81	1,69E-08
CD14	1,96	2,02E-03
LGALS1	2,06	4,17E-06
C1QA	2,50	3,31E-03
S100A9	2,82	1,71E-03
S100A8	2,93	1,65E-03
